# Comparative genomic analysis of *Bacteroides fragilis* from intestinal and extra-intestinal sites

**DOI:** 10.1128/spectrum.00306-26

**Published:** 2026-07-14

**Authors:** Renee E. Oles, Marvic Carrillo Terrrazas, Luke R. Loomis, Simone Zuffa, Maxwell J. Neal, Chia-Yun Hsu, Adriana Vasquez Ayala, Caitlin Tribelhorn, Pedro Belda-Ferre, Jianshu Zhao, MacKenzie Bryant, Jasmine Zemlin, Jocelyn Young, Parambir S. Dulai, William J. Sandborn, Mamata Sivagnanam, David Pride, Karsten Zengler, Pieter C. Dorrestein, Rob Knight, Hiutung Chu

**Affiliations:** 1 Department of Pathology, University of California 8784, San Diego, California, USA; 2 Division of Host-Microbe Systems and Therapeutics, Department of Pediatrics, University of California 8784, San Diego, California, USA; 3 Skaggs School of Pharmacy and Pharmaceutical Sciences, University of California 8784, San Diego, California, USA; 4 Collaborative Mass Spectrometry Innovation Center, Skaggs School of Pharmacy and Pharmaceutical Sciences, University of California 8784, San Diego, California, USA; 5 Department of Bioengineering, University of California 8784, San Diego, California, USA; 6 Division of Gastroenterology, Hepatology and Nutrition, University of California, San Diego and Rady Children’s Hospital, San Diego, California, USA; 7 Division of Gastroenterology, University of California 8784, San Diego, California, USA; 8 Division of Gastroenterology, Northwestern University 205058, Chicago, Illinois, USA; 9 Center for Microbiome Innovation, University of California 8784, San Diego, California, USA; 10 Center for Innovative Phage Applications and Therapeutics (IPATH), University of California 8784, San Diego, California, USA; 11 Center of Advanced Laboratory Medicine (CALM), University of California 8784, San Diego, California, USA; 12 Program in Materials Science and Engineering, University of California 8784, San Diego, California, USA; 13 Shu Chien-Gene Lay Department of Bioengineering, University of California 8784, San Diego, California, USA; 14 Department of Computer Science & Engineering, University of California 8784, San Diego, California, USA; 15 Halıcıoğlu Data Science Institute, University of California 8784, San Diego, California, USA; 16 Hong Kong University of Science and Technology Jockey Club Institute for Advanced Study, Hong Kong University of Science and Technology 58207, Hong Kong, Hong Kong SAR, China; 17 Chiba University-UC San Diego Center for Mucosal Immunology, Allergy and Vaccines (cMAV), University of California 8784, San Diego, California, USA; University of Guelph College of Biological Science, Guelph, Canada

**Keywords:** *Bacteroides fragilis*, pangenome, mGWAS, phylogenetics, microbial genomics

## Abstract

*Bacteroides fragilis,* a key member of the human gut microbiota, contributes to host health by maintaining intestinal homeostasis. Yet, it is also the most frequently isolated anaerobe in clinical infections. These contrasting roles raise questions about the genetic and ecological factors that explain why this common symbiont is disproportionately linked to infection. We analyzed 813 Division I *B. fragilis* genomes, including 147 new isolates from intestinal and extra-intestinal sites. Infection-associated isolates spanned all phylogroups, indicating no pathogenic lineage. We identified 16 phylogroups, distinguished by genes associated with capsule biosynthesis and interbacterial competition. Additionally, differential metabolomic analysis identified 12 metabolites associated with isolation source, while a microbial genome-wide association study uncovered 44 genes enriched in isolates from extra-intestinal sites, providing the first population-scale markers tied to clinical recovery sites. These results do not implicate a pathogenic lineage; instead, they point to associational links between accessory modules and recovery from extra-intestinal sites under permissive host conditions. This work underscores how genomic diversity and ecological context may jointly shape the clinical impact of gut commensals.

**IMPORTANCE:**

*Bacteroides fragilis,* a human gut resident, is paradoxically one of the most frequent anaerobes recovered from bloodstream and abscess infections. The genetic features that enable frequent recovery from extra-intestinal sites remain poorly defined. Using comparative genomic and metabolomic analyses of strains from intestinal and extra-intestinal sources, we show that strains isolated from infections are phylogenetically dispersed rather than restricted to a single lineage. We observe lineage-linked differences in capsule loci and competition systems, which suggests constrained gene flow and lineage-specific adaptation within the gut. Additionally, a subset of genes and metabolites is enriched among extra-intestinal isolates. Together, these findings suggest that extra-intestinal survival among *B. fragilis* strains reflects the interplay between species-wide genomic diversity and permissive host conditions, rather than the emergence of a single pathogenic lineage.

## INTRODUCTION

The human microbiota plays a pivotal role in health and disease, influencing host metabolism and immunity (1, 2). *Bacteroides fragilis,* a prominent member of the human gut microbiome, contributes to these processes by promoting intestinal homeostasis and immune regulation (3–7). In particular, *B. fragilis* is well-documented for its tolerogenic properties, including the induction of regulatory T cells and suppression of inflammation (3, 4). *B. fragilis* also supports host metabolism through fermentation of dietary polysaccharides and utilization of host-derived glycans (8–10), while participating in cross-feeding interactions that stabilize microbial communities (11, 12). Despite these beneficial roles as a gut symbiont, *B. fragilis* is also the most frequently isolated anaerobe in bloodstream infections and intra-abdominal abscesses (13–16). It has been implicated in peritonitis, soft tissue and surgical site infections, and even necrotizing fasciitis, often following trauma or surgery (17–19). While this high recovery rate is often attributed to its presence as a common gut resident, this explanation alone is insufficient, as other *Bacteroides* species are more abundant yet far less frequently linked to infection (15). This discrepancy raises fundamental questions about the genetic and ecological features that make *B. fragilis* disproportionately represented in extra-intestinal infections (20).

Pioneering studies have explored the duality of commensal and pathogenic *B. fragilis* strains (15, 16, 21, 22), yet definitive associations between clinical isolates and specific virulence factors have yet to be established (23–28). Some reports suggest that the *B. fragilis* capsule enhances serum resistance, agglutination, or adherence in strains isolated from abscesses and bloodstream infections (23–25, 29, 30). Moreover, purified capsular material from *B. fragilis* is sufficient to induce abscess formation in animal models (22, 31), supporting its potential as a major virulence factor. However, these same capsular structures also promote immune tolerance and stable colonization in the gut (12, 32). This suggests that infection does not arise from a specialized pathogenic clade but instead from traits that serve dual ecological roles, promoting both commensal persistence and incidental survival outside the gut.

Leveraging whole genome sequences, including 147 newly isolated strains from intestinal and extra-intestinal sites, we present the largest comparative analysis of *B. fragilis* to date. Our results show that infection-derived isolates are distributed across all phylogroups, with no evidence of a lineage-specific pathogenic subset. Instead, we identify strain-level variation in capsule operons, interbacterial competition systems, plasmid-associated loci, and metabolites, alongside mobile elements that may contribute to opportunistic persistence outside the gut. These findings support a model in which *B. fragilis* infections represent ecological accidents: opportunistic events shaped by the species’ expansive accessory genome and the permissive conditions of the host environment.

## RESULTS

### Extensive genetic diversity in *B. fragilis* isolates from intestinal and extra-intestinal sites

**Fig 1:**
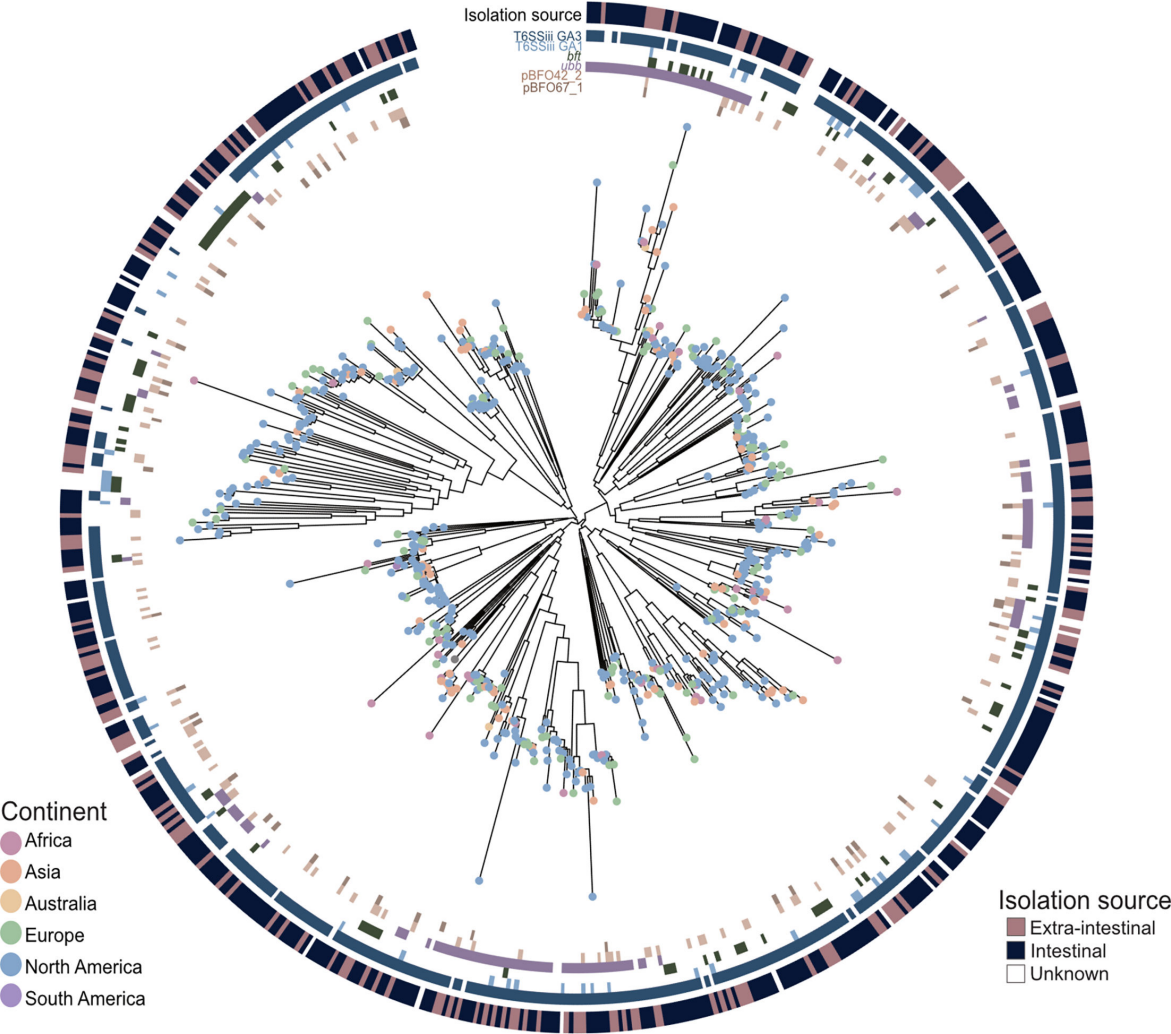
Pangenomic analysis of *Bacteroides fragilis* strains reveals extensive genetic variation. A core genome alignment-derived phylogenetic tree of *B. fragilis* strains by maximum-likelihood annotated with continent of isolate origin (located as dots at the tips), specific phylogroup clusters indicated by the gray inner ring, genetic regions of interest (the six colored rings which signify Type VI secretion system (T6SS), GA1 and GA3, pBFO42_2 and pBFO67_1, plasmid; *ubb*; *bft*, *B. fragilis* toxin with the three isoforms as separate shades of green (*n* = 664; anatomic site PERMANOVA, *P* ≤ 0.001; geography PERMANOVA, *P* ≤ 0.001) with the anatomic site of isolation coloring the outermost ring.

Here, we analyze 813 whole genome sequences, including 147 newly isolated and/or sequenced strains (Table S1) and 666 publicly available genomes (Table S2). These included 510 isolates from the gastrointestinal tract, 221 from extra-intestinal infections, and 82 from unknown origins, spanning 29 countries across 6 continents. Isolates originated from diverse anatomical sites, including feces, intestinal biopsies, blood, and skin infections. To focus exclusively on *B. fragilis* Division I *B. fragilis* strains, we excluded all Division II strains, previously demonstrated by our group and others to represent a distinct genomospecies (16, 33, 34). We further de-duplicated the sample set by removing clusters of genomes with >99.95% identity, resulting in 664 final genomes. We constructed a phylogenetic tree of all 664 isolates from an alignment of 2,349 orthologous core genes, annotated with geographic origins, isolation sites, and key genetic features, including the presence or absence of type VI secretion systems (T6SS GA1 and GA3), the *bft* enterotoxin gene, and select plasmids (Fig. 1). Extra-intestinal isolates were broadly distributed across the phylogeny with modest, non-random distribution by geography and by isolation site (PERMANOVA, Continent *R*² = 0.075, *P* < 0.001; Isolation site *R*² = 0.093, *P* < 0.001). A binary-trait test showed weak but significant phylogenetic signal for isolation site (Fritz & Purvis’ *D* = 0.883; vs random *P* = 0.008; vs Brownian *P* < 0.001). Overall, clustering by isolation site and geography is modest but non-random.

**Fig 2:**
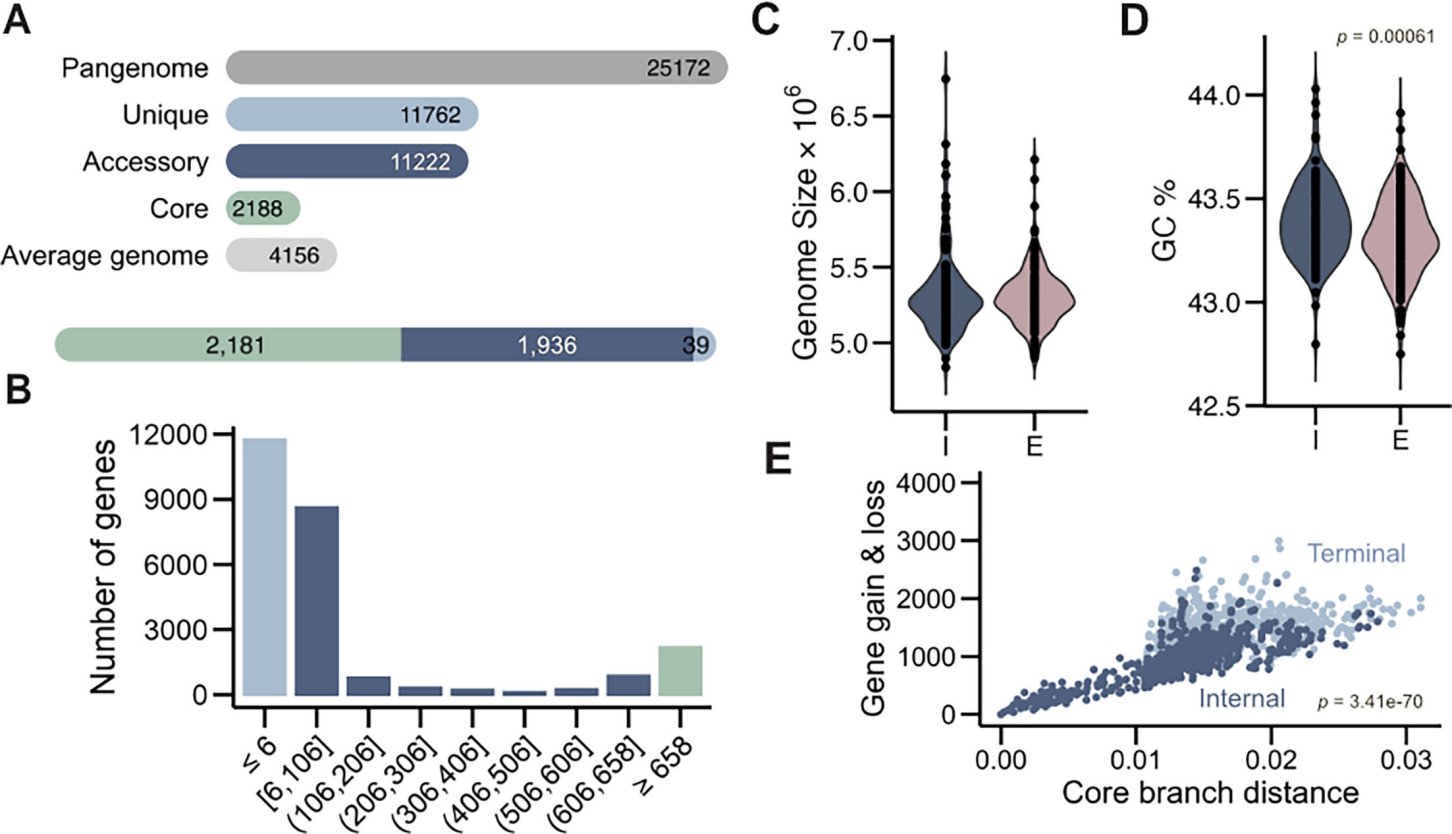
Pangenome structure of *B. fragilis.* (**A**) Pangenome size and composition across 664 isolates. Bars indicate total pangenome, genes unique to ≤1% of isolates (unique), genes present in >1% and <99% (accessory), and genes present in ≥99% (core). The lower bar shows the average genome composition per isolate (core, accessory, and rare/unique gene counts). (**B**) Gene frequency spectrum across the pangenome. Histogram shows the number of genes observed in a given fraction of isolates (bin labels reflect counts out of 664). The rightmost bin (green) denotes core genes (≥658/664 isolates). (**C**) Genome size by isolation source, intestinal (I, *n* = 281) and extra-intestinal (E, *n* = 280), excluding assemblies derived from metagenomic assemblies, Welch’s *t*-test, ns. (**D**) GC content by isolation source, intestinal (I, *n* = 281) and extra-intestinal (E, *n* = 280), excluding assemblies derived from metagenomic assemblies, Welch’s *t*-test, *P* = 0.00061. (**E**) A measure of core compared with accessory genome evolution through cumulative gene gain/loss events (accessory genome) vs cumulative phylogenetic branch distance (core genome) (Student’s *t*-test, *P* = 3.41e^−70^), colored by terminal (light blue) and internal branches (dark blue) using the Panstripe algorithm (35).

To assess genomic diversity, we classified genes as core (2,188 orthologous genes found in ≥99% of strains), accessory (11,222 orthologous genes in <99% and >1%), or unique/strain-specific (11,762 genes present in ≤1% of strains) (Fig. 2A and B). On average, *B. fragilis* genomes contain 4,156 genes, with 52% classified as core and 48% as accessory genes (Fig. 2A). Genome size did not differ significantly between intestinal and extra-intestinal isolates (Fig. 2C) although strains isolated from extra-intestinal sites had a slightly lower GC content (43.4% vs 43.3%, Welch’s *P* = 0.00061) (Fig. 2D). Classifying genes by COG category, most accessory genes have no known COG identification (Fig. S1A). While some COG categories were more abundant in the core genome, no major differences were observed between intestinal and extra-intestinal isolates (Fig. S1B). Gene turnover was evaluated using Panstripe (35), comparing core-genome divergence (phylogenetic branch length) with patterns of gene gain and loss. We observed a significant correlation (*P* = 3.4 × 10⁻^70^), indicating that extensive gene gain and loss events accompany the evolutionary divergence of *B. fragilis* (Fig. 2E). These findings highlight the species’ pronounced genomic plasticity and its capacity to continuously expand its accessory gene pool, a hallmark of bacteria inhabiting diverse, dynamic communities (36).

### Phylogroup-associated genetic signatures in *B. fragilis*

**Fig 3:**
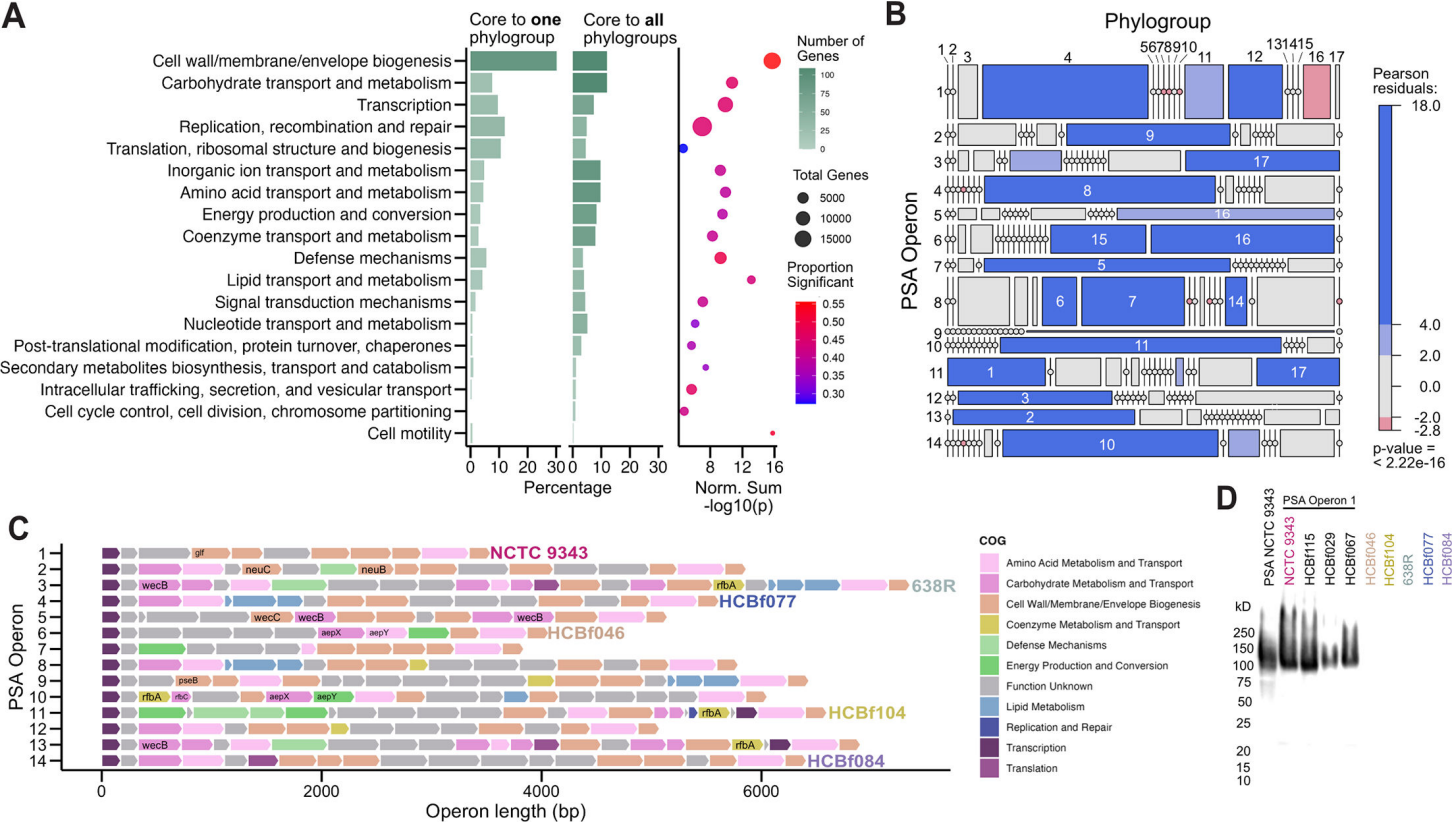
*B. fragilis* phylogroups have unique core-phylogroup genes. (**A**) Left (Bar Plot): The bar plot displays the distribution (percentage) of genes assigned to each COG category for two subsets: (I) genes that are core to exactly one phylogroup and (ii) genes that are core to all 16 phylogroups. Right (Dot Plot): The dot plot shows the normalized sum of −log₁₀(*P*) values, capturing how strongly each COG category is associated with gene presence across different phylogroups (aggregating the significance of multiple differential abundance tests). The bubble size indicates the total number of genes in that COG category, and the bubble color represents the proportion of tests within that category that are statistically significant. (**B**) The mosaic plot illustrates the relationship between polysaccharide operon (PSA) pathways and phylogroups. Each tile corresponds to a specific PSA pathway–phylogroup combination, and the tile’s area is proportional to the observed frequency of that combination. The shading intensity reflects how much the observed frequency deviates from what would be expected if PSA pathway and phylogroup were independent: darker tiles indicate higher‐than‐expected frequencies (positive standardized residuals), whereas lighter or differently shaded tiles indicate lower‐than‐expected frequencies (negative standardized residuals). Phylogroup 10 is missing due to no confirmed isolate information for PSA. (**C**) PSA operon structure of *B. fragilis* strains of high-quality assemblies (*n* = 262). Genes colored by COG category and annotated with a gene name if available by Bakta annotation. (**D**) Western blot analysis of PSA of *B. fragilis* strains representing six PSA operon structures. Rabbit anti-PSA antibody was raised against NCTC 9343, which represents PSA operon 1.

To assess the diversity between genome clusters, we used a full genome, *k*-mer-based clustering approach, grouping isolates into 17 broad groups (hereafter “phylogroups”; Table S3) (37). Consistent with the phylogenetic analysis, isolates from extra-intestinal infections were distributed across phylogroups (Fisher’s exact *P* = 0.33, Fig. S2D), indicating that no single clade is exclusively associated with invasive or pathogenic potential. We next identified genes with phylogroup-specific distributions by analyzing core genes in each phylogroup (present in ≥95% of isolates within a group) and comparing gene abundance across phylogroups. Genes shared across all phylogroups showed a relatively even distribution across COG categories (Fig. 3A). In contrast, genes core to only one phylogroup were enriched for membrane-related functions (25.7% vs 11.4% in universal core genes), particularly capsular polysaccharide genes (Fig. 3A).

Polysaccharide A (PSA), one of eight *B. fragilis* capsular polysaccharides, is the most extensively studied and well known for its potent immunomodulatory activity in the gut, but it can also drive abscess formation following intestinal translocation (3, 6, 22, 31, 38–41). While structural variation in PSA has been described in select laboratory strains (42, 43), our analysis identified 14 distinct PSA operon configurations (Table S5) that strongly correlate with phylogroup as well as with the core genome phylogenetic structure (*χ*², BH *P* = 2.2 × 10⁻^308^, PERMANOVA BH *P* ≤ 1.5 × 10⁻⁴ Fig. 3B and C; Fig. S2E and F, Table S5). These operons share conserved start (*upaY, upaZ*) and end (*wcfS*) genes (31, 42) but vary in their intermediary genes (Fig. 3C). Notably, the canonical PSA operon 1 from the type strain NCTC 9343 was present in only 16.6% (80 out of 481) of the strains examined. Western blot analysis confirmed antigenic specificity, as antibodies raised against NCTC 9343 PSA reacted only with strains carrying PSA operon 1 (Fig. 3D). Importantly, because *B. fragilis* CPS loci are regulated by phase-variable invertible promoters and shufflons, locus presence and configuration reflect genotypic capacity rather than constitutive expression. Consistent with this, our Western blot analysis serves as antigen specificity checks rather than a global survey of CPS expression. Similar phylogroup-linked variation was observed across other capsular polysaccharides (PSB–PSH) (Fig. S3), with capsular polysaccharide configurations significantly associated with phylogroup (*χ*², BH *P* ≤ 9.8 × 10⁻^47^, PERMANOVA BH *P* ≤ 1.5 × 10⁻⁴) (Fig. S2E, Table S5).

Beyond capsule loci, several genes were unevenly distributed across phylogroups. The *B. fragilis* toxin (*bft*) gene was detected in all phylogroup 1 isolates in our data set and showed a strong non-random distribution (*χ*², BH *P* = 9.39 × 10^−23^). Its distribution was also non-random with respect to the phylogeny (PERMANOVA on patristic distances, BH *P* ≤ 6 × 10⁻⁴; Fig. 1). In contrast, the T6SS-GA3 locus (BF9343_1919–1925, 1931, 1940–1943) was absent from phylogroup 1 and was significantly depleted relative to other phylogroups (*χ*², BH *P* = 2.63 × 10^−62^; PERMANOVA, BH *P* ≤ 6 × 10⁻⁴; Fig. 1) (44–49). The *B. fragilis* ubiquitin homolog BfUbb (BF9343_3779), has been shown to mediate antagonism among *B. fragilis* strains (50, 51), was core in phylogroup 8, frequent in phylogroups 17 and 4 (~80%), and rare elsewhere (<15%) (*χ*², BH *P* = 3.42 × 10^−77^; PERMANOVA, BH *P* ≤ 6 × 10⁻⁴; Fig. 1). Together, these findings demonstrate that certain host-associated and competitive genetic traits are strongly phylogroup-associated, but extra-intestinal isolates are not strongly phylogroup-associated.

### Genetic and metabolic traits associated with extra-intestinal isolation

**Fig 4:**
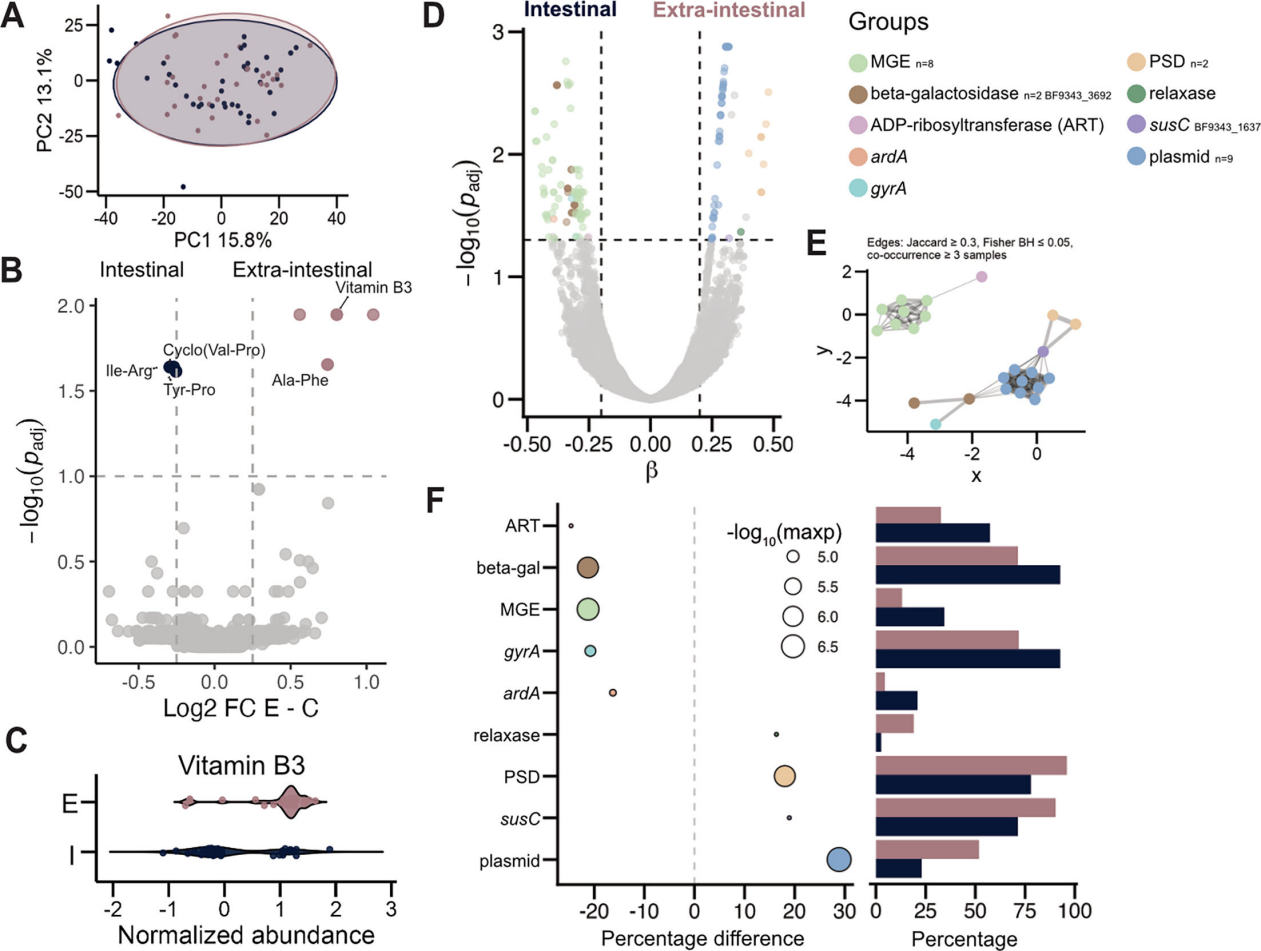
*B. fragilis* strains have features associated with their isolation site. (**A**) PCA plot of media-corrected normalized abundances of metabolites per each isolate (total, *n* = 69; intestinal = 31; extra-intestinal = 38) connected to the centroid of each group, colored by isolation source (PERMANOVA, *P* = 0.992). (**B**) Volcano plot of differentially abundant metabolites of the isolation sources, intestinal (*n* = 40) compared with extra-intestinal (*n* = 33). *P*-values (*y*-axis) are from Wilcoxon tests comparing metabolite levels in intestinal vs extra-intestinal strains (after subtracting media control values). Dashed lines indicate the significance threshold (BH-adjusted *P* ≤ 0.1) and a log₂(fold-change) of 0.25. (**C**) Violin plot of vitamin B3 normalized abundance (normalized to BHI-S control) per intestinal and extra-intestinal strains. (**D**) mGWAS volcano plot of unitigs associated with isolation source (*n* = 289 isolates; MAGs excluded). Points are colored by the locus group assigned from genome context (legend). Dashed lines indicate *β* = ±0.25 and Holm-adjusted *P* ≤ 0.05. (**E**) Co-occurrence network of significant genes from assigning unitigs from panel D to genes in the pangenome. Nodes are colored as in D; edges indicate that two genes co-occur in the same isolates more often than expected (Jaccard ≥ 0.3, Fisher *P* ≤ 0.05), requiring co-occurrence in ≥3 samples. (**F**) Unitigs grouped into locus, showing prevalence by source. Right: fraction of I (navy) and E (rose) isolates carrying each highlighted locus. Left: percent-difference (I–E) in prevalence; point size scales with –log10(max adjusted p) across association tests and colors match locus groups. (*n* = 289 isolates; MAGs excluded.)

We next examined whether *B. fragilis* strains isolated from extra-intestinal sites exhibit distinct metabolic capabilities compared to those from intestinal sites. Growth profiling of 72 isolates in nutrient-rich media revealed no significant differences between intestinal and extra-intestinal strains (Fig. S4A). Untargeted LC-MS/MS analysis of 84 *B. fragilis* strains (intestinal, *n* = 38; extra-intestinal, *n* = 31; unknown, *n* = 18) likewise showed no overall clustering by isolation source (PERMANOVA, *P* = 0.992; Fig. 4A), indicating that broad metabolic reprogramming is not a defining feature of extra-intestinal isolates. Nevertheless, 12 metabolic features were differentially produced between strains from intestinal and extra-intestinal sites (Fig. 4B, Wilcoxon rank-sum test, adjusted *P* ≤ 0.05). Vitamin B3 (niacin/nicotinamide) was enriched in the *in vitro* metabolite supernatants of extra-intestinal isolates relative to intestinal isolates under the BHI-S growth condition, along with the dipeptide Ala–Phe, whereas proline- and arginine-containing dipeptides (Tyr–Pro, Ile–Arg) were higher in intestinal isolates. These differences were modest in magnitude, consistent with the idea that extra-intestinal persistence is not driven by wholesale metabolic reprogramming. Still, the enrichment of vitamin B3, a NAD precursor and central to redox metabolism (52), may reflect a potential stress-response link. Future work will be needed to test this hypothesis through targeted validation of vitamin B3 production and examination of potential genomic or regulatory loci that might underlie its variation.

We tested whether specific genomic features tracked with the recovery site using a unitig microbial genome wide association study (mGWAS) on non-MAG isolate genomes (*n* = 287), with a tree-derived kinship matrix to control for population structure. The QQ plot showed modest inflation with clear tail enrichment (λGC ≈ 1.23; Fig. S4B). At Holm-adjusted *P* ≤ 0.05 and |*β*| ≥ 0.25, we detected 167 significant unitigs mapping to 23 genes (Fig. 4D through F; Table S7). These signals collapsed into nine ≤15 kb loci with within-locus co-occurrence supporting functional grouping (Fig. 4E). A small cassette enriched in intestinal isolates contained mobility/replication features (e.g., a tRNA-Val–adjacent replicon/CTn segment with CRISPR-adjacent and DNA-replication genes). Furthermore, plasmid-associated genes (pBFO42_2 and pBFO67_1), more prevalent in extra-intestinal isolates, carry toxin–antitoxin maintenance systems and a predicted poly-galacturonosidase. Additionally, components of the capsular polysaccharide PSD were associated with extra-intestinal isolates. Because many of these loci were plasmid-borne or otherwise mobile, linkage among mobile genes may underlie some of the observed associations. Therefore, these observations are best interpreted as associative patterns rather than deterministic traits. Significant loci were distributed across the phylogenetic tree rather than restricted to a single lineage (Fig. S4C), and hits tended to cluster on mobile elements. Together, these patterns suggest that horizontal gene flow, especially plasmid carriage, rather than lineage-restricted factors, is the main correlate of extra-intestinal recovery. Extra-intestinal and intestinal isolates remain broadly similar in core genome and metabolism, with accessory modules potentially influencing persistence following translocation under host permissive conditions.

## DISCUSSION

Our comprehensive comparative genomic analysis of *B. fragilis* strains reveals an open pangenome with extensive accessory gene diversity. We identified many features historically classified as virulence factors, including bacterial secretion systems, capsule, and toxins, are unevenly distributed across lineages and associate more strongly with phylogeny than with infection source (Fig. 1). This pattern argues against a model in which specific pathogenic determinants universally drive extra-intestinal disease. This interpretation aligns with the “niche factor” model, which proposes that many features often labeled as “virulence factors” evolved to support colonization, competition, or persistence across different environmental contexts rather than to cause disease *per se* (53). From this ecological perspective, infection reflects a shift in niche occupancy, an opportunistic outcome when host barriers are compromised. Accordingly, *B. fragilis* strains across the phylogeny appear to carry the genetic potential for extra-intestinal survival, not because of a shared virulence program, but because of a highly plastic accessory genome that equips them to exploit new conditions under permissive host contexts (15, 21, 54–57).

Consistent with the niche factor model, the distribution of the BFT enterotoxin illustrates how *B. fragilis* lineages acquire different accessory genes to fulfill ecological roles. Enterotoxigenic *B. fragilis* (ETBF) strains do not form a distinct monophyletic group (Fig. 1), indicating that the *bft* gene has been acquired independently multiple times across different lineages, consistent with a previous report (58). Of note, all strains in phylogroup 11 harbor *bft* and uniformly lack the GA3 T6SS island. If *bft*+ strains principally occupy low-density niches (e.g., lamina propria) with limited interbacterial competition (44), the maintenance cost of GA3 when not beneficial could select against its retention (49), providing a plausible explanation for the frequent *bft*+/GA3− combination. Consistent with this ecological view, *bft* promotes mucosal colonization and persistence (44, 45), aligning a host-interaction advantage (*bft*) with reduced selection for an antagonism system (GA3) in low-competition settings (46). Variation in colonization-associated features, such as *bft*, T6SS modules, capsule variants, underscores the genomic plasticity that supports robust intestinal colonization. Similarly, our mGWAS analysis revealed that genes enriched in strains from extra-intestinal environments are distributed across the phylogenetic tree, consistent with horizontal acquisition. We identified 23 loci (many plasmid-borne) and 12 metabolites whose frequencies differ by isolation site. Although none is exclusive to clinical isolates, their combined presence is compatible with two non-exclusive explanations—enhanced persistence following epithelial barrier breach and/or improved fitness outside the gut (e.g., oxidative-stress resistance). Although this study focuses on Division I, both Divisions I and II are commonly isolated outside the gut. One plausible hypothesis is that shared oxidative/oxygen-stress tolerance, rather than division-specific factors, facilitates recovery under permissive host conditions.

Our observations support an emerging paradigm that pathogenicity is often a feature of context and gene combinations, rather than distinct lineages. This echoes findings in other species (*S. epidermidis*, *Enterococcus*) (59, 60) and suggests that predicting a microbe’s pathogenic potential may require considering both its pangenome and the host state. Despite differences in accessory gene content, extra-intestinal and intestinal isolates share core genetic and metabolic traits. The recurrent recovery of *B. fragilis* from infections is consistent with a large, variable accessory genome which may contribute to persistence across diverse host contexts, a pattern also suggested for other commensals that are frequently isolated clinically (59–62). These findings highlight the limitations of binary classifications such as “commensal” or “pathogen” and underscore the importance of host contexts and ecological opportunity in shaping infectious outcomes (58, 63–65). As such, reassessing how we define and predict pathogenicity across the microbiome is critical for understanding, and ultimately mitigating, the clinical impact of opportunistic infections.

## MATERIALS AND METHODS

### Bacterial strains and culture conditions

Bacterial strains are described in Table S1. *Bacteroides fragilis* strain NCTC9343 was obtained from the American Type Culture Collection (ATCC). *Bacteroides fragilis* strains were grown anaerobically (10% H_2_, 10% CO_2_, 80% N_2_; Coy Lab Products) at 37°C in brain heart infusion (BHI) broth (BD Biosciences) supplemented with 5 μg/mL hemin (Sigma) and 0.5 μg/mL vitamin K (Sigma) (BHI-S).

### Sample collection and bacterial isolation

Samples from healthy donors and patients were collected with the approval of the University of California San Diego Institutional Research Board and with written informed consent signed by subjects prior to sample collection. Healthy and IBD strains were collected under IRB #141853, #150675, and #190012, and extra-intestinal *B. fragilis* strains were collected from the UCSD Clinical Microbiology Facility under IRB# 160524. *B. fragilis* strains from healthy donors were generously provided by Dr. Eric J. Alm (Massachusetts Institute of Technology) (56) and Dr. Laurie Comstock (University of Chicago). The historical *B. fragilis* strains were curated from the laboratory of Dr. Abigail Salyers (University of Illinois at Urbana-Champaign) (66) and provided by Dr. Eric Martens (University of Michigan) (67–69). Public strains were obtained from the NCBI repository for *Bacteroides fragilis* samples. In addition, we analyzed samples from previously published studies (16, 70, 71).

*B. fragilis* strains were isolated from approximately 0.5 g fecal materials, which were homogenized in 30% glycerol/0.1% cysteine, diluted 1:10, and plated in BHI-S with gentamicin (100 µg/mL). Colonies were picked from BHI-S plates, and identities were determined using *Bacteroides* species-specific primers by qPCR (Table S6), followed by confirmation by Sanger sequencing using primers for 16S rRNA, 27F and 1492R. *B. fragilis* strains were banked in glycerol and stored in −80°C for downstream whole genome sequencing and functional studies.

### Metagenomic library preparation and sequencing

*Bacteroides* strains were grown anaerobically for 72 h at 37°C in 1 mL of BHI-S. Nucleic acid extraction was performed with the MagMAX CORE Nucleic Acid Purification Kit (ThermoFisher) (72). Extracted genomic DNA was transferred from 2.0 mL Eppendorf tubes to 96-well plates and then compressed into a 384-well plate. Using 1 µL of each sample, gDNA was quantified using the PicoGreen dsDNA Assay Kit (ThermoFisher Scientific). Based on this quantification, each sample was normalized to 3.5 ng in 5 µL water before generating libraries using a 1:10 Kapa HyperPlus (Roche) miniaturized protocol with barcoded. The libraries were quantified using the PicoGreen dsDNA Assay Kit (Thermo Fisher Scientific), and an equal volume of each sample was pooled using an Echo 550 acoustic liquid handler (Labcyte), PCR cleaned using the QIAquick PCR Purification Kit (QIAGEN), and then size-selected to fragments of 300-700 bp using a PippinHT (Sage Science). The average fragment length was determined using a High Sensitivity D1000 Tapestation (Agilent), and this was used to calculate the average molarity of the pool. This pool was diluted to 90 pM and sequenced with paired-end 150 bp on an iSeq v2 (300 cycle) (Illumina). Raw reads generated from the iSeq were demultiplexed, and new normalized pooling values were calculated based on read counts (73). These new normalized pooling volumes were pooled from the original libraries, PCR cleaned, and again size-selected to fragment sizes of 300–700 bp (73). The iSeq-normalized pool was sequenced on an Illumina NovaSeq 6000 with 2 × 150 bp chemistry at the Institute for Genomic Medicine at UC San Diego.

### Post-sequencing data processing

After raw sequence reads were generated from the NovaSeq, adapter trimming was performed by Fastp (74). Human filtering was performed by Minimap2 (75) by alignment to one database containing human reference genome GRCh38 and PhiX, and a second database containing human reference genome CHM13 (76, 77). Resulting FASTQ were uploaded into Qiita (78) study ID #14360 (https://qiita.ucsd.edu/public/?study_id=14360) or at EBI under accession numbers PRJEB76295 and ERP160853. FASTQ files were further quality controlled via Fastp (74).

### *De novo* assembly, assembly filtering, and annotation

Assembly, quality control, pangenomic analysis, and genome-wide association studies were conducted with the package Panpiper (79). Briefly, draft assemblies were assembled using Shovill v1.1.0 (80) (parameters --nocorr). Shovill filtered contigs with low coverage (<2×). Contigs were also filtered using BBMAP v38.93 if they were less than 500 bp long (81). Quality control removed assemblies with less than 95% completeness, greater than 5% contamination by Checkm2 (82), less than 95% of reads mapping to *B. fragilis*, and an average nucleotide identity to the reference 9343 *B. fragilis* strain less than 95% by FastANI v1.32 (83). Of the 953 *B. fragilis* strains that passed assembly quality filtering, 140 mapped at approximately 87% sequence identity to the *B. fragilis* type strain NCTC 9343 indicating that they belonged to *B. fragilis* division II (16, 33, 34). Thus, these strains were excluded from further analysis in this study. We also removed strains which were isolated over multiple timepoints from the same host to decrease redundancy in our sample pool. We further de-duplicated the sample set by removing clusters of genomes with >99.95% identity. This filtered the input to 664 samples. Bakta v1.6.0 (84) and EggnogMapper v2.1.11 (85) were used to annotate the assemblies.

### Pangenomic analysis

A pangenome from the annotated files was created using Panaroo v1.2.10 (86) (parameters --remove-invalid-genes --clean-mode strict -a core --core_threshold 0.98 --len_dif_percent 0.98 -f 0.7 --merge_paralogs -t 20 --refind_prop_match 0.5 --search_radius 5000). The pangenome size was estimated using the Infinitely Many Genes model as implemented in Panaroo and the openness of the genome determined through gene gain and loss events compared with the branch lengths in the core genome using Panstripe (35), with significance determined through a Student’s *t*-test. We determined core genes as genes present in ≥99% of *B. fragilis* strains as defined by Panaroo, accessory genes as present in <99% and >1%, and strain-specific genes as genes present in ≤1%.

### Phylogenetics and phylogrouping

Protein-coding genes were clustered into orthologous groups with Panaroo v1.2.10 (86) with a threshold of 98%. Single copy orthologs were aligned with MAFFT, concatenated into a nucleotide supermatrix, and gaps introduced for genes absent in a minority of genomes. The resulting core-gene alignment (2,349 orthologs) was used to infer a maximum-likelihood tree. FastTree2 v2.1.11 (87), IQ-TREE 2 v2.2.0.3 (88), and RAxML v8.2.12 (89) were used to build the phylogenetic tree. Using the distances from the midpoint rooted phylogeny created by RAxML v8.2.12, a distance matrix was created to account for lineage effects in the genome-wide association study. Additionally, to quantify associations between phylogenetic relatedness and metadata, we computed a pairwise phylogenetic distance matrix using patristic (branch-length) distances from the rooted core-genome maximum-likelihood phylogeny. We then performed PERMANOVA with 9,999 permutations to test the effects of isolation source (I vs E) and geography (continent) while controlling for the other term. We assessed the homogeneity-of-dispersion assumption using vegan::betadisper with permutation tests.

To analyze population structure through whole genomes to complement the core-genome phylogeny, we utilized Mash v2.3 (90) to create a distance matrix between the whole genome assemblies of the samples using locality-sensitive hashing (parameters -s 200000, *k*-mer size = 21). We clustered the isolates following the methods described in references 37, 37 analyzing the clusters with both an elbow analysis of within-cluster sum-of-squares and the silhouette criterion. The elbow showed a pronounced inflection at *k* ≈ 8, beyond which additional clusters yielded marginal reductions in WSS. Average silhouette values rose sharply to > 0.30 by *k* = 6 and continued to improve at *k* = 17 (Fig. S2A). Per-sample silhouettes (Fig. S2B) confirmed that most genomes exhibit positive cohesion. Taken together, we retained 17 phylogroups for downstream analyses, acknowledging that a few groups may represent transitional lineages (phylogroups 3 and 16). Whole-genome assemblies of *B. fragilis* isolates were summarized in a matrix where each row represented a unique gene and each column corresponded to a designated phylogroup. Entries in this matrix indicated how many isolates in each phylogroup carried the gene. Analyses were performed in R v4.1.1 (91) using tidyverse (92). We identified all genes core to individual phylogroups defined by 95% or more presence in all isolates of a phylogroup. We then calculated the number of phylogroups in which each gene met this core criterion, allowing us to quantify how widely distributed each gene was across all phylogroups.

### Differential gene abundance analysis in phylogroups

To assess whether each gene’s abundance significantly varied across phylogroups, we constructed a 2×N contingency table for each gene, where the first row contained the presence counts (number of isolates with the gene) and the second row contained the absence counts (i.e., total isolates in each phylogroup minus presence). We then performed Fisher’s test on these tables. Resulting *P*-values were adjusted for multiple comparisons using the Benjamini–Hochberg (BH) procedure. For each gene, the adjusted *P*-values (padj) were incorporated into a master table, facilitating the calculation of summary metrics for various functional categories. Specifically, we grouped genes by their COG labels and computed the proportion of significant genes, mean *P*-values, and other descriptive statistics. We visualized the results with bar plots (e.g., normalized sums of −log10(*P*-values)) and bubble charts reflecting the mean *P*-values, total gene counts, and proportion of significant hits per category. Plots were generated using ggplot2 (93).

### Identification of capsular polysaccharide operons

We filtered the assemblies to the most high-quality assemblies (less than or equal to 50 contigs, *n* = 262), creating a pangenome of just these assemblies using Panaroo as described in the previous section (Table S4). To systematically identify distinct polysaccharide (PS) biosynthesis operon paths across 262 high-quality *B. fragilis* genome assemblies, we analyzed the pangenome graph using NetworkX (94), representing genes as nodes with attributes such as gene name and associated genome identifiers, and connecting nodes with edges denoting gene relationships. A custom Python script was developed to traverse this graph recursively (https://github.com/rolesucsd/Travis.git). Starting from nodes corresponding to conserved PS operon initiation genes, the traversal algorithm explored all possible paths leading to terminal nodes defined by conserved stop genes. At each step, the algorithm tracked a list of genomes shared across the traversed edges, ensuring continuity by only extending paths through neighbors that maintained at least one common genome identifier. When a traversal reached a designated end node within these limits, the complete operon was recorded. These outputs constituted the final set of candidate PS paths, capturing the diversity of PS operon architectures present in the data set. We then assigned specific PS paths to individual isolates and examined associations between PS operon variants, phylogroups, and isolation sources. For each PS group, we generated contingency tables of PS paths vs isolation source and PS paths vs phylogroups. Chi-square tests were conducted on these tables to assess the significance of associations.

Using the vcd package (95, 96), mosaic plots were created for PSA to visually represent the association between PS paths and categorical variables such as isolation source and phylogroup. All statistical analyses and visualizations were executed in R (version 4.1.1) (91) utilizing packages including tidyverse (92) for data manipulation, vcd for mosaic plots (95, 96), and FactoMineR/factoextra (97) for correspondence analysis. This integrative approach allowed for rigorous assignment of PS paths to isolates and comprehensive exploration of their associations with phylogenetic and ecological factors.

### Microbial genome-wide association study

We conducted a bacterial genome-wide association study of unitig presence/absence patterns tested between intestinal vs extra-intestinal sources. Unitigs are variable-length DNA sequences corresponding to maximal non-branching paths in a compacted de Bruijn graph built across the genome set (as implemented in pyseer). For each genome assembly, a unitig is encoded as present (1) if that exact unitig sequence occurs in the assembly and absent (0) otherwise, yielding a binary feature matrix for association testing. Unitig-based GWAS can capture multiple classes of genetic variation, gene presence/absence, SNP/indel haplotypes, and variation in poorly annotated or fragmented/mobile regions. We excluded assemblies from MAGs in the gene presence/absence test as the MAGs have incomplete genomes and have lost any associated plasmids, leading to 289 isolate genomes. The association study was conducted using FastLMM as implemented in Pyseer v1.3.9 (98) using a kinship matrix derived from the phylogenetic tree from the core genome alignment to account for population structure. We applied Holm’s correction to the unitig presence/absence *P*-values using the total number of unitigs present in greater than 5% or less than 95% of genomes as the test count. We mapped significant unitigs to the pangenome graph to identify distinct loci of interest. Significant genes fell into approximately nine clusters of linked genes (each cluster spanning <15 kb). We then examined the co-occurrence of these genes across isolates (Jaccard similarity and Fisher’s test) to confirm that genes within each locus tend to be gained or lost together (Fig. 4E).

### *Bacteroides fragilis* growth assays

*Bacteroides fragilis* strains were grown anaerobically, a single colony was picked for each strain and inoculated in 5 mL of BHI-S broth and grown anaerobically for 18 h. Overnight cultures were subcultured at 1:25 (vol:vol) in fresh BHI-S and grown to OD_600_ of 0.8; 5 μL of this culture was taken and inoculated into a 96-well plate with 200 μL of BHI-S media. Five microliters of the washed bacterial culture was used to inoculate before the plate was read (BioTek) at OD_600_ every 15 min for 48 h. All data were finally processed using the gcplyr (99) and growthcurver (100) packages within Rstudio (101).

### Western blot analysis of polysaccharide A

One hundred microliters of overnight *B. fragilis* culture was spun down at 800 × *g* for 5 min. Bacterial pellets were resuspended in Laemmli Sample Buffer (Bio-Rad) and 1% 2-mercaptoethanol. Samples were incubated at 99°C for 10 min. Electrophoresis was conducted with 4%–20% Tris-Glycine Mini Protein Gel (Invitrogen) and Tris-SDS glycine running buffer at 120 V for 2 h using a Mini Gel Tank (Invitrogen). Transfer to the PVDF membrane was performed overnight at 10 mA. Membrane was blocked 1 h at room temperature (RT) in 5% non-fat dry milk in 1× PBS. Membrane was incubated with a primary antibody (anti-PSA at 1:1,000) for 1 h at RT and washed three times with 0.05% PBS-Tween (Fisher Scientific). Incubation with a secondary antibody (goat anti-rabbit IgG at 1:10,000, Millipore) was performed for 1 h at RT, followed by three washes with 0.05% PBS-Tween. Reaction bands were detected using enhanced chemiluminescence (ECL) (Life Technologies) using Chemiluminescent Western Blot Imager Azure 300 (Azure Biosystems).

### Extraction of bacterial cultures for LC-MS/MS

*Bacteroides fragilis* strains were grown anaerobically for 24 h at 37°C in 500 μL of brain heart infusion broth (BD Biosciences) supplemented with 5 μg/mL hemin and 0.5 μg/mL vitamin K (BHI-S) and stored at −80°C until subsequent analysis.

Samples underwent three freeze-thaw cycles to lyse the cells. A total of 180 μL was collected from each sample and transferred to a deep 96-well plate. Subsequently, 600 μL of LC-MS grade methanol (MeOH) was added to each well. The samples were then covered and sonicated for 10 min. After that, samples were centrifuged for 15 min at 2,000 rpm. Supernatant (200 μL) was then transferred to a shallow 96-well plate to be evaporated in vacuum pressure under centrifugation. The dried samples were then stored at −80°C until LC-MS/MS analysis. On the day of the LC-MS/MS experiment, dried extracts were resuspended in a 50% MeOH/H_2_O solution containing sulfadimethoxine (1 μM) as internal standard. Resuspended samples were then sonicated for 10 min before the analysis.

### LC-MS/MS analysis of culture extracts

Culture extracts (5 µL) were analyzed using a Vanquish ultra high-performance liquid chromatography (UHPLC) system coupled to a Q Exactive quadrupole orbitrap mass spectrometer (Thermo Fisher Scientific, Waltham, MA, USA). A Kinetex C18 column (50 × 2.1 mm, 1.7 mm particle size, 100 Å pore size, Phenomenex, Torrance, USA) was employed with a SecurityGuard ULTRA C18 cartridges (2.1 mm ID; 30°C column temperature). The mobile phases (0.5 mL/min flow rate) were 0.1% formic acid in both water (A) and acetonitrile (B) with the following gradient: 0–1 min 5% B, 1–7 min 5%–100% B, 7–7.5 min 100% B, 7.5–8 min 100%–5% B, 8–10 min 5% B. Mass spectrometer was operated under the data-dependent acquisition mode with positive heated electrospray ionization. The source parameters were sheath gas flow, 53 AU; auxiliary gas flow, 14 AU; sweep gas flow, 3 AU; auxiliary gas temperature, 400°C; spray voltage, 3.5 kV; inlet capillary temperature, 269°C; S-lens level, 50 V. For data-dependent acquisition, MS1 scan was performed at *m*/*z* 100–1,500 with the following parameters: resolution, 35,000 at *m*/*z* 200; maximum ion injection time, 100 ms; automated gain control (AGC) target, 5.0E5. For MS/MS acquisition, up to 5 MS/MS spectra per MS1 scan were recorded with the following parameters: resolution, 35,000 at *m*/*z* 200; maximum ion injection time, 100 ms; AGC target, 5.0E5; MS/MS precursor isolation window, *m*/*z* 3; isolation offset, *m*/*z* 0.5; normalized collision energy, a stepwise increase from 20% to 30% to 40%; minimum AGC for MS/MS spectrum, 5.0E3; apex trigger, 2 to 15 s; dynamic precursor exclusion, 10 s. Obtained the raw data (.raw) was converted to .mzML format using MSConvert.

### Metabolite annotation

Raw data and mzML files are deposited in the MassIVE data repository (http://massive.ucsd.edu) under the accession number MSV000090088. Feature extraction was performed using MZmine v3.2.8 (102). The data were filtered by removing all MS/MS fragment ions within ±17 Da of the precursor *m*/*z*. MS/MS spectra were window filtered by choosing only the top 6 fragment ions in the ±50 Da window throughout the spectrum. A feature-based molecular network (FBMN) (103) was then created setting precursor ion mass tolerance to 0.02 Da and a MS/MS fragment ion tolerance of 0.02 Da. Edges were filtered to have a modified cosine score above 0.7 and more than 5 matched peaks. Furthermore, edges between two nodes were kept in the network if and only if each of the nodes appeared in each other’s respective top 10 most similar nodes. Finally, the maximum size of a molecular family was set to 100, and the lowest scoring edges were removed from molecular families until the molecular family size was below this threshold.

The MZmine parameters were as follows—mass detection (parameters: MS1 noise level 3E5, MS2 noise level 0), ADAP chromatogram builder (parameters: minimum group size in number of scans 4, group intensity threshold 9E5, minimum highest intensity 3E6, 0.005 *m*/*z* tolerance or 10 ppm, retention time 0.88–7.5 min), local minimum resolver (parameters: chromatographic threshold 85%, minimum search range 0.05, minimum relative height 0.0%, minimum absolute height 1.0E3, min ratio of peak top/edge 1.7, peak duration range [min/mobility] 0–2.00, min number of data points 4), isotopic peaks grouper (*m*/*z* tolerance 0.01 *m*/*z* or 10 ppm, RT tolerance 0.30 min, maximum charge 5), join aligner (*m*/*z* tolerance 0.01 *m*/*z* or 10 ppm, weight for *m*/*z* 3, RT tolerance 0.10 min, weight for RT 1), gap filler (intensity tolerance 20%, *m*/*z* tolerance 0.001 *m*/*z* or 5.00 ppm, RT tolerance 0.1 min, minimum data points 2).

The spectra in the network were then searched against GNPS2’s spectral libraries (104). The library spectra were filtered in the same manner as the input data. All matches kept between network spectra and library spectra were required to have a score above 0.7 and at least 6 matched peaks. The final annotation table produced 2,754 features of which the GNPS had 413 level 2 annotations.

### Metabolite analysis

The annotations and hits per sample were analyzed in R v4.1.1 (91). A cluster of samples (*n* = 29) were found which were grown in a different batch of BHI media, and these samples were removed from subsequent analysis resulting in 84 *B. fragilis* isolates (intestinal, *n* = 38, extra-intestinal, *n* = 31, unknown, *n* =18). The samples were normalized through the robust centered log ratio transformation (using the decostand function of the package vegan v2.6) (92), which divides each value by the geometric mean of the observed features before applying the log transformation. Zeros are excluded from this transformation. The samples were corrected for the media blanks by subtracting the media blanks from the sample counts. We created a PCA plot of metabolites produced by the prcomp function of the normalized, corrected metabolite abundances. Violin plots were created using ggplot2 of media-corrected normalized metabolite production (93). The Wilcoxon rank-sum test was conducted to determine differential abundance of media-corrected and normalized metabolomics features between strains from extra-intestinal and intestinal sites. We determined significance with a Benjamini, Hochberg adjusted *P*-value 0.05 and log2-fold change 0.25. We calculated differential abundance of metabolites in all strains against the media controls with a student’s *t*-test with a significance cutoff of a Benjamini, Hochberg adjusted *P*-value 0.1 and log2-fold change 0.25. We created a heatmap in R with Euclidean clustering of both rows (metabolites) and columns (isolates).

## SUPPLEMENTAL MATERIAL

Fig. S1 spectrum.00306-26-s0001.tiff COG annotation coverage and source-specific functional biases.

Fig. S2 spectrum.00306-26-s0002.tiff *B. fragilis* isolation source distribution among phylogroups and polysaccharide operon-path traits.

Fig. S3 spectrum.00306-26-s0003.tiff Polysaccharide operon structures among *B. fragilis* strains.

Fig. S4 spectrum.00306-26-s0004.tiff Growth phenotypes and mGWAS.

Supplemental material spectrum.00306-26-s0005.docx Legends for all Supplemental figures and tables.

Supplemental tables spectrum.00306-26-s0006.pdf Tables S1 to S7.

ASM does not own the copyrights to Supplemental Material that may be linked to, or accessed through, an article. The authors have granted ASM a non-exclusive, world-wide license to publish the Supplemental Material files. Please contact the corresponding author directly for reuse.
