# Supplementary material for: Comparative genomic analysis of Bacteroides fragilis from intestinal and extra-intestinal sites
Source: Microbiol Spectr. 2026 Jul 14;14(8):e00306-26. doi: 10.1128/spectrum.00306-26 (PMC13435655; doi:10.1128/spectrum.00306-26)
Supplement: Supplementary file 1 [file spectrum.00306-26-s0006.pdf]

TABLE 1

Sample	Chu	Sample_Name	Cohort	Group	Site	City	State	Country
363192542_S33_L001.3	HCBf005	106-16	Chu	Healthy	Fecal			USA
363192558_S49_L001	HCBf007	110-29	AGP	Healthy	Fecal	San_Diego	California	USA
363192566_S57_L001	HCBf008	110-31	AGP	Healthy	Fecal	San_Diego	California	USA
363192598_S89_L001	HCBf012	111-20	AGP	Healthy	Fecal	San_Diego	California	USA
MCT420_32_S40_L002	HCBf013	111-28	AGP	Healthy	Fecal	San Diego	California	USA
363192527_S10_L001	HCBf014	112-3	AGP	Healthy	Fecal	San_Diego	California	USA
MCT420_29_S37_L002	HCBf016	112-15	AGP	Healthy	Fecal	San Diego	California	USA
363192559_S50_L001	HCBf019	S04-0107-6 (Alm)	Alm	Healthy	Fecal	Boston		USA
363192575_S66_L001.1	HCBf021	S02-0001-1 (Alm)	Chu	Healthy	Fecal			USA
363192583_S74_L001	HCBf022	S02-0024-14 (Alm)	Alm	Healthy	Fecal	Boston	Massachusetts	USA
363192591_S82_L001	HCBf023	S04-0006-7 (Alm)	Alm	Healthy	Fecal	Boston	Massachusetts	USA
363192520_S3_L001	HCBf025	S01-0228-1 (Alm)	Alm	Healthy	Fecal	Boston	Massachusetts	USA
363192528_S11_L001	HCBf026	S03-0090-12 (Alm)	Alm	Healthy	Fecal	Boston	Massachusetts	USA
363192539_S19_L001	HCBf027	S05-0002-1 (Alm)	Alm	Healthy	Fecal	Boston	Massachusetts	USA
363192544_S35_L001	HCBf029	S07-0068-16 (Alm)	Alm	Healthy	Fecal	Boston	Massachusetts	USA
363192552_S43_L001	HCBf030	S09-0001-16 (Alm)	Alm	Healthy	Fecal	Boston	Massachusetts	USA
363192560_S51_L001	HCBf031	S10-0039-19 (Alm)	Alm	Healthy	Fecal	Boston	Massachusetts	USA
363192568_S59_L001	HCBf032	S11-0001-18 (Alm)	Alm	Healthy	Fecal	Boston	Massachusetts	USA
363192592_S83_L001	HCBf035	FIT073E3CAL B7	Chu	IBD	Fecal	San_Diego	Califonia	USA
363192600_S91_L001	HCBf036	FIT073E3CAL B8	Chu	IBD	Fecal	San_Diego	Califonia	USA
MCT420_47_S55_L002	HCBf037	FIT073E3CAL B9	Chu	IBD	Fecal	San Diego	California	USA
363192529_S12_L001	HCBf038	FIT073E3CAL B11	Chu	IBD	Fecal	San_Diego	Califonia	USA
363192541_S20_L001	HCBf039	FIT073E3CAL B10	Chu	IBD	Fecal	San_Diego	Califonia	USA
363192577_S68_L001	HCBf045	FIT268E3CAL C1	Chu	IBD	Fecal	San_Diego	Califonia	USA
363192585_S76_L001	HCBf046	FIT268E3CAL C2	Chu	IBD	Fecal	San_Diego	Califonia	USA
363192601_S92_L001	HCBf048	FIT236E3CAL	Chu	IBD	Fecal	San_Diego	Califonia	USA
363192522_S5_L001	HCBf049	FIT025E3CAL P2-G8	Chu	IBD	Fecal	San_Diego	Califonia	USA
363192546_S37_L001.1	HCBf053	FIT053E3CAL	Chu	IBD	Fecal			USA
363192554_S45_L001.2	HCBf054	FIT049E3CAL	Chu	IBD	Fecal			USA
363192578_S69_L001	HCBf057	FIT134E3CAL P1-G2	Chu	IBD	Fecal	San_Diego	Califonia	USA
363192602_S93_L001	HCBf059	Non-IBD Sample C2 B6	Chu	Healthy	Fecal	San_Diego	Califonia	USA

363192594_S85_L001	HCBf060	Non-IBD Sample C2 C5	Chu	Healthy	Fecal	San_Diego	Califonia	USA
363193756_S6_L001	HCBf061	Non-IBD Sample C6 D7	Chu	Healthy	Fecal	San_Diego	Califonia	USA
363193763_S14_L001	HCBf062	Non-IBD Sample C6 E10	Chu	Healthy	Fecal	San_Diego	Califonia	USA
363192524_S22_L001.1	HCBf063	Non-IBD Sample C12	Chu	Healthy	Fecal			USA
363192555_S46_L001	HCBf066	FIT037SC1.C3 A3	Chu	IBD	Fecal	San_Diego	Califonia	USA
363192563_S54_L001	HCBf067	FIT037SC1.C3 C5	Chu	IBD	Fecal	San_Diego	Califonia	USA
363192579_S70_L001	HCBf068	FIT122SC1.E3 D1	Chu	IBD	Fecal	San_Diego	Califonia	USA
363192571_S62_L001	HCBf069	FIT122SC1.E3 E1	Chu	IBD	Fecal	San_Diego	Califonia	USA
363192587_S78_L001	HCBf070	FIT104SC1.E3 B10	Chu	IBD	Fecal	San_Diego	Califonia	USA
363192595_S86_L001	HCBf071	FIT104SC1.E3 C2	Chu	IBD	Fecal	San_Diego	Califonia	USA
363192603_S94_L001	HCBf072	FIT291SC1.E3 A3	Chu	IBD	Fecal	San_Diego	Califonia	USA
MCT420_36_S44_L002	HCBf074	RA44-A5	Chu	IBD	Colon	San Diego	California	USA
363192533_S31_L001	HCBf076	RA24-trans B4	Chu	Healthy	Colon	San_Diego	California	USA
363192548_S39_L001	HCBf077	RA28-trans GS4	Chu	Healthy	Colon	San_Diego	California	USA
363192564_S55_L001	HCBf078	RA30-trans G6	Chu	Healthy	Colon	San_Diego	California	USA
363192556_S47_L001	HCBf079	RA30-trans G8	Chu	Healthy	Colon	San_Diego	California	USA
363192572_S63_L001	HCBf080	RA38-trans GS7	Pediatric biopsy	Healthy	Colon	San_Diego	California	USA
363192588_S79_L001.1	HCBf082	RA44-trans	Chu	IBD	Colon			USA
363192596_S87_L001.1	HCBf083	RA44-trans	Chu	IBD	Colon			USA
363192604_S95_L001	HCBf084	1-PL	Chu	Infection	Drainage	San_Diego	Califonia	USA
363193757_S8_L001	HCBf085	2-PL	Chu	Infection	Foot	San_Diego	Califonia	USA
363193754_S16_L001.2	HCBf086	4-PL	Chu	Infection	Scrotum			USA
MCT420_41_S49_L002	HCBf087	6-PL	Chu	Infection	Fluid	San Diego	California	USA
363192525_S32_L001.2	HCBf088	7-PL	Chu	Infection	Blood			USA
363192557_S48_L001	HCBf090	12-PL	Chu	Infection	Tissue	San_Diego	Califonia	USA
363192573_S64_L001	HCBf092	BAC06	Chu	Infection	Bone	San_Diego	Califonia	USA
363192581_S72_L001	HCBf093	BAC07	Chu	Infection	Swab	San_Diego	Califonia	USA
363192589_S80_L001	HCBf094	BAC09	Chu	Infection	Drainage	San_Diego	Califonia	USA
363192597_S88_L001	HCBf095	S01-0001-1	Chu	Healthy	Fecal	San_Diego	Califonia	USA
363192605_S96_L001	HCBf096	BAC14	Chu	Infection	Swab	San_Diego	Califonia	USA
363193002_S105_L001	HCBf098	BAC18	Chu	Infection	Lesion	San_Diego	Califonia	USA
363193003_S113_L001.4	HCBf099	BAC29	Chu	Infection	Back			USA
363193027_S137_L001	HCBf102	BAC36	Chu	Infection	NA	San_Diego	Califonia	USA

363193035_S145_L001	HCBf103	BAC37	Chu	Infection	Swab	San_Diego	Califonia	USA
363193043_S153_L001	HCBf104	BAC41	Chu	Infection	Swab	San_Diego	Califonia	USA
363193059_S169_L001	HCBf106	BAC44	Chu	Infection	Tissue	San_Diego	Califonia	USA
MCT420_49_S57_L002	HCBf107	BAC45	Chu	Infection	NA	San Diego	California	USA
363193076_S185_L001	HCBf108	BAC60	Chu	Infection	Tissue	San_Diego	Califonia	USA
363192793_S98_L001	HCBf109	BAC52	Chu	Infection	Swab	San_Diego	Califonia	USA
363192998_S106_L001	HCBf110	BAC72	Chu	Infection	Lesion	San_Diego	Califonia	USA
363193004_S114_L001	HCBf111	BAC73	Chu	Infection	Drainage	San_Diego	Califonia	USA
MCT420_35_S43_L002	HCBf112	BAC74	Chu	Infection	Abscess	San Diego	California	USA
363193022_S130_L001.1	HCBf113	BAC75	Chu	Infection	Foot			USA
363193028_S138_L001	HCBf114	BAC77	Chu	Infection	Swab	San_Diego	Califonia	USA
363193036_S146_L001	HCBf115	BAC79	Chu	Infection	Tissue	San_Diego	Califonia	USA
363193044_S154_L001	HCBf116	BAC80	Chu	Infection	Swab	San_Diego	Califonia	USA
363193053_S162_L001	HCBf117	BAC81	Chu	Infection	Aspirate	San_Diego	Califonia	USA
363193060_S170_L001	HCBf118	BAC83	Chu	Infection	Bed Sore	San_Diego	Califonia	USA
363193068_S178_L001	HCBf119	BAC86	Chu	Infection	Aspirate	San_Diego	Califonia	USA
363192794_S99_L001	HCBf121	BAC89	Chu	Infection	Aspirate	San_Diego	Califonia	USA
363192999_S107_L001	HCBf122	BAC92	Chu	Infection	Drainage	San_Diego	Califonia	USA
363193013_S123_L001	HCBf124	VPI-4517	Chu	Unknown	NA		Virginia	USA
363193029_S139_L001	HCBf126	VPI-BF7639	Chu	Unknown	NA		Virginia	USA
363193037_S147_L001	HCBf127	58_3_6	Chu	Unknown	NA			USA
363193045_S155_L001	HCBf128	VPI-5383 / 23745	Chu	Infection	Pleural fluid			USA
363193048_S163_L001	HCBf129	VPI-BF7397	Chu	Unknown	NA		Virginia	USA
363193061_S171_L001	HCBf130	VPI-BF119	Chu	Unknown	NA		Virginia	USA
363193069_S179_L001	HCBf131	VPI-BF7567	Chu	Unknown	NA		Virginia	USA
MCT420_50_S58_L002	HCBf132	VPI-BF8371	Chu	Unknown	NA	San Diego	California	USA
363192795_S100_L001	HCBf133	VPI-3277	Chu	Infection	Blood			USA
363193001_S108_L001	HCBf134	VPI-2553	Chu	Infection	Appendix abscess			USA
363193046_S156_L001	HCBf140	WAL8762	Chu	Infection	NA			USA
363193054_S164_L001	HCBf141	VPI-499	Chu	Unknown	NA		Virginia	USA
363193062_S172_L001	HCBf142	638R	Chu	Infection	Intestine			USA
363193071_S180_L001	HCBf143	WH706	Chu	Healthy	Fecal	Boston	Massachusetts	USA
363193078_S188_L001	HCBf144	WH707	Chu	Healthy	Fecal	Boston	Massachusetts	USA
363193015_S125_L001.1	HCBf148	WAL8916	Chu	Historical	Unknown			USA
363193031_S141_L001.1	HCBf150	VPI-4361	Chu	Infection	Unknown			USA
363193039_S149_L001	HCBf151	VPI-BF8223	Chu	Unknown	NA		Virginia	USA
363193047_S157_L001	HCBf152	WAL8774	Chu	Infection	NA			USA

363193055_S165_L001	HCBf153	WH705	Chu	Healthy	Fecal	Boston	Massachusetts	USA
363193063_S173_L001.2	HCBf154	VPI-2044	Chu					USA
363193072_S181_L001	HCBf155	VPI-BF-AK87	Chu	Unknown	NA		Virginia	USA
363193079_S189_L001	HCBf156	VPI-12256	Chu	Unknown	NA		Virginia	USA
363192797_S102_L001	HCBf157	WH718	Chu	Healthy	Fecal	Boston	Massachusetts	USA
363192805_S110_L001	HCBf158	CL03T00C08	Broad Institute	Healthy	Fecal	Boston	Massachusetts	USA
MCT420_18_S26_L002	HCBf159	YCH46	Chu	Infection	Blood	San Diego	California	USA
363193016_S126_L001.1	HCBf160	VPI-BF8928	Chu					USA
363193021_S134_L001	HCBf161	3_2_5	Chu	Healthy	Fecal	Boston	Massachusetts	USA
363193032_S142_L001	HCBf162	VPI-29765	Chu	Unknown	NA		Virginia	USA
363193040_S150_L001	HCBf163	WAL8790	Chu	Infection	NA			USA
363193049_S158_L001	HCBf164	WH605	Chu	Healthy	Fecal	Boston	Massachusetts	USA
363193056_S166_L001	HCBf165	CL05T00C42	Chu	Healthy	Fecal	Boston	Massachusetts	USA
363193064_S174_L001	HCBf166	VPI-2556I	Chu	Unknown	NA		Virginia	USA
MCT420_79_S87_L002	HCBf167	CL07T12C05	Chu	Healthy	Fecal	San Diego	California	USA
363193073_S182_L001	HCBf167	CL07T12C05	Chu	Healthy	Fecal	Boston	Massachusetts	USA
363193080_S190_L001	HCBf168	CL03T12C07	Brigham & Women's Hospital	Healthy	Fecal	Boston	Massachusetts	USA
MCT420_17_S25_L002	HCBf169	VPI-2552	Historical	Unknown	NA			USA
363192804_S111_L001	HCBf170	3_1_12	Historical	Healthy				NA
363193009_S119_L001	HCBf171	VPI-2627-J2	Historical	Unknown	NA		Virginia	USA
363193017_S127_L001	HCBf172	VPI-3392		Unknown	NA		Virginia	USA
363193025_S135_L001	HCBf173	VPI-4076		Infection	Blood			NA
363193041_S151_L001	HCBf175	VPI-2393		Unknown	NA		Virginia	USA
363193057_S167_L001	HCBf177	VPI-A11-24B	Historical	Unknown	NA		Virginia	USA
363193065_S175_L001	HCBf178	VPI-4117		Unknown	NA		Virginia	USA
363197087_S326_L001.5	HCBf181	Non-IBD Sample C10	Chu	Healthy	Fecal			USA
363191840_S199_L001.1	HCBf182	Non-IBD Sample C17 E5	Chu	Healthy	Fecal			USA
363192044_S216_L001.3	HCBf183	Mayo07 - d5_HBOT	Chu	IBD	Fecal			USA
363192053_S223_L001.4	HCBf184	Non-IBD Sample C21	Chu	Healthy	Fecal			USA
363197048_S313_L001.1	HCBf186	102-27	Chu	Healthy	Fecal			USA
363197054_S337_L001.2	HCBf187	103-27	Chu	Healthy	Fecal			USA
363197065_S315_L001	HCBf189	112-16	AGP	Healthy	Fecal	San_Diego	California	USA

363197078_S309_L001.3	HCBf191	FIT036CCAL	Chu	IBD	Fecal			USA
363197090_S325_L001.1	HCBf192	FIT292E3CAL	Chu	IBD	Fecal			USA
363197827_S378_L001	HCBf194	111-9	AGP	Healthy	Fecal	San_Diego	California	USA
MCT420_2_S10_L002	HCBf209	infant_twin 1	Chu	Healthy	Fecal	San Diego	California	USA
MCT420_10_S18_L002	HCBf213	J665_Rhesus	NHP	Animal	Fecal	San Diego	California	USA
MCT420_52_S60_L002	HCBf216	RA 49	Chu	Healthy	Colon	San Diego	California	USA
MCT420_54_S62_L002	HCBf218	RA 87	Chu	IBD	Colon	San Diego	California	USA
MCT420_55_S63_L002	HCBf219	RA 53	Chu	IBD	Colon	San Diego	California	USA
MCT420_56_S64_L002	HCBf220	RA 56	Chu	Healthy	Colon	San Diego	California	USA
MCT420_58_S66_L002	HCBf222	RA 80	Chu	Healthy	Colon	San Diego	California	USA
MCT420_59_S67_L002	HCBf223	RA 81	Chu	Healthy	Colon	San Diego	California	USA
MCT420_60_S68_L002	HCBf224	RA 52	Chu	Healthy	Colon	San Diego	California	USA
MCT420_63_S71_L002	HCBf227	RA56	Chu	Healthy	Colon	San Diego	California	USA
MCT420_75_S83_L002	HCBf239	PA5	Chu	Infection	Fecal	San Diego	California	USA
MCT420_76_S84_L002	HCBf240	CL10T01C02	Chu	Healthy	Fecal	San Diego	California	USA
MCT420_77_S85_L002	HCBf241	CM12	Chu	Infection	Abscess	San Diego	California	USA
MCT420_80_S88_L002	HCBf244	CL13T01C12	Chu	Healthy	Fecal	San Diego	California	USA
MCT420_81_S89_L002	HCBf245	US390	Chu	Infection	Abscess	San Diego	California	USA
MCT420_86_S94_L002	HCBf250	CMR2896	Chu	Unknown	NA	San Diego	California	USA
MCT420_28_S36_L002	HCBf251	US398	Chu	Infection	Blood	San Diego	California	USA
MCT420_6_S14_L002	HCBf252	B35	Chu	Infection	Fecal	San Diego	California	USA
MCT420_7_S15_L002	HCBf253	12905-23V	Chu	Infection	Abscess	San Diego	California	USA

TABLE 2

Sample	Source
10317.X00178933_38	Qiita study 56124
10317.X00178992_47	Qiita study 56124
10317.X00178995_62	Qiita study 56124
10317.X00179017_10317.X00179017_bin.7	Qiita study 56124
10317.X00179025_65	Qiita study 56124
10317.X00179043_48	Qiita study 56124
10317.X00179047_10317.X00179047_bin.7	Qiita study 56124
10317.X00179093_41	Qiita study 56124
10317.X00179153_19	Qiita study 56124
10317.X00179156_10	Qiita study 56124
10317.X00179186_39	Qiita study 56124
10317.X00179225_54	Qiita study 56124
10317.X00179274_24	Qiita study 56124
10317.X00179278_57	Qiita study 56124
10317.X00179298_6	Qiita study 56124
10317.X00179300_11	Qiita study 56124
10317.X00179306_29	Qiita study 56124
10317.X00179317_4	Qiita study 56124
10317.X00179327_33	Qiita study 56124
10317.X00179381_10317.X00179381_bin.18	Qiita study 56124
10317.X00179407_24	Qiita study 56124
10317.X00179467_5	Qiita study 56124
10317.X00179487_25	Qiita study 56124
10317.X00179564_10317.X00179564_bin.11	Qiita study 56124
10317.X00179651_55	Qiita study 56124
10317.X00179673_25	Qiita study 56124
10317.X00179680_33	Qiita study 56124
10317.X00179725_20	Qiita study 56124
10317.X00179729_16	Qiita study 56124
10317.X00179736_10317.X00179736_bin.20	Qiita study 56124
10317.X00179805_8	Qiita study 56124

10317.X00179842_5	Qiita study 56124
10317.X00179954_75	Qiita study 56124
10317.X00180095_68	Qiita study 56124
10317.X00180154_10317.X00180154_bin.17	Qiita study 56124
10317.X00180160_15	Qiita study 56124
10317.X00180216_72	Qiita study 56124
10317.X00180243_84	Qiita study 56124
10317.X00180266_69	Qiita study 56124
10317.X00180334_10317.X00180334_bin.001	Qiita study 56124
10317.X00180334_10317.X00180334_bin.1	Qiita study 56124
10317.X00180463_38	Qiita study 56124
10317.X00180464_10317.X00180464_bin.2	Qiita study 56124
10317.X00180500_8	Qiita study 56124
10317.X00182267_21	Qiita study 56124
10317.X00185507_10317.X00185507_bin.17	Qiita study 56124
10317.X00185521_58	Qiita study 56124
10317.X00185597_21	Qiita study 56124
10317.X00185597_21_sub	Qiita study 56124
10317.X00185696_11	Qiita study 56124
10317.X00185732_3	Qiita study 56124
10317.X00185745_38	Qiita study 56124
10317.X00185752_7	Qiita study 56124
10317.X00185882_3	Qiita study 56124
10317.X00185925_50	Qiita study 56124
10317.X00214026_19	Qiita study 56124
10317.X00214096_10317.X00214096_bin.002	Qiita study 56124
10317.X00214097_10317.X00214097_bin.3	Qiita study 56124
10317.X00214119_32	Qiita study 56124
10317.X00214127_44_sub	Qiita study 56124
10317.X00214130_23	Qiita study 56124
10317.X00214152_5	Qiita study 56124
10317.X00214153_64	Qiita study 56124
10317.X00214161_10317.X00214161_bin.4	Qiita study 56124

10317.X00214162_22	Qiita study 56124
10317.X00214239_55	Qiita study 56124
10317.X00214299_94	Qiita study 56124
10317.X00214333_11	Qiita study 56124
10317.X00214694_67	Qiita study 56124
10317.X00214812_39	Qiita study 56124
10317.X00215088_24	Qiita study 56124
10317.X00215207_10317.X00215207_bin.26	Qiita study 56124
10317.X00215215_15	Qiita study 56124
10317.X00215241_67	Qiita study 56124
10317.X00215482_24	Qiita study 56124
BackhedF_2015__SID1_4M__bin.8	Pasoli et al 2019
BackhedF_2015__SID10_12M__bin.4	Pasoli et al 2019
BackhedF_2015__SID10_4M__bin.11	Pasoli et al 2019
BackhedF_2015__SID130_12M__bin.13	Pasoli et al 2019
BackhedF_2015__SID130_4M__bin.3	Pasoli et al 2019
BackhedF_2015__SID133_4M__bin.23	Pasoli et al 2019
BackhedF_2015__SID137_12M__bin.25	Pasoli et al 2019
BackhedF_2015__SID137_4M__bin.2	Pasoli et al 2019
BackhedF_2015__SID137_M__bin.72	Pasoli et al 2019
BackhedF_2015__SID150_12M__bin.19	Pasoli et al 2019
BackhedF_2015__SID150_M__bin.47	Pasoli et al 2019
BackhedF_2015__SID172_12M__bin.1	Pasoli et al 2019
BackhedF_2015__SID180_12M__bin.6	Pasoli et al 2019
BackhedF_2015__SID180_4M__bin.6	Pasoli et al 2019
BackhedF_2015__SID203_12M__bin.9	Pasoli et al 2019
BackhedF_2015__SID229_B__bin.3	Pasoli et al 2019
BackhedF_2015__SID238_12M__bin.7	Pasoli et al 2019
BackhedF_2015__SID238_B__bin.1	Pasoli et al 2019
BackhedF_2015__SID274_4M__bin.2	Pasoli et al 2019
BackhedF_2015__SID275_B__bin.6	Pasoli et al 2019
BackhedF_2015__SID275_M__bin.26	Pasoli et al 2019
BackhedF_2015__SID338_B__bin.1	Pasoli et al 2019
BackhedF_2015__SID503_12M__bin.45	Pasoli et al 2019
BackhedF_2015__SID503_4M__bin.20	Pasoli et al 2019
BackhedF_2015__SID521_4M__bin.12	Pasoli et al 2019
BackhedF_2015__SID526_4M__bin.6	Pasoli et al 2019
BackhedF_2015__SID53_4M__bin.14	Pasoli et al 2019

BackhedF_2015__SID546_12M__bin.30	Pasoli et al 2019
BackhedF_2015__SID546_4M__bin.5	Pasoli et al 2019
BackhedF_2015__SID565_4M__bin.10	Pasoli et al 2019
BackhedF_2015__SID566_12M__bin.34	Pasoli et al 2019
BackhedF_2015__SID567_12M__bin.4	Pasoli et al 2019
BackhedF_2015__SID570_4M__bin.7	Pasoli et al 2019
BackhedF_2015__SID582_12M__bin.6	Pasoli et al 2019
BackhedF_2015__SID587_12M__bin.17	Pasoli et al 2019
BackhedF_2015__SID591_4M__bin.17	Pasoli et al 2019
BackhedF_2015__SID615_12M__bin.2	Pasoli et al 2019
BackhedF_2015__SID615_4M__bin.5	Pasoli et al 2019
BackhedF_2015__SID626_4M__bin.17	Pasoli et al 2019
BackhedF_2015__SID635_B__bin.2	Pasoli et al 2019
BackhedF_2015__SID7_M__bin.18	Pasoli et al 2019
BackhedF_2015__SID78_4M__bin.8	Pasoli et al 2019
BritoIL_2016__M1.10.ST__bin.49	Pasoli et al 2019
ChengpingW_2017__AS123raw__bin.19	Pasoli et al 2019
ChengpingW_2017__AS18raw__bin.15	Pasoli et al 2019
ChengpingW_2017__AS5raw__bin.14	Pasoli et al 2019
CM_madagascar__A39_02_1FE__bin.19	Pasoli et al 2019
DavidLA_2015__LD-Run1-08__bin.7	Pasoli et al 2019
GCA_000009925.1	NCBI
GCA_000025985.1	NCBI
GCA_000162135.1	NCBI
GCA_000210835.1	NCBI
GCA_000218345.1	NCBI
GCA_000263115.1	NCBI
GCA_000269525.1	NCBI
GCA_000273095.1	NCBI
GCA_000273115.1	NCBI
GCA_000273135.1	NCBI
GCA_000273155.1	NCBI
GCA_000273765.1	NCBI
GCA_000297735.1	NCBI
GCA_000598145.1	NCBI
GCA_000598165.1	NCBI
GCA_000598185.2	NCBI
GCA_000598205.1	NCBI

GCA_000598225.1	NCBI
GCA_000598245.1	NCBI
GCA_000598265.1	NCBI
GCA_000598285.1	NCBI
GCA_000598305.1	NCBI
GCA_000598325.1	NCBI
GCA_000598345.1	NCBI
GCA_000598365.1	NCBI
GCA_000598385.1	NCBI
GCA_000598425.1	NCBI
GCA_000598445.1	NCBI
GCA_000598505.1	NCBI
GCA_000598525.1	NCBI
GCA_000598545.2	NCBI
GCA_000598565.1	NCBI
GCA_000598585.1	NCBI
GCA_000598645.1	NCBI
GCA_000598665.1	NCBI
GCA_000598685.2	NCBI
GCA_000598705.1	NCBI
GCA_000598725.1	NCBI
GCA_000598745.1	NCBI
GCA_000598765.2	NCBI
GCA_000598785.2	NCBI
GCA_000598805.1	NCBI
GCA_000598825.1	NCBI
GCA_000598845.1	NCBI
GCA_000598865.1	NCBI
GCA_000598885.1	NCBI
GCA_000598905.1	NCBI
GCA_000598925.1	NCBI
GCA_000598945.1	NCBI
GCA_000598965.1	NCBI
GCA_000598985.1	NCBI
GCA_000599005.1	NCBI
GCA_000599025.1	NCBI
GCA_000599045.1	NCBI
GCA_000599065.2	NCBI

GCA_000599085.1	NCBI
GCA_000599125.1	NCBI
GCA_000599145.2	NCBI
GCA_000599165.2	NCBI
GCA_000599185.2	NCBI
GCA_000599225.1	NCBI
GCA_000599245.1	NCBI
GCA_000599305.1	NCBI
GCA_000599325.1	NCBI
GCA_000599365.1	NCBI
GCA_000599385.1	NCBI
GCA_000601035.1	NCBI
GCA_000601055.1	NCBI
GCA_000601075.2	NCBI
GCA_000601095.1	NCBI
GCA_000601115.1	NCBI
GCA_000724795.2	NCBI
GCA_000965785.1	NCBI
GCA_001054865.1	NCBI
GCA_001054895.1	NCBI
GCA_001056335.1	NCBI
GCA_001058755.1	NCBI
GCA_001058775.1	NCBI
GCA_001286525.1	NCBI
GCA_001580095.1	NCBI
GCA_001580105.1	NCBI
GCA_001682215.1	NCBI
GCA_001692695.1	NCBI
GCA_001699855.1	NCBI
GCA_001699865.1	NCBI
GCA_001699875.1	NCBI
GCA_001699885.1	NCBI
GCA_001816225.2	NCBI
GCA_001997325.1	NCBI
GCA_002810985.1	NCBI
GCA_002810995.1	NCBI
GCA_002811025.1	NCBI
GCA_002811035.1	NCBI

GCA_002811065.1	NCBI
GCA_002811085.1	NCBI
GCA_003363115.1	NCBI
GCA_003436285.1	NCBI
GCA_003436935.1	NCBI
GCA_003437815.1	NCBI
GCA_003438775.1	NCBI
GCA_003438895.1	NCBI
GCA_003439285.1	NCBI
GCA_003439505.1	NCBI
GCA_003439635.1	NCBI
GCA_003439675.1	NCBI
GCA_003458215.1	NCBI
GCA_003458955.1	NCBI
GCA_003463555.1	NCBI
GCA_003464385.1	NCBI
GCA_003468145.1	NCBI
GCA_003469305.1	NCBI
GCA_003469705.1	NCBI
GCA_003471925.1	NCBI
GCA_003472025.1	NCBI
GCA_003472195.1	NCBI
GCA_003473955.1	NCBI
GCA_003475385.1	NCBI
GCA_003475745.1	NCBI
GCA_004167855.1	NCBI
GCA_004798445.1	NCBI
GCA_004798525.1	NCBI
GCA_005706655.1	NCBI
GCA_005844845.1	NCBI
GCA_007896575.1	NCBI
GCA_007896595.1	NCBI
GCA_007896605.1	NCBI
GCA_007896675.1	NCBI
GCA_007896685.1	NCBI
GCA_007896745.1	NCBI
GCA_007896795.1	NCBI
GCA_008086695.1	NCBI

GCA_008369705.1	NCBI
GCA_008569055.1	NCBI
GCA_008569225.1	NCBI
GCA_008569265.1	NCBI
GCA_008569305.1	NCBI
GCA_008569335.1	NCBI
GCA_008569345.1	NCBI
GCA_008569365.1	NCBI
GCA_008569395.1	NCBI
GCA_008569415.1	NCBI
GCA_008569445.1	NCBI
GCA_008829725.1	NCBI
GCA_009024655.1	NCBI
GCA_009025495.1	NCBI
GCA_009025695.1	NCBI
GCA_009025705.1	NCBI
GCA_012844105.1	NCBI
GCA_013327835.1	NCBI
GCA_013694025.1	NCBI
GCA_013867505.1	NCBI
GCA_014042405.1	NCBI
GCA_014042415.1	NCBI
GCA_014042445.1	NCBI
GCA_014042455.1	NCBI
GCA_014042465.1	NCBI
GCA_014042505.1	NCBI
GCA_014042525.1	NCBI
GCA_014893755.1	NCBI
GCA_015548495.1	NCBI
GCA_015548595.1	NCBI
GCA_015548825.1	NCBI
GCA_015551365.1	NCBI
GCA_015553295.1	NCBI
GCA_015553495.1	NCBI
GCA_015553755.1	NCBI
GCA_015556305.1	NCBI
GCA_015558985.1	NCBI
GCA_015560515.1	NCBI

GCA_015669295.1	NCBI
GCA_015669435.1	NCBI
GCA_015831745.1	NCBI
GCA_015831765.1	NCBI
GCA_016889925.1	NCBI
GCA_018292185.1	NCBI
GCA_018369215.1	NCBI
GCA_018786025.1	NCBI
GCA_019013355.1	NCBI
GCA_019041735.1	NCBI
GCA_019041775.1	NCBI
GCA_019041875.1	NCBI
GCA_019130335.1	NCBI
GCA_019130425.1	NCBI
GCA_019733435.1	NCBI
GCA_019915805.1	NCBI
GCA_020026255.1	NCBI
GCA_020091565.1	NCBI
GCA_020097275.1	NCBI
GCA_020256775.1	NCBI
GCA_020256835.1	NCBI
GCA_020256925.1	NCBI
GCA_020257085.1	NCBI
GCA_020257125.1	NCBI
GCA_020297425.1	NCBI
GCA_020297435.1	NCBI
GCA_020297545.1	NCBI
GCA_020297585.1	NCBI
GCA_020538785.1	NCBI
GCA_020539125.1	NCBI
GCA_020558655.1	NCBI
GCA_020558865.1	NCBI
GCA_020859785.1	NCBI
GCA_021202455.1	NCBI
GCA_021633405.1	NCBI
GCA_022714225.1	NCBI
GCA_022714305.1	NCBI
GCA_022716295.1	NCBI

GCA_022716895.1	NCBI
GCA_022717255.1	NCBI
GCA_022718435.1	NCBI
GCA_022719595.1	NCBI
GCA_022719775.1	NCBI
GCA_022720585.1	NCBI
GCA_022721415.1	NCBI
GCA_022722555.1	NCBI
GCA_022724695.1	NCBI
GCA_022725025.1	NCBI
GCA_022725225.1	NCBI
GCA_022727875.1	NCBI
GCA_022731325.1	NCBI
GCA_022733095.1	NCBI
GCA_022733395.1	NCBI
GCA_022733815.1	NCBI
GCA_022734035.1	NCBI
GCA_022734285.1	NCBI
GCA_022734965.1	NCBI
GCA_022735135.1	NCBI
GCA_022737435.1	NCBI
GCA_022737795.1	NCBI
GCA_022738395.1	NCBI
GCA_022741265.1	NCBI
GCA_022741435.1	NCBI
GCA_022741515.1	NCBI
GCA_022742205.1	NCBI
GCA_022742265.1	NCBI
GCA_022744835.1	NCBI
GCA_022744925.1	NCBI
GCA_022745295.1	NCBI
GCA_022745625.1	NCBI
GCA_022746345.1	NCBI
GCA_022746405.1	NCBI
GCA_022746755.1	NCBI
GCA_022747335.1	NCBI
GCA_022780605.1	NCBI
GCA_022782465.1	NCBI

GCA_022832315.1	NCBI
GCA_022832325.1	NCBI
GCA_022832355.1	NCBI
GCA_023274245.1	NCBI
GCA_023274315.1	NCBI
GCA_023274495.1	NCBI
GCA_023274715.1	NCBI
GCA_023274755.1	NCBI
GCA_023274775.1	NCBI
GCA_023274895.1	NCBI
GCA_023275035.1	NCBI
GCA_023276265.1	NCBI
GCA_023276305.1	NCBI
GCA_023276335.1	NCBI
GCA_023702735.1	NCBI
GCA_023894135.1	NCBI
GCA_023958435.1	NCBI
GCA_024007805.1	NCBI
GCA_900445515.1	NCBI
GCA_900624785.1	NCBI
GCA_901212445.1	NCBI
GCA_902374115.1	NCBI
GCA_905188165.1	NCBI
GeversD_2014__SKBSTL063__bin.2	Pasoli et al 2019
GeversD_2014__SKSTL0001__bin.2	Pasoli et al 2019
HeQ_2017__RSZAXPI003062-102__bin.4	Pasoli et al 2019
HeQ_2017__RSZAXPI003078-111__bin.2	Pasoli et al 2019
HeQ_2017__RSZAXPI003098-132__bin.12	Pasoli et al 2019
HeQ_2017__SZAXPI029475-44__bin.35	Pasoli et al 2019
HeQ_2017__SZAXPI029574-89__bin.37	Pasoli et al 2019
HMP_2012__SRS018817__bin.4	Pasoli et al 2019
HMP_2012__SRS023346__bin.6	Pasoli et al 2019
HMP_2012__SRS075398__bin.15	Pasoli et al 2019
KarlssonFH_2013__S248__bin.36	Pasoli et al 2019
KosticAD_2015__PRJNA231909.3101436__bin.15	Pasoli et al 2019
KosticAD_2015__PRJNA231909.3101439__bin.2	Pasoli et al 2019
KosticAD_2015__PRJNA231909.3107649__bin.14	Pasoli et al 2019
KosticAD_2015__PRJNA231909.3108874__bin.9	Pasoli et al 2019

KosticAD_2015__PRJNA231909.3112348__bin.8	Pasoli et al 2019
KosticAD_2015__PRJNA231909.3113035__bin.1	Pasoli et al 2019
KosticAD_2015__PRJNA231909.3113831__bin.8	Pasoli et al 2019
KosticAD_2015__PRJNA231909.3113834__bin.5	Pasoli et al 2019
LeChatelierE_2013__MH0068__bin.12	Pasoli et al 2019
LeChatelierE_2013__MH0193__bin.44	Pasoli et al 2019
LeChatelierE_2013__MH0410__bin.30	Pasoli et al 2019
LiJ_2014__MH0221__bin.8	Pasoli et al 2019
LiJ_2014__V1.CD3-0-PN__bin.42	Pasoli et al 2019
LiJ_2014__V1.FI21__bin.42	Pasoli et al 2019
LiJ_2017__H1M213648__bin.18	Pasoli et al 2019
LiJ_2017__H3M515950__bin.24	Pasoli et al 2019
LiSS_2016__FAT_015-22-42-0__bin.55	Pasoli et al 2019
LiuW_2016__SRR3993030__bin.36	Pasoli et al 2019
LiuW_2016__SRR3993044__bin.123	Pasoli et al 2019
LoombaR_2017__SID5362_xgk__bin.16	Pasoli et al 2019
NielsenHB_2014__O2_UC34_2__bin.41	Pasoli et al 2019
NielsenHB_2014__V1_CD21_4__bin.5	Pasoli et al 2019
NielsenHB_2014__V1_CD6_4__bin.58	Pasoli et al 2019
NielsenHB_2014__V1_UC15_0__bin.1	Pasoli et al 2019
NielsenHB_2014__V1_UC15_3__bin.64	Pasoli et al 2019
NielsenHB_2014__V1_UC34_0__bin.49	Pasoli et al 2019
Obregon-TitoAJ_2015__NO05__bin.48	Pasoli et al 2019
QinJ_2012__CON-005__bin.52	Pasoli et al 2019
QinJ_2012__DLF009__bin.9	Pasoli et al 2019
QinJ_2012__NLM028__bin.25	Pasoli et al 2019
QinN_2014__LD-26__bin.50	Pasoli et al 2019
QinN_2014__LD-33__bin.18	Pasoli et al 2019
QinN_2014__LD-34__bin.5	Pasoli et al 2019
QinN_2014__LD-35__bin.21	Pasoli et al 2019
QinN_2014__LD-50__bin.33	Pasoli et al 2019
QinN_2014__LD-93__bin.8	Pasoli et al 2019
RaymondF_2016__P22E7__bin.4	Pasoli et al 2019
SchirmerM_2016__G88708__bin.4	Pasoli et al 2019
SRR12280270	
SRR12280314	
SRR12280315	
SRR12280316	

SRR12280336	
SRR12280416	
SRR12280417	
SRR12280492	
SRR12280493	
SRR12280496	
SRR12280498	
SRR12280499	
SRR12280500	
SRR12280501	
SRR12280503	
SRR12280504	
SRR12280511	
SRR12280522	
SRR12280549	
SRR12280561	
SRR12280572	
SRR12280583	
SRR12280589	
SRR12280591	
SRR12280608	
SRR12280619	
SRR12280632	
SRR12280635	
SRR12280642	NCBI
SRR12280643	NCBI
SRR12280645	NCBI
SRR12280646	NCBI
SRR12280647	NCBI
SRR12280648	NCBI
SRR12280649	NCBI
SRR12280650	NCBI
SRR12280651	NCBI
SRR12280658	NCBI
SRR13269111	NCBI
SRR13269122	NCBI
SRR13269147	NCBI
SRR13269148	NCBI

SRR13269149	NCBI
SRR13269150	NCBI
SRR13269152	NCBI
SRR13269154	NCBI
SRR13269155	NCBI
SRR13269156	NCBI
SRR13269157	NCBI
SRR13269158	NCBI
SRR13269159	NCBI
SRR13269161	NCBI
SRR13269162	NCBI
SRR13269163	NCBI
SRR13269164	NCBI
SRR13269165	NCBI
SRR13269166	NCBI
SRR13269167	NCBI
SRR13269178	NCBI
SRR13269194	NCBI
SRR13269204	NCBI
SRR13269207	NCBI
SRR13269209	NCBI
SRR13269219	NCBI
SRR13269220	NCBI
SRR13269221	NCBI
SRR13269222	NCBI
SRR13269223	NCBI
SRR13269224	NCBI
SRR13269245	NCBI
SRR15283685	NCBI
SRR15283687	NCBI
SRR15283688	NCBI
SRR15283691	NCBI
SRR15283695	NCBI
SRR15283697	NCBI
SRR15283699	NCBI
SRR15283700	NCBI
SRR15283701	NCBI
SRR15283704	NCBI

SRR15283705	NCBI
SRR15283706	NCBI
SRR15283707	NCBI
SRR15283708	NCBI
SRR15283713	NCBI
SRR15283714	NCBI
SRR15283717	NCBI
SRR15283720	NCBI
SRR15283722	NCBI
SRR15283724	NCBI
SRR15283728	NCBI
SRR15283731	NCBI
SRR15283737	NCBI
SRR15283738	NCBI
SRR15283742	NCBI
SRR15283745	NCBI
SRR15283746	NCBI
SRR15283747	NCBI
SRR15283748	NCBI
SRR15283749	NCBI
SRR15283750	NCBI
SRR15283752	NCBI
SRR15283753	NCBI
SRR15283757	NCBI
SRR15283758	NCBI
SRR15283762	NCBI
SRR15283763	NCBI
SRR15283766	NCBI
SRR15283767	NCBI
SRR15283771	NCBI
SRR15283795	NCBI
SRR15283815	NCBI
SRR15283820	NCBI
SRR15283822	NCBI
SRR15283825	NCBI
SRR15283826	NCBI
SRR15283828	NCBI
SRR15283834	NCBI

SRR15283837	NCBI
SRR15283838	NCBI
SRR15283839	NCBI
SRR15283841	NCBI
SRR15283842	NCBI
SRR15283844	NCBI
SRR15283848	NCBI
SRR15283850	NCBI
SRR15283852	NCBI
SRR15283854	NCBI
SRR15283861	NCBI
SRR15283864	NCBI
SRR15283865	NCBI
SRR15283867	NCBI
SRR15283868	NCBI
SRR15283869	NCBI
SRR15283871	NCBI
SRR15283873	NCBI
SRR15283876	NCBI
SRR15283887	NCBI
SRR15283888	NCBI
SRR15283890	NCBI
SRR15283891	NCBI
SRR15283892	NCBI
SRR15283894	NCBI
SRR15283896	NCBI
SRR9205639	NCBI
SRR9205744	NCBI
SRR9205813	NCBI
SRR9206004	NCBI
SRR9206010	NCBI
SRR9206014	NCBI
SRR9206104	NCBI
SRR9206107	NCBI
SRR9206177	NCBI
SRR9206233	NCBI
SRR9206239	NCBI
SRR9206387	NCBI

SRR9206391	NCBI
SRR9206392	NCBI
SRR9206393	NCBI
SRR9206396	NCBI
VatanenT_2016__G69155__bin.3	Pasoli et al 2019
VatanenT_2016__G78526__bin.12	Pasoli et al 2020
VatanenT_2016__G78527__bin.3	Pasoli et al 2021
VatanenT_2016__G78583__bin.3	Pasoli et al 2022
VatanenT_2016__G78592__bin.3	Pasoli et al 2023
VatanenT_2016__G78604__bin.1	Pasoli et al 2024
VatanenT_2016__G78605__bin.3	Pasoli et al 2025
VatanenT_2016__G78619__bin.7	Pasoli et al 2026
VatanenT_2016__G78646__bin.3	Pasoli et al 2027
VatanenT_2016__G78669__bin.1	Pasoli et al 2028
VatanenT_2016__G78676__bin.4	Pasoli et al 2029
VatanenT_2016__G78715__bin.6	Pasoli et al 2030
VatanenT_2016__G78716__bin.3	Pasoli et al 2031
VatanenT_2016__G78739__bin.3	Pasoli et al 2032
VatanenT_2016__G78794__bin.3	Pasoli et al 2033
VatanenT_2016__G78795__bin.5	Pasoli et al 2034
VatanenT_2016__G78826__bin.7	Pasoli et al 2035
VatanenT_2016__G78890__bin.1	Pasoli et al 2036
VatanenT_2016__G78910__bin.4	Pasoli et al 2037
VatanenT_2016__G80158__bin.16	Pasoli et al 2038
VatanenT_2016__G80290__bin.3	Pasoli et al 2039
VatanenT_2016__G80297__bin.3	Pasoli et al 2040
VatanenT_2016__G80299__bin.1	Pasoli et al 2041
VatanenT_2016__G80313__bin.13	Pasoli et al 2042
VatanenT_2016__G80388__bin.7	Pasoli et al 2043
VatanenT_2016__G80399__bin.4	Pasoli et al 2044
VatanenT_2016__G80406__bin.2	Pasoli et al 2045
VatanenT_2016__G80458__bin.3	Pasoli et al 2046
VatanenT_2016__G80469__bin.5	Pasoli et al 2047
VatanenT_2016__G80499__bin.3	Pasoli et al 2048
VatanenT_2016__G80507__bin.5	Pasoli et al 2049
VatanenT_2016__G80518__bin.7	Pasoli et al 2050
VatanenT_2016__G80532__bin.12	Pasoli et al 2051
VatanenT_2016__G80556__bin.10	Pasoli et al 2052

VatanenT_2016__G80584__bin.1	Pasoli et al 2053
VatanenT_2016__G80590__bin.4	Pasoli et al 2054
VatanenT_2016__G80592__bin.1	Pasoli et al 2055
VatanenT_2016__G80601__bin.2	Pasoli et al 2056
VatanenT_2016__G80602__bin.2	Pasoli et al 2057
VatanenT_2016__G80611__bin.2	Pasoli et al 2058
VincentC_2016__MM022.2__bin.34	Pasoli et al 2059
VincentC_2016__MM032.3__bin.19	Pasoli et al 2060
VincentC_2016__MM060.2__bin.1	Pasoli et al 2061
VogtmannE_2016__MMRS62192467ST-27-0-0__bin.44	Pasoli et al 2062
VogtmannE_2016__MMRS68910755ST-27-0-0__bin.44	Pasoli et al 2063
VogtmannE_2016__MMRS72980899ST-27-0-0__bin.27	Pasoli et al 2064
VogtmannE_2016__MMRS77321055ST-27-0-0__bin.39	Pasoli et al 2065
WenC_2017__N016__bin.21	Pasoli et al 2066
WenC_2017__N281__bin.22	Pasoli et al 2067
WenC_2017__RA132__bin.29	Pasoli et al 2068
XieH_2016__YSZC12003_35524__bin.22	Pasoli et al 2069
XieH_2016__YSZC12003_36052__bin.34	Pasoli et al 2070
XieH_2016__YSZC12003_37205__bin.26	Pasoli et al 2071
XieH_2016__YSZC12003_37207R1__bin.36	Pasoli et al 2072
XieH_2016__YSZC12003_37269__bin.22	Pasoli et al 2073
XieH_2016__YSZC12003_37879__bin.30	Pasoli et al 2074
XieH_2016__YSZC12003_37880__bin.18	Pasoli et al 2075
YuJ_2015__SZAXPI003422-11__bin.33	Pasoli et al 2076
YuJ_2015__SZAXPI003435-11__bin.64	Pasoli et al 2077
YuJ_2015__SZAXPI015216-169__bin.42	Pasoli et al 2078
YuJ_2015__SZAXPI015250-39__bin.22	Pasoli et al 2079
ZeeviD_2015__PNP_Main_174__bin.22	Pasoli et al 2080
ZeeviD_2015__PNP_Main_319__bin.2	Pasoli et al 2081
ZeeviD_2015__PNP_Validation_92__bin.15	Pasoli et al 2082
ZellerG_2014__CCIS58234805ST-4-0__bin.17	Pasoli et al 2083
ZellerG_2014__CCIS76845094ST-20-0__bin.49	Pasoli et al 2084

TABLE 3

Sample	Phylogroup
10317.X00178933_38	12
10317.X00178992_47	4
10317.X00178995_62	9
10317.X00179017_10317.X00'	4
10317.X00179025_65	17
10317.X00179043_48	16
10317.X00179047_10317.X00'	4
10317.X00179093_41	6
10317.X00179153_19	16
10317.X00179156_10	16
10317.X00179186_39	6
10317.X00179225_54	16
10317.X00179274_24	3
10317.X00179278_57	16
10317.X00179298_6	11
10317.X00179300_11	5
10317.X00179306_29	9
10317.X00179317_4	16
10317.X00179327_33	16
10317.X00179381_10317.X00'	3
10317.X00179407_24	3
10317.X00179467_5	10
10317.X00179487_25	3
10317.X00179564_10317.X00'	7
10317.X00179651_55	16
10317.X00179673_25	11
10317.X00179680_33	3
10317.X00179725_20	6
10317.X00179729_16	13
10317.X00179736_10317.X00'	16
10317.X00179805_8	11
10317.X00179842_5	2
10317.X00179954_75	11
10317.X00180095_68	7
10317.X00180154_10317.X00'	16
10317.X00180160_15	7
10317.X00180216_72	16
10317.X00180243_84	5
10317.X00180266_69	1
10317.X00180334_10317.X00'	1
10317.X00180334_10317.X00'	1
10317.X00180463_38	16
10317.X00180464_10317.X00'	9

10317.X00180500_8	16
10317.X00182267_21	4
10317.X00185507_10317.X00	3
10317.X00185521_58	4
10317.X00185597_21_sub	16
10317.X00185696_11	5
10317.X00185732_3	16
10317.X00185745_38	7
10317.X00185752_7	16
10317.X00185882_3	3
10317.X00185925_50	7
10317.X00214026_19	3
10317.X00214096_10317.X00	2
10317.X00214097_10317.X00	11
10317.X00214119_32	3
10317.X00214127_44_sub	3
10317.X00214130_23	3
10317.X00214152_5	8
10317.X00214153_64	16
10317.X00214161_10317.X00	10
10317.X00214162_22	4
10317.X00214239_55	13
10317.X00214299_94	3
10317.X00214333_11	10
10317.X00214694_67	3
10317.X00214812_39	12
10317.X00215088_24	13
10317.X00215207_10317.X00	3
10317.X00215215_15	8
10317.X00215241_67	16
10317.X00215482_24	9
363191840_S199_L001.1	4
363192044_S216_L001.3	4
363192053_S223_L001.4	8
363192522_S5_L001	12
363192524_S22_L001.1	16
363192525_S32_L001.2	16
363192527_S10_L001	16
363192528_S11_L001	9
363192533_S31_L001	8
363192539_S19_L001	17
363192541_S20_L001	14
363192542_S33_L001.3	2
363192544_S35_L001	4
363192546_S37_L001.1	16
363192548_S39_L001	8
363192552_S43_L001	3
363192554_S45_L001.2	16

363192555_S46_L001	4
363192557_S48_L001	8
363192559_S50_L001	16
363192560_S51_L001	2
363192563_S54_L001	4
363192564_S55_L001	16
363192566_S57_L001	8
363192568_S59_L001	16
363192573_S64_L001	4
363192575_S66_L001.1	10
363192577_S68_L001	16
363192579_S70_L001	10
363192581_S72_L001	4
363192583_S74_L001	10
363192585_S76_L001	16
363192589_S80_L001	9
363192591_S82_L001	16
363192595_S86_L001	16
363192601_S92_L001	16
363192602_S93_L001	4
363192603_S94_L001	11
363192605_S96_L001	4
363192793_S98_L001	16
363192795_S100_L001	16
363192797_S102_L001	7
363192803_S112_L001.2	11
363192805_S110_L001	11
363192998_S106_L001	17
363192999_S107_L001	13
363193001_S108_L001	4
363193002_S105_L001	6
363193003_S113_L001.4	6
363193004_S114_L001	13
363193013_S123_L001	3
363193015_S125_L001.1	4
363193016_S126_L001.1	16
363193022_S130_L001.1	4
363193027_S137_L001	3
363193028_S138_L001	17
363193029_S139_L001	11
363193031_S141_L001.1	4
363193032_S142_L001	8
363193035_S145_L001	16
363193036_S146_L001	4
363193037_S147_L001	14
363193039_S149_L001	16
363193040_S150_L001	12
363193043_S153_L001	1
363193044_S154_L001	17
363193046_S156_L001	17
363193047_S157_L001	10
363193048_S163_L001	11

363193049_S158_L001	15
363193054_S164_L001	16
363193059_S169_L001	7
363193060_S170_L001	16
363193061_S171_L001	16
363193063_S173_L001.2	10
363193064_S174_L001	15
363193068_S178_L001	16
363193069_S179_L001	11
363193072_S181_L001	7
363193076_S185_L001	15
363193078_S188_L001	7
363193079_S189_L001	14
363193754_S16_L001.2	11
363193756_S6_L001	11
363193757_S8_L001	4
363193763_S14_L001	7
363197048_S313_L001.1	16
363197054_S337_L001.2	4
363197065_S315_L001	13
363197078_S309_L001.3	16
363197087_S326_L001.5	6
363197090_S325_L001.1	6
BackhedF_2015__SID10_12M	4
BackhedF_2015__SID130_12l	16
BackhedF_2015__SID130_4M	16
BackhedF_2015__SID133_4M	8
BackhedF_2015__SID137_12l	15
BackhedF_2015__SID137_4M	15
BackhedF_2015__SID137_M_	15
BackhedF_2015__SID150_12l	8
BackhedF_2015__SID150_M_	8
BackhedF_2015__SID172_12l	4
BackhedF_2015__SID180_4M	16
BackhedF_2015__SID1_4M_	2
BackhedF_2015__SID203_12l	4
BackhedF_2015__SID229_B_	3
BackhedF_2015__SID238_12l	3
BackhedF_2015__SID238_B_	3
BackhedF_2015__SID274_4M	17
BackhedF_2015__SID275_B_	16
BackhedF_2015__SID275_M_	16
BackhedF_2015__SID338_B_	16
BackhedF_2015__SID503_12l	8
BackhedF_2015__SID503_4M	8
BackhedF_2015__SID521_4M	14
BackhedF_2015__SID526_4M	15
BackhedF_2015__SID53_4M_	14
BackhedF_2015__SID546_12l	17
BackhedF_2015__SID546_4M	17
BackhedF_2015__SID565_4M	13
BackhedF_2015__SID566_12l	4

BackhedF_2015__SID567_12I	4
BackhedF_2015__SID570_4M	8
BackhedF_2015__SID582_12I	6
BackhedF_2015__SID587_12I	16
BackhedF_2015__SID591_4M	6
BackhedF_2015__SID615_12I	16
BackhedF_2015__SID615_4M	16
BackhedF_2015__SID626_4M	16
BackhedF_2015__SID635_B_	16
BackhedF_2015__SID78_4M_	9
BackhedF_2015__SID7_M__t	12
BritoIL_2016__M1.10.ST__bir	8
CM_madagascar__A39_02_1	4
ChengpingW_2017__AS123ra	8
ChengpingW_2017__AS18rav	10
ChengpingW_2017__AS5raw_	4
DavidLA_2015__LD-Run1-08_	16
GCA_000210835.1	17
GCA_000218345.1	10
GCA_000273155.1	14
GCA_000273765.1	3
GCA_000297735.1	1
GCA_000598145.1	3
GCA_000598185.2	6
GCA_000598205.1	2
GCA_000598225.1	7
GCA_000598245.1	11
GCA_000598285.1	3
GCA_000598305.1	5
GCA_000598325.1	16
GCA_000598345.1	16
GCA_000598365.1	2
GCA_000598385.1	7
GCA_000598425.1	3
GCA_000598445.1	3
GCA_000598505.1	3
GCA_000598525.1	16
GCA_000598545.2	16
GCA_000598565.1	16
GCA_000598585.1	16
GCA_000598645.1	16
GCA_000598665.1	16
GCA_000598705.1	13
GCA_000598725.1	3
GCA_000598785.2	8
GCA_000598805.1	16
GCA_000598825.1	3
GCA_000598845.1	1
GCA_000598865.1	13
GCA_000598905.1	3
GCA_000598985.1	8
GCA_000599065.2	16

GCA_000599085.1	11
GCA_000599125.1	11
GCA_000599165.2	16
GCA_000599185.2	3
GCA_000599325.1	10
GCA_000599365.1	4
GCA_000601055.1	16
GCA_000601075.2	11
GCA_000601095.1	16
GCA_000601115.1	6
GCA_000724795.2	5
GCA_000965785.1	8
GCA_001056335.1	7
GCA_001058775.1	16
GCA_001286525.1	9
GCA_001682215.1	4
GCA_001692695.1	12
GCA_001699855.1	3
GCA_001699865.1	10
GCA_001699875.1	16
GCA_001816225.2	15
GCA_002811025.1	4
GCA_002811035.1	11
GCA_002811085.1	10
GCA_003436285.1	16
GCA_003436935.1	16
GCA_003437815.1	7
GCA_003438895.1	11
GCA_003439505.1	2
GCA_003439675.1	10
GCA_003458215.1	10
GCA_003458955.1	4
GCA_003463555.1	2
GCA_003464385.1	4
GCA_003468145.1	4
GCA_003469305.1	3
GCA_003469705.1	8
GCA_003472025.1	7
GCA_003472195.1	4
GCA_003473955.1	15
GCA_003475385.1	4
GCA_003475745.1	10
GCA_004167855.1	16
GCA_004798445.1	17
GCA_004798525.1	17
GCA_007896595.1	7
GCA_008086695.1	13
GCA_008369705.1	10
GCA_008569345.1	9
GCA_008829725.1	3
GCA_009024655.1	16
GCA_009025705.1	16

GCA_012844105.1	16
GCA_013327835.1	12
GCA_013694025.1	7
GCA_013867505.1	16
GCA_014042405.1	16
GCA_014042415.1	7
GCA_014042445.1	7
GCA_014042455.1	1
GCA_014042465.1	9
GCA_014042505.1	16
GCA_014042525.1	3
GCA_014893755.1	11
GCA_015548495.1	2
GCA_015548595.1	7
GCA_015548825.1	4
GCA_015551365.1	10
GCA_015553295.1	16
GCA_015553495.1	5
GCA_015553755.1	16
GCA_015556305.1	16
GCA_015558985.1	16
GCA_015560515.1	14
GCA_015669295.1	1
GCA_015669435.1	10
GCA_015831765.1	16
GCA_016889925.1	7
GCA_018292185.1	11
GCA_018369215.1	17
GCA_018786025.1	3
GCA_019013355.1	1
GCA_019041875.1	9
GCA_019130425.1	10
GCA_019733435.1	3
GCA_019915805.1	17
GCA_020026255.1	16
GCA_020257125.1	15
GCA_020297425.1	16
GCA_020297435.1	9
GCA_020297545.1	6
GCA_020297585.1	4
GCA_020539125.1	7
GCA_020558655.1	5
GCA_020558865.1	15
GCA_020859785.1	4
GCA_021202455.1	8
GCA_021633405.1	16
GCA_022714225.1	10
GCA_022714305.1	5
GCA_022716295.1	17
GCA_022716895.1	16
GCA_022717255.1	16
GCA_022718435.1	4

GCA_022719595.1	16
GCA_022719775.1	16
GCA_022720585.1	16
GCA_022721415.1	16
GCA_022722555.1	4
GCA_022724695.1	17
GCA_022725025.1	10
GCA_022725225.1	11
GCA_022727875.1	8
GCA_022731325.1	11
GCA_022733095.1	16
GCA_022733395.1	17
GCA_022733815.1	16
GCA_022734035.1	13
GCA_022734285.1	10
GCA_022734965.1	16
GCA_022735135.1	2
GCA_022737435.1	3
GCA_022737795.1	16
GCA_022738395.1	16
GCA_022741265.1	10
GCA_022741435.1	4
GCA_022741515.1	16
GCA_022742205.1	8
GCA_022742265.1	16
GCA_022744835.1	17
GCA_022744925.1	17
GCA_022745295.1	16
GCA_022745625.1	11
GCA_022746345.1	11
GCA_022746405.1	17
GCA_022746755.1	9
GCA_022747335.1	16
GCA_022780605.1	14
GCA_022782465.1	1
GCA_022832315.1	11
GCA_022832325.1	5
GCA_022832355.1	5
GCA_023702735.1	10
GCA_024007805.1	4
GCA_900624785.1	3
GCA_901212445.1	13
GCA_902374115.1	6
GCA_905188165.1	16
GeversD_2014__SKBSTL063	8
GeversD_2014__SKSTL0001_	5
HMP_2012__SRS018817__bi	11
HMP_2012__SRS023346__bi	3
HMP_2012__SRS075398__bi	16
HeQ_2017__RSZAXPI003062	13
HeQ_2017__RSZAXPI003078	4
HeQ_2017__RSZAXPI003098	4

HeQ_2017__SZAXPI029475-4	12
HeQ_2017__SZAXPI029574-8	11
KarlssonFH_2013__S248__bi	16
KosticAD_2015__PRJNA2319	17
KosticAD_2015__PRJNA2319	17
KosticAD_2015__PRJNA2319	17
KosticAD_2015__PRJNA2319	13
KosticAD_2015__PRJNA2319	6
KosticAD_2015__PRJNA2319	6
KosticAD_2015__PRJNA2319	4
KosticAD_2015__PRJNA2319	4
LeChatelierE_2013__MH0068	1
LeChatelierE_2013__MH0193	11
LeChatelierE_2013__MH0410	8
LiJ_2014__MH0221__bin.8	16
LiJ_2014__V1.CD3-0-PN__bi	8
LiJ_2014__V1.FI21__bin.42	3
LiJ_2017__H1M213648__bin.	13
LiJ_2017__H3M515950__bin.	10
LiSS_2016__FAT_015-22-42-	10
LiuW_2016__SRR3993030__l	16
LiuW_2016__SRR3993044__l	11
LoombaR_2017__SID5362_xç	16
MCT420_10_S18_L002	3
MCT420_18_S26_L002	16
MCT420_28_S36_L002	11
MCT420_29_S37_L002	16
MCT420_2_S10_L002	16
MCT420_32_S40_L002	13
MCT420_35_S43_L002	16
MCT420_36_S44_L002	9
MCT420_41_S49_L002	4
MCT420_47_S55_L002	14
MCT420_49_S57_L002	16
MCT420_50_S58_L002	1
MCT420_52_S60_L002	16
MCT420_54_S62_L002	6
MCT420_55_S63_L002	16
MCT420_58_S66_L002	5
MCT420_59_S67_L002	16
MCT420_60_S68_L002	4
MCT420_63_S71_L002	16
MCT420_6_S14_L002	4
MCT420_75_S83_L002	1
MCT420_76_S84_L002	16
MCT420_77_S85_L002	3
MCT420_7_S15_L002	16
MCT420_80_S88_L002	16
MCT420_81_S89_L002	4
MCT420_86_S94_L002	4
MCT420_8_S16_L002	4
NielsenHB_2014__O2_UC34_	5

NielsenHB_2014__V1_CD21_	16
NielsenHB_2014__V1_CD6_4	16
NielsenHB_2014__V1_UC15_	2
NielsenHB_2014__V1_UC15_	2
NielsenHB_2014__V1_UC34_	14
Obregon-TitoAJ_2015__NO05	6
QinJ_2012__CON-005__bin.5	12
QinJ_2012__DLF009__bin.9	16
QinJ_2012__NLM028__bin.25	15
QinN_2014__LD-26__bin.50	15
QinN_2014__LD-33__bin.18	10
QinN_2014__LD-34__bin.5	16
QinN_2014__LD-35__bin.21	16
QinN_2014__LD-50__bin.33	16
QinN_2014__LD-93__bin.8	8
RaymondF_2016__P22E7__b	3
SRR12280270	16
SRR12280314	5
SRR12280315	3
SRR12280336	12
SRR12280416	8
SRR12280417	16
SRR12280492	2
SRR12280493	10
SRR12280496	11
SRR12280498	3
SRR12280499	8
SRR12280500	17
SRR12280501	3
SRR12280503	16
SRR12280504	16
SRR12280511	8
SRR12280522	4
SRR12280549	17
SRR12280561	2
SRR12280572	16
SRR12280583	16
SRR12280589	9
SRR12280591	16
SRR12280608	16
SRR12280632	8
SRR12280635	16
SRR12280643	12
SRR12280645	2
SRR12280646	10
SRR12280649	1
SRR12280650	3
SRR12280651	16
SRR12280658	17
SRR13269111	3
SRR13269122	10
SRR13269148	16

SRR13269149	7
SRR13269150	1
SRR13269154	16
SRR13269155	16
SRR13269156	7
SRR13269157	10
SRR13269158	6
SRR13269159	1
SRR13269161	4
SRR13269162	8
SRR13269163	7
SRR13269164	4
SRR13269165	6
SRR13269166	11
SRR13269167	16
SRR13269178	3
SRR13269194	16
SRR13269204	10
SRR13269207	3
SRR13269209	15
SRR13269219	6
SRR13269220	4
SRR13269221	10
SRR13269222	17
SRR13269223	16
SRR13269224	16
SRR13269245	4
SRR15283685	16
SRR15283687	8
SRR15283688	15
SRR15283691	10
SRR15283695	3
SRR15283697	16
SRR15283700	5
SRR15283701	16
SRR15283706	3
SRR15283707	7
SRR15283708	17
SRR15283713	14
SRR15283714	3
SRR15283720	4
SRR15283722	5
SRR15283724	3
SRR15283728	6
SRR15283731	16
SRR15283738	16
SRR15283742	9
SRR15283746	12
SRR15283747	4
SRR15283749	16
SRR15283750	11
SRR15283752	16

SRR15283753	4
SRR15283757	3
SRR15283758	16
SRR15283762	5
SRR15283763	8
SRR15283766	10
SRR15283767	9
SRR15283771	5
SRR15283795	4
SRR15283820	4
SRR15283822	16
SRR15283826	16
SRR15283828	7
SRR15283834	11
SRR15283837	17
SRR15283838	16
SRR15283839	10
SRR15283842	17
SRR15283844	3
SRR15283848	12
SRR15283850	7
SRR15283854	7
SRR15283861	10
SRR15283864	16
SRR15283865	8
SRR15283867	12
SRR15283868	16
SRR15283869	7
SRR15283871	3
SRR15283873	16
SRR15283876	11
SRR15283891	4
SRR15283892	7
SRR15283894	10
SRR15283896	3
SRR9206104	16
SRR9206239	3
SRR9206393	3
SchirmerM_2016__G88708__	3
VatanenT_2016__G69155__bi	4
VatanenT_2016__G78526__bi	16
VatanenT_2016__G78527__bi	17
VatanenT_2016__G78583__bi	9
VatanenT_2016__G78592__bi	4
VatanenT_2016__G78604__bi	9
VatanenT_2016__G78605__bi	9
VatanenT_2016__G78619__bi	13
VatanenT_2016__G78646__bi	12
VatanenT_2016__G78669__bi	4
VatanenT_2016__G78676__bi	12
VatanenT_2016__G78715__bi	4
VatanenT_2016__G78716__bi	6

VatanenT_2016__G78739__bi	17
VatanenT_2016__G78794__bi	12
VatanenT_2016__G78795__bi	12
VatanenT_2016__G78826__bi	6
VatanenT_2016__G78890__bi	16
VatanenT_2016__G78910__bi	6
VatanenT_2016__G80158__bi	9
VatanenT_2016__G80290__bi	8
VatanenT_2016__G80297__bi	4
VatanenT_2016__G80299__bi	9
VatanenT_2016__G80313__bi	8
VatanenT_2016__G80388__bi	16
VatanenT_2016__G80399__bi	17
VatanenT_2016__G80406__bi	16
VatanenT_2016__G80458__bi	11
VatanenT_2016__G80469__bi	6
VatanenT_2016__G80499__bi	13
VatanenT_2016__G80507__bi	16
VatanenT_2016__G80518__bi	8
VatanenT_2016__G80532__bi	16
VatanenT_2016__G80556__bi	16
VatanenT_2016__G80584__bi	16
VatanenT_2016__G80590__bi	16
VatanenT_2016__G80592__bi	14
VatanenT_2016__G80601__bi	15
VatanenT_2016__G80602__bi	16
VatanenT_2016__G80611__bi	3
VincentC_2016__MM022.2__l	16
VincentC_2016__MM032.3__l	1
VincentC_2016__MM060.2__l	14
VogtmannE_2016__MMRS62	10
VogtmannE_2016__MMRS68	7
VogtmannE_2016__MMRS72	12
VogtmannE_2016__MMRS77	6
WenC_2017__N016__bin.21	3
WenC_2017__N281__bin.22	10
WenC_2017__RA132__bin.29	8
XieH_2016__YSZC12003_355	10
XieH_2016__YSZC12003_360	10
XieH_2016__YSZC12003_372	10
XieH_2016__YSZC12003_372	4
XieH_2016__YSZC12003_372	16
XieH_2016__YSZC12003_378	10
XieH_2016__YSZC12003_378	16
YuJ_2015__SZAXPI003422-1	12
YuJ_2015__SZAXPI003435-1	11
YuJ_2015__SZAXPI015216-1	8
YuJ_2015__SZAXPI015250-3	11
ZeeviD_2015__PNP_Main_17	6
ZeeviD_2015__PNP_Main_3	16
ZeeviD_2015__PNP_Validatio	6
ZellerG_2014__CCIS5823480	7

TABLE 4

Sample	GC	GC std	Genome size	contigs	Longest scaffold	Longest contig	Mean scaffold length	Mean contig length	Coding density	Translation table	predicted genes	High quality
GCA_000210835.1	0.43	0	5373121	1	5373121	5373121	5373121	5373121	0.88876	11	4433	Yes
GCA_000965785.1	0.43	0	5282232	1	5282232	5282232	5282232	5282232	0.88843	11	4392	Yes
GCA_001286525.1	0.43	0	5188967	1	5188967	5188967	5188967	5188967	0.88721	11	4224	Yes
GCA_001682215.1	0.43	0	4902215	1	4902215	4902214	4902215	4902214	0.89087	11	3977	Yes
GCA_018292185.1	0.43	0	5224217	1	5224217	5224217	5224217	5224217	0.89246	11	4369	Yes
GCA_023702735.1	0.43	0	5369373	1	5369373	5369373	5369373	5369373	0.88661	11	4519	Yes
GCA_000009925.1	0.43	0.0683	5310990	2	5277274	5277274	2655495	2655495	0.88854	11	4416	Yes
GCA_000025985.1	0.43	0.0769	5241700	2	5205140	5205140	2620850	2620850	0.88993	11	4352	Yes
GCA_005706655.1	0.43	0.0769	5241693	2	5205133	5205133	2620847	2620847	0.88983	11	4349	Yes
GCA_008369705.1	0.43	0.0449	5419725	2	5330584	5330584	2709863	2709863	0.88698	11	4512	Yes
GCA_016889925.1	0.43	0.0036	5248938	2	5150703	5150703	2624469	2624469	0.89028	11	4410	Yes
GCA_020091565.1	0.43	0.079	5223334	2	5187754	5187754	2611667	2611667	0.88866	11	4352	Yes
GCA_020097275.1	0.43	0.0769	5236049	2	5199476	5199476	2618025	2618025	0.8899	11	4349	Yes
GCA_000724795.2	0.43	0.0214	5155157	3	5141257	5141257	1718386	1718386	0.89165	11	4223	Yes
GCA_019915805.1	0.44	0.0032	5447306	4	3936861	3936861	1815769	1361522	0.88772	11	4576	Yes
GCA_020297425.1	0.43	0.002	5305407	4	3933042	3933042	1326352	1326352	0.88942	11	4447	Yes
GCA_900445515.1	0.43	0.0551	5315005	5	5018953	5018953	1063001	1063001	0.88868	11	4446	Yes
GCA_014893755.1	0.43	0.0559	5141158	6	4986460	4986460	856859.7	856860	0.89118	11	4268	Yes
GCA_020297585.1	0.43	0.0602	5351678	7	3949773	3949773	764525.4	764525	0.88682	11	4562	Yes
GCA_020297435.1	0.43	0.0335	5428300	8	4110164	4110164	678537.5	678538	0.88606	11	4513	Yes
GCA_020297545.1	0.44	0.0447	5731674	9	1554842	1554842	636852.7	636853	0.88476	11	4871	Yes
GCA_000269525.1	0.43	0.0381	5300647	12	1406492	1406492	441720.6	441721	0.88981	11	4427	Yes
SRR15283861	0.43	0.0243	5173823	16	3035160	3035160	323363.9	323364	0.89435	11	4259	Yes
GCA_902374115.1	0.43	0.0054	5116282	17	3253542	716030	1023256	300887	0.89335	11	4218	Yes
SRR15283722	0.43	0.0427	5212662	18	952378	952378	289592.3	289592	0.8956	11	4329	Yes
SRR15283871	0.43	0.0233	5097397	19	697363	697363	268284.1	268284	0.89384	11	4274	Yes
GCA_020256925.1	0.43	0.022	5201753	19	1433054	1433053	273776.5	273776	0.89368	11	4305	Yes
SRR9205744	0.43	0.0233	5215210	20	1076017	1076017	260760.5	260761	0.89746	11	4368	Yes
GCA_020256835.1	0.43	0.0265	5200575	22	1199563	1199562	236389.8	236390	0.89392	11	4310	Yes
GCA_020257125.1	0.44	0.019	5269890	22	1142084	1142083	239540.5	239540	0.89348	11	4387	Yes
363192571_S62_L001	0.43	0.0354	5101039	24	955006	955006	212543.3	212543	0.89301	11	4181	Yes
SRR15283894	0.43	0.0355	5007900	24	1026914	1026914	208662.5	208663	0.89479	11	4087	Yes
GCA_000263115.1	0.44	0.0323	5423654	24	3868655	1356635	602628.2	225593	0.89082	11	4591	Yes
GCA_020257085.1	0.44	0.0245	5270023	24	1199644	1199643	219584.3	219584	0.89324	11	4380	Yes
GCA_023274315.1	0.43	0.0499	5222967	24	870030	870030	217623.6	217624	0.89399	11	4336	Yes
GCA_023274495.1	0.43	0.0499	5222622	24	870030	870030	217609.3	217609	0.89413	11	4339	Yes
GCA_023276265.1	0.43	0.0495	5222967	24	870030	870030	217623.6	217624	0.89403	11	4338	Yes
GCA_023894135.1	0.43	0.05	5222967	24	870030	870030	217623.6	217624	0.89385	11	4337	Yes
GCA_000273765.1	0.43	0.0038	5290489	25	2641955	1366195	1058098	211177	0.88844	11	4421	Yes
GCA_023276305.1	0.43	0.0496	5223584	25	870030	870030	208943.4	208943	0.89407	11	4341	Yes
BackhedF_2015__SID10_4M__bin.11	0.43	0.0101	5043414	25	1082750	1082750	201736.6	201737	0.89569	11	4193	Yes

363192589_S80_L001	0.43	0.0304	5266040	26	1000651	1000651	202540	202540	0.88939	11	4333	Yes
363193047_S157_L001	0.43	0.0508	5216782	26	1494104	1494104	200645.5	200645	0.89223	11	4332	Yes
GCA_007896795.1	0.43	0.034	5253757	26	1573133	1573133	202067.6	202068	0.89373	11	4464	Yes
GCA_020256775.1	0.43	0.0249	5201880	26	666967	666967	200072.3	200072	0.8936	11	4309	Yes
GCA_023274715.1	0.43	0.0492	5222669	26	865137	865137	200871.9	200872	0.89389	11	4334	Yes
GCA_023274755.1	0.43	0.0487	5223724	26	870030	870030	200912.5	200912	0.89395	11	4341	Yes
GCA_023958435.1	0.43	0.0492	5248878	26	785379	785379	201879.9	201880	0.894	11	4378	Yes
363192605_S96_L001	0.43	0.0308	5394663	27	788304	788304	199802.3	199802	0.89251	11	4561	Yes
GCA_023274775.1	0.43	0.0651	5223116	27	870030	870030	193448.7	193449	0.89399	11	4342	Yes
GCA_023276335.1	0.43	0.0664	5223195	27	794723	794723	193451.7	193452	0.89401	11	4340	Yes
SRR15283758	0.43	0.0395	5291904	28	538566	538566	188996.6	188997	0.89377	11	4428	Yes
363192573_S64_L001	0.43	0.0523	5347630	28	982887	982887	190986.8	190987	0.89349	11	4503	Yes
SRR15283742	0.44	0.0306	5402812	28	1028920	1028920	192957.6	192958	0.89275	11	4532	Yes
SRR9206004	0.43	0.0304	5312468	28	894454	894454	189731	189731	0.89204	11	4408	Yes
SRR15283865	0.43	0.0313	5259902	28	1249856	1249856	187853.6	187854	0.89515	11	4369	Yes
VatanenT_2016__G78583__bin.3	0.44	0.0132	5544569	28	798841	798841	198020.3	198020	0.89384	11	4682	Yes
363192528_S11_L001	0.43	0.03	5324516	29	650512	650512	183604	183604	0.89186	11	4414	Yes
363192579_S70_L001	0.43	0.0415	5100425	29	784976	784976	175876.7	175877	0.8931	11	4184	Yes
SRR15283753	0.43	0.0622	5292784	29	1134116	1134116	182509.8	182510	0.89404	11	4435	Yes
SRR9205639	0.43	0.0396	5211548	29	870959	870959	179708.6	179709	0.89733	11	4368	Yes
GCA_014042525.1	0.43	0.0269	5175039	29	644423	644423	184822.8	178447	0.89344	11	4375	Yes
GCA_024007805.1	0.43	0.049	5226328	29	785379	785379	180218.2	180218	0.89369	11	4342	Yes
SRR15283691	0.43	0.0452	5428278	30	843855	843855	180942.6	180943	0.89118	11	4581	Yes
SRR9206014	0.43	0.0268	5313037	30	596091	596091	177101.2	177101	0.89225	11	4409	Yes
SRR9206177	0.43	0.0393	5221863	30	792598	792598	174062.1	174062	0.89721	11	4382	Yes
363193036_S146_L001	0.43	0.0453	5136680	30	763866	763866	171222.7	171223	0.89574	11	4234	Yes
SRR15283731	0.43	0.0505	5363201	30	1023976	1023976	178773.4	178773	0.89148	11	4559	Yes
SRR15283852	0.43	0.0279	5281413	30	1076432	1076432	176047.1	176047	0.89382	11	4400	Yes
SRR15283869	0.44	0.0311	5183460	30	834136	834136	172782	172782	0.89528	11	4331	Yes
SRR15283854	0.43	0.0349	5272508	30	1075341	1075341	175750.3	175750	0.89389	11	4389	Yes
10317.X00214130_23	0.44	0.0166	4726040	30	426087	426087	157534.7	157535	0.89604	11	3898	Yes
BackhedF_2015__SID180_4M__bin.6	0.43	0.0163	5172115	30	765537	765537	172403.8	172404	0.89479	11	4314	Yes
WenC_2017__RA132__bin.29	0.43	0.0156	4970542	30	734931	734931	165684.7	165685	0.89777	11	4111	Yes
SRR9205813	0.43	0.0365	5217024	31	1169484	1169484	168291.1	168291	0.89742	11	4381	Yes
SRR15283728	0.43	0.0214	5251100	31	1534429	1534429	169390.3	169390	0.89306	11	4382	Yes
SRR12280632	0.43	0.0265	5095029	31	963377	963377	164355.8	164356	0.89569	11	4221	Yes
363193048_S163_L001	0.43	0.053	5097191	31	615219	615219	164425.5	164426	0.89604	11	4249	Yes
MCT420_55_S63_L002	0.43	0.0372	5107594	31	1097965	1097965	164761.1	164761	0.89424	11	4251	Yes
SRR15283896	0.44	0.0379	5276424	31	1137011	1137011	170207.2	170207	0.89325	11	4448	Yes
GCA_000598945.1	0.43	0.0367	5050706	31	798787	798787	162926	162926	0.89278	11	4183	Yes

GCA_003438895.1	0.43	0.0368	5081261	31	1140463	985827	241964.8	163908	0.89504	11	4246	Yes
363193054_S164_L001	0.43	0.0371	5084154	32	811760	811760	158879.8	158880	0.89221	11	4210	Yes
363193069_S179_L001	0.43	0.0255	5069819	32	824591	824591	158431.8	158432	0.89717	11	4215	Yes
GCA_014042415.1	0.43	0.0302	5236934	32	723807	723807	163654.2	163654	0.8941	11	4401	Yes
GCA_022832315.1	0.43	0.0353	5256558	32	1225208	1225208	164267.4	164267	0.89372	11	4468	Yes
10317.X00185752_7	0.43	0.0149	4652999	32	484184	484184	145406.2	145406	0.89327	11	3870	Yes
363193072_S181_L001	0.43	0.0508	5273920	33	849897	849897	159815.8	159816	0.89477	11	4450	Yes
SRR15283795	0.43	0.0291	5304129	33	1035985	1035985	160731.2	160731	0.89262	11	4438	Yes
SRR15283700	0.43	0.0452	5266148	33	664456	664456	159580.2	159580	0.89459	11	4403	Yes
GCA_003438775.1	0.43	0.0292	5079420	33	884988	797565	211642.5	153919	0.89517	11	4241	Yes
GCA_007896745.1	0.43	0.0371	5217758	33	987079	987079	158113.9	158114	0.89579	11	4366	Yes
VogtmannE_2016__MMRS77321055ST-27-0-0__bin.39	0.43	0.0154	5173970	33	595519	595519	156787	156787	0.89583	11	4397	Yes
363192578_S69_L001	0.43	0.0293	5241564	34	805492	805492	154163.6	154164	0.8954	11	4424	Yes
SRR9206010	0.43	0.0424	5219908	34	1020945	1020945	153526.7	153527	0.89725	11	4371	Yes
SRR15283876	0.43	0.0417	5138385	34	791843	791843	151129	151129	0.89651	11	4323	Yes
SRR15283767	0.43	0.0288	5117959	34	902407	902407	150528.2	150528	0.89322	11	4219	Yes
LiJ_2017__H1M213648__bin.18	0.43	0.0106	4955921	34	626091	626091	145762.4	145762	0.89567	11	4091	Yes
GCA_023274895.1	0.43	0.0603	5222451	34	785379	785379	153601.5	153602	0.89403	11	4344	Yes
GCA_023275035.1	0.43	0.0599	5222185	34	794723	794723	153593.7	153594	0.89393	11	4340	Yes
BackhedF_2015__SID180_12M__bin.6	0.43	0.0175	5122742	34	477443	477443	150668.9	150669	0.89519	11	4270	Yes
QinN_2014__LD-93__bin.8	0.43	0.0161	4907877	34	522242	522242	144349.3	144349	0.89781	11	4059	Yes
XieH_2016__YSZC12003_37207R1__bin.36	0.43	0.0136	5041576	34	596915	596915	148281.6	148282	0.89627	11	4226	Yes
SRR15283745	0.43	0.0323	5064627	35	579826	579826	144703.6	144704	0.89396	11	4247	Yes
363192577_S68_L001	0.43	0.0575	5332119	35	931114	931114	152346.3	152346	0.89421	11	4553	Yes
GCA_000273095.1	0.43	0.0232	5238793	35	3104543	765640	654849.1	149310	0.88752	11	4363	Yes
GCA_000273115.1	0.43	0.0453	5226222	35	1812676	698375	746603.1	148972	0.89097	11	4354	Yes
GCA_000273155.1	0.44	0.0411	5444546	35	3416594	984935	494958.7	155149	0.88798	11	4600	Yes
GCA_003469705.1	0.43	0.0162	5112303	35	1059671	1059671	204492.1	146063	0.89636	11	4240	Yes
GCA_019733435.1	0.43	0.042	5282608	35	988989	988989	155370.8	150931	0.89225	11	4486	Yes
BackhedF_2015__SID338_B__bin.1	0.43	0.0135	4825086	35	578066	578066	137859.6	137860	0.89711	11	4033	Yes
ChengpingW_2017__AS123raw__bin.19	0.43	0.0138	4967962	35	768174	768174	141941.8	141942	0.89618	11	4099	Yes
MCT420_59_S67_L002	0.43	0.0339	5276145	36	583174	583174	146559.6	146560	0.89541	11	4433	Yes
MCT420_6_S14_L002	0.43	0.057	5161867	36	893140	893140	143385.2	143385	0.89537	11	4293	Yes
363193027_S137_L001	0.44	0.0311	5322663	36	1031711	1031711	147851.8	147852	0.89196	11	4532	Yes
GCA_000601115.1	0.43	0.0358	5093658	36	1383888	1383888	141490.5	141491	0.89328	11	4220	Yes
GCA_004167855.1	0.43	0.0395	5209622	36	1066996	1066996	144711.7	144712	0.89671	11	4369	Yes
BackhedF_2015__SID503_4M__bin.20	0.43	0.0116	4986413	36	434428	434428	138511.5	138511	0.89717	11	4127	Yes
QinN_2014__LD-50__bin.33	0.43	0.0113	4874015	36	487850	487850	135389.3	135389	0.89796	11	4046	Yes
VogtmannE_2016__MMRS62192467ST-27-0-0__bin.44	0.43	0.0255	5308140	36	1090925	1090925	147448.3	147448	0.89429	11	4453	Yes
XieH_2016__YSZC12003_36052__bin.34	0.43	0.0136	4919008	36	632918	632918	136639.1	136639	0.89275	11	4037	Yes
363192603_S94_L001	0.43	0.0324	4837965	37	1504077	1504077	130755.8	130756	0.89601	11	3958	Yes

MCT420_54_S62_L002	0.43	0.0358	5214072	37	591944	591944	140920.9	140921	0.89515	11	4363	Yes
SRR15283891	0.43	0.0342	4907412	37	930576	930576	132632.8	132633	0.89544	11	4005	Yes
LiJ_2014__V1.CD3-0-PN__bin.42	0.43	0.0145	5072000	37	544262	544262	137081.1	137081	0.89726	11	4241	Yes
GCA_014042465.1	0.43	0.0251	5242965	37	624044	624044	145637.9	141699	0.89144	11	4328	Yes
BackhedF_2015__SID566_12M__bin.34	0.43	0.0139	5027737	37	816747	816747	135884.8	135885	0.89613	11	4197	Yes
MCT420_50_S58_L002	0.44	0.0361	5252897	38	877121	877121	138234.1	138234	0.89293	11	4357	Yes
363192556_S47_L001	0.43	0.0405	5097193	38	656993	656993	134136.7	134137	0.89519	11	4269	Yes
363193064_S174_L001	0.43	0.031	5113098	38	853837	853837	134555.2	134555	0.8954	11	4256	Yes
363193080_S190_L001	0.43	0.0299	5174243	38	636516	636516	136164.3	136164	0.89611	11	4337	Yes
SRR9206233	0.43	0.0593	5262401	38	1413516	1413516	138484.2	138484	0.893	11	4450	Yes
363193056_S166_L001	0.43	0.0404	5246151	38	541376	541376	138056.6	138057	0.89412	11	4424	Yes
10317.X00179017_10317.X00179017_bin.7	0.43	0.0183	5118176	38	462795	462795	134688.8	134689	0.89656	11	4210	Yes
HeQ_2017__RSZAXPI003062-102__bin.4	0.43	0.014	5061928	38	658030	658030	133208.6	133209	0.89403	11	4222	Yes
GCA_023274245.1	0.43	0.0439	5222481	38	785379	785379	137433.7	137434	0.89406	11	4349	Yes
SRR15283701	0.43	0.0269	5238020	39	620339	620339	134308.2	134308	0.89118	11	4326	Yes
SRR15283838	0.43	0.0336	5092021	39	779334	779334	130564.6	130565	0.89511	11	4246	Yes
SRR15283749	0.43	0.0363	5063824	39	516235	516235	129841.6	129842	0.89397	11	4247	Yes
BackhedF_2015__SID615_4M__bin.5	0.43	0.0163	5230883	39	629970	629970	134125.2	134125	0.89471	11	4430	Yes
CM_madagascar__A39_02_1FE__bin.19	0.43	0.02	5019571	39	505761	505761	128706.9	128707	0.89564	11	4136	Yes
BackhedF_2015__SID238_B__bin.1	0.43	0.0149	5013476	39	575799	575799	128550.7	128551	0.89413	11	4182	Yes
WenC_2017__N016__bin.21	0.43	0.0186	4910020	39	557539	557539	125897.9	125898	0.89498	11	4048	Yes
YuJ_2015__SZAXPI003435-11__bin.64	0.43	0.0175	4654889	39	737845	737845	119356.1	119356	0.89859	11	3811	Yes
363193060_S170_L001	0.43	0.0369	5182939	40	657878	657878	129573.5	129573	0.89239	11	4267	Yes
363193061_S171_L001	0.43	0.0319	5281900	40	692005	692005	132047.5	132048	0.8943	11	4436	Yes
SRR9206104	0.43	0.0298	5257731	40	772616	772616	131443.3	131443	0.89597	11	4459	Yes
363193045_S155_L001	0.43	0.0347	5230741	40	822814	822814	130768.5	130769	0.89456	11	4406	Yes
363193035_S145_L001	0.43	0.038	5258822	40	1150066	1150066	131470.6	131471	0.89343	11	4409	Yes
SRR15283699	0.43	0.0542	5266862	40	653024	653024	131671.6	131672	0.89448	11	4403	Yes
MCT420_75_S83_L002	0.43	0.0369	5042568	40	918696	918696	126064.2	126064	0.89406	11	4155	Yes
GCA_000598765.2	0.43	0.0368	4955059	40	896013	896013	123876.5	123876	0.89206	11	4051	Yes
GeversD_2014__SKSTL0001__bin.2	0.44	0.0171	4949112	40	872388	872388	123727.8	123728	0.89831	11	4085	Yes
GCA_015556305.1	0.44	0.0291	5516929	40	684676	524413	167179.7	137906	0.89278	11	4705	Yes
BackhedF_2015__SID567_12M__bin.4	0.43	0.0152	5023171	40	403427	403427	125579.3	125579	0.89578	11	4138	Yes
10317.X00182267_21	0.43	0.0342	4888919	40	486553	486553	122223	122223	0.89559	11	4033	Yes
XieH_2016__YSZC12003_35524__bin.22	0.43	0.0163	4945572	40	616341	616341	123639.3	123639	0.89581	11	4063	Yes
SRR15283708	0.43	0.0383	5311239	41	582625	582625	129542.4	129542	0.89239	11	4529	Yes
SRR15283704	0.44	0.0264	5185063	41	746771	746771	126465	126465	0.89489	11	4323	Yes
SRR15283837	0.43	0.0476	5532683	41	693158	693158	134943.5	134943	0.8919	11	4768	Yes
MCT420_18_S26_L002	0.43	0.0561	5261492	41	848585	848585	128329.1	128329	0.89338	11	4409	Yes
363192520_S3_L001	0.43	0.0432	5224857	41	1169572	1169572	127435.5	127436	0.89729	11	4386	Yes
SRR15283868	0.43	0.0358	5388892	41	619355	619355	131436.4	131436	0.89124	11	4516	Yes
SRR12280314	0.43	0.0406	4993204	41	603692	603692	121785.5	121785	0.89672	11	4080	Yes

GCA_003436935.1	0.43	0.0286	5253326	41	938005	938005	187618.8	128122	0.89277	11	4405	Yes
GCA_014042445.1	0.43	0.0278	5243270	41	1098546	1098546	131081.8	127882	0.89442	11	4369	Yes
GCA_014042505.1	0.43	0.035	5259406	41	872623	872623	128278.2	128278	0.89341	11	4414	Yes
GCA_022780605.1	0.44	0.0238	5226845	41	478500	478500	127484	127484	0.89576	11	4387	Yes
BackhedF_2015__SID565_4M__bin.10	0.43	0.0155	5025962	41	603493	603493	122584.4	122584	0.89677	11	4100	Yes
10317.X00180334_10317.X00180334_bin.1	0.43	0.0227	4972686	41	568477	568477	121285	121285	0.89515	11	4095	Yes
GCA_905188165.1	0.43	0.0228	5063915	41	614932	614932	123510.1	123510	0.89257	11	4221	Yes
BackhedF_2015__SID275_B__bin.6	0.43	0.0181	4870982	41	577323	577323	118804.4	118804	0.89519	11	4063	Yes
QinN_2014__LD-34__bin.5	0.43	0.0166	4996114	41	417440	417440	121856.4	121856	0.89678	11	4161	Yes
363192564_S55_L001	0.43	0.0362	5098122	42	956502	956502	121383.9	121384	0.89554	11	4277	Yes
MCT420_10_S18_L002	0.43	0.0319	5129356	42	779034	779034	122127.5	122128	0.89325	11	4288	Yes
SRR15283842	0.43	0.0348	5244046	42	765652	765652	124858.2	124858	0.89293	11	4411	Yes
363193044_S154_L001	0.43	0.0296	5289519	42	783852	783852	125940.9	125941	0.89452	11	4433	Yes
363193071_S180_L001	0.43	0.034	5134395	42	537537	537537	122247.5	122248	0.89391	11	4263	Yes
SRR15283687	0.43	0.0574	5454725	42	583796	583796	129874.4	129874	0.89379	11	4624	Yes
GCA_009025705.1	0.43	0.0441	5224890	42	987079	987079	124402.1	124402	0.89522	11	4380	Yes
GCA_022832355.1	0.43	0.0425	5268612	42	954953	954953	125443.1	125443	0.89556	11	4384	Yes
BackhedF_2015__SID521_4M__bin.12	0.43	0.0272	5043474	42	783864	783864	120082.7	120083	0.89723	11	4205	Yes
BackhedF_2015__SID53_4M__bin.14	0.43	0.0166	4914465	42	603217	603217	117011.1	117011	0.89652	11	4065	Yes
BackhedF_2015__SID546_4M__bin.5	0.43	0.0222	5232047	42	508724	508724	124572.5	124573	0.89611	11	4478	Yes
MCT420_60_S68_L002	0.43	0.0442	5301810	43	906050	906050	123297.9	123298	0.89277	11	4518	Yes
363192583_S74_L001	0.43	0.0388	5269073	43	485586	485586	122536.6	122537	0.89175	11	4436	Yes
SRR15283815	0.44	0.0466	5340707	43	1081287	1081287	124202.5	124202	0.89343	11	4533	Yes
SRR15283887	0.43	0.0468	5295371	43	690854	690854	123148.2	123148	0.89489	11	4464	Yes
GCA_000297735.1	0.43	0.0455	5419684	43	2054707	854036	387120.3	124610	0.87862	11	4501	Yes
GCA_012844105.1	0.43	0.0351	5289120	43	602508	602508	123002.8	123003	0.89446	11	4451	Yes
GCA_015553495.1	0.43	0.0393	5311203	43	776247	776247	129541.5	123512	0.89691	11	4465	Yes
10317.X00179673_25	0.43	0.0197	4615473	43	584668	584668	107336.6	107337	0.89711	11	3773	Yes
363193021_S134_L001	0.43	0.0372	5152419	44	969142	969142	117100.4	117100	0.89322	11	4283	Yes
363197827_S378_L001	0.43	0.0263	5056110	44	577742	577742	114911.6	114912	0.89505	11	4180	Yes
MCT420_36_S44_L002	0.43	0.0364	5359090	44	665920	665920	121797.5	121798	0.89097	11	4464	Yes
MCT420_63_S71_L002	0.43	0.0473	5335593	44	827132	827132	121263.5	121263	0.89389	11	4467	Yes
GCA_000598965.1	0.43	0.0431	5062782	44	977923	977923	115063.2	115063	0.89323	11	4200	Yes
GCA_003439635.1	0.44	0.0334	5340087	44	780270	746828	161820.8	121363	0.89321	11	4507	Yes
GCA_008569395.1	0.43	0.0289	5306916	44	672184	672184	120611.7	120612	0.89216	11	4419	Yes
BackhedF_2015__SID582_12M__bin.6	0.43	0.0153	4883564	44	689896	689896	110990.1	110990	0.89842	11	4071	Yes
BackhedF_2015__SID130_4M__bin.3	0.43	0.0195	5213805	44	396291	396291	118495.6	118496	0.89353	11	4382	Yes
363192598_S89_L001	0.43	0.0238	5058222	45	577766	577766	112404.9	112405	0.8949	11	4179	Yes
SRR12280417	0.43	0.0303	5305184	45	421667	421667	117893	117893	0.89341	11	4467	Yes
363192587_S78_L001	0.43	0.0347	5291757	45	621002	621002	117594.6	117595	0.89377	11	4464	Yes
363193055_S165_L001	0.43	0.0332	5133397	45	518873	518873	114075.5	114075	0.89406	11	4264	Yes
SRR15283750	0.43	0.0488	5406207	45	604934	604934	120137.9	120138	0.8936	11	4633	Yes
GCA_003439675.1	0.44	0.0278	5398873	45	780248	777677	179962.4	119972	0.8929	11	4596	Yes
10317.X00179225_54	0.43	0.026	4700075	45	389607	389607	104446.1	104446	0.89668	11	3870	Yes

10317.X00185882_3	0.43	0.0292	5173944	45	535796	535796	114976.5	114977	0.89586	11	4381	Yes
XieH_2016__YSZC12003_37269__bin.22	0.43	0.0164	4867227	45	560751	560751	108160.6	108161	0.89513	11	4007	Yes
ZeeviD_2015__PNP_Main_319__bin.2	0.43	0.02	4934868	45	589804	589804	109663.7	109664	0.896	11	4113	Yes
363193062_S172_L001	0.43	0.0267	5328569	46	723728	723728	115838.5	115838	0.89317	11	4433	Yes
SRR15283841	0.43	0.0354	5244772	46	780400	780400	114016.8	114017	0.89276	11	4413	Yes
363193073_S182_L001	0.44	0.0309	5403818	46	843909	843909	117474.3	117474	0.89462	11	4604	Yes
SRR15283873	0.43	0.0507	5288169	46	1107819	1107819	114960.2	114960	0.89146	11	4505	Yes
SRR15283746	0.44	0.0514	5514938	46	697842	697842	119890	119890	0.89246	11	4652	Yes
SRR15283890	0.43	0.0451	5293354	46	1291085	1291085	115072.9	115073	0.89466	11	4462	Yes
GCA_008569445.1	0.43	0.0295	5306094	46	671996	671996	115349.9	115350	0.89188	11	4420	Yes
KosticAD_2015__PRJNA231909.3113831__bin.8	0.43	0.0284	5094387	46	630547	630547	110747.5	110748	0.8961	11	4230	Yes
BackhedF_2015__SID570_4M__bin.7	0.43	0.0256	5031934	46	623182	623182	109389.9	109390	0.89692	11	4214	Yes
BackhedF_2015__SID591_4M__bin.17	0.43	0.0182	4930818	46	457903	457903	107191.7	107192	0.89635	11	4089	Yes
BackhedF_2015__SID626_4M__bin.17	0.43	0.0173	4842436	46	506154	506154	105270.3	105270	0.89697	11	3960	Yes
10317.X00179729_16	0.43	0.0313	4859808	46	379554	379554	105648	105648	0.89502	11	4010	Yes
SRR15283705	0.44	0.0381	5183542	47	746479	746479	110288.1	110288	0.89508	11	4330	Yes
SRR9206391	0.43	0.0592	5117047	47	580050	580050	108873.3	108873	0.89296	11	4282	Yes
SRR15283820	0.44	0.0511	5398463	47	1145467	1145467	114860.9	114861	0.89466	11	4619	Yes
363192595_S86_L001	0.43	0.0364	5281836	47	621026	621026	112379.5	112379	0.89467	11	4455	Yes
GCA_007896675.1	0.44	0.0485	5260633	47	754196	754196	111928.4	111928	0.89524	11	4391	Yes
GCA_015548825.1	0.43	0.031	5157891	47	783997	731029	139402.5	109722	0.89256	11	4312	Yes
BackhedF_2015__SID130_12M__bin.13	0.43	0.0189	5252316	47	349289	349289	111751.4	111751	0.89318	11	4423	Yes
363193078_S188_L001	0.43	0.0331	5145113	48	504438	504438	107189.9	107190	0.8942	11	4280	Yes
363192585_S76_L001	0.43	0.0548	5488435	48	945845	945845	114342.4	114342	0.89245	11	4731	Yes
363192581_S72_L001	0.43	0.0465	5233050	48	662764	662764	109021.9	109022	0.89262	11	4431	Yes
SRR15283867	0.44	0.0447	5347211	48	525780	525780	111400.2	111400	0.89284	11	4536	Yes
363193049_S158_L001	0.43	0.0306	5243410	48	578020	578020	109237.7	109238	0.89336	11	4411	Yes
363193079_S189_L001	0.44	0.0425	5342014	48	664604	664604	111292	111292	0.89422	11	4477	Yes
GCA_003436285.1	0.43	0.0408	5195045	48	666198	666198	157425.6	108227	0.89394	11	4371	Yes
GCA_008569415.1	0.43	0.0316	5315029	48	954181	954181	110729.8	110730	0.89199	11	4426	Yes
GCA_022782465.1	0.43	0.0256	4969778	48	633813	633813	103537	103537	0.89514	11	4061	Yes
DavidLA_2015__LD-Run1-08__bin.7	0.43	0.0248	5082289	48	544632	544632	105881	105881	0.89616	11	4252	Yes
BackhedF_2015__SID78_4M__bin.8	0.43	0.0252	5168151	48	368033	368033	107669.8	107670	0.89332	11	4300	Yes
10317.X00179805_8	0.43	0.0288	4811210	48	545780	545780	100233.5	100234	0.89814	11	3940	Yes
10317.X00180160_15	0.43	0.0261	4590063	48	517635	517635	95626.31	95626.3	0.89612	11	3779	Yes
10317.X00180243_84	0.43	0.0309	4717767	48	429386	429386	98286.81	98286.8	0.89796	11	3817	Yes
10317.X00215482_24	0.43	0.023	5096615	48	433118	433118	106179.5	106179	0.89166	11	4250	Yes
QinN_2014__LD-33__bin.18	0.43	0.0151	5168686	48	363875	363875	107681	107681	0.89222	11	4296	Yes
VatanenT_2016__G78526__bin.12	0.43	0.0187	4897460	48	540695	540695	102030.4	102030	0.89443	11	4071	Yes
SRR15283834	0.43	0.0284	5078554	49	590978	590978	103644	103644	0.89617	11	4242	Yes
SRR15283720	0.43	0.0392	5194534	49	815178	815178	106010.9	106011	0.89249	11	4363	Yes
SRR15283747	0.43	0.0421	5486178	49	701596	701596	111962.8	111963	0.89253	11	4717	Yes

GCA_007896685.1	0.44	0.0479	5259515	49	960932	960932	107337	107337	0.89535	11	4391	Yes
GCA_008569305.1	0.43	0.0346	5115137	49	595308	595308	104390.6	104390	0.89254	11	4281	Yes
HeQ_2017__RSZAXPI003078-111__bin.2	0.43	0.0175	4738815	49	483335	483335	96710.51	96710.5	0.89826	11	3899	Yes
10317.X00179327_33	0.43	0.025	4859629	49	471046	471046	99176.1	99176.1	0.89578	11	3992	Yes
SRR15283844	0.43	0.0585	5304140	50	590619	590619	106082.8	106083	0.89309	11	4561	Yes
SRR15283717	0.44	0.0574	5341074	50	842092	842092	106821.5	106821	0.89369	11	4543	Yes
SRR15283771	0.43	0.0343	5271572	50	711108	711108	105431.4	105431	0.8949	11	4386	Yes
GCA_003472025.1	0.43	0.0567	5292183	50	796128	796128	132304.6	105842	0.89419	11	4449	Yes
GCA_015551365.1	0.43	0.039	5277783	50	706552	465506	125661.5	105538	0.89017	11	4431	Yes
10317.X00179381_10317.X00179381_bin.18	0.43	0.0211	4908265	50	412942	412942	98165.3	98165.3	0.89375	11	4074	Yes
10317.X00179487_25	0.43	0.0294	4967304	50	381420	381420	99346.08	99346.1	0.8937	11	4158	Yes
VatanenT_2016__G80584__bin.1	0.44	0.0247	4959988	50	514344	514344	99199.76	99199.8	0.89656	11	4131	Yes
SRR15283864	0.43	0.0408	5187228	51	803537	803537	101710.4	101710	0.89467	11	4386	
SRR12280504	0.43	0.0376	5385023	51	823975	823975	105588.7	105589	0.89474	11	4546	
SRR13269111	0.43	0.0373	5016130	51	621617	621617	98355.49	98355.5	0.89305	11	4119	
MCT420_81_S89_L002	0.43	0.0521	5248793	51	765087	765087	102917.5	102918	0.89293	11	4421	
10317.X00214127_44_sub	0.44	0.0342	4856789	51	433923	433923	95231.16	95231.2	0.89499	11	4008	
10317.X00215088_24	0.43	0.0364	4952210	51	509440	509440	97102.16	97102.2	0.89397	11	4081	
VatanenT_2016__G80469__bin.5	0.43	0.0203	5072263	51	591468	591468	99456.14	99456.1	0.89575	11	4220	
VogtmannE_2016__MMRS68910755ST-27-0-0__bin.44	0.43	0.0165	5038567	51	498511	498511	98795.43	98795.4	0.89555	11	4197	
ZeeviD_2015__PNP_Main_174__bin.22	0.43	0.0172	5041409	51	581022	581022	98851.16	98851.2	0.89528	11	4192	
SRR9206239	0.44	0.0459	4998306	52	688851	688851	96121.27	96121.3	0.89355	11	4179	
SRR15283737	0.43	0.0462	5486682	52	701596	701596	105513.1	105513	0.8924	11	4721	
363192591_S82_L001	0.43	0.059	5259391	52	981181	981181	101142.1	101142	0.89242	11	4395	
SRR15283826	0.43	0.0449	5499506	52	667537	667537	105759.7	105760	0.89293	11	4738	
MCT420_56_S64_L002	0.43	0.0396	5329574	52	584297	584297	102491.8	102492	0.8941	11	4465	
GCA_001699855.1	0.43	0.0374	5212804	52	639633	639631	100246.2	100246	0.89361	11	4389	
BackhedF_2015__SID137_4M__bin.2	0.44	0.0227	5390786	52	491206	491206	103669	103669	0.896	11	4604	
ZellerG_2014__CCIS58234805ST-4-0__bin.17	0.43	0.0161	4923447	52	307212	307212	94681.67	94681.7	0.89563	11	4111	
SRR13269155	0.43	0.0355	5107524	53	647925	647925	96368.38	96368.4	0.89207	11	4266	
363193028_S138_L001	0.44	0.0377	5409853	53	646130	646130	102072.7	102073	0.89192	11	4679	
MCT420_32_S40_L002	0.43	0.0343	5055617	53	445876	445876	95389	95389	0.89497	11	4183	
363192604_S95_L001	0.43	0.0412	5161173	53	895122	895122	97380.62	97380.6	0.89518	11	4339	
LiJ_2014__V1.FI21__bin.42	0.43	0.0169	4712650	53	454629	454629	88917.92	88917.9	0.89838	11	3852	
GCA_009025495.1	0.43	0.0445	5276079	53	1226009	1226009	99548.66	99548.7	0.89211	11	4485	
GCA_014042455.1	0.43	0.0346	5125231	53	501904	501904	96702.47	96702.5	0.89401	11	4258	
10317.X00180464_10317.X00180464_bin.2	0.43	0.028	5049351	53	438779	438779	95270.77	95270.8	0.89525	11	4162	
RaymondF_2016__P22E7__bin.4	0.43	0.0264	5236954	53	831735	831735	98810.45	98810.5	0.89565	11	4366	
VatanenT_2016__G80590__bin.4	0.44	0.0215	4848538	53	562902	562902	91481.85	91481.8	0.89813	11	4057	
363192602_S93_L001	0.43	0.0371	5225341	54	962460	962460	96765.57	96765.6	0.89375	11	4370	

GCA_000599065.2	0.43	0.0467	5428950	54	1327911	1327911	100536.1	100536	0.88941	11	4595
GCA_008569225.1	0.43	0.0358	5130787	54	1016092	570869	125141.1	94983	0.89132	11	4292
GCA_008569265.1	0.43	0.0385	5261198	54	470138	470138	97429.59	97429.4	0.89311	11	4471
KosticAD_2015__PRJNA231909.3101436__bin.15	0.43	0.0216	4873329	54	379017	379017	90246.83	90246.8	0.89501	11	4056
KosticAD_2015__PRJNA231909.3113834__bin.5	0.43	0.0251	5134800	54	372816	372816	95088.89	95088.9	0.89631	11	4297
GCA_009025695.1	0.43	0.0406	5235981	54	987079	987079	96962.61	96962.6	0.89501	11	4392
GCA_015553755.1	0.43	0.0301	5084960	54	530673	516569	103774.7	94157.3	0.89395	11	4233
GCA_020859785.1	0.43	0.0271	5040691	54	417605	417605	100813.8	93338.7	0.89706	11	4182
BackhedF_2015__SID503_12M__bin.45	0.43	0.0204	5039341	54	500941	500941	93321.13	93321.1	0.89676	11	4207
QinN_2014__LD-35__bin.21	0.43	0.0158	4890870	54	486113	486113	90571.67	90571.7	0.89604	11	4069
363193059_S169_L001	0.43	0.0443	5301778	55	495539	495539	96395.96	96396	0.89233	11	4465
GCA_008569335.1	0.43	0.0275	5132025	55	573524	572748	135053.3	93268.7	0.89004	11	4284
GCA_008569365.1	0.43	0.0353	5135047	55	660807	592294	160470.2	93294.2	0.89005	11	4287
BackhedF_2015__SID526_4M__bin.6	0.43	0.0169	5033915	55	608508	608508	91525.73	91525.7	0.89419	11	4179
363192557_S48_L001	0.44	0.0465	5748111	56	597671	597671	102644.8	102645	0.89435	11	4947
SRR13269122	0.43	0.0385	5141338	56	689722	689722	91809.61	91809.6	0.89513	11	4307
SRR15283748	0.43	0.0502	5291840	56	624888	624888	94497.14	94497.1	0.89403	11	4441
SRR15283888	0.43	0.0442	5267865	56	921768	921768	94069.02	94069	0.89444	11	4452
GCA_003439285.1	0.43	0.0391	4990390	56	1041720	1018539	124759.8	89111.3	0.89284	11	4124
GCA_003475385.1	0.43	0.0574	5306467	56	710298	661446	126344.5	94754.3	0.89431	11	4501
GCA_008569055.1	0.44	0.0447	4994175	56	624982	624982	92484.72	89177.2	0.89314	11	4172
HMP_2012__SRS018817__bin.4	0.43	0.0213	4794025	56	401549	401549	85607.59	85607.6	0.89885	11	4052
BackhedF_2015__SID10_12M__bin.4	0.43	0.0223	5013023	56	452014	452014	89518.27	89518.3	0.89518	11	4170
XieH_2016__YSZC12003_37879__bin.30	0.43	0.0198	4830092	56	310788	310788	86251.64	86251.6	0.89543	11	3966
MCT420_76_S84_L002	0.43	0.0332	5250894	57	499884	499884	92120.95	92120.9	0.8944	11	4430
SRR12280647	0.43	0.031	5201070	57	1150203	1150203	91246.84	91246.8	0.89338	11	4319
SRR13269166	0.43	0.0366	5375745	57	714225	714225	94311.32	94311.3	0.89348	11	4603
363192594_S85_L001	0.43	0.0445	5217410	57	985708	985708	91533.51	91533.5	0.89403	11	4362
SRR15283714	0.43	0.0352	5083834	57	696525	696525	89190.07	89190.1	0.89298	11	4221
10317.X00179153_19	0.43	0.0279	4822252	57	460191	460191	84600.91	84600.9	0.89621	11	3916
SRR15283848	0.43	0.0377	5243692	58	359505	359505	90408.48	90408.5	0.89242	11	4403
SRR12280646	0.43	0.0364	5124882	58	477883	477883	88360.03	88360	0.89206	11	4300
SRR9206387	0.43	0.0429	5253122	58	840542	840542	90571.07	90571.1	0.8932	11	4460
363193032_S142_L001	0.43	0.0361	5111376	58	641690	641690	88127.17	88127.2	0.89609	11	4242
SRR12280270	0.43	0.0349	4957814	58	588757	588757	85479.55	85479.6	0.89529	11	4092
SRR13269220	0.43	0.0264	5260779	58	736451	736451	90703.09	90703.1	0.89336	11	4439
10317.X00179300_11	0.43	0.0277	5023048	58	512679	512679	86604.28	86604.3	0.89602	11	4150
10317.X00185732_3	0.43	0.0252	4764727	58	299766	299766	82150.47	82150.5	0.89443	11	3984
VatanenT_2016__G80507__bin.5	0.43	0.0224	5019887	58	368165	368165	86549.78	86549.8	0.8927	11	4180
SRR15283762	0.43	0.0365	5198853	59	1127562	1127562	88116.15	88116.2	0.89428	11	4323
SRR15283839	0.44	0.0452	5342869	59	479015	479015	90557.1	90557.1	0.89343	11	4537
GCA_000273135.1	0.43	0.0215	5281556	59	2643952	421282	406273.5	89363.7	0.89104	11	4424
GCA_001997325.1	0.43	0.0474	5210800	59	600562	600560	89841.38	88317.2	0.89457	11	4361

LiJ_2014__MH0221__bin.8	0.43	0.0332	5072167	59	611063	611063	85968.93	85968.9	0.89566	11	4253
SRR15283763	0.43	0.0519	5329171	60	512008	512008	88819.52	88819.5	0.89378	11	4565
SRR13269224	0.43	0.0459	5144466	60	575315	575315	85741.1	85741.1	0.89276	11	4280
363192592_S83_L001	0.44	0.0432	5339906	60	678351	678351	88998.43	88998.4	0.8949	11	4512
SRR15283892	0.43	0.0269	5289140	60	713576	713576	88152.33	88152.3	0.89448	11	4462
SRR13269207	0.43	0.0389	5267911	60	620776	620776	87798.52	87798.5	0.89418	11	4495
10317.X00179093_41	0.43	0.0233	4896109	60	328357	328357	81601.82	81601.8	0.89707	11	4037
10317.X00179651_55	0.43	0.0351	4919983	60	383158	383158	81999.72	81999.7	0.89574	11	4128
10317.X00185507_10317.X00185507_bin.17	0.44	0.0227	4670353	60	266770	266770	77839.22	77839.2	0.89547	11	3869
SRR15283707	0.43	0.0444	5442936	61	667446	667446	89228.46	89228.5	0.89316	11	4627
SRR13269158	0.43	0.0357	5262113	61	508149	508149	86264.15	86264.1	0.89371	11	4407
GCA_003437815.1	0.43	0.0356	5062331	61	592937	592937	117728.6	82985.7	0.89496	11	4184
LiJ_2017__H3M515950__bin.24	0.43	0.0227	4873159	61	399893	399893	79887.85	79887.9	0.89437	11	3983
BackhedF_2015__SID615_12M__bin.2	0.43	0.0222	5350040	61	407454	407454	87705.57	87705.6	0.89401	11	4532
10317.X00179954_75	0.43	0.0259	4861814	61	585659	585659	79701.87	79701.9	0.8966	11	4012
BackhedF_2015__SID275_M__bin.26	0.43	0.0192	5097126	61	427690	427690	83559.44	83559.4	0.89467	11	4320
363192568_S59_L001	0.43	0.0539	5255215	62	856554	856554	84761.53	84761.5	0.89447	11	4412
SRR12280549	0.43	0.0364	5213300	62	525336	525336	84085.48	84085.5	0.89463	11	4341
SRR13269245	0.43	0.042	5210448	62	699508	699508	84039.48	84039.5	0.8941	11	4343
363193053_S162_L001	0.43	0.0267	5292276	62	452027	452027	85359.29	85359.3	0.89327	11	4475
SRR15283688	0.44	0.0392	5601081	62	472154	472154	90340.02	90340	0.89462	11	4893
GCA_005844845.1	0.44	0.0354	5684644	62	907546	825121	111463.6	91670.4	0.892	11	4922
GCA_015548595.1	0.44	0.0354	5684644	62	907546	825121	111463.6	91670.4	0.892	11	4922
VatanenT_2016__G78794__bin.3	0.43	0.0239	4965237	62	454975	454975	80084.47	80084.5	0.89586	11	4086
363192548_S39_L001	0.44	0.0532	5276297	63	714588	714588	83750.75	83750.7	0.89515	11	4413
SRR12280619	0.43	0.0522	5127983	63	501324	501324	81396.56	81396.6	0.89376	11	4300
MCT420_41_S49_L002	0.43	0.0347	5186363	63	470848	470848	82323.22	82323.2	0.8956	11	4381
SRR12280522	0.43	0.0408	5311478	63	578269	578269	84309.17	84309.2	0.89418	11	4449
GCA_000598705.1	0.43	0.0424	5117277	63	1218737	1218737	81226.62	81226.6	0.88651	11	4189
GCA_003471925.1	0.43	0.0558	5295367	63	795779	766008	129155.3	84050	0.89402	11	4449
10317.X00179025_65	0.43	0.0356	4969418	63	375703	375703	78879.65	78879.7	0.89415	11	4196
BackhedF_2015__SID546_12M__bin.30	0.43	0.0223	5111435	63	291121	291121	81133.89	81133.9	0.89603	11	4272
BackhedF_2015__SID1_4M__bin.8	0.44	0.0235	4957378	63	362152	362152	78688.54	78688.5	0.89521	11	4118
BackhedF_2015__SID203_12M__bin.9	0.43	0.0182	4707199	63	600589	600589	74717.44	74717.4	0.89698	11	3864
BackhedF_2015__SID238_12M__bin.7	0.43	0.0284	5143167	63	465080	465080	81637.57	81637.6	0.89263	11	4335
363192601_S92_L001	0.43	0.0377	5292929	64	1286752	1286752	82702.02	82702	0.89431	11	4455
363193076_S185_L001	0.44	0.0411	5284468	64	631589	631589	82569.81	82569.8	0.89597	11	4467
SRR13269194	0.44	0.0374	5400263	64	753463	753463	84379.11	84379.1	0.8937	11	4560
GCA_000162135.1	0.43	0.0331	5236303	64	2152962	332517	290905.7	81401.5	0.891	11	4418
GCA_000598585.1	0.43	0.0556	5591413	64	643870	643870	87365.83	87365.8	0.89002	11	4810
GCA_000598725.1	0.43	0.0396	4986076	64	650508	650508	77907.44	77907.4	0.8905	11	4227
GCA_000598885.1	0.43	0.0378	5520411	64	818835	818835	86256.42	86256.4	0.89013	11	4734
GCA_007896595.1	0.44	0.0334	5683897	64	479420	479420	88810.89	88810.9	0.8919	11	4981

VatanenT_2016__G80602__bin.2	0.43	0.022	4854523	64	484820	484820	75851.92	75851.9	0.89623	11	4008
MCT420_49_S57_L002	0.43	0.0373	5151697	65	471905	471905	79256.88	79256.9	0.89465	11	4301
SRR12280511	0.43	0.0447	5384617	65	396671	396671	82840.26	82840.3	0.89316	11	4532
363192558_S49_L001	0.43	0.0433	5347050	65	814024	814024	82262.31	82262.3	0.89621	11	4567
GCA_000599325.1	0.43	0.0407	5249753	65	780956	780956	80765.43	80765.4	0.89014	11	4369
BackhedF_2015__SID137_12M__bin.25	0.43	0.0234	4950150	65	421539	421539	76156.15	76156.2	0.89724	11	4173
Obregon-TitoAJ_2015__NO05__bin.48	0.43	0.0173	5249687	65	402866	402866	80764.42	80764.4	0.89708	11	4445
ZellerG_2014__CCIS76845094ST-20-0__bin.49	0.43	0.0212	4759189	65	526406	526406	73218.29	73218.3	0.8967	11	3941
SRR15283757	0.44	0.0565	5076989	66	550492	550492	76924.08	76924.1	0.89203	11	4207
SRR12280500	0.44	0.0482	5512482	66	525230	525230	83522.45	83522.5	0.89271	11	4734
363193040_S150_L001	0.43	0.0478	5329594	66	663153	663153	80751.42	80751.4	0.89352	11	4503
SRR15283713	0.44	0.0365	5370415	66	624244	624244	81369.92	81369.9	0.89418	11	4604
363193068_S178_L001	0.44	0.0313	5395355	66	452051	452051	81747.8	81747.8	0.89365	11	4559
GCA_003464385.1	0.43	0.0444	5170789	66	951093	951093	114906.4	78340.4	0.89415	11	4305
LiuW_2016__SRR3993044__bin.123	0.44	0.0183	4791048	66	257783	257783	72591.64	72591.6	0.8972	11	3949
QinJ_2012__CON-005__bin.52	0.43	0.0152	4929778	66	375117	375117	74693.61	74693.6	0.896	11	4093
SRR13269163	0.43	0.0366	5081849	67	586720	586720	75848.49	75848.5	0.89403	11	4227
SRR12280503	0.43	0.0444	5135116	67	706518	706518	76643.52	76643.5	0.89281	11	4325
SRR13269156	0.44	0.03	5247036	67	981737	981737	78313.97	78314	0.8945	11	4435
10317.X00214096_10317.X00214096_bin.002	0.43	0.0494	4779386	67	555700	555700	71334.12	71334.1	0.89427	11	3932
363192566_S57_L001	0.43	0.0431	5351244	68	814024	814024	78694.76	78694.8	0.89636	11	4575
363192600_S91_L001	0.44	0.0403	5339819	68	658276	658276	78526.75	78526.8	0.89475	11	4516
GCA_001699875.1	0.43	0.049	4896431	68	754132	754132	72006.34	72006.1	0.89296	11	4042
KosticAD_2015__PRJNA231909.3112348__bin.8	0.43	0.0224	5009102	68	466537	466537	73663.26	73663.3	0.89651	11	4165
LeChatelierE_2013__MH0193__bin.44	0.43	0.0259	4964103	68	373913	373913	73001.51	73001.5	0.89694	11	4106
BackhedF_2015__SID133_4M__bin.23	0.43	0.0229	5157709	68	432262	432262	75848.66	75848.7	0.89707	11	4340
BackhedF_2015__SID150_12M__bin.19	0.43	0.0204	5067669	68	389556	389556	74524.54	74524.5	0.89524	11	4277
VatanenT_2016__G78716__bin.3	0.43	0.0212	5107940	68	789071	789071	75116.76	75116.8	0.89621	11	4318
VatanenT_2016__G80592__bin.1	0.44	0.0259	5155849	68	452365	452365	75821.31	75821.3	0.89808	11	4331
SRR13269154	0.43	0.0416	5097680	69	934893	934893	73879.42	73879.4	0.89534	11	4251
SRR15283825	0.43	0.0513	5493676	69	499506	499506	79618.49	79618.5	0.8931	11	4751
363192794_S99_L001	0.43	0.0301	5290778	69	452028	452028	76677.94	76677.9	0.89342	11	4480
SRR15283766	0.43	0.0513	5410104	69	558600	558600	78407.3	78407.3	0.89183	11	4471
GCA_003458955.1	0.43	0.0425	5224118	69	1074800	1074800	130603	75705	0.89466	11	4400
10317.X00214097_10317.X00214097_bin.3	0.43	0.023	4823347	69	607704	607704	69903.58	69903.6	0.89743	11	3989
VatanenT_2016__G78739__bin.3	0.43	0.0242	5341086	69	489607	489607	77407.04	77407	0.89349	11	4501
363192597_S88_L001	0.43	0.0382	5246263	70	654517	654517	74946.61	74946.6	0.89703	11	4425
SRR9206396	0.43	0.065	5170793	70	889061	889061	73868.47	73868.5	0.89185	11	4362

SRR15283697	0.43	0.0437	5438293	70	581471	581471	77689.9	77689.9	0.89145	11	4694
MCT420_35_S43_L002	0.43	0.0441	5238425	70	704996	704996	74834.64	74834.6	0.89535	11	4474
GCA_003472195.1	0.43	0.0509	5491309	70	967165	763781	119376.3	78440.8	0.8914	11	4653
GCA_022714305.1	0.43	0.0433	5062037	70	431601	431601	72314.81	72314.8	0.89752	11	4227
BackhedF_2015__SID7_M__bin.18	0.43	0.0226	5243679	70	368535	368535	74909.7	74909.7	0.89398	11	4477
10317.X00179306_29	0.43	0.0264	4899367	70	441499	441499	69990.96	69991	0.89275	11	4056
10317.X00214299_94	0.43	0.0292	4844824	70	341832	341832	69211.77	69211.8	0.89461	11	4030
SRR12280658	0.43	0.0359	5310832	71	590198	590198	74800.45	74800.5	0.89395	11	4455
363193046_S156_L001	0.43	0.081	5255432	71	1294217	1294217	74020.17	74020.2	0.89303	11	4431
GCA_015669295.1	0.44	0.044	5161885	71	661670	661670	73741.21	72701.2	0.89437	11	4312
BackhedF_2015__SID587_12M__bin.17	0.43	0.0271	5078429	71	291132	291132	71527.17	71527.2	0.89377	11	4254
YuJ_2015__SZAXPI003422-11__bin.33	0.43	0.028	5141801	71	450530	450530	72419.73	72419.7	0.8947	11	4274
SRR13269223	0.43	0.0304	5387284	72	651530	651530	74823.39	74823.4	0.89314	11	4570
GCA_000598745.1	0.43	0.0432	5273785	72	726158	726158	73247.01	73247	0.88936	11	4379
KarlssonFH_2013__S248__bin.36	0.43	0.0239	5353201	72	387688	387688	74350.01	74350	0.8926	11	4551
GCA_018369215.1	0.43	0.0479	5178168	72	463631	463631	71919	71919	0.89507	11	4340
SRR9206107	0.43	0.0418	5295942	73	526602	526602	72547.15	72547.2	0.89279	11	4493
GCA_000599185.2	0.44	0.0354	5407695	73	337055	337055	74078.01	74078	0.89289	11	4532
GCA_015548495.1	0.43	0.0501	5102436	73	648979	447475	86481.97	69878.9	0.89253	11	4238
10317.X00214153_64	0.43	0.0318	4676587	73	370989	370989	64062.84	64062.8	0.89505	11	3877
SRR12280316	0.43	0.055	5131930	74	501052	501052	69350.41	69350.4	0.89367	11	4317
363193757_S8_L001	0.43	0.0565	5307828	74	628752	628752	71727.41	71727.4	0.89392	11	4479
GCA_008086695.1	0.43	0.0443	5158698	74	1260093	490379	125821.9	69665.2	0.89032	11	4246
10317.X00178995_62	0.43	0.0234	4743343	74	284474	284474	64099.23	64099.2	0.89609	11	3899
HeQ_2017__SZAXPI029475-44__bin.35	0.43	0.0217	4898773	74	499752	499752	66199.64	66199.6	0.89565	11	4054
ChengpingW_2017__AS5raw__bin.14	0.43	0.0215	4996460	74	442614	442614	67519.73	67519.7	0.89466	11	4099
SRR13269152	0.43	0.0363	5366261	75	386253	386253	71550.15	71550.1	0.8932	11	4528
SRR12280648	0.43	0.0434	5195582	75	665830	665830	69274.43	69274.4	0.89355	11	4344
GCA_003363115.1	0.43	0.0555	5252832	75	525726	525725	70037.76	70037.2	0.89394	11	4487
10317.X00178992_47	0.43	0.0289	4871097	75	299702	299702	64947.96	64948	0.89574	11	4102
GCA_015558985.1	0.43	0.0465	5278842	75	385360	385360	78788.69	70369.6	0.8927	11	4453
GCA_015831765.1	0.43	0.0424	4943232	75	678395	678395	68656	65905.1	0.89474	11	4094
BritoIL_2016__M1.10.ST__bin.49	0.43	0.0413	5105544	75	623215	623215	68073.92	68073.9	0.89503	11	4214
SRR15283850	0.43	0.0504	5903127	76	573075	573075	77672.72	77672.7	0.89095	11	5184
GCA_000598785.2	0.44	0.0381	5317743	76	609336	609336	69970.3	69970.3	0.89329	11	4430
10317.X00178933_38	0.43	0.03	5157409	76	355866	355866	67860.64	67860.6	0.89555	11	4369
SRR9206392	0.43	0.0747	5382751	77	769510	769510	69905.86	69905.9	0.8898	11	4503
SRR13269150	0.43	0.0378	5260058	77	541431	541431	68312.44	68312.4	0.89507	11	4383
SRR12280498	0.43	0.0424	5236030	77	1149236	1149236	68000.39	68000.4	0.89191	11	4455
SRR15283695	0.44	0.0379	5374747	77	651823	651823	69801.91	69801.9	0.89277	11	4601
10317.X00179156_10	0.43	0.0272	4854921	77	262491	262491	63050.92	63050.9	0.89635	11	4038
GCA_022722555.1	0.43	0.042	5067696	77	284510	284510	65814.23	65814.2	0.89633	11	4219
10317.X00214694_67	0.43	0.031	4814441	77	231096	231096	62525.21	62525.2	0.89424	11	4028
VatanenT_2016__G80499__bin.3	0.43	0.0216	5031948	77	513260	513260	65349.97	65350	0.89402	11	4204
GCA_013867505.1	0.43	0.035	5219927	78	350801	350801	67791.26	66920.9	0.89429	11	4404

GCA_015553295.1	0.44	0.0395	5359295	78	442999	398192	83738.98	68691.9	0.89397	11	4540
10317.X00180334_10317.X00180334_bin.001	0.43	0.0979	5034211	78	568477	568477	64541.17	64541.2	0.89517	11	4184
BackhedF_2015__SID150_M__bin.47	0.43	0.024	4766181	78	238717	238717	61104.88	61104.9	0.89694	11	3901
BackhedF_2015__SID274_4M__bin.2	0.43	0.0226	5393856	78	299803	299803	69152	69152	0.89436	11	4608
SRR12280649	0.43	0.0528	5376890	79	415058	415058	68061.9	68061.9	0.89262	11	4567
363193029_S139_L001	0.43	0.0382	5084171	79	759136	759136	64356.59	64356.6	0.89583	11	4230
GCA_000598685.2	0.43	0.0409	5535792	79	821616	821616	70073.32	70073.3	0.88876	11	4757
GCA_013327835.1	0.43	0.0519	5404901	79	656032	656032	68416.47	68416.5	0.88871	11	4729
GCA_022733095.1	0.43	0.0422	4924024	79	656436	656436	62329.42	62329.4	0.89788	11	4090
GCA_022735135.1	0.43	0.0422	4735970	79	340261	340261	59948.99	59949	0.89661	11	3908
GCA_000599045.1	0.43	0.0411	5249251	80	700185	700185	65615.64	65615.6	0.8867	11	4440
GCA_008569345.1	0.43	0.0812	5427422	80	872877	872876	67842.78	67842.8	0.8883	11	4554
GCA_021202455.1	0.43	0.0272	4953792	80	381322	381322	64334.96	61918.7	0.89703	11	4098
GCA_022716295.1	0.43	0.0356	4956441	80	367541	367541	61955.51	61955.5	0.89522	11	4072
10317.X00179564_10317.X00179564_bin.11	0.43	0.0337	4814072	80	497049	497049	60175.9	60175.9	0.89671	11	4030
10317.X00179736_10317.X00179736_bin.20	0.43	0.0285	4913712	80	358026	358026	61421.4	61421.4	0.8964	11	4119
10317.X00214812_39	0.43	0.0249	4744877	80	291039	291039	59310.96	59311	0.8921	11	3942
QinN_2014__LD-26__bin.50	0.43	0.021	4922092	80	312464	312464	61526.15	61526.2	0.89825	11	4033
YuJ_2015__SZAXPI015250-39__bin.22	0.43	0.0211	4862442	80	377137	377137	60780.53	60780.5	0.89831	11	4027
GCA_001692695.1	0.43	0.0402	5247065	81	436986	436986	64778.58	64778.6	0.89318	11	4396
GCA_007896605.1	0.43	0.0598	5601214	81	824632	824632	69150.79	69150.8	0.88999	11	4813
VatanenT_2016__G78619__bin.7	0.43	0.0204	4825390	81	254298	254298	59572.72	59572.7	0.8952	11	3971
VatanenT_2016__G78795__bin.5	0.43	0.0454	4984721	81	324976	324976	61539.77	61539.8	0.89577	11	4094
WenC_2017__N281__bin.22	0.43	0.0218	4994801	81	305459	305459	61664.21	61664.2	0.89429	11	4180
363193039_S149_L001	0.43	0.0541	5385178	82	626840	626840	65672.9	65672.9	0.89127	11	4609
GCA_020558655.1	0.43	0.0534	5335813	82	608625	608625	65070.89	65070.9	0.89497	11	4508
10317.X00185745_38	0.43	0.0277	4629645	82	336251	336251	56459.09	56459.1	0.8965	11	3784
SRR13269147	0.43	0.0395	5413497	83	435031	435031	65222.86	65222.9	0.89326	11	4588
SRR15283752	0.43	0.0367	5067274	83	420933	420933	61051.49	61051.5	0.89408	11	4237
GCA_000599365.1	0.43	0.0422	4971985	83	511517	511517	59903.43	59903.4	0.89091	11	4075
GCA_007896575.1	0.43	0.0592	5601691	83	824632	824632	67490.25	67490.3	0.88993	11	4820
GCA_022725225.1	0.43	0.037	4893906	83	454759	454759	58962.72	58962.7	0.89735	11	4017
QinJ_2012__NLM028__bin.25	0.43	0.0186	5132860	83	372394	372394	61841.69	61841.7	0.89227	11	4281
VatanenT_2016__G78910__bin.4	0.44	0.0201	5112631	83	381755	381755	61597.96	61598	0.89667	11	4346
363192563_S54_L001	0.43	0.0438	4999799	84	565423	565423	59521.42	59521.4	0.89459	11	4124
363192795_S100_L001	0.43	0.0489	5348405	84	654437	654437	63671.49	63671.5	0.89126	11	4575
GCA_000599245.1	0.43	0.059	5246809	84	480014	480014	62462.01	62462	0.89281	11	4444
GCA_003469305.1	0.43	0.0376	5065519	84	633690	365731	95575.83	60297.3	0.89313	11	4209
NielsenHB_2014__V1_UC15_3__bin.64	0.43	0.0295	4659306	84	294585	294585	55467.93	55467.9	0.89491	11	3860
SRR13269161	0.43	0.046	5361250	85	509753	509753	63073.53	63073.5	0.89263	11	4567
SRR12280493	0.43	0.0487	5504028	85	728876	728876	64753.27	64753.3	0.8898	11	4767

GCA_008829725.1	0.43	0.0462	5274446	85	434791	434791	62052.31	62052.3	0.89286	11	4471
SRR15283822	0.43	0.0435	5440497	86	549459	549459	63261.59	63261.6	0.89145	11	4670
GCA_000601095.1	0.43	0.0379	5602130	86	798744	798744	65141.05	65141	0.88805	11	4815
GCA_000598985.1	0.43	0.043	5201070	87	897750	897750	59782.41	59782.4	0.89214	11	4308
10317.X00214026_19	0.43	0.0362	4625007	87	352967	352967	53161	53161	0.89519	11	3795
SRR12280645	0.43	0.0565	5093573	88	893897	893897	57881.51	57881.5	0.89279	11	4337
SchirmerM_2016__G88708__bin.4	0.43	0.0275	4930155	88	213219	213219	56024.49	56024.5	0.89581	11	4089
HeQ_2017__SZAXPI029574-89__bin.37	0.43	0.021	4855843	89	368214	368214	54560.03	54560	0.89904	11	4010
GCA_022744835.1	0.43	0.0403	5021901	89	306019	306019	56425.85	56425.9	0.89603	11	4155
SRR15283724	0.44	0.0586	5612317	90	437955	437955	62359.08	62359.1	0.89215	11	4897
SRR15283828	0.44	0.05	5540994	90	336613	336613	61566.6	61566.6	0.89286	11	4764
SRR12280608	0.44	0.0495	5258627	90	566233	566233	58429.19	58429.2	0.89295	11	4495
MCT420_28_S36_L002	0.43	0.0428	5012326	90	495004	495004	55692.51	55692.5	0.89682	11	4203
GCA_020026255.1	0.43	0.0187	4749691	90	245948	245948	52774.34	52774.3	0.89892	11	3934
SRR13269164	0.43	0.0509	5146685	91	407847	407847	56556.98	56557	0.8937	11	4336
QinJ_2012__DLF009__bin.9	0.43	0.0243	5204374	91	652826	652826	57190.92	57190.9	0.89502	11	4419
MCT420_29_S37_L002	0.44	0.0373	5220606	92	289323	289323	56745.72	56745.7	0.89438	11	4324
GCA_014042405.1	0.43	0.0288	5321320	92	519253	519253	57840.43	57840.4	0.89458	11	4545
GCA_000598565.1	0.43	0.0391	5117152	93	507975	507975	55023.14	55023.1	0.88888	11	4295
10317.X00179317_4	0.43	0.0266	4711404	93	236119	236119	50660.26	50660.3	0.89565	11	3896
10317.X00180216_72	0.43	0.0317	4688365	93	216848	216848	50412.53	50412.5	0.89617	11	3880
GCA_900624785.1	0.43	0.0521	5447734	93	377070	377070	58577.78	58577.8	0.89253	11	4684
VatanenT_2016__G78527__bin.3	0.43	0.0372	5167901	93	309262	309262	55568.83	55568.8	0.89313	11	4297
SRR15283706	0.43	0.0327	5216273	94	460976	460976	55492.27	55492.3	0.89201	11	4466
GCA_000599165.2	0.43	0.0352	5238350	94	424528	424528	55727.13	55727.1	0.89048	11	4410
GCA_003439505.1	0.43	0.0684	4987072	94	1018640	1018640	81755.28	53028.5	0.89257	11	4130
10317.X00179274_24	0.43	0.0288	4763451	94	262206	262206	50675.01	50675	0.89504	11	3960
NielsenHB_2014__V1_CD21_4__bin.5	0.43	0.0324	5102948	94	359712	359712	54286.68	54286.7	0.88983	11	4285
SRR13269219	0.43	0.0483	5399177	95	491410	491410	56833.44	56833.4	0.89323	11	4654
GCA_022745625.1	0.43	0.0379	5007809	95	280302	280302	52713.78	52713.8	0.89624	11	4126
BackhedF_2015__SID229_B__bin.3	0.43	0.0256	4951257	95	264688	264688	52118.49	52118.5	0.8929	11	4186
GCA_000218345.1	0.44	0.0713	5648441	96	2314291	661282	120179.6	58669.9	0.8836	11	4888
GCA_022734965.1	0.43	0.0363	4884286	96	365532	365532	50877.98	50878	0.8968	11	4064
10317.X00215241_67	0.43	0.0298	4711440	96	183497	183497	49077.5	49077.5	0.89601	11	3848
SRR9206393	0.43	0.0463	5288000	97	667533	667533	54515.46	54515.5	0.89286	11	4517
SRR12280336	0.43	0.0474	5152330	98	546782	546782	52574.8	52574.8	0.89409	11	4345
GCA_015560515.1	0.43	0.0408	5626867	98	369567	369567	63223.22	57401.9	0.88932	11	4884
GCA_015831745.1	0.43	0.0427	4942533	98	622851	622851	52026.66	50430.3	0.89548	11	4114
GCA_022747335.1	0.43	0.0446	4934366	99	441360	441360	49842.08	49842.1	0.895	11	4041
VatanenT_2016__G80601__bin.2	0.44	0.0241	5096302	99	237003	237003	51477.8	51477.8	0.89517	11	4281
363192533_S31_L001	0.43	0.0466	5221787	100	814351	814351	52217.87	52217.9	0.89495	11	4376
GCA_000599385.1	0.43	0.0464	5225713	100	716595	716595	52257.13	52257.1	0.89209	11	4459
GCA_009024655.1	0.43	0.0587	5614970	100	824632	824632	56149.7	56149.7	0.88925	11	4833
GCA_022733395.1	0.43	0.0436	4952285	100	306006	306006	49522.85	49522.9	0.89513	11	4123
10317.X00185696_11	0.43	0.0285	4653865	100	233693	233693	46538.65	46538.7	0.89799	11	3798
SRR12280643	0.43	0.0495	5303722	101	515182	515182	52512.1	52512.1	0.89239	11	4483

GCA_003468145.1	0.43	0.0528	5165672	101	520858	520858	73795.31	51142.1	0.89512	11	4319
SRR12280561	0.43	0.0434	5077181	102	592581	592581	49776.28	49776.3	0.8924	11	4294
GCA_000598925.1	0.43	0.0387	5204756	102	622654	622654	51027.02	51027	0.88974	11	4378
VatanenT_2016__G80532__bin.12	0.43	0.0356	4967534	102	232075	232075	48701.31	48701.3	0.89517	11	4165
VatanenT_2016__G80313__bin.13	0.43	0.0264	4994479	103	259129	259129	48490.09	48490.1	0.89602	11	4136
363193043_S153_L001	0.43	0.0448	5310802	104	761147	761147	51065.4	51065.4	0.89221	11	4489
363193001_S108_L001	0.43	0.0782	5351331	104	982885	982885	51455.11	51455.1	0.89297	11	4546
MCT420_47_S55_L002	0.44	0.0417	5355315	104	619939	619939	51493.41	51493.4	0.89448	11	4557
SRR13269222	0.43	0.0478	5538432	104	529673	529673	53254.15	53254.2	0.89157	11	4732
XieH_2016__YSZC12003_37205__bin.26	0.43	0.0201	4816556	104	222722	222722	46313.04	46313	0.89777	11	3994
MCT420_8_S16_L002	0.43	0.0296	5221702	105	365941	365941	49730.5	49730.5	0.89349	11	4410
GCA_000599025.1	0.44	0.0529	5743609	105	388901	388901	54701.04	54701	0.89104	11	5076
MCT420_7_S15_L002	0.43	0.0473	5405542	106	585008	585008	50995.68	50995.7	0.89117	11	4568
10317.X00179043_48	0.43	0.0299	4706466	106	259446	259446	44400.62	44400.6	0.8956	11	3876
MCT420_52_S60_L002	0.43	0.0634	5384008	107	519684	519684	50317.83	50317.8	0.8937	11	4592
GCA_022745295.1	0.43	0.0351	4841187	107	385422	385422	45244.74	45244.7	0.89724	11	4028
SRR13269159	0.43	0.0473	5210495	108	618340	618340	48245.32	48245.3	0.89268	11	4384
363192529_S12_L001	0.44	0.0381	5373160	108	461524	461524	49751.48	49751.5	0.89426	11	4578
10317.X00214161_10317.X00214161_bin.4	0.44	0.0325	4505665	108	280059	280059	41719.12	41719.1	0.89632	11	3710
SRR15283685	0.43	0.044	5510347	109	400617	400617	50553.64	50553.6	0.88839	11	4715
NielsenHB_2014__O2_UC34_2__bin.41	0.43	0.0276	4695318	109	308338	308338	43076.31	43076.3	0.898	11	3833
10317.X00215207_10317.X00215207_bin.26	0.44	0.026	4496160	110	170718	170718	40874.18	40874.2	0.89582	11	3741
GCA_022720585.1	0.43	0.0445	5093464	111	237452	237452	45887.06	45887.1	0.89518	11	4244
VatanenT_2016__G80611__bin.2	0.44	0.0258	4782060	111	364525	364525	43081.62	43081.6	0.89585	11	3966
363193756_S6_L001	0.44	0.0444	5365521	112	787081	787081	47906.44	47906.4	0.89496	11	4640
GCA_022741265.1	0.43	0.0377	4898013	112	366995	366995	43732.26	43732.3	0.89689	11	4039
SRR13269157	0.43	0.054	5379806	113	389612	389612	47608.9	47608.9	0.88989	11	4608
SRR12280572	0.43	0.0488	5312271	113	316351	316351	47011.25	47011.2	0.8944	11	4538
GCA_002810995.1	0.43	0.0551	5221750	113	1080273	364585	96699.07	46182.3	0.89002	11	4372
GCA_022746405.1	0.43	0.0441	5034911	113	303038	303038	44556.73	44556.7	0.89531	11	4231
10317.X00185597_21_sub	0.43	0.0296	4660130	113	202396	202396	41240.09	41240.1	0.89297	11	3833
BackhedF_2015__SID137_M__bin.72	0.43	0.0316	5165461	113	361267	361267	45712.04	45712	0.89425	11	4333
SRR12280315	0.44	0.0459	5621064	114	384367	384367	49307.58	49307.6	0.88987	11	4855
SRR12280651	0.43	0.0456	5406406	114	553856	553856	47424.61	47424.6	0.89116	11	4615
GCA_022727875.1	0.43	0.0442	4963616	114	395653	395653	43540.49	43540.5	0.89796	11	4095
10317.X00185597_21	0.43	0.0299	4662858	114	202396	202396	40902.26	40902.3	0.89292	11	3837
GCA_022733815.1	0.43	0.036	4905164	115	369570	369570	42653.6	42653.6	0.89672	11	4081
10317.X00179725_20	0.43	0.0268	4581992	115	293680	293680	39843.41	39843.4	0.89626	11	3791
GCA_022721415.1	0.43	0.0377	4831478	116	218163	218163	41650.67	41650.7	0.89675	11	3995
GCA_000598845.1	0.43	0.0445	5245216	117	349293	349293	44830.91	44830.9	0.88916	11	4411
SRR12280589	0.43	0.0458	5368649	118	410494	410494	45497.03	45497	0.8914	11	4571
HMP_2012__SRS023346__bin.6	0.43	0.0285	4756349	118	201858	201858	40308.04	40308	0.89636	11	3983
GCA_022746755.1	0.43	0.0327	5035663	118	231702	231702	42675.11	42675.1	0.89461	11	4156
SRR15283738	0.44	0.0481	5663294	119	363366	363366	47590.71	47590.7	0.89129	11	4971

GCA_000599225.1	0.43	0.0472	5307885	119	352500	352500	44604.08	44604.1	0.89219	11	4545
10317.X00179298_6	0.43	0.0293	4761917	119	204892	204892	40016.11	40016.1	0.8968	11	3962
KosticAD_2015__PRJNA231909.3101439__bin.2	0.43	0.047	4931656	119	215183	215183	41442.49	41442.5	0.8937	11	4134
GCA_022746345.1	0.43	0.0418	4848732	119	292893	292893	40745.65	40745.6	0.89748	11	3956
GCA_000598645.1	0.43	0.0551	5144743	120	620440	620440	42872.86	42872.9	0.8883	11	4264
GCA_002810985.1	0.43	0.0367	5208842	121	345402	319589	60567.93	43029.2	0.88991	11	4404
GCA_019013355.1	0.43	0.0263	4864580	121	232922	232922	40203.14	40203.1	0.89185	11	4045
GCA_021633405.1	0.43	0.0235	4836769	121	249819	249819	43970.63	39969.4	0.89657	11	3984
GCA_000599145.2	0.43	0.0435	5549782	122	423731	423731	45490.02	45490	0.88947	11	4808
GCA_000599305.1	0.43	0.0476	5322122	122	305039	305039	43623.95	43624	0.89122	11	4546
363193002_S105_L001	0.43	0.0377	5318194	123	1006163	1006163	43237.35	43237.3	0.89195	11	4555
GCA_000598245.1	0.43	0.058	5167326	124	380200	380200	41671.98	41672	0.89231	11	4343
GCA_022725025.1	0.43	0.0353	4738145	124	271913	271913	38210.85	38210.8	0.89672	11	3895
SRR12280501	0.43	0.0535	5229821	125	330894	330894	41838.57	41838.6	0.89288	11	4508
GCA_018786025.1	0.44	0.0366	5288074	126	449067	321514	51843.86	41957.9	0.89092	11	4479
SRR13269221	0.43	0.0353	5095672	127	349896	349896	40123.4	40123.4	0.89294	11	4366
GCA_002811035.1	0.43	0.0366	5224159	127	447641	282178	62192.37	41126.6	0.89261	11	4430
LiSS_2016__FAT_015-22-42-0__bin.55	0.43	0.0249	4849069	127	182248	182248	38181.65	38181.6	0.89445	11	4022
GCA_020558865.1	0.44	0.0583	5764972	127	501049	501049	45393.48	45393.5	0.89269	11	5225
GCA_013694025.1	0.44	0.0345	4920846	128	442497	442497	38444.11	38444.1	0.89375	11	4140
10317.X00185925_50	0.43	0.0309	4796270	128	354246	354246	37470.86	37470.9	0.89558	11	4030
VatanenT_2016__G78715__bin.6	0.43	0.0378	4922170	129	185669	185669	38156.36	38156.4	0.89558	11	4054
10317.X00214119_32	0.44	0.0372	4500330	130	172402	172402	34617.92	34617.9	0.89427	11	3761
10317.X00214152_5	0.44	0.0345	4462790	130	203259	203259	34329.15	34329.2	0.89532	11	3703
SRR13269209	0.43	0.0649	5566499	132	467127	467127	42170.45	42170.4	0.89133	11	4815
MCT420_77_S85_L002	0.43	0.0374	5205580	133	318403	318403	39139.7	39139.7	0.89343	11	4450
GCA_000601055.1	0.43	0.0507	5333423	133	444802	444802	40100.92	40100.9	0.89049	11	4550
GCA_000601075.2	0.43	0.0511	5214644	133	559073	559073	39207.85	39207.8	0.89018	11	4461
YuJ_2015__SZAXPI015216-169__bin.42	0.43	0.0234	5774429	133	445221	445221	43416.76	43416.8	0.89555	11	4778
GCA_001054865.1	0.44	0.0418	5496361	135	274582	274582	40713.79	40713.8	0.8922	11	4652
GCA_002811025.1	0.43	0.0327	5144622	135	572068	559725	57162.47	38094.1	0.89017	11	4281
SRR13269178	0.43	0.0444	5194059	136	367559	367559	38191.61	38191.6	0.8913	11	4425
GCA_019041735.1	0.43	0.0476	5453915	138	456282	456282	39521.12	39521.1	0.88967	11	4731
GCA_003463555.1	0.43	0.0603	5044825	139	247803	247803	36293.71	36293.7	0.88797	11	4714
GCA_001056335.1	0.44	0.043	5487974	140	307578	307578	39481.83	39199.1	0.89157	11	4651
GCA_001699865.1	0.43	0.0503	5210816	140	585236	585236	37220.11	37220.1	0.88871	11	4380
GCA_001816225.2	0.43	0.0499	5354648	141	472857	472857	40565.52	37971	0.89233	11	4529
VatanenT_2016__G80458__bin.3	0.43	0.0326	4886995	141	208051	208051	34659.54	34659.5	0.89536	11	4069
GCA_003458215.1	0.43	0.0811	5420590	143	741298	467418	60905.51	37901.7	0.89063	11	4613
GCA_019041775.1	0.43	0.0503	5455891	143	456282	456282	38153.08	38153.1	0.88978	11	4736
GCA_019130425.1	0.44	0.0544	5193175	144	431792	431792	36063.72	36063.7	0.89519	11	4424
GCA_000601035.1	0.43	0.0534	5486951	145	326416	326416	37841.04	37841	0.89174	11	4766
GCA_001580095.1	0.43	0.0484	5206456	145	448317	185220	100124.2	35842.5	0.88805	11	4354
GCA_001580105.1	0.43	0.0484	5206456	145	448317	185220	100124.2	35842.5	0.88805	11	4354
GCA_019130335.1	0.44	0.0541	5193048	145	431792	431792	35814.12	35814.1	0.89532	11	4426
GCA_000599005.1	0.43	0.0619	5239256	147	422931	422931	35641.2	35641.2	0.89051	11	4390

10317.X00180154_10317.X00180154_bin.17	0.43	0.0314	4698672	147	189988	189988	31963.76	31963.8	0.8934	11	3916
363192522_S5_L001	0.44	0.0502	5394365	148	584366	584366	36448.41	36448.4	0.89092	11	4616
GCA_000598905.1	0.43	0.0442	5282071	148	426431	426431	35689.67	35689.7	0.88801	11	4489
HMP_2012__SRS075398__bin.15	0.43	0.0302	4966477	150	177622	177622	33109.85	33109.8	0.89654	11	4108
VatanenT_2016__G78592__bin.3	0.43	0.0331	4927522	150	243892	243892	32850.15	32850.1	0.89285	11	4178
VincentC_2016__MM060.2__bin.1	0.43	0.0354	4991432	150	223007	223007	33276.21	33276.2	0.89471	11	4286
XieH_2016__YSZC12003_37880__bin.18	0.43	0.0292	5167529	150	235055	235055	34450.19	34450.2	0.89534	11	4297
SRR12280416	0.43	0.0451	5359356	153	638897	638897	35028.47	35028.5	0.89544	11	4580
VogtmannE_2016__MMRS72980899ST-27-0-0__bin.27	0.43	0.0296	5603209	154	327844	327844	36384.47	36384.5	0.89218	11	4768
LeChatelierE_2013__MH0410__bin.30	0.43	0.0314	5032496	155	152319	152319	32467.72	32467.7	0.89638	11	4219
GCA_022832325.1	0.44	0.0579	5302663	155	960591	960591	34210.73	34210.7	0.89331	11	4490
GCA_000598545.2	0.43	0.0506	5409011	156	441791	441791	34673.15	34673.1	0.8899	11	4699
GCA_001699885.1	0.43	0.0422	5258210	156	350802	350802	33706.47	33706.5	0.89184	11	4490
GCA_019041875.1	0.43	0.0492	5451927	156	456282	456282	34948.25	34948.3	0.88976	11	4746
10317.X00214162_22	0.44	0.0421	4507763	156	125878	125878	28895.92	28895.9	0.89392	11	3699
GCA_002811085.1	0.43	0.0251	5080019	157	666618	483297	44174.08	32345.5	0.88918	11	4190
GCA_022731325.1	0.43	0.042	4732284	157	197318	197318	30141.94	30141.9	0.89895	11	3894
GCA_002811065.1	0.43	0.0418	5419893	158	349209	259041	58911.88	34290.1	0.88981	11	4622
SRR12280496	0.43	0.0394	5346531	159	383793	383793	33625.98	33626	0.8917	11	4640
VatanenT_2016__G80290__bin.3	0.43	0.0335	5036137	161	164710	164710	31280.35	31280.4	0.89547	11	4181
363192998_S106_L001	0.44	0.054	5620137	162	620751	620751	34692.2	34692.2	0.89153	11	4944
SRR13269204	0.43	0.0535	5253949	162	478742	478742	32431.78	32431.8	0.88967	11	4484
KosticAD_2015__PRJNA231909.3107649__bin.14	0.43	0.0378	4917800	163	215420	215420	30170.55	30170.6	0.89444	11	4153
10317.X00179407_24	0.43	0.0323	4643336	163	143919	143919	28486.72	28486.7	0.89446	11	3917
10317.X00179842_5	0.43	0.0328	4479936	166	247782	247782	26987.57	26987.6	0.89554	11	3703
SRR13269149	0.44	0.0424	5292031	168	987782	987782	31500.18	31500.2	0.8924	11	4574
ChengpingW_2017__AS18raw__bin.15	0.43	0.0233	4802615	168	175980	175980	28586.99	28587	0.89776	11	3982
VatanenT_2016__G80406__bin.2	0.44	0.0282	4653750	168	170003	170003	27700.89	27700.9	0.89768	11	3839
SRR12280650	0.43	0.0566	5228983	169	226125	226125	30940.73	30940.7	0.89201	11	4553
GCA_022742265.1	0.43	0.0373	4879329	169	189513	189513	28871.77	28871.8	0.89702	11	4084
VatanenT_2016__G78890__bin.1	0.43	0.0333	4864475	170	166364	166364	28614.56	28614.6	0.89841	11	4041
GCA_022734285.1	0.43	0.0335	4422691	171	144186	144186	25863.69	25863.7	0.89982	11	3599
10317.X00179278_57	0.43	0.0317	4605520	172	208068	208068	26776.28	26776.3	0.89553	11	3860
SRR12280499	0.43	0.0473	5573286	173	620027	620027	32215.53	32215.5	0.89352	11	4827
BackhedF_2015__SID172_12M__bin.1	0.43	0.0373	5259543	173	190642	190642	30401.98	30402	0.89529	11	4402
GCA_015669435.1	0.43	0.0584	5386214	177	570700	542833	31683.61	30426.7	0.89453	11	4591
NielsenHB_2014__V1_CD6_4__bin.58	0.43	0.0298	5386058	178	210321	210321	30258.75	30258.8	0.89491	11	4547
10317.X00179680_33	0.43	0.0291	4534299	180	91002	91002	25190.55	25190.6	0.89369	11	3754

MCT420_2_S10_L002	0.43	0.0361	5228138	183	247394	247394	28569.06	28569.1	0.89487	11	4499
SRR12280492	0.43	0.0427	5096432	185	204716	204716	27548.28	27548.3	0.89237	11	4475
10317.X00179047_10317.X00179047_bin.7	0.43	0.0321	4818253	186	138546	138546	25904.59	25904.6	0.89606	11	4095
VatanenT_2016__G80297__bin.3	0.43	0.0349	4931149	187	92332	92332	26369.78	26369.8	0.89594	11	4166
363193037_S147_L001	0.44	0.0517	5589447	191	402143	402143	29264.12	29264.1	0.89391	11	4832
VatanenT_2016__G80399__bin.4	0.44	0.0258	4662039	191	179645	179645	24408.58	24408.6	0.8961	11	3875
GCA_022737795.1	0.43	0.0395	4953393	192	164741	164741	25798.92	25798.9	0.89592	11	4129
GCA_001054895.1	0.44	0.0434	5450450	193	229066	229066	28240.67	28240.6	0.89204	11	4629
SRR12280642	0.44	0.0443	5314484	194	750273	750273	27394.25	27394.2	0.89281	11	4611
10317.X00180266_69	0.44	0.0308	4530305	196	142711	142711	23113.8	23113.8	0.89408	11	3768
SRR13269167	0.43	0.0526	5472199	197	278096	278096	27777.66	27777.7	0.8929	11	4798
HeQ_2017__RSZAXPI003098-132__bin.12	0.43	0.0268	4680510	197	146903	146903	23758.93	23758.9	0.89764	11	3985
10317.X00180463_38	0.43	0.0315	4493442	200	149213	149213	22467.21	22467.2	0.89585	11	3752
GCA_000598865.1	0.43	0.0545	5300459	201	416644	416644	26370.44	26370.4	0.88995	11	4550
LeChatelierE_2013__MH0068__bin.12	0.43	0.0331	4699576	201	137018	137018	23380.98	23381	0.89583	11	3979
LiuW_2016__SRR3993030__bin.36	0.43	0.0263	5064995	202	166817	166817	25074.23	25074.2	0.8925	11	4218
VatanenT_2016__G78826__bin.7	0.44	0.0272	4850145	202	106018	106018	24010.62	24010.6	0.8981	11	4068
GCA_000598825.1	0.43	0.0647	5572666	203	366183	366183	27451.56	27451.6	0.88932	11	4799
363192999_S107_L001	0.43	0.0507	5351696	204	494868	494868	26233.8	26233.8	0.89284	11	4562
363192793_S98_L001	0.43	0.0584	5511565	205	349050	349050	26885.68	26885.7	0.89278	11	4776
VatanenT_2016__G80299__bin.1	0.43	0.0428	5001175	205	154892	154892	24395.98	24396	0.89323	11	4182
10317.X00214239_55	0.44	0.0308	4344976	209	94955	94955	20789.36	20789.4	0.89363	11	3598
10317.X00179467_5	0.43	0.0305	4736945	210	126336	126336	22556.88	22556.9	0.89326	11	4056
SRR13269148	0.43	0.0443	5094109	217	171640	171640	23475.16	23475.2	0.89125	11	4436
10317.X00214333_11	0.44	0.0366	4280179	225	121165	121165	19023.02	19023	0.89475	11	3595
363192797_S102_L001	0.43	0.0608	6106417	226	228130	228130	27019.54	27019.5	0.89396	11	5408
VatanenT_2016__G78669__bin.1	0.43	0.041	4890422	227	163208	163208	21543.71	21543.7	0.89533	11	4060
10317.X00180095_68	0.43	0.0312	4469419	230	275504	275504	19432.26	19432.3	0.89633	11	3742
ZeeviD_2015__PNP_Validation_92__bin.15	0.43	0.0403	4946967	233	122690	122690	21231.62	21231.6	0.89743	11	4151
MCT420_80_S88_L002	0.43	0.0432	5371525	235	254643	254643	22857.55	22857.6	0.89194	11	4690
GCA_003473955.1	0.44	0.0537	5472193	238	230846	230846	23089.42	22991.8	0.88459	11	5348
GCA_000598285.1	0.43	0.0492	5770122	239	251995	251995	24142.77	24142.8	0.8889	11	4965
10317.X00179186_39	0.43	0.0311	4711304	239	117924	117924	19712.57	19712.6	0.89419	11	4037
GCA_000598525.1	0.43	0.0528	5509867	241	237380	237380	22862.52	22862.5	0.88549	11	4822
GCA_020539125.1	0.44	0.0451	5433156	242	524665	524665	22451.06	22451.1	0.8943	11	4750
SRR13269162	0.43	0.0474	5278406	244	131738	131738	21632.81	21632.8	0.89298	11	4714
363192560_S51_L001	0.43	0.0591	5753866	246	420053	420053	23389.7	23389.7	0.89519	11	4997
GCA_003475745.1	0.44	0.0519	5040582	249	221859	221859	20243.3	20243.3	0.88254	11	4865
GCA_901212445.1	0.43	0.0532	5045202	258	280019	280019	28028.9	19548.2	0.8952	11	4219
GCA_004798525.1	0.43	0.0632	5621897	261	1044743	1044743	21539.84	21539.8	0.88789	11	5453
VatanenT_2016__G80388__bin.7	0.44	0.0294	4746694	262	97438	97438	18117.15	18117.2	0.89732	11	3973
GCA_000598665.1	0.43	0.0505	5261742	263	233552	233552	20006.62	20006.6	0.88859	11	4609
GCA_020538785.1	0.44	0.061	5316117	264	236953	236953	20136.81	20136.8	0.8959	11	4829

GCA_000598505.1	0.44	0.0601	5051299	271	192775	192775	18639.48	18639.5	0.88975	11	4340
VatanenT_2016__G78676__bin.4	0.44	0.031	5165044	273	126256	126256	18919.58	18919.6	0.89359	11	4354
GCA_022719775.1	0.44	0.0413	4556361	274	104075	104075	16629.05	16629.1	0.8991	11	3772
GCA_000598225.1	0.43	0.0411	5644786	275	217537	217537	20526.49	20526.5	0.88794	11	5012
GCA_000599125.1	0.44	0.0603	5717113	278	260344	260344	20565.15	20565.2	0.89075	11	5120
MCT420_86_S94_L002	0.43	0.0425	5478153	279	119519	119519	19634.96	19635	0.89213	11	4859
363192541_S20_L001	0.43	0.0712	6105470	282	309418	309418	21650.6	21650.6	0.89322	11	5446
363192805_S110_L001	0.43	0.0616	6183263	283	405604	405604	21848.99	21849	0.89634	11	5601
363192527_S10_L001	0.43	0.0548	5685650	284	270731	270731	20019.89	20019.9	0.89037	11	4943
GCA_000598265.1	0.43	0.0568	5481195	286	166043	166043	19165.02	19165	0.89105	11	4840
363192555_S46_L001	0.43	0.0628	5913902	294	275110	275110	20115.31	20115.3	0.89354	11	5218
SRR12280583	0.43	0.0596	5492373	302	243241	243241	18186.67	18186.7	0.89214	11	4852
NielsenHB_2014__V1_UC34_0__bin.49	0.43	0.0336	6483326	304	226035	226035	21326.73	21326.7	0.90585	11	5255
GCA_000599085.1	0.43	0.054	5038892	308	231806	231806	16360.04	16360	0.89287	11	4232
NielsenHB_2014__V1_UC15_0__bin.1	0.43	0.0404	5166407	311	102617	102617	16612.24	16612.2	0.89053	11	4541
GCA_000598145.1	0.43	0.0637	5635496	312	345687	345687	18062.49	18062.5	0.88941	11	4951
GCA_000598185.2	0.43	0.0559	5028768	313	231745	231745	16066.35	16066.4	0.89354	11	4280
GCA_000598305.1	0.44	0.0573	5411964	313	136033	136033	17290.62	17290.6	0.89454	11	4720
KosticAD_2015__PRJNA231909.3113035__bin.1	0.43	0.0412	4925055	316	148250	148250	15585.62	15585.6	0.89377	11	4256
LoombaR_2017__SID5362_xgk__bin.16	0.43	0.0393	5826864	319	195181	195181	18266.03	18266	0.89056	11	5089
363193763_S14_L001	0.44	0.0606	5891708	320	431767	431767	18411.59	18411.6	0.89449	11	5346
10317.X00180500_8	0.44	0.0337	4411424	321	77630	77630	13742.75	13742.8	0.89636	11	3761
MCT420_58_S66_L002	0.43	0.0388	5233034	328	130957	130957	15954.37	15954.4	0.88965	11	4837
VatanenT_2016__G69155__bin.3	0.43	0.0379	4889912	328	68656	68656	14908.27	14908.3	0.89592	11	4196
GCA_022738395.1	0.44	0.0405	4637136	332	95752	95752	13967.28	13967.3	0.89865	11	3860
363193013_S123_L001	0.43	0.0584	5998962	333	247763	247763	18014.9	18014.9	0.8921	11	5340
GCA_022716895.1	0.44	0.04	4654615	333	96554	96554	13977.82	13977.8	0.8976	11	3937
GCA_022714225.1	0.44	0.0475	4714878	338	107489	107489	13949.34	13949.3	0.8981	11	3953
10317.X00215215_15	0.44	0.0344	4505691	345	124926	124926	13059.97	13060	0.89649	11	3865
VatanenT_2016__G80518__bin.7	0.43	0.0476	5116769	353	94736	94736	14495.1	14495.1	0.89436	11	4396
VatanenT_2016__G78646__bin.3	0.43	0.0362	4956040	356	63060	63060	13921.46	13921.5	0.89533	11	4322
VincentC_2016__MM032.3__bin.19	0.43	0.0355	5021357	363	131551	131551	13832.94	13832.9	0.89462	11	4397
SRR12280591	0.43	0.0432	5215780	364	103594	103594	14329.07	14329.1	0.89126	11	4603
363192552_S43_L001	0.44	0.0665	5967478	369	223592	223592	16172.03	16172	0.89172	11	5368
GCA_001058775.1	0.44	0.0535	5314495	370	189489	189489	14363.5	14363.5	0.89429	11	4623
VatanenT_2016__G80158__bin.16	0.43	0.0357	4907027	370	65588	65588	13262.24	13262.2	0.89267	11	4207
KosticAD_2015__PRJNA231909.3108874__bin.9	0.42	0.0447	5443290	372	124052	124052	14632.5	14632.5	0.89248	11	4847
GCA_000598385.1	0.44	0.0559	5496616	387	181075	181075	14203.14	14203.1	0.8921	11	4824
363192539_S19_L001	0.44	0.0597	6744606	392	378044	378044	17205.63	17205.6	0.89456	11	6173
363192559_S50_L001	0.44	0.0716	5825100	394	811951	811951	14784.52	14784.5	0.88984	11	5313
SRR13269165	0.43	0.0572	6080204	397	205588	205588	15315.38	15315.4	0.89128	11	5502

BackhedF_2015__SID635_B__bin.2	0.43	0.0411	5165142	397	125716	125716	13010.43	13010.4	0.89383	11	4625
GCA_022718435.1	0.44	0.0419	4527184	404	105391	105391	11205.9	11205.9	0.90048	11	3737
GCA_022742205.1	0.43	0.0388	4845547	404	88871	88871	11993.93	11993.9	0.89835	11	4171
GCA_000598365.1	0.43	0.057	5190460	408	188232	188232	12721.72	12721.7	0.88818	11	4564
GCA_022741515.1	0.44	0.0436	4684184	409	164277	164277	11452.77	11452.8	0.8981	11	3959
VatanenT_2016__G80556__bin.10	0.43	0.0396	4899676	409	55876	55876	11979.65	11979.6	0.8929	11	4379
GCA_000598425.1	0.43	0.0529	5262880	426	130036	130036	12354.18	12354.2	0.89226	11	4594
GCA_022734035.1	0.43	0.038	4650059	426	71655	71655	10915.63	10915.6	0.89614	11	4001
GCA_001058755.1	0.44	0.0524	5314007	435	136154	136154	12216.11	12216.1	0.89354	11	4652
GCA_000598345.1	0.44	0.0508	5185876	436	161827	161827	11894.21	11894.2	0.89239	11	4492
VincentC_2016__MM022.2__bin.34	0.43	0.0318	5142846	442	100384	100384	11635.4	11635.4	0.89488	11	4596
363197065_S315_L001	0.44	0.0622	5477522	447	528412	528412	12253.96	12254	0.89621	11	4854
GCA_000598165.1	0.44	0.0485	5225257	447	259474	259474	11689.61	11689.6	0.89367	11	4515
GCA_000598325.1	0.44	0.0531	5287786	449	182559	182559	11776.81	11776.8	0.89169	11	4575
SRR12280635	0.43	0.0408	4960285	451	64448	64448	10998.41	10998.4	0.89264	11	4514
GCA_022737435.1	0.43	0.0369	4522938	458	92147	92147	9875.41	9875.41	0.89547	11	3960
GCA_000598205.1	0.43	0.064	5349243	462	188077	188077	11578.45	11578.4	0.88728	11	4749
GCA_022741435.1	0.44	0.0446	4794456	479	72665	72665	10009.3	10009.3	0.89971	11	4073
363192544_S35_L001	0.44	0.0623	6313040	503	404411	404411	12550.78	12550.8	0.8924	11	5805
GCA_022719595.1	0.44	0.0428	4590763	517	56872	56872	8896.828	8879.59	0.8979	11	3892
10317.X00185521_58	0.44	0.0354	4316980	533	49830	49830	8099.4	8099.4	0.89568	11	3860
VatanenT_2016__G78605__bin.3	0.43	0.0495	5669093	541	90490	90490	10478.91	10478.9	0.88955	11	5155
GCA_000598805.1	0.44	0.0462	5016071	557	186241	186241	9005.513	9005.51	0.89931	11	4334
GCA_022724695.1	0.44	0.0413	4550909	560	63674	63674	8126.623	8126.62	0.9026	11	3858
GCA_022717255.1	0.43	0.0387	4658008	572	48965	48965	8143.371	8143.37	0.89741	11	4139
GeversD_2014__SKBSTL063__bin.2	0.43	0.039	4796286	678	85873	85873	7074.168	7074.17	0.89821	11	4413
GCA_022744925.1	0.44	0.0409	4680395	686	58795	58795	6822.733	6822.73	0.90213	11	4103
363193004_S114_L001	0.43	0.0494	6210918	706	136333	136333	8797.334	8797.33	0.89154	11	5839
VatanenT_2016__G78604__bin.1	0.44	0.0413	5086942	743	107867	107867	6846.49	6846.49	0.89522	11	4653
GCA_000598445.1	0.44	0.0505	5311676	792	91276	91276	6706.662	6706.66	0.89511	11	4856
GCA_004798445.1	0.43	0.0592	5552690	840	536376	536376	6610.345	6610.35	0.87925	11	5584

TABLE 5

Sample	PS	Path
10317.X00178933_38	PSA	PSA_01
10317.X00178992_47	PSA	PSA_01
10317.X00179017_10317.X00179017_bin.7	PSA	PSA_01
10317.X00179298_6	PSA	PSA_01
10317.X00179381_10317.X00179381_bin.18	PSA	PSA_01
10317.X00179487_25	PSA	PSA_01
10317.X00179673_25	PSA	PSA_01
10317.X00214130_23	PSA	PSA_01
363192044_S216_L001.3	PSA	PSA_01
363192522_S5_L001	PSA	PSA_01
363192544_S35_L001	PSA	PSA_01
363192563_S54_L001	PSA	PSA_01
363192573_S64_L001	PSA	PSA_01
363192581_S72_L001	PSA	PSA_01
363192602_S93_L001	PSA	PSA_01
363192603_S94_L001	PSA	PSA_01
363192605_S96_L001	PSA	PSA_01
363192795_S100_L001	PSA	PSA_01
363192805_S110_L001	PSA	PSA_01
363193001_S108_L001	PSA	PSA_01
363193015_S125_L001.1	PSA	PSA_01
363193022_S130_L001.1	PSA	PSA_01
363193029_S139_L001	PSA	PSA_01
363193031_S141_L001.1	PSA	PSA_01
363193036_S146_L001	PSA	PSA_01
363193048_S163_L001	PSA	PSA_01
363193069_S179_L001	PSA	PSA_01
363193757_S8_L001	PSA	PSA_01
BackhedF_2015__SID203_12M__bin.9	PSA	PSA_01
BackhedF_2015__SID567_12M__bin.4	PSA	PSA_01
BackhedF_2015__SID7_M__bin.18	PSA	PSA_01
CM_madagascar__A39_02_1FE__bin.19	PSA	PSA_01
GCA_001692695.1	PSA	PSA_01
GCA_002811025.1	PSA	PSA_01
GCA_003458955.1	PSA	PSA_01
GCA_003464385.1	PSA	PSA_01

GCA_003468145.1	PSA	PSA_01
GCA_003472195.1	PSA	PSA_01
GCA_015548825.1	PSA	PSA_01
GCA_018292185.1	PSA	PSA_01
GCA_020297585.1	PSA	PSA_01
GCA_022722555.1	PSA	PSA_01
GCA_022744835.1	PSA	PSA_01
GCA_024007805.1	PSA	PSA_01
HeQ_2017__RSZAXPI003078-111__bin.2	PSA	PSA_01
HeQ_2017__SZAXPI029475-44__bin.35	PSA	PSA_01
KosticAD_2015__PRJNA231909.3113831__bin.8	PSA	PSA_01
KosticAD_2015__PRJNA231909.3113834__bin.5	PSA	PSA_01
MCT420_28_S36_L002	PSA	PSA_01
MCT420_35_S43_L002	PSA	PSA_01
MCT420_41_S49_L002	PSA	PSA_01
MCT420_6_S14_L002	PSA	PSA_01
MCT420_81_S89_L002	PSA	PSA_01
MCT420_86_S94_L002	PSA	PSA_01
MCT420_8_S16_L002	PSA	PSA_01
NielsenHB_2014__V1_CD21_4__bin.5	PSA	PSA_01
QinJ_2012__CON-005__bin.52	PSA	PSA_01
SRR12280501	PSA	PSA_01
SRR12280522	PSA	PSA_01
SRR12280591	PSA	PSA_01
SRR12280643	PSA	PSA_01
SRR13269148	PSA	PSA_01
SRR13269161	PSA	PSA_01
SRR13269220	PSA	PSA_01
SRR13269224	PSA	PSA_01
SRR13269245	PSA	PSA_01
SRR15283706	PSA	PSA_01
SRR15283746	PSA	PSA_01
SRR15283747	PSA	PSA_01
SRR15283795	PSA	PSA_01
SRR15283820	PSA	PSA_01
SRR15283834	PSA	PSA_01
SRR15283848	PSA	PSA_01
SRR15283867	PSA	PSA_01

VatanenT_2016__G78646__bin.3	PSA	PSA_01
VatanenT_2016__G78794__bin.3	PSA	PSA_01
VatanenT_2016__G78795__bin.5	PSA	PSA_01
XieH_2016__YSZC12003_37207R1__bin.36	PSA	PSA_01
XieH_2016__YSZC12003_37269__bin.22	PSA	PSA_01
YuJ_2015__SZAXPI003422-11__bin.33	PSA	PSA_01
10317.X00178995_62	PSA	PSA_02
10317.X00179306_29	PSA	PSA_02
10317.X00179680_33	PSA	PSA_02
10317.X00180464_10317.X00180464_bin.2	PSA	PSA_02
10317.X00215482_24	PSA	PSA_02
363192528_S11_L001	PSA	PSA_02
363192546_S37_L001.1	PSA	PSA_02
363192589_S80_L001	PSA	PSA_02
363192797_S102_L001	PSA	PSA_02
363193756_S6_L001	PSA	PSA_02
363193763_S14_L001	PSA	PSA_02
BackhedF_2015__SID180_4M__bin.6	PSA	PSA_02
BackhedF_2015__SID238_12M__bin.7	PSA	PSA_02
BackhedF_2015__SID238_B__bin.1	PSA	PSA_02
BackhedF_2015__SID626_4M__bin.17	PSA	PSA_02
BackhedF_2015__SID78_4M__bin.8	PSA	PSA_02
GCA_000599185.2	PSA	PSA_02
GCA_001286525.1	PSA	PSA_02
GCA_008569345.1	PSA	PSA_02
GCA_014042465.1	PSA	PSA_02
GCA_015831765.1	PSA	PSA_02
GCA_020297435.1	PSA	PSA_02
GCA_022746755.1	PSA	PSA_02
MCT420_36_S44_L002	PSA	PSA_02
SRR12280589	PSA	PSA_02
SRR13269178	PSA	PSA_02
SRR15283742	PSA	PSA_02
SRR15283767	PSA	PSA_02
SRR15283871	PSA	PSA_02
SRR9206104	PSA	PSA_02
VatanenT_2016__G78583__bin.3	PSA	PSA_02
VatanenT_2016__G80602__bin.2	PSA	PSA_02

10317.X00179025_65	PSA	PSA_03
10317.X00185882_3	PSA	PSA_03
363192998_S106_L001	PSA	PSA_03
363193028_S138_L001	PSA	PSA_03
363193046_S156_L001	PSA	PSA_03
BackhedF_2015__SID10_12M__bin.4	PSA	PSA_03
BackhedF_2015__SID274_4M__bin.2	PSA	PSA_03
BackhedF_2015__SID275_B__bin.6	PSA	PSA_03
BackhedF_2015__SID275_M__bin.26	PSA	PSA_03
BackhedF_2015__SID338_B__bin.1	PSA	PSA_03
BackhedF_2015__SID566_12M__bin.34	PSA	PSA_03
GCA_000210835.1	PSA	PSA_03
GCA_004798445.1	PSA	PSA_03
GCA_004798525.1	PSA	PSA_03
GCA_014042445.1	PSA	PSA_03
GCA_015556305.1	PSA	PSA_03
GCA_016889925.1	PSA	PSA_03
GCA_018369215.1	PSA	PSA_03
GCA_019915805.1	PSA	PSA_03
MCT420_55_S63_L002	PSA	PSA_03
SRR12280549	PSA	PSA_03
SRR12280658	PSA	PSA_03
SRR13269222	PSA	PSA_03
SRR15283826	PSA	PSA_03
SRR15283838	PSA	PSA_03
SRR15283854	PSA	PSA_03
SRR15283892	PSA	PSA_03
VatanenT_2016__G78527__bin.3	PSA	PSA_03
VatanenT_2016__G78739__bin.3	PSA	PSA_03
VogtmannE_2016__MMRS68910755ST-27-0-0__bin.44	PSA	PSA_03
10317.X00179043_48	PSA	PSA_04
10317.X00185597_21_sub	PSA	PSA_04
363192533_S31_L001	PSA	PSA_04
363192548_S39_L001	PSA	PSA_04
363192557_S48_L001	PSA	PSA_04
363192566_S57_L001	PSA	PSA_04
363193035_S145_L001	PSA	PSA_04
BackhedF_2015__SID133_4M__bin.23	PSA	PSA_04

BackhedF_2015__SID150_12M__bin.19	PSA	PSA_04
BackhedF_2015__SID150_M__bin.47	PSA	PSA_04
BackhedF_2015__SID503_12M__bin.45	PSA	PSA_04
BackhedF_2015__SID503_4M__bin.20	PSA	PSA_04
BackhedF_2015__SID570_4M__bin.7	PSA	PSA_04
BritoIL_2016__M1.10.ST__bin.49	PSA	PSA_04
ChengpingW_2017__AS123raw__bin.19	PSA	PSA_04
GCA_000598585.1	PSA	PSA_04
GCA_000598785.2	PSA	PSA_04
GCA_000598985.1	PSA	PSA_04
GCA_000965785.1	PSA	PSA_04
GCA_003469705.1	PSA	PSA_04
GCA_014042505.1	PSA	PSA_04
GCA_015553755.1	PSA	PSA_04
GCA_021202455.1	PSA	PSA_04
GCA_022727875.1	PSA	PSA_04
LiJ_2014__V1.CD3-0-PN__bin.42	PSA	PSA_04
MCT420_18_S26_L002	PSA	PSA_04
QinN_2014__LD-93__bin.8	PSA	PSA_04
SRR12280416	PSA	PSA_04
SRR12280417	PSA	PSA_04
SRR12280499	PSA	PSA_04
SRR12280511	PSA	PSA_04
SRR12280632	PSA	PSA_04
SRR13269122	PSA	PSA_04
SRR13269162	PSA	PSA_04
SRR15283687	PSA	PSA_04
SRR15283758	PSA	PSA_04
SRR15283763	PSA	PSA_04
SRR15283865	PSA	PSA_04
WenC_2017__RA132__bin.29	PSA	PSA_04
YuJ_2015__SZAXPI015216-169__bin.42	PSA	PSA_04
10317.X00179153_19	PSA	PSA_05
10317.X00179274_24	PSA	PSA_05
10317.X00179278_57	PSA	PSA_05
10317.X00179317_4	PSA	PSA_05
363192554_S45_L001.2	PSA	PSA_05
363192555_S46_L001	PSA	PSA_05

363193060_S170_L001	PSA	PSA_05
GCA_000598565.1	PSA	PSA_05
GCA_000599165.2	PSA	PSA_05
GCA_003439675.1	PSA	PSA_05
GCA_015669435.1	PSA	PSA_05
GCA_022747335.1	PSA	PSA_05
MCT420_49_S57_L002	PSA	PSA_05
MCT420_7_S15_L002	PSA	PSA_05
SRR15283701	PSA	PSA_05
SRR15283738	PSA	PSA_05
XieH_2016__YSZC12003_37879__bin.30	PSA	PSA_05
10317.X00179156_10	PSA	PSA_06
10317.X00179225_54	PSA	PSA_06
10317.X00179327_33	PSA	PSA_06
10317.X00180154_10317.X00180154_bin.17	PSA	PSA_06
10317.X00185752_7	PSA	PSA_06
10317.X00214153_64	PSA	PSA_06
363192525_S32_L001.2	PSA	PSA_06
363192552_S43_L001	PSA	PSA_06
363192577_S68_L001	PSA	PSA_06
363192585_S76_L001	PSA	PSA_06
363192595_S86_L001	PSA	PSA_06
363193049_S158_L001	PSA	PSA_06
363193064_S174_L001	PSA	PSA_06
363193076_S185_L001	PSA	PSA_06
BackhedF_2015__SID137_12M__bin.25	PSA	PSA_06
BackhedF_2015__SID137_4M__bin.2	PSA	PSA_06
BackhedF_2015__SID137_M__bin.72	PSA	PSA_06
BackhedF_2015__SID526_4M__bin.6	PSA	PSA_06
GCA_000598525.1	PSA	PSA_06
GCA_001816225.2	PSA	PSA_06
GCA_003436285.1	PSA	PSA_06
GCA_003436935.1	PSA	PSA_06
GCA_003475385.1	PSA	PSA_06
GCA_009024655.1	PSA	PSA_06
GCA_015558985.1	PSA	PSA_06
GCA_020257125.1	PSA	PSA_06
GCA_020297425.1	PSA	PSA_06

GCA_020558865.1	PSA	PSA_06
GCA_022721415.1	PSA	PSA_06
KarlssonFH_2013__S248__bin.36	PSA	PSA_06
MCT420_2_S10_L002	PSA	PSA_06
MCT420_60_S68_L002	PSA	PSA_06
QinJ_2012__NLM028__bin.25	PSA	PSA_06
SRR12280504	PSA	PSA_06
SRR12280583	PSA	PSA_06
SRR12280608	PSA	PSA_06
SRR13269164	PSA	PSA_06
SRR13269167	PSA	PSA_06
SRR13269209	PSA	PSA_06
SRR15283688	PSA	PSA_06
SRR15283868	PSA	PSA_06
VatanenT_2016__G80507__bin.5	PSA	PSA_06
10317.X00179300_11	PSA	PSA_07
10317.X00180243_84	PSA	PSA_07
10317.X00185696_11	PSA	PSA_07
GCA_000598665.1	PSA	PSA_07
GCA_000601055.1	PSA	PSA_07
GCA_000724795.2	PSA	PSA_07
GCA_015553495.1	PSA	PSA_07
GCA_020558655.1	PSA	PSA_07
GCA_022714305.1	PSA	PSA_07
GCA_022832325.1	PSA	PSA_07
GCA_022832355.1	PSA	PSA_07
GeversD_2014__SKSTL0001__bin.2	PSA	PSA_07
MCT420_58_S66_L002	PSA	PSA_07
NielsenHB_2014__O2_UC34_2__bin.41	PSA	PSA_07
SRR12280314	PSA	PSA_07
SRR12280572	PSA	PSA_07
SRR15283722	PSA	PSA_07
SRR15283762	PSA	PSA_07
SRR15283771	PSA	PSA_07
WenC_2017__N016__bin.21	PSA	PSA_07
10317.X00179564_10317.X00179564_bin.11	PSA	PSA_08
10317.X00180160_15	PSA	PSA_08
10317.X00182267_21	PSA	PSA_08

10317.X00185745_38	PSA	PSA_08
10317.X00185925_50	PSA	PSA_08
363192541_S20_L001	PSA	PSA_08
363192601_S92_L001	PSA	PSA_08
363192797_S102_L001	PSA	PSA_08
363193002_S105_L001	PSA	PSA_08
363193013_S123_L001	PSA	PSA_08
363193027_S137_L001	PSA	PSA_08
363193037_S147_L001	PSA	PSA_08
363193059_S169_L001	PSA	PSA_08
363193061_S171_L001	PSA	PSA_08
363193068_S178_L001	PSA	PSA_08
363193072_S181_L001	PSA	PSA_08
363193078_S188_L001	PSA	PSA_08
363197078_S309_L001.3	PSA	PSA_08
GCA_000273155.1	PSA	PSA_08
GCA_000273765.1	PSA	PSA_08
GCA_000598145.1	PSA	PSA_08
GCA_000598225.1	PSA	PSA_08
GCA_000598285.1	PSA	PSA_08
GCA_000601115.1	PSA	PSA_08
GCA_001056335.1	PSA	PSA_08
GCA_001682215.1	PSA	PSA_08
GCA_003437815.1	PSA	PSA_08
GCA_003472025.1	PSA	PSA_08
GCA_004167855.1	PSA	PSA_08
GCA_007896595.1	PSA	PSA_08
GCA_008369705.1	PSA	PSA_08
GCA_009025705.1	PSA	PSA_08
GCA_012844105.1	PSA	PSA_08
GCA_014042415.1	PSA	PSA_08
GCA_015548595.1	PSA	PSA_08
GCA_015553295.1	PSA	PSA_08
GCA_020297545.1	PSA	PSA_08
GCA_020539125.1	PSA	PSA_08
GCA_022733095.1	PSA	PSA_08
GCA_022745295.1	PSA	PSA_08
GCA_900624785.1	PSA	PSA_08

GCA_902374115.1	PSA	PSA_08
LiJ_2014__MH0221__bin.8	PSA	PSA_08
MCT420_10_S18_L002	PSA	PSA_08
MCT420_54_S62_L002	PSA	PSA_08
MCT420_63_S71_L002	PSA	PSA_08
RaymondF_2016__P22E7__bin.4	PSA	PSA_08
SRR12280498	PSA	PSA_08
SRR13269149	PSA	PSA_08
SRR13269154	PSA	PSA_08
SRR13269156	PSA	PSA_08
SRR13269158	PSA	PSA_08
SRR13269163	PSA	PSA_08
SRR13269219	PSA	PSA_08
SRR13269223	PSA	PSA_08
SRR15283700	PSA	PSA_08
SRR15283707	PSA	PSA_08
SRR15283713	PSA	PSA_08
SRR15283724	PSA	PSA_08
SRR15283728	PSA	PSA_08
SRR15283753	PSA	PSA_08
SRR15283828	PSA	PSA_08
SRR15283844	PSA	PSA_08
SRR15283850	PSA	PSA_08
SRR15283864	PSA	PSA_08
SRR15283869	PSA	PSA_08
SRR9206239	PSA	PSA_08
VatanenT_2016__G80584__bin.1	PSA	PSA_08
VincentC_2016__MM022.2__bin.34	PSA	PSA_08
VincentC_2016__MM060.2__bin.1	PSA	PSA_08
ZeeviD_2015__PNP_Main_319__bin.2	PSA	PSA_08
ZellerG_2014__CCIS58234805ST-4-0__bin.17	PSA	PSA_08
10317.X00179651_55	PSA	PSA_09
SRR15283873	PSA	PSA_09
10317.X00179805_8	PSA	PSA_10
10317.X00179954_75	PSA	PSA_10
10317.X00214097_10317.X00214097_bin.3	PSA	PSA_10
363193754_S16_L001.2	PSA	PSA_10
GCA_000598245.1	PSA	PSA_10

GCA_000601075.2	PSA	PSA_10
GCA_002811035.1	PSA	PSA_10
GCA_003438895.1	PSA	PSA_10
GCA_014893755.1	PSA	PSA_10
GCA_020026255.1	PSA	PSA_10
GCA_022725225.1	PSA	PSA_10
GCA_022731325.1	PSA	PSA_10
GCA_022733815.1	PSA	PSA_10
GCA_022745625.1	PSA	PSA_10
GCA_022746345.1	PSA	PSA_10
GCA_022832315.1	PSA	PSA_10
HMP_2012__SRS018817__bin.4	PSA	PSA_10
LeChatelierE_2013__MH0193__bin.44	PSA	PSA_10
SRR13269166	PSA	PSA_10
SRR15283750	PSA	PSA_10
SRR15283876	PSA	PSA_10
YuJ_2015__SZAXPI003435-11__bin.64	PSA	PSA_10
YuJ_2015__SZAXPI015250-39__bin.22	PSA	PSA_10
10317.X00180334_10317.X00180334_bin.001	PSA	PSA_11
10317.X00180334_10317.X00180334_bin.1	PSA	PSA_11
10317.X00180463_38	PSA	PSA_11
363192539_S19_L001	PSA	PSA_11
363193043_S153_L001	PSA	PSA_11
363193054_S164_L001	PSA	PSA_11
BackhedF_2015__SID546_12M__bin.30	PSA	PSA_11
BackhedF_2015__SID546_4M__bin.5	PSA	PSA_11
GCA_000297735.1	PSA	PSA_11
GCA_000598845.1	PSA	PSA_11
GCA_000599065.2	PSA	PSA_11
GCA_008829725.1	PSA	PSA_11
GCA_015669295.1	PSA	PSA_11
GCA_022716295.1	PSA	PSA_11
GCA_022782465.1	PSA	PSA_11
HeQ_2017__RSZAXPI003062-102__bin.4	PSA	PSA_11
KosticAD_2015__PRJNA231909.3101436__bin.15	PSA	PSA_11
KosticAD_2015__PRJNA231909.3101439__bin.2	PSA	PSA_11
KosticAD_2015__PRJNA231909.3107649__bin.14	PSA	PSA_11
MCT420_50_S58_L002	PSA	PSA_11

MCT420_75_S83_L002	PSA	PSA_11
NielsenHB_2014__V1_CD6_4__bin.58	PSA	PSA_11
SRR12280270	PSA	PSA_11
SRR12280315	PSA	PSA_11
SRR12280500	PSA	PSA_11
SRR12280649	PSA	PSA_11
SRR12280650	PSA	PSA_11
SRR13269150	PSA	PSA_11
SRR13269155	PSA	PSA_11
SRR13269159	PSA	PSA_11
SRR13269165	PSA	PSA_11
SRR15283708	PSA	PSA_11
SRR15283714	PSA	PSA_11
SRR15283720	PSA	PSA_11
SRR15283752	PSA	PSA_11
SRR15283757	PSA	PSA_11
SRR15283837	PSA	PSA_11
SRR15283842	PSA	PSA_11
SRR9206393	PSA	PSA_11
VatanenT_2016__G78592__bin.3	PSA	PSA_11
VincentC_2016__MM032.3__bin.19	PSA	PSA_11
10317.X00185507_10317.X00185507_bin.17	PSA	PSA_12
10317.X00214127_44_sub	PSA	PSA_12
BackhedF_2015__SID130_12M__bin.13	PSA	PSA_12
BackhedF_2015__SID130_4M__bin.3	PSA	PSA_12
BackhedF_2015__SID587_12M__bin.17	PSA	PSA_12
BackhedF_2015__SID615_12M__bin.2	PSA	PSA_12
BackhedF_2015__SID615_4M__bin.5	PSA	PSA_12
GCA_000598905.1	PSA	PSA_12
GCA_000601095.1	PSA	PSA_12
GCA_001699855.1	PSA	PSA_12
GCA_014042525.1	PSA	PSA_12
GCA_905188165.1	PSA	PSA_12
MCT420_77_S85_L002	PSA	PSA_12
SRR13269111	PSA	PSA_12
SRR13269207	PSA	PSA_12
SRR15283695	PSA	PSA_12
SRR15283731	PSA	PSA_12

SRR15283896	PSA	PSA_12
XieH_2016__YSZC12003_37205__bin.26	PSA	PSA_12
XieH_2016__YSZC12003_37880__bin.18	PSA	PSA_12
10317.X00214096_10317.X00214096_bin.002	PSA	PSA_13
363192542_S33_L001.3	PSA	PSA_13
363192560_S51_L001	PSA	PSA_13
363192793_S98_L001	PSA	PSA_13
363193044_S154_L001	PSA	PSA_13
BackhedF_2015__SID1_4M__bin.8	PSA	PSA_13
BackhedF_2015__SID229_B__bin.3	PSA	PSA_13
GCA_000598205.1	PSA	PSA_13
GCA_001699875.1	PSA	PSA_13
GCA_003439505.1	PSA	PSA_13
GCA_003463555.1	PSA	PSA_13
GCA_015548495.1	PSA	PSA_13
GCA_018786025.1	PSA	PSA_13
GCA_019733435.1	PSA	PSA_13
MCT420_80_S88_L002	PSA	PSA_13
NielsenHB_2014__V1_UC15_0__bin.1	PSA	PSA_13
NielsenHB_2014__V1_UC15_3__bin.64	PSA	PSA_13
SRR12280492	PSA	PSA_13
SRR12280561	PSA	PSA_13
SRR12280645	PSA	PSA_13
SRR12280651	PSA	PSA_13
SRR15283891	PSA	PSA_13
363192559_S50_L001	PSA	PSA_14
363192568_S59_L001	PSA	PSA_14
363192575_S66_L001.1	PSA	PSA_14
363192579_S70_L001	PSA	PSA_14
363192583_S74_L001	PSA	PSA_14
363192591_S82_L001	PSA	PSA_14
363193032_S142_L001	PSA	PSA_14
363193040_S150_L001	PSA	PSA_14
363193047_S157_L001	PSA	PSA_14
363193063_S173_L001.2	PSA	PSA_14
GCA_000218345.1	PSA	PSA_14
GCA_000599325.1	PSA	PSA_14
GCA_001058775.1	PSA	PSA_14

GCA_001699865.1	PSA	PSA_14
GCA_002811085.1	PSA	PSA_14
GCA_003458215.1	PSA	PSA_14
GCA_013327835.1	PSA	PSA_14
GCA_015551365.1	PSA	PSA_14
GCA_019130425.1	PSA	PSA_14
GCA_023702735.1	PSA	PSA_14
QinN_2014__LD-33__bin.18	PSA	PSA_14
SRR12280336	PSA	PSA_14
SRR12280493	PSA	PSA_14
SRR12280635	PSA	PSA_14
SRR12280646	PSA	PSA_14
SRR13269157	PSA	PSA_14
SRR13269204	PSA	PSA_14
SRR13269221	PSA	PSA_14
SRR15283691	PSA	PSA_14
SRR15283697	PSA	PSA_14
SRR15283749	PSA	PSA_14
SRR15283766	PSA	PSA_14
SRR15283839	PSA	PSA_14
SRR15283861	PSA	PSA_14
SRR15283894	PSA	PSA_14
VogtmannE_2016__MMRS62192467ST-27-0-0__bin.44	PSA	PSA_14
VogtmannE_2016__MMRS72980899ST-27-0-0__bin.27	PSA	PSA_14
WenC_2017__N281__bin.22	PSA	PSA_14
XieH_2016__YSZC12003_35524__bin.22	PSA	PSA_14
XieH_2016__YSZC12003_36052__bin.34	PSA	PSA_14
10317.X00178933_38	PSB	PSB_01
10317.X00179327_33	PSB	PSB_01
10317.X00179407_24	PSB	PSB_01
10317.X00180154_10317.X00180154_bin.17	PSB	PSB_01
10317.X00185752_7	PSB	PSB_01
10317.X00185882_3	PSB	PSB_01
363192522_S5_L001	PSB	PSB_01
363192575_S66_L001.1	PSB	PSB_01
363192577_S68_L001	PSB	PSB_01
363192583_S74_L001	PSB	PSB_01
363192585_S76_L001	PSB	PSB_01

363192795_S100_L001	PSB	PSB_01
BackhedF_2015__SID10_12M__bin.4	PSB	PSB_01
BackhedF_2015__SID566_12M__bin.34	PSB	PSB_01
BackhedF_2015__SID587_12M__bin.17	PSB	PSB_01
BackhedF_2015__SID615_12M__bin.2	PSB	PSB_01
BackhedF_2015__SID615_4M__bin.5	PSB	PSB_01
BackhedF_2015__SID7_M__bin.18	PSB	PSB_01
GCA_000598545.2	PSB	PSB_01
GCA_000599365.1	PSB	PSB_01
GCA_000601095.1	PSB	PSB_01
GCA_003469305.1	PSB	PSB_01
GCA_009024655.1	PSB	PSB_01
GCA_015551365.1	PSB	PSB_01
HeQ_2017__SZAXPI029475-44__bin.35	PSB	PSB_01
KarlssonFH_2013__S248__bin.36	PSB	PSB_01
NielsenHB_2014__V1_CD21_4__bin.5	PSB	PSB_01
SRR12280315	PSB	PSB_01
SRR12280493	PSB	PSB_01
SRR12280583	PSB	PSB_01
SRR12280591	PSB	PSB_01
SRR12280608	PSB	PSB_01
SRR13269157	PSB	PSB_01
SRR13269167	PSB	PSB_01
SRR13269204	PSB	PSB_01
SRR13269224	PSB	PSB_01
SRR15283691	PSB	PSB_01
SRR15283714	PSB	PSB_01
SRR15283731	PSB	PSB_01
SRR15283753	PSB	PSB_01
SRR15283757	PSB	PSB_01
SRR15283848	PSB	PSB_01
SRR15283867	PSB	PSB_01
SRR15283868	PSB	PSB_01
VatanenT_2016__G80507__bin.5	PSB	PSB_01
VatanenT_2016__G80556__bin.10	PSB	PSB_01
VogtmannE_2016__MMRS72980899ST-27-0-0__bin.27	PSB	PSB_01
XieH_2016__YSZC12003_36052__bin.34	PSB	PSB_01
10317.X00179017_10317.X00179017_bin.7	PSB	PSB_02

10317.X00179047_10317.X00179047_bin.7	PSB	PSB_02
10317.X00179317_4	PSB	PSB_02
10317.X00180334_10317.X00180334_bin.001	PSB	PSB_02
10317.X00180334_10317.X00180334_bin.1	PSB	PSB_02
363192527_S10_L001	PSB	PSB_02
363192541_S20_L001	PSB	PSB_02
363192544_S35_L001	PSB	PSB_02
363192554_S45_L001.2	PSB	PSB_02
363192555_S46_L001	PSB	PSB_02
363192573_S64_L001	PSB	PSB_02
363192581_S72_L001	PSB	PSB_02
363192601_S92_L001	PSB	PSB_02
363192602_S93_L001	PSB	PSB_02
363192605_S96_L001	PSB	PSB_02
363193001_S108_L001	PSB	PSB_02
363193004_S114_L001	PSB	PSB_02
363193022_S130_L001.1	PSB	PSB_02
363193031_S141_L001.1	PSB	PSB_02
363193036_S146_L001	PSB	PSB_02
363193043_S153_L001	PSB	PSB_02
363193068_S178_L001	PSB	PSB_02
363193757_S8_L001	PSB	PSB_02
363197054_S337_L001.2	PSB	PSB_02
BackhedF_2015__SID133_4M__bin.23	PSB	PSB_02
BackhedF_2015__SID203_12M__bin.9	PSB	PSB_02
GCA_000297735.1	PSB	PSB_02
GCA_000598845.1	PSB	PSB_02
GCA_002811025.1	PSB	PSB_02
GCA_003472195.1	PSB	PSB_02
GCA_014042455.1	PSB	PSB_02
GCA_015548825.1	PSB	PSB_02
GCA_015669295.1	PSB	PSB_02
GCA_019013355.1	PSB	PSB_02
GCA_020297585.1	PSB	PSB_02
GCA_022722555.1	PSB	PSB_02
GCA_022782465.1	PSB	PSB_02
GCA_024007805.1	PSB	PSB_02
HeQ_2017__RSZAXPI003078-111__bin.2	PSB	PSB_02

HeQ_2017__RSZAXPI003098-132__bin.12	PSB	PSB_02
LiJ_2014__V1.FI21__bin.42	PSB	PSB_02
MCT420_29_S37_L002	PSB	PSB_02
MCT420_50_S58_L002	PSB	PSB_02
MCT420_75_S83_L002	PSB	PSB_02
MCT420_8_S16_L002	PSB	PSB_02
QinN_2014__LD-93__bin.8	PSB	PSB_02
SRR12280649	PSB	PSB_02
SRR13269150	PSB	PSB_02
SRR13269159	PSB	PSB_02
SRR13269161	PSB	PSB_02
SRR13269162	PSB	PSB_02
SRR13269223	PSB	PSB_02
SRR15283687	PSB	PSB_02
SRR15283720	PSB	PSB_02
SRR15283747	PSB	PSB_02
SRR15283763	PSB	PSB_02
SRR15283795	PSB	PSB_02
SRR15283864	PSB	PSB_02
SRR15283873	PSB	PSB_02
VatanenT_2016__G78592__bin.3	PSB	PSB_02
10317.X00179025_65	PSB	PSB_03
363192998_S106_L001	PSB	PSB_03
363193028_S138_L001	PSB	PSB_03
363193046_S156_L001	PSB	PSB_03
BackhedF_2015__SID274_4M__bin.2	PSB	PSB_03
GCA_000210835.1	PSB	PSB_03
GCA_001692695.1	PSB	PSB_03
GCA_001699865.1	PSB	PSB_03
GCA_003439675.1	PSB	PSB_03
GCA_004798445.1	PSB	PSB_03
GCA_004798525.1	PSB	PSB_03
GCA_018369215.1	PSB	PSB_03
GCA_019130425.1	PSB	PSB_03
GCA_019915805.1	PSB	PSB_03
GCA_022725025.1	PSB	PSB_03
GCA_022733395.1	PSB	PSB_03
GCA_022734285.1	PSB	PSB_03

GCA_022741265.1	PSB	PSB_03
GCA_022746405.1	PSB	PSB_03
GCA_023702735.1	PSB	PSB_03
MCT420_63_S71_L002	PSB	PSB_03
QinJ_2012__CON-005__bin.52	PSB	PSB_03
SRR12280549	PSB	PSB_03
SRR12280658	PSB	PSB_03
SRR13269122	PSB	PSB_03
SRR13269221	PSB	PSB_03
SRR13269222	PSB	PSB_03
SRR15283738	PSB	PSB_03
SRR15283839	PSB	PSB_03
VatanenT_2016__G78527__bin.3	PSB	PSB_03
WenC_2017__N016__bin.21	PSB	PSB_03
XieH_2016__YSZC12003_35524__bin.22	PSB	PSB_03
XieH_2016__YSZC12003_37205__bin.26	PSB	PSB_03
10317.X00179093_41	PSB	PSB_04
10317.X00179725_20	PSB	PSB_04
10317.X00179729_16	PSB	PSB_04
10317.X00180463_38	PSB	PSB_04
10317.X00215088_24	PSB	PSB_04
363193002_S105_L001	PSB	PSB_04
363193040_S150_L001	PSB	PSB_04
363193049_S158_L001	PSB	PSB_04
363193060_S170_L001	PSB	PSB_04
363193064_S174_L001	PSB	PSB_04
363193076_S185_L001	PSB	PSB_04
363197065_S315_L001	PSB	PSB_04
BackhedF_2015__SID137_12M__bin.25	PSB	PSB_04
BackhedF_2015__SID137_4M__bin.2	PSB	PSB_04
BackhedF_2015__SID137_M__bin.72	PSB	PSB_04
BackhedF_2015__SID180_4M__bin.6	PSB	PSB_04
BackhedF_2015__SID526_4M__bin.6	PSB	PSB_04
BackhedF_2015__SID565_4M__bin.10	PSB	PSB_04
BackhedF_2015__SID582_12M__bin.6	PSB	PSB_04
BackhedF_2015__SID591_4M__bin.17	PSB	PSB_04
GCA_000598565.1	PSB	PSB_04
GCA_000598705.1	PSB	PSB_04

GCA_000598905.1	PSB	PSB_04
GCA_000599165.2	PSB	PSB_04
GCA_000599185.2	PSB	PSB_04
GCA_000601115.1	PSB	PSB_04
GCA_001699855.1	PSB	PSB_04
GCA_001699875.1	PSB	PSB_04
GCA_001816225.2	PSB	PSB_04
GCA_008086695.1	PSB	PSB_04
GCA_015556305.1	PSB	PSB_04
GCA_020257125.1	PSB	PSB_04
GCA_020297425.1	PSB	PSB_04
GCA_020297545.1	PSB	PSB_04
GCA_020558865.1	PSB	PSB_04
GCA_022725225.1	PSB	PSB_04
GCA_022733095.1	PSB	PSB_04
GCA_022734035.1	PSB	PSB_04
GCA_022737795.1	PSB	PSB_04
GCA_022744835.1	PSB	PSB_04
GCA_022745295.1	PSB	PSB_04
GCA_022745625.1	PSB	PSB_04
GCA_022746345.1	PSB	PSB_04
GCA_022747335.1	PSB	PSB_04
GCA_900624785.1	PSB	PSB_04
GCA_902374115.1	PSB	PSB_04
HMP_2012__SRS075398__bin.15	PSB	PSB_04
KosticAD_2015__PRJNA231909.3112348__bin.8	PSB	PSB_04
MCT420_32_S40_L002	PSB	PSB_04
MCT420_54_S62_L002	PSB	PSB_04
MCT420_77_S85_L002	PSB	PSB_04
MCT420_7_S15_L002	PSB	PSB_04
Obregon-TitoAJ_2015__NO05__bin.48	PSB	PSB_04
QinJ_2012__NLM028__bin.25	PSB	PSB_04
QinN_2014__LD-26__bin.50	PSB	PSB_04
SRR12280501	PSB	PSB_04
SRR13269155	PSB	PSB_04
SRR13269158	PSB	PSB_04
SRR13269165	PSB	PSB_04
SRR13269207	PSB	PSB_04

SRR13269209	PSB	PSB_04
SRR13269219	PSB	PSB_04
SRR13269220	PSB	PSB_04
SRR15283688	PSB	PSB_04
SRR15283701	PSB	PSB_04
SRR15283728	PSB	PSB_04
SRR15283752	PSB	PSB_04
VatanenT_2016__G80499__bin.3	PSB	PSB_04
VatanenT_2016__G80601__bin.2	PSB	PSB_04
VogtmannE_2016__MMRS77321055ST-27-0-0__bin.39	PSB	PSB_04
ZeeviD_2015__PNP_Main_174__bin.22	PSB	PSB_04
ZeeviD_2015__PNP_Validation_92__bin.15	PSB	PSB_04
10317.X00179153_19	PSB	PSB_05
10317.X00179805_8	PSB	PSB_05
GCA_000598525.1	PSB	PSB_05
GCA_003436935.1	PSB	PSB_05
GCA_014893755.1	PSB	PSB_05
XieH_2016__YSZC12003_37880__bin.18	PSB	PSB_05
YuJ_2015__SZAXPI003435-11__bin.64	PSB	PSB_05
YuJ_2015__SZAXPI015250-39__bin.22	PSB	PSB_05
10317.X00179156_10	PSB	PSB_06
10317.X00179225_54	PSB	PSB_06
10317.X00179300_11	PSB	PSB_06
10317.X00185507_10317.X00185507_bin.17	PSB	PSB_06
10317.X00185696_11	PSB	PSB_06
363192525_S32_L001.2	PSB	PSB_06
363192595_S86_L001	PSB	PSB_06
363193039_S149_L001	PSB	PSB_06
BackhedF_2015__SID626_4M__bin.17	PSB	PSB_06
GCA_000598305.1	PSB	PSB_06
GCA_000598505.1	PSB	PSB_06
GCA_000724795.2	PSB	PSB_06
GCA_014042525.1	PSB	PSB_06
GCA_015558985.1	PSB	PSB_06
GCA_022721415.1	PSB	PSB_06
GCA_022832325.1	PSB	PSB_06
GCA_022832355.1	PSB	PSB_06
MCT420_2_S10_L002	PSB	PSB_06

MCT420_52_S60_L002	PSB	PSB_06
SRR12280503	PSB	PSB_06
SRR12280504	PSB	PSB_06
SRR15283685	PSB	PSB_06
SRR15283695	PSB	PSB_06
SRR15283700	PSB	PSB_06
SRR15283722	PSB	PSB_06
SRR15283749	PSB	PSB_06
SRR15283762	PSB	PSB_06
SRR15283771	PSB	PSB_06
SRR15283822	PSB	PSB_06
SRR15283896	PSB	PSB_06
SRR9206393	PSB	PSB_06
VatanenT_2016__G80602__bin.2	PSB	PSB_06
10317.X00179298_6	PSB	PSB_07
10317.X00179673_25	PSB	PSB_07
363192603_S94_L001	PSB	PSB_07
363192805_S110_L001	PSB	PSB_07
363193029_S139_L001	PSB	PSB_07
363193048_S163_L001	PSB	PSB_07
363193069_S179_L001	PSB	PSB_07
GCA_018292185.1	PSB	PSB_07
HeQ_2017__SZAXPI029574-89__bin.37	PSB	PSB_07
MCT420_28_S36_L002	PSB	PSB_07
SRR15283834	PSB	PSB_07
10317.X00179306_29	PSB	PSB_08
10317.X00185745_38	PSB	PSB_08
363192528_S11_L001	PSB	PSB_08
363192563_S54_L001	PSB	PSB_08
363192579_S70_L001	PSB	PSB_08
363192589_S80_L001	PSB	PSB_08
363192797_S102_L001	PSB	PSB_08
363193059_S169_L001	PSB	PSB_08
363193072_S181_L001	PSB	PSB_08
363193078_S188_L001	PSB	PSB_08
363193756_S6_L001	PSB	PSB_08
363193763_S14_L001	PSB	PSB_08
BackhedF_2015__SID338_B__bin.1	PSB	PSB_08

BackhedF_2015__SID78_4M__bin.8	PSB	PSB_08
GCA_000598225.1	PSB	PSB_08
GCA_001056335.1	PSB	PSB_08
GCA_001286525.1	PSB	PSB_08
GCA_001682215.1	PSB	PSB_08
GCA_003437815.1	PSB	PSB_08
GCA_003458955.1	PSB	PSB_08
GCA_003464385.1	PSB	PSB_08
GCA_003468145.1	PSB	PSB_08
GCA_003472025.1	PSB	PSB_08
GCA_004167855.1	PSB	PSB_08
GCA_007896595.1	PSB	PSB_08
GCA_008569345.1	PSB	PSB_08
GCA_014042415.1	PSB	PSB_08
GCA_014042465.1	PSB	PSB_08
GCA_015548595.1	PSB	PSB_08
GCA_015669435.1	PSB	PSB_08
GCA_020297435.1	PSB	PSB_08
GCA_020539125.1	PSB	PSB_08
LiJ_2014__MH0221__bin.8	PSB	PSB_08
MCT420_36_S44_L002	PSB	PSB_08
SRR12280589	PSB	PSB_08
SRR13269149	PSB	PSB_08
SRR13269154	PSB	PSB_08
SRR13269156	PSB	PSB_08
SRR13269163	PSB	PSB_08
SRR15283707	PSB	PSB_08
SRR15283742	PSB	PSB_08
SRR15283820	PSB	PSB_08
SRR15283828	PSB	PSB_08
SRR15283838	PSB	PSB_08
SRR15283850	PSB	PSB_08
SRR15283861	PSB	PSB_08
SRR15283869	PSB	PSB_08
SRR15283894	PSB	PSB_08
VatanenT_2016__G78583__bin.3	PSB	PSB_08
10317.X00179954_75	PSB	PSB_09
363193754_S16_L001.2	PSB	PSB_09

GCA_000598245.1	PSB	PSB_09
GCA_000599125.1	PSB	PSB_09
GCA_000601075.2	PSB	PSB_09
GCA_002811035.1	PSB	PSB_09
GCA_003438895.1	PSB	PSB_09
GCA_013327835.1	PSB	PSB_09
GCA_022832315.1	PSB	PSB_09
HMP_2012__SRS018817__bin.4	PSB	PSB_09
SRR12280336	PSB	PSB_09
SRR12280496	PSB	PSB_09
SRR12280643	PSB	PSB_09
SRR13269148	PSB	PSB_09
SRR13269166	PSB	PSB_09
SRR15283750	PSB	PSB_09
VatanenT_2016__G78646__bin.3	PSB	PSB_09
VatanenT_2016__G78794__bin.3	PSB	PSB_09
VatanenT_2016__G78795__bin.5	PSB	PSB_09
YuJ_2015__SZAXPI003422-11__bin.33	PSB	PSB_09
ZellerG_2014__CCIS76845094ST-20-0__bin.49	PSB	PSB_09
10317.X00214096_10317.X00214096_bin.002	PSB	PSB_10
10317.X00214127_44_sub	PSB	PSB_10
10317.X00214694_67	PSB	PSB_10
363192542_S33_L001.3	PSB	PSB_10
363192548_S39_L001	PSB	PSB_10
363192557_S48_L001	PSB	PSB_10
363192560_S51_L001	PSB	PSB_10
BackhedF_2015__SID150_12M__bin.19	PSB	PSB_10
BackhedF_2015__SID150_M__bin.47	PSB	PSB_10
BackhedF_2015__SID229_B__bin.3	PSB	PSB_10
BackhedF_2015__SID238_12M__bin.7	PSB	PSB_10
BackhedF_2015__SID238_B__bin.1	PSB	PSB_10
BackhedF_2015__SID503_12M__bin.45	PSB	PSB_10
BackhedF_2015__SID503_4M__bin.20	PSB	PSB_10
GCA_000598205.1	PSB	PSB_10
GCA_000598365.1	PSB	PSB_10
GCA_000598785.2	PSB	PSB_10
GCA_000965785.1	PSB	PSB_10
GCA_003439505.1	PSB	PSB_10

GCA_003463555.1	PSB	PSB_10
GCA_003469705.1	PSB	PSB_10
GCA_015548495.1	PSB	PSB_10
GCA_018786025.1	PSB	PSB_10
GCA_019733435.1	PSB	PSB_10
GCA_022735135.1	PSB	PSB_10
NielsenHB_2014__V1_UC15_0__bin.1	PSB	PSB_10
NielsenHB_2014__V1_UC15_3__bin.64	PSB	PSB_10
SRR12280416	PSB	PSB_10
SRR12280499	PSB	PSB_10
SRR12280511	PSB	PSB_10
SRR12280561	PSB	PSB_10
SRR12280632	PSB	PSB_10
SRR12280645	PSB	PSB_10
SRR13269111	PSB	PSB_10
SRR13269178	PSB	PSB_10
SRR15283844	PSB	PSB_10
SRR15283865	PSB	PSB_10
WenC_2017__RA132__bin.29	PSB	PSB_10
YuJ_2015__SZAXPI015216-169__bin.42	PSB	PSB_10
363192053_S223_L001.4	PSB	PSB_11
363192533_S31_L001	PSB	PSB_11
363192539_S19_L001	PSB	PSB_11
363192566_S57_L001	PSB	PSB_11
363192793_S98_L001	PSB	PSB_11
363193032_S142_L001	PSB	PSB_11
363193044_S154_L001	PSB	PSB_11
363193054_S164_L001	PSB	PSB_11
BackhedF_2015__SID546_12M__bin.30	PSB	PSB_11
BackhedF_2015__SID546_4M__bin.5	PSB	PSB_11
BackhedF_2015__SID570_4M__bin.7	PSB	PSB_11
BritoIL_2016__M1.10.ST__bin.49	PSB	PSB_11
ChengpingW_2017__AS123raw__bin.19	PSB	PSB_11
GCA_000598985.1	PSB	PSB_11
GCA_000599065.2	PSB	PSB_11
GCA_015560515.1	PSB	PSB_11
GCA_015831765.1	PSB	PSB_11
GCA_022716295.1	PSB	PSB_11

GCA_022727875.1	PSB	PSB_11
GCA_022733815.1	PSB	PSB_11
KosticAD_2015__PRJNA231909.3101436__bin.15	PSB	PSB_11
KosticAD_2015__PRJNA231909.3107649__bin.14	PSB	PSB_11
LiJ_2014__V1.CD3-0-PN__bin.42	PSB	PSB_11
MCT420_49_S57_L002	PSB	PSB_11
MCT420_80_S88_L002	PSB	PSB_11
QinN_2014__LD-50__bin.33	PSB	PSB_11
SRR12280500	PSB	PSB_11
SRR12280651	PSB	PSB_11
SRR13269194	PSB	PSB_11
SRR15283708	PSB	PSB_11
SRR15283837	PSB	PSB_11
SRR15283842	PSB	PSB_11
SRR15283876	PSB	PSB_11
SRR9206104	PSB	PSB_11
363192546_S37_L001.1	PSB	PSB_12
363193013_S123_L001	PSB	PSB_12
363193027_S137_L001	PSB	PSB_12
GCA_000273765.1	PSB	PSB_12
RaymondF_2016__P22E7__bin.4	PSB	PSB_12
SRR12280498	PSB	PSB_12
SRR15283767	PSB	PSB_12
363192552_S43_L001	PSB	PSB_13
363193037_S147_L001	PSB	PSB_13
363193079_S189_L001	PSB	PSB_13
BackhedF_2015__SID521_4M__bin.12	PSB	PSB_13
BackhedF_2015__SID53_4M__bin.14	PSB	PSB_13
GCA_000273155.1	PSB	PSB_13
GCA_022780605.1	PSB	PSB_13
MCT420_47_S55_L002	PSB	PSB_13
NielsenHB_2014__V1_UC34_0__bin.49	PSB	PSB_13
SRR15283713	PSB	PSB_13
VincentC_2016__MM060.2__bin.1	PSB	PSB_13
363192559_S50_L001	PSB	PSB_14
363192591_S82_L001	PSB	PSB_14
GCA_001058775.1	PSB	PSB_14
SRR12280635	PSB	PSB_14

363192999_S107_L001	PSB	PSB_15
363193035_S145_L001	PSB	PSB_15
GCA_000598585.1	PSB	PSB_15
GCA_003436285.1	PSB	PSB_15
GCA_003475385.1	PSB	PSB_15
GCA_014042505.1	PSB	PSB_15
GCA_015553755.1	PSB	PSB_15
MCT420_18_S26_L002	PSB	PSB_15
MCT420_59_S67_L002	PSB	PSB_15
SRR15283758	PSB	PSB_15
363193061_S171_L001	PSB	PSB_16
GCA_009025705.1	PSB	PSB_16
GCA_012844105.1	PSB	PSB_16
GCA_015553295.1	PSB	PSB_16
SRR12280270	PSB	PSB_16
BackhedF_2015__SID130_12M__bin.13	PSB	PSB_17
BackhedF_2015__SID130_4M__bin.3	PSB	PSB_17
GCA_014042445.1	PSB	PSB_18
GCA_016889925.1	PSB	PSB_18
SRR15283854	PSB	PSB_18
SRR15283892	PSB	PSB_18
VogtmannE_2016__MMRS68910755ST-27-0-0__bin.44	PSB	PSB_18
SRR15283746	PSB	PSB_19
10317.X00178933_38	PSC	PSC_01
10317.X00179043_48	PSC	PSC_01
10317.X00179225_54	PSC	PSC_01
10317.X00179306_29	PSC	PSC_01
10317.X00180464_10317.X00180464_bin.2	PSC	PSC_01
10317.X00185597_21_sub	PSC	PSC_01
10317.X00214812_39	PSC	PSC_01
363192522_S5_L001	PSC	PSC_01
363192525_S32_L001.2	PSC	PSC_01
363192528_S11_L001	PSC	PSC_01
363192541_S20_L001	PSC	PSC_01
363192548_S39_L001	PSC	PSC_01
363192557_S48_L001	PSC	PSC_01
363192589_S80_L001	PSC	PSC_01
363192595_S86_L001	PSC	PSC_01

363193035_S145_L001	PSC	PSC_01
363193037_S147_L001	PSC	PSC_01
363193060_S170_L001	PSC	PSC_01
363193079_S189_L001	PSC	PSC_01
BackhedF_2015__SID133_4M__bin.23	PSC	PSC_01
BackhedF_2015__SID150_12M__bin.19	PSC	PSC_01
BackhedF_2015__SID150_M__bin.47	PSC	PSC_01
BackhedF_2015__SID503_12M__bin.45	PSC	PSC_01
BackhedF_2015__SID503_4M__bin.20	PSC	PSC_01
BackhedF_2015__SID570_4M__bin.7	PSC	PSC_01
BackhedF_2015__SID78_4M__bin.8	PSC	PSC_01
BackhedF_2015__SID7_M__bin.18	PSC	PSC_01
GCA_000210835.1	PSC	PSC_01
GCA_000273155.1	PSC	PSC_01
GCA_000598525.1	PSC	PSC_01
GCA_000598565.1	PSC	PSC_01
GCA_000598585.1	PSC	PSC_01
GCA_000598665.1	PSC	PSC_01
GCA_000598785.2	PSC	PSC_01
GCA_000598905.1	PSC	PSC_01
GCA_000599165.2	PSC	PSC_01
GCA_000599325.1	PSC	PSC_01
GCA_000601055.1	PSC	PSC_01
GCA_000965785.1	PSC	PSC_01
GCA_001286525.1	PSC	PSC_01
GCA_001692695.1	PSC	PSC_01
GCA_001699855.1	PSC	PSC_01
GCA_003436935.1	PSC	PSC_01
GCA_003469705.1	PSC	PSC_01
GCA_014042465.1	PSC	PSC_01
GCA_014042505.1	PSC	PSC_01
GCA_015553755.1	PSC	PSC_01
GCA_015558985.1	PSC	PSC_01
GCA_018369215.1	PSC	PSC_01
GCA_020297425.1	PSC	PSC_01
GCA_020297435.1	PSC	PSC_01
GCA_022780605.1	PSC	PSC_01
HeQ_2017__SZAXPI029475-44__bin.35	PSC	PSC_01

LeChatelierE_2013__MH0410__bin.30	PSC	PSC_01
LiJ_2014__MH0221__bin.8	PSC	PSC_01
MCT420_18_S26_L002	PSC	PSC_01
MCT420_2_S10_L002	PSC	PSC_01
MCT420_36_S44_L002	PSC	PSC_01
MCT420_47_S55_L002	PSC	PSC_01
MCT420_59_S67_L002	PSC	PSC_01
MCT420_77_S85_L002	PSC	PSC_01
MCT420_7_S15_L002	PSC	PSC_01
QinJ_2012__CON-005__bin.52	PSC	PSC_01
SRR12280336	PSC	PSC_01
SRR12280416	PSC	PSC_01
SRR12280417	PSC	PSC_01
SRR12280499	PSC	PSC_01
SRR12280504	PSC	PSC_01
SRR12280511	PSC	PSC_01
SRR12280549	PSC	PSC_01
SRR12280572	PSC	PSC_01
SRR12280589	PSC	PSC_01
SRR12280632	PSC	PSC_01
SRR12280651	PSC	PSC_01
SRR12280658	PSC	PSC_01
SRR13269154	PSC	PSC_01
SRR13269162	PSC	PSC_01
SRR13269207	PSC	PSC_01
SRR13269222	PSC	PSC_01
SRR15283687	PSC	PSC_01
SRR15283701	PSC	PSC_01
SRR15283713	PSC	PSC_01
SRR15283742	PSC	PSC_01
SRR15283746	PSC	PSC_01
SRR15283758	PSC	PSC_01
SRR15283763	PSC	PSC_01
SRR15283767	PSC	PSC_01
SRR15283820	PSC	PSC_01
SRR15283838	PSC	PSC_01
SRR15283848	PSC	PSC_01
SRR15283865	PSC	PSC_01

SRR15283867	PSC	PSC_01
VatanenT_2016__G78526__bin.12	PSC	PSC_01
VatanenT_2016__G78583__bin.3	PSC	PSC_01
VatanenT_2016__G80313__bin.13	PSC	PSC_01
VatanenT_2016__G80592__bin.1	PSC	PSC_01
VincentC_2016__MM060.2__bin.1	PSC	PSC_01
YuJ_2015__SZAXPI015216-169__bin.42	PSC	PSC_01
10317.X00178992_47	PSC	PSC_02
10317.X00179047_10317.X00179047_bin.7	PSC	PSC_02
10317.X00182267_21	PSC	PSC_02
363192539_S19_L001	PSC	PSC_02
363192555_S46_L001	PSC	PSC_02
363192563_S54_L001	PSC	PSC_02
363192602_S93_L001	PSC	PSC_02
363192605_S96_L001	PSC	PSC_02
363193031_S141_L001.1	PSC	PSC_02
BackhedF_2015__SID172_12M__bin.1	PSC	PSC_02
BackhedF_2015__SID180_4M__bin.6	PSC	PSC_02
BackhedF_2015__SID546_12M__bin.30	PSC	PSC_02
BackhedF_2015__SID546_4M__bin.5	PSC	PSC_02
GCA_000598145.1	PSC	PSC_02
GCA_000598285.1	PSC	PSC_02
GCA_000598825.1	PSC	PSC_02
GCA_001682215.1	PSC	PSC_02
GCA_002811025.1	PSC	PSC_02
GCA_003458955.1	PSC	PSC_02
GCA_003464385.1	PSC	PSC_02
GCA_003468145.1	PSC	PSC_02
GCA_015548825.1	PSC	PSC_02
GCA_020859785.1	PSC	PSC_02
GCA_022733095.1	PSC	PSC_02
KosticAD_2015__PRJNA231909.3101436__bin.15	PSC	PSC_02
KosticAD_2015__PRJNA231909.3101439__bin.2	PSC	PSC_02
KosticAD_2015__PRJNA231909.3107649__bin.14	PSC	PSC_02
MCT420_35_S43_L002	PSC	PSC_02
SRR12280270	PSC	PSC_02
SRR12280500	PSC	PSC_02
SRR13269161	PSC	PSC_02

SRR13269220	PSC	PSC_02
SRR15283708	PSC	PSC_02
SRR15283747	PSC	PSC_02
SRR15283795	PSC	PSC_02
SRR15283837	PSC	PSC_02
SRR15283842	PSC	PSC_02
10317.X00179017_10317.X00179017_bin.7	PSC	PSC_03
10317.X00179093_41	PSC	PSC_03
10317.X00179186_39	PSC	PSC_03
10317.X00179300_11	PSC	PSC_03
10317.X00179651_55	PSC	PSC_03
10317.X00179725_20	PSC	PSC_03
10317.X00180243_84	PSC	PSC_03
10317.X00180334_10317.X00180334_bin.001	PSC	PSC_03
10317.X00180334_10317.X00180334_bin.1	PSC	PSC_03
10317.X00185696_11	PSC	PSC_03
363192544_S35_L001	PSC	PSC_03
363192573_S64_L001	PSC	PSC_03
363192581_S72_L001	PSC	PSC_03
363193001_S108_L001	PSC	PSC_03
363193002_S105_L001	PSC	PSC_03
363193003_S113_L001.4	PSC	PSC_03
363193013_S123_L001	PSC	PSC_03
363193022_S130_L001.1	PSC	PSC_03
363193036_S146_L001	PSC	PSC_03
363193043_S153_L001	PSC	PSC_03
363193757_S8_L001	PSC	PSC_03
363197054_S337_L001.2	PSC	PSC_03
BackhedF_2015__SID203_12M__bin.9	PSC	PSC_03
BackhedF_2015__SID582_12M__bin.6	PSC	PSC_03
BackhedF_2015__SID591_4M__bin.17	PSC	PSC_03
GCA_000297735.1	PSC	PSC_03
GCA_000598845.1	PSC	PSC_03
GCA_000601115.1	PSC	PSC_03
GCA_000724795.2	PSC	PSC_03
GCA_014042455.1	PSC	PSC_03
GCA_015553495.1	PSC	PSC_03
GCA_015669295.1	PSC	PSC_03

GCA_019013355.1	PSC	PSC_03
GCA_020297545.1	PSC	PSC_03
GCA_020297585.1	PSC	PSC_03
GCA_020558655.1	PSC	PSC_03
GCA_022714305.1	PSC	PSC_03
GCA_022722555.1	PSC	PSC_03
GCA_022782465.1	PSC	PSC_03
GCA_022832325.1	PSC	PSC_03
GCA_022832355.1	PSC	PSC_03
GCA_902374115.1	PSC	PSC_03
GeversD_2014__SKSTL0001__bin.2	PSC	PSC_03
HeQ_2017__RSZAXPI003078-111__bin.2	PSC	PSC_03
HeQ_2017__RSZAXPI003098-132__bin.12	PSC	PSC_03
KosticAD_2015__PRJNA231909.3112348__bin.8	PSC	PSC_03
LeChatelierE_2013__MH0068__bin.12	PSC	PSC_03
LiJ_2014__V1.FI21__bin.42	PSC	PSC_03
MCT420_50_S58_L002	PSC	PSC_03
MCT420_54_S62_L002	PSC	PSC_03
MCT420_58_S66_L002	PSC	PSC_03
MCT420_75_S83_L002	PSC	PSC_03
MCT420_8_S16_L002	PSC	PSC_03
NielsenHB_2014__O2_UC34_2__bin.41	PSC	PSC_03
Obregon-TitoAJ_2015__NO05__bin.48	PSC	PSC_03
SRR12280314	PSC	PSC_03
SRR12280649	PSC	PSC_03
SRR13269150	PSC	PSC_03
SRR13269158	PSC	PSC_03
SRR13269159	PSC	PSC_03
SRR13269165	PSC	PSC_03
SRR13269219	PSC	PSC_03
SRR13269223	PSC	PSC_03
SRR15283700	PSC	PSC_03
SRR15283722	PSC	PSC_03
SRR15283724	PSC	PSC_03
SRR15283728	PSC	PSC_03
SRR15283762	PSC	PSC_03
SRR15283771	PSC	PSC_03
SRR15283871	PSC	PSC_03

VatanenT_2016__G78716__bin.3	PSC	PSC_03
VatanenT_2016__G78910__bin.4	PSC	PSC_03
VatanenT_2016__G80297__bin.3	PSC	PSC_03
VatanenT_2016__G80469__bin.5	PSC	PSC_03
VincentC_2016__MM032.3__bin.19	PSC	PSC_03
VogtmannE_2016__MMRS77321055ST-27-0-0__bin.39	PSC	PSC_03
ZeeviD_2015__PNP_Main_174__bin.22	PSC	PSC_03
ZeeviD_2015__PNP_Validation_92__bin.15	PSC	PSC_03
10317.X00179025_65	PSC	PSC_04
10317.X00179381_10317.X00179381_bin.18	PSC	PSC_04
10317.X00179805_8	PSC	PSC_04
10317.X00185507_10317.X00185507_bin.17	PSC	PSC_04
10317.X00214097_10317.X00214097_bin.3	PSC	PSC_04
10317.X00214299_94	PSC	PSC_04
363192601_S92_L001	PSC	PSC_04
363193040_S150_L001	PSC	PSC_04
363193046_S156_L001	PSC	PSC_04
363193054_S164_L001	PSC	PSC_04
363193068_S178_L001	PSC	PSC_04
BackhedF_2015__SID274_4M__bin.2	PSC	PSC_04
GCA_000599065.2	PSC	PSC_04
GCA_003439675.1	PSC	PSC_04
GCA_003469305.1	PSC	PSC_04
GCA_004798445.1	PSC	PSC_04
GCA_004798525.1	PSC	PSC_04
GCA_008829725.1	PSC	PSC_04
GCA_014042525.1	PSC	PSC_04
GCA_014893755.1	PSC	PSC_04
GCA_015556305.1	PSC	PSC_04
GCA_019915805.1	PSC	PSC_04
GCA_021633405.1	PSC	PSC_04
GCA_022725225.1	PSC	PSC_04
GCA_022733395.1	PSC	PSC_04
GCA_022734965.1	PSC	PSC_04
GCA_022744835.1	PSC	PSC_04
GCA_022745625.1	PSC	PSC_04
GCA_022746345.1	PSC	PSC_04
GCA_022746405.1	PSC	PSC_04

GCA_900624785.1	PSC	PSC_04
HeQ_2017__RSZAXPI003062-102__bin.4	PSC	PSC_04
LeChatelierE_2013__MH0193__bin.44	PSC	PSC_04
NielsenHB_2014__V1_CD6_4__bin.58	PSC	PSC_04
SRR12280501	PSC	PSC_04
SRR12280643	PSC	PSC_04
SRR15283695	PSC	PSC_04
SRR15283738	PSC	PSC_04
SRR15283844	PSC	PSC_04
SRR15283864	PSC	PSC_04
SRR15283896	PSC	PSC_04
SRR9206393	PSC	PSC_04
SchirmerM_2016__G88708__bin.4	PSC	PSC_04
VatanenT_2016__G78646__bin.3	PSC	PSC_04
VatanenT_2016__G78739__bin.3	PSC	PSC_04
VatanenT_2016__G78794__bin.3	PSC	PSC_04
VatanenT_2016__G78795__bin.5	PSC	PSC_04
VatanenT_2016__G80584__bin.1	PSC	PSC_04
VincentC_2016__MM022.2__bin.34	PSC	PSC_04
WenC_2017__N016__bin.21	PSC	PSC_04
XieH_2016__YSZC12003_37879__bin.30	PSC	PSC_04
YuJ_2015__SZAXPI003422-11__bin.33	PSC	PSC_04
YuJ_2015__SZAXPI003435-11__bin.64	PSC	PSC_04
YuJ_2015__SZAXPI015250-39__bin.22	PSC	PSC_04
10317.X00179153_19	PSC	PSC_05
10317.X00179736_10317.X00179736_bin.20	PSC	PSC_05
10317.X00180154_10317.X00180154_bin.17	PSC	PSC_05
10317.X00185732_3	PSC	PSC_05
363192524_S22_L001.1	PSC	PSC_05
363192559_S50_L001	PSC	PSC_05
363192564_S55_L001	PSC	PSC_05
363192568_S59_L001	PSC	PSC_05
363192591_S82_L001	PSC	PSC_05
363192795_S100_L001	PSC	PSC_05
363193015_S125_L001.1	PSC	PSC_05
363193039_S149_L001	PSC	PSC_05
363193061_S171_L001	PSC	PSC_05
CM_madagascar__A39_02_1FE__bin.19	PSC	PSC_05

DavidLA_2015__LD-Run1-08__bin.7	PSC	PSC_05
GCA_000598725.1	PSC	PSC_05
GCA_001058775.1	PSC	PSC_05
GCA_003472195.1	PSC	PSC_05
GCA_009024655.1	PSC	PSC_05
GCA_009025705.1	PSC	PSC_05
GCA_012844105.1	PSC	PSC_05
GCA_013867505.1	PSC	PSC_05
GCA_014042405.1	PSC	PSC_05
GCA_022733815.1	PSC	PSC_05
GCA_024007805.1	PSC	PSC_05
HMP_2012__SRS023346__bin.6	PSC	PSC_05
KarlssonFH_2013__S248__bin.36	PSC	PSC_05
MCT420_63_S71_L002	PSC	PSC_05
MCT420_76_S84_L002	PSC	PSC_05
NielsenHB_2014__V1_CD21_4__bin.5	PSC	PSC_05
QinJ_2012__DLF009__bin.9	PSC	PSC_05
QinN_2014__LD-34__bin.5	PSC	PSC_05
QinN_2014__LD-35__bin.21	PSC	PSC_05
SRR12280503	PSC	PSC_05
SRR12280591	PSC	PSC_05
SRR12280635	PSC	PSC_05
SRR12280650	PSC	PSC_05
SRR13269148	PSC	PSC_05
SRR13269224	PSC	PSC_05
SRR15283685	PSC	PSC_05
SRR15283766	PSC	PSC_05
SRR15283822	PSC	PSC_05
VatanenT_2016__G80507__bin.5	PSC	PSC_05
VatanenT_2016__G80590__bin.4	PSC	PSC_05
VatanenT_2016__G80602__bin.2	PSC	PSC_05
XieH_2016__YSZC12003_35524__bin.22	PSC	PSC_05
XieH_2016__YSZC12003_37880__bin.18	PSC	PSC_05
ZeeviD_2015__PNP_Main_319__bin.2	PSC	PSC_05
10317.X00179274_24	PSC	PSC_06
10317.X00179278_57	PSC	PSC_06
10317.X00179564_10317.X00179564_bin.11	PSC	PSC_06
10317.X00179680_33	PSC	PSC_06

10317.X00179954_75	PSC	PSC_06
10317.X00180160_15	PSC	PSC_06
10317.X00185925_50	PSC	PSC_06
363192552_S43_L001	PSC	PSC_06
363192797_S102_L001	PSC	PSC_06
363193059_S169_L001	PSC	PSC_06
363193072_S181_L001	PSC	PSC_06
363193078_S188_L001	PSC	PSC_06
363193754_S16_L001.2	PSC	PSC_06
BackhedF_2015__SID10_12M__bin.4	PSC	PSC_06
BackhedF_2015__SID566_12M__bin.34	PSC	PSC_06
GCA_000598225.1	PSC	PSC_06
GCA_000598245.1	PSC	PSC_06
GCA_000599125.1	PSC	PSC_06
GCA_000599185.2	PSC	PSC_06
GCA_000601075.2	PSC	PSC_06
GCA_001056335.1	PSC	PSC_06
GCA_002811035.1	PSC	PSC_06
GCA_003437815.1	PSC	PSC_06
GCA_003472025.1	PSC	PSC_06
GCA_007896595.1	PSC	PSC_06
GCA_014042415.1	PSC	PSC_06
GCA_015548595.1	PSC	PSC_06
GCA_015553295.1	PSC	PSC_06
GCA_020539125.1	PSC	PSC_06
MCT420_49_S57_L002	PSC	PSC_06
QinN_2014__LD-50__bin.33	PSC	PSC_06
SRR12280496	PSC	PSC_06
SRR13269149	PSC	PSC_06
SRR13269156	PSC	PSC_06
SRR13269163	PSC	PSC_06
SRR13269166	PSC	PSC_06
SRR15283707	PSC	PSC_06
SRR15283750	PSC	PSC_06
SRR15283828	PSC	PSC_06
SRR15283850	PSC	PSC_06
SRR15283869	PSC	PSC_06
SRR15283876	PSC	PSC_06

10317.X00179298_6	PSC	PSC_07
10317.X00179673_25	PSC	PSC_07
10317.X00214096_10317.X00214096_bin.002	PSC	PSC_07
363192527_S10_L001	PSC	PSC_07
363192542_S33_L001.3	PSC	PSC_07
363192560_S51_L001	PSC	PSC_07
363192603_S94_L001	PSC	PSC_07
363192805_S110_L001	PSC	PSC_07
363193004_S114_L001	PSC	PSC_07
363193029_S139_L001	PSC	PSC_07
363193048_S163_L001	PSC	PSC_07
363193069_S179_L001	PSC	PSC_07
GCA_000598365.1	PSC	PSC_07
GCA_003439505.1	PSC	PSC_07
GCA_015548495.1	PSC	PSC_07
GCA_018292185.1	PSC	PSC_07
GCA_022735135.1	PSC	PSC_07
HeQ_2017__SZAXPI029574-89__bin.37	PSC	PSC_07
MCT420_28_S36_L002	PSC	PSC_07
MCT420_29_S37_L002	PSC	PSC_07
NielsenHB_2014__V1_UC15_0__bin.1	PSC	PSC_07
NielsenHB_2014__V1_UC15_3__bin.64	PSC	PSC_07
SRR12280492	PSC	PSC_07
SRR12280561	PSC	PSC_07
SRR12280645	PSC	PSC_07
SRR15283834	PSC	PSC_07
10317.X00179327_33	PSC	PSC_08
10317.X00185752_7	PSC	PSC_08
363192577_S68_L001	PSC	PSC_08
363192579_S70_L001	PSC	PSC_08
363192585_S76_L001	PSC	PSC_08
363193047_S157_L001	PSC	PSC_08
363193063_S173_L001.2	PSC	PSC_08
BackhedF_2015__SID130_12M__bin.13	PSC	PSC_08
BackhedF_2015__SID130_4M__bin.3	PSC	PSC_08
BackhedF_2015__SID615_4M__bin.5	PSC	PSC_08
GCA_000218345.1	PSC	PSC_08
GCA_000598545.2	PSC	PSC_08

GCA_000601095.1	PSC	PSC_08
GCA_001699865.1	PSC	PSC_08
GCA_002811085.1	PSC	PSC_08
GCA_003436285.1	PSC	PSC_08
GCA_003458215.1	PSC	PSC_08
GCA_003475385.1	PSC	PSC_08
GCA_008369705.1	PSC	PSC_08
GCA_019130425.1	PSC	PSC_08
GCA_023702735.1	PSC	PSC_08
LiJ_2017__H3M515950__bin.24	PSC	PSC_08
LiSS_2016__FAT_015-22-42-0__bin.55	PSC	PSC_08
MCT420_55_S63_L002	PSC	PSC_08
QinN_2014__LD-33__bin.18	PSC	PSC_08
SRR12280583	PSC	PSC_08
SRR12280608	PSC	PSC_08
SRR12280646	PSC	PSC_08
SRR13269167	PSC	PSC_08
SRR13269221	PSC	PSC_08
SRR15283706	PSC	PSC_08
SRR15283731	PSC	PSC_08
SRR15283839	PSC	PSC_08
SRR15283861	PSC	PSC_08
SRR15283868	PSC	PSC_08
SRR15283894	PSC	PSC_08
VatanenT_2016__G80556__bin.10	PSC	PSC_08
VogtmannE_2016__MMRS62192467ST-27-0-0__bin.44	PSC	PSC_08
WenC_2017__N281__bin.22	PSC	PSC_08
10317.X00179407_24	PSC	PSC_09
10317.X00179487_25	PSC	PSC_09
10317.X00179729_16	PSC	PSC_09
10317.X00215088_24	PSC	PSC_09
363192999_S107_L001	PSC	PSC_09
363193049_S158_L001	PSC	PSC_09
363193064_S174_L001	PSC	PSC_09
363193076_S185_L001	PSC	PSC_09
363197065_S315_L001	PSC	PSC_09
BackhedF_2015__SID137_12M__bin.25	PSC	PSC_09
BackhedF_2015__SID137_4M__bin.2	PSC	PSC_09

BackhedF_2015__SID137_M__bin.72	PSC	PSC_09
BackhedF_2015__SID526_4M__bin.6	PSC	PSC_09
BackhedF_2015__SID565_4M__bin.10	PSC	PSC_09
BackhedF_2015__SID626_4M__bin.17	PSC	PSC_09
GCA_000598705.1	PSC	PSC_09
GCA_000598865.1	PSC	PSC_09
GCA_001816225.2	PSC	PSC_09
GCA_008086695.1	PSC	PSC_09
GCA_020026255.1	PSC	PSC_09
GCA_020257125.1	PSC	PSC_09
GCA_020558865.1	PSC	PSC_09
GCA_022734035.1	PSC	PSC_09
KosticAD_2015__PRJNA231909.3108874__bin.9	PSC	PSC_09
LiJ_2017__H1M213648__bin.18	PSC	PSC_09
MCT420_32_S40_L002	PSC	PSC_09
QinJ_2012__NLM028__bin.25	PSC	PSC_09
QinN_2014__LD-26__bin.50	PSC	PSC_09
SRR12280315	PSC	PSC_09
SRR13269209	PSC	PSC_09
SRR15283688	PSC	PSC_09
SRR15283697	PSC	PSC_09
SRR15283720	PSC	PSC_09
SRR15283749	PSC	PSC_09
SRR15283826	PSC	PSC_09
SRR15283873	PSC	PSC_09
VatanenT_2016__G78592__bin.3	PSC	PSC_09
VatanenT_2016__G80601__bin.2	PSC	PSC_09
10317.X00180216_72	PSC	PSC_10
363192583_S74_L001	PSC	PSC_10
BackhedF_2015__SID229_B__bin.3	PSC	PSC_10
GCA_022737435.1	PSC	PSC_10
SRR12280493	PSC	PSC_10
SRR13269157	PSC	PSC_10
SRR13269204	PSC	PSC_10
SRR15283691	PSC	PSC_10
SRR15283757	PSC	PSC_10
XieH_2016__YSZC12003_36052__bin.34	PSC	PSC_10
10317.X00185882_3	PSC	PSC_11

BackhedF_2015__SID238_12M__bin.7	PSC	PSC_11
BackhedF_2015__SID238_B__bin.1	PSC	PSC_11
BackhedF_2015__SID275_B__bin.6	PSC	PSC_11
BackhedF_2015__SID275_M__bin.26	PSC	PSC_11
BackhedF_2015__SID635_B__bin.2	PSC	PSC_11
GCA_014042445.1	PSC	PSC_11
GCA_016889925.1	PSC	PSC_11
MCT420_52_S60_L002	PSC	PSC_11
SRR13269155	PSC	PSC_11
SRR13269178	PSC	PSC_11
SRR13269194	PSC	PSC_11
SRR15283752	PSC	PSC_11
SRR15283854	PSC	PSC_11
SRR15283891	PSC	PSC_11
SRR15283892	PSC	PSC_11
VogtmannE_2016__MMRS68910755ST-2	PSC	PSC_11
GCA_003438895.1	PSC	PSC_12
GCA_022832315.1	PSC	PSC_12
GCA_018786025.1	PSC	PSC_13
GCA_019733435.1	PSC	PSC_13
10317.X00178933_38	PSD	PSD_01
10317.X00179327_33	PSD	PSD_01
363192522_S5_L001	PSD	PSD_01
363192577_S68_L001	PSD	PSD_01
363192585_S76_L001	PSD	PSD_01
363193040_S150_L001	PSD	PSD_01
363193049_S158_L001	PSD	PSD_01
363193064_S174_L001	PSD	PSD_01
363193076_S185_L001	PSD	PSD_01
BackhedF_2015__SID137_12M__bin.25	PSD	PSD_01
BackhedF_2015__SID7_M__bin.18	PSD	PSD_01
GCA_020257125.1	PSD	PSD_01
HeQ_2017__SZAXPI029475-44__bin.35	PSD	PSD_01
MCT420_35_S43_L002	PSD	PSD_01
QinJ_2012__NLM028__bin.25	PSD	PSD_01
SRR12280643	PSD	PSD_01
SRR13269209	PSD	PSD_01
SRR15283688	PSD	PSD_01

SRR15283746	PSD	PSD_01
SRR15283848	PSD	PSD_01
SRR15283867	PSD	PSD_01
VatanenT_2016__G78646__bin.3	PSD	PSD_01
VatanenT_2016__G78794__bin.3	PSD	PSD_01
VatanenT_2016__G78795__bin.5	PSD	PSD_01
VatanenT_2016__G80532__bin.12	PSD	PSD_01
WenC_2017__N281__bin.22	PSD	PSD_01
YuJ_2015__SZAXPI003422-11__bin.33	PSD	PSD_01
10317.X00178995_62	PSD	PSD_02
10317.X00179805_8	PSD	PSD_02
10317.X00214097_10317.X00214097_b	PSD	PSD_02
363192533_S31_L001	PSD	PSD_02
363192566_S57_L001	PSD	PSD_02
363193032_S142_L001	PSD	PSD_02
BackhedF_2015__SID78_4M__bin.8	PSD	PSD_02
BritoIL_2016__M1.10.ST__bin.49	PSD	PSD_02
ChengpingW_2017__AS123raw__bin.19	PSD	PSD_02
GCA_000598985.1	PSD	PSD_02
GCA_000599365.1	PSD	PSD_02
GCA_004167855.1	PSD	PSD_02
GCA_014893755.1	PSD	PSD_02
GCA_022727875.1	PSD	PSD_02
LeChatelierE_2013__MH0193__bin.44	PSD	PSD_02
LiJ_2014__V1.CD3-0-PN__bin.42	PSD	PSD_02
SRR13269154	PSD	PSD_02
SRR15283753	PSD	PSD_02
VatanenT_2016__G80299__bin.1	PSD	PSD_02
YuJ_2015__SZAXPI003435-11__bin.64	PSD	PSD_02
10317.X00179017_10317.X00179017_b	PSD	PSD_03
10317.X00179156_10	PSD	PSD_03
10317.X00179225_54	PSD	PSD_03
10317.X00185752_7	PSD	PSD_03
10317.X00214153_64	PSD	PSD_03
363191840_S199_L001.1	PSD	PSD_03
363192525_S32_L001.2	PSD	PSD_03
363192544_S35_L001	PSD	PSD_03
363192573_S64_L001	PSD	PSD_03

363192581_S72_L001	PSD	PSD_03
363192595_S86_L001	PSD	PSD_03
363192795_S100_L001	PSD	PSD_03
363193001_S108_L001	PSD	PSD_03
363193022_S130_L001.1	PSD	PSD_03
363193036_S146_L001	PSD	PSD_03
363193757_S8_L001	PSD	PSD_03
363197054_S337_L001.2	PSD	PSD_03
BackhedF_2015__SID203_12M__bin.9	PSD	PSD_03
BackhedF_2015__SID338_B__bin.1	PSD	PSD_03
BackhedF_2015__SID587_12M__bin.17	PSD	PSD_03
BackhedF_2015__SID615_12M__bin.2	PSD	PSD_03
BackhedF_2015__SID615_4M__bin.5	PSD	PSD_03
GCA_000598545.2	PSD	PSD_03
GCA_000598665.1	PSD	PSD_03
GCA_000601055.1	PSD	PSD_03
GCA_000601095.1	PSD	PSD_03
GCA_015558985.1	PSD	PSD_03
GCA_015831765.1	PSD	PSD_03
GCA_020297585.1	PSD	PSD_03
GCA_022722555.1	PSD	PSD_03
HeQ_2017__RSZAXPI003078-111__bin.	PSD	PSD_03
MCT420_2_S10_L002	PSD	PSD_03
MCT420_8_S16_L002	PSD	PSD_03
NielsenHB_2014__V1_CD21_4__bin.5	PSD	PSD_03
QinN_2014__LD-93__bin.8	PSD	PSD_03
SRR12280504	PSD	PSD_03
SRR12280572	PSD	PSD_03
SRR12280583	PSD	PSD_03
SRR12280591	PSD	PSD_03
SRR12280608	PSD	PSD_03
SRR13269148	PSD	PSD_03
SRR13269167	PSD	PSD_03
SRR13269224	PSD	PSD_03
SRR15283731	PSD	PSD_03
SRR15283838	PSD	PSD_03
WenC_2017__N016__bin.21	PSD	PSD_03
XieH_2016__YSZC12003_37269__bin.2	PSD	PSD_03

10317.X00179025_65	PSI	PSD_04
10317.X00214096_10317.X00214096_b	PSI	PSD_04
363192542_S33_L001.3	PSI	PSD_04
363192560_S51_L001	PSI	PSD_04
363193015_S125_L001.1	PSI	PSD_04
BackhedF_2015__SID1_4M__bin.8	PSI	PSD_04
BackhedF_2015__SID567_12M__bin.4	PSI	PSD_04
ChengpingW_2017__AS5raw__bin.14	PSI	PSD_04
GCA_003439505.1	PSI	PSD_04
GCA_003463555.1	PSI	PSD_04
GCA_015548495.1	PSI	PSD_04
GCA_022735135.1	PSI	PSD_04
GCA_024007805.1	PSI	PSD_04
KosticAD_2015__PRJNA231909.311383	PSI	PSD_04
KosticAD_2015__PRJNA231909.311383	PSI	PSD_04
MCT420_41_S49_L002	PSI	PSD_04
MCT420_55_S63_L002	PSI	PSD_04
MCT420_81_S89_L002	PSI	PSD_04
NielsenHB_2014__V1_UC15_3__bin.64	PSI	PSD_04
SRR12280270	PSI	PSD_04
SRR12280492	PSI	PSD_04
SRR12280561	PSI	PSD_04
SRR12280645	PSI	PSD_04
VatanenT_2016__G80556__bin.10	PSI	PSD_04
XieH_2016__YSZC12003_37207R1__bir	PSI	PSD_04
10317.X00179043_48	PSI	PSD_05
363193035_S145_L001	PSI	PSD_05
GCA_000598585.1	PSI	PSD_05
GCA_002811085.1	PSI	PSD_05
GCA_014042505.1	PSI	PSD_05
GCA_015553755.1	PSI	PSD_05
MCT420_18_S26_L002	PSI	PSD_05
SRR12280417	PSI	PSD_05
SRR13269155	PSI	PSD_05
SRR15283752	PSI	PSD_05
SRR15283758	PSI	PSD_05
VatanenT_2016__G78526__bin.12	PSI	PSD_05
10317.X00179093_41	PSI	PSD_06

10317.X00179186_39	PSI	PSD_06
10317.X00179725_20	PSI	PSD_06
10317.X00179954_75	PSI	PSD_06
GCA_000297735.1	PSI	PSD_06
GCA_000598185.2	PSI	PSD_06
GCA_000598845.1	PSI	PSD_06
GCA_001699875.1	PSI	PSD_06
GCA_015669295.1	PSI	PSD_06
GCA_019733435.1	PSI	PSD_06
GCA_022832315.1	PSI	PSD_06
MCT420_75_S83_L002	PSI	PSD_06
SRR12280650	PSI	PSD_06
SRR13269150	PSI	PSD_06
SRR13269166	PSI	PSD_06
SRR15283750	PSI	PSD_06
SRR15283767	PSI	PSD_06
10317.X00179298_6	PSI	PSD_07
10317.X00179673_25	PSI	PSD_07
363192603_S94_L001	PSI	PSD_07
363192805_S110_L001	PSI	PSD_07
363192998_S106_L001	PSI	PSD_07
363193028_S138_L001	PSI	PSD_07
363193029_S139_L001	PSI	PSD_07
363193048_S163_L001	PSI	PSD_07
363193069_S179_L001	PSI	PSD_07
BackhedF_2015__SID274_4M__bin.2	PSI	PSD_07
BackhedF_2015__SID635_B__bin.2	PSI	PSD_07
GCA_000210835.1	PSI	PSD_07
GCA_004798445.1	PSI	PSD_07
GCA_004798525.1	PSI	PSD_07
GCA_018292185.1	PSI	PSD_07
GCA_018369215.1	PSI	PSD_07
GCA_019915805.1	PSI	PSD_07
GCA_022744835.1	PSI	PSD_07
MCT420_28_S36_L002	PSI	PSD_07
SRR12280549	PSI	PSD_07
SRR12280658	PSI	PSD_07
SRR13269222	PSI	PSD_07

SRR15283724	PSD	PSD_07
SRR15283834	PSD	PSD_07
SRR15283871	PSD	PSD_07
VatanenT_2016__G78739__bin.3	PSD	PSD_07
VatanenT_2016__G80458__bin.3	PSD	PSD_07
10317.X00179306_29	PSD	PSD_08
10317.X00215482_24	PSD	PSD_08
363192528_S11_L001	PSD	PSD_08
363192575_S66_L001.1	PSD	PSD_08
363192579_S70_L001	PSD	PSD_08
363192583_S74_L001	PSD	PSD_08
363192589_S80_L001	PSD	PSD_08
363192793_S98_L001	PSD	PSD_08
363193054_S164_L001	PSD	PSD_08
363193060_S170_L001	PSD	PSD_08
BackhedF_2015__SID130_12M__bin.13	PSD	PSD_08
GCA_000599065.2	PSD	PSD_08
GCA_000599325.1	PSD	PSD_08
GCA_001286525.1	PSD	PSD_08
GCA_001699865.1	PSD	PSD_08
GCA_003439675.1	PSD	PSD_08
GCA_003458215.1	PSD	PSD_08
GCA_008569345.1	PSD	PSD_08
GCA_014042465.1	PSD	PSD_08
GCA_015551365.1	PSD	PSD_08
GCA_015669435.1	PSD	PSD_08
GCA_019041875.1	PSD	PSD_08
GCA_020297435.1	PSD	PSD_08
GCA_023702735.1	PSD	PSD_08
GCA_905188165.1	PSD	PSD_08
HeQ_2017__RSZAXPI003062-102__bin.	PSD	PSD_08
LiJ_2017__H3M515950__bin.24	PSD	PSD_08
MCT420_36_S44_L002	PSD	PSD_08
MCT420_7_S15_L002	PSD	PSD_08
MCT420_80_S88_L002	PSD	PSD_08
NielsenHB_2014__V1_CD6_4__bin.58	PSD	PSD_08
SRR12280493	PSD	PSD_08
SRR12280589	PSD	PSD_08

SRR12280651	PSI	PSD_08
SRR13269122	PSI	PSD_08
SRR13269157	PSI	PSD_08
SRR13269221	PSI	PSD_08
SRR15283691	PSI	PSD_08
SRR15283701	PSI	PSD_08
SRR15283742	PSI	PSD_08
SRR15283766	PSI	PSD_08
SRR15283839	PSI	PSD_08
SRR15283861	PSI	PSD_08
SRR15283873	PSI	PSD_08
SRR15283894	PSI	PSD_08
VogtmannE_2016__MMRS72980899ST-2	PSI	PSD_08
XieH_2016__YSZC12003_35524__bin.2	PSI	PSD_08
XieH_2016__YSZC12003_36052__bin.3	PSI	PSD_08
XieH_2016__YSZC12003_37205__bin.2	PSI	PSD_08
XieH_2016__YSZC12003_37879__bin.3	PSI	PSD_08
10317.X00179487_25	PSI	PSD_09
363192555_S46_L001	PSI	PSD_09
363192563_S54_L001	PSI	PSD_09
BackhedF_2015__SID10_12M__bin.4	PSI	PSD_09
BackhedF_2015__SID566_12M__bin.34	PSI	PSD_09
CM_madagascar__A39_02_1FE__bin.19	PSI	PSD_09
GCA_003458955.1	PSI	PSD_09
GCA_003464385.1	PSI	PSD_09
GCA_003472195.1	PSI	PSD_09
MCT420_6_S14_L002	PSI	PSD_09
SRR12280522	PSI	PSD_09
SRR13269220	PSI	PSD_09
SRR13269245	PSI	PSD_09
SRR15283738	PSI	PSD_09
SRR15283844	PSI	PSD_09
10317.X00179564_10317.X00179564_b	PSI	PSD_10
10317.X00182267_21	PSI	PSD_10
10317.X00185745_38	PSI	PSD_10
363192541_S20_L001	PSI	PSD_10
363192601_S92_L001	PSI	PSD_10
363192797_S102_L001	PSI	PSD_10

363193002_S105_L001	PSI	PSD_10
363193013_S123_L001	PSI	PSD_10
363193037_S147_L001	PSI	PSD_10
363193059_S169_L001	PSI	PSD_10
363193061_S171_L001	PSI	PSD_10
363193068_S178_L001	PSI	PSD_10
363193072_S181_L001	PSI	PSD_10
363193078_S188_L001	PSI	PSD_10
GCA_000273155.1	PSI	PSD_10
GCA_000601115.1	PSI	PSD_10
GCA_001056335.1	PSI	PSD_10
GCA_001682215.1	PSI	PSD_10
GCA_003437815.1	PSI	PSD_10
GCA_003472025.1	PSI	PSD_10
GCA_007896595.1	PSI	PSD_10
GCA_009025705.1	PSI	PSD_10
GCA_012844105.1	PSI	PSD_10
GCA_014042415.1	PSI	PSD_10
GCA_015548595.1	PSI	PSD_10
GCA_015553295.1	PSI	PSD_10
GCA_020297545.1	PSI	PSD_10
GCA_020539125.1	PSI	PSD_10
GCA_022733095.1	PSI	PSD_10
GCA_022745295.1	PSI	PSD_10
GCA_902374115.1	PSI	PSD_10
LiJ_2014__MH0221__bin.8	PSI	PSD_10
MCT420_10_S18_L002	PSI	PSD_10
MCT420_54_S62_L002	PSI	PSD_10
SRR13269149	PSI	PSD_10
SRR13269156	PSI	PSD_10
SRR13269158	PSI	PSD_10
SRR13269163	PSI	PSD_10
SRR13269165	PSI	PSD_10
SRR13269219	PSI	PSD_10
SRR13269223	PSI	PSD_10
SRR15283707	PSI	PSD_10
SRR15283713	PSI	PSD_10
SRR15283728	PSI	PSD_10

SRR15283828	PSD	PSD_10
SRR15283850	PSD	PSD_10
SRR15283864	PSD	PSD_10
SRR15283869	PSD	PSD_10
VatanenT_2016__G80584__bin.1	PSD	PSD_10
VincentC_2016__MM022.2__bin.34	PSD	PSD_10
VincentC_2016__MM060.2__bin.1	PSD	PSD_10
ZeeviD_2015__PNP_Main_319__bin.2	PSD	PSD_10
10317.X00180334_10317.X00180334_b	PSD	PSD_11
10317.X00180334_10317.X00180334_b	PSD	PSD_11
363193043_S153_L001	PSD	PSD_11
GCA_022782465.1	PSD	PSD_11
LeChatelierE_2013__MH0068__bin.12	PSD	PSD_11
MCT420_50_S58_L002	PSD	PSD_11
SRR12280649	PSD	PSD_11
SRR13269159	PSD	PSD_11
10317.X00185507_10317.X00185507_b	PSD	PSD_12
GCA_014042445.1	PSD	PSD_12
GCA_014042525.1	PSD	PSD_12
GCA_016889925.1	PSD	PSD_12
SRR12280501	PSD	PSD_12
SRR15283854	PSD	PSD_12
SRR15283892	PSD	PSD_12
SRR15283896	PSD	PSD_12
VogtmannE_2016__MMRS68910755ST-2	PSD	PSD_12
10317.X00215241_67	PSD	PSD_13
GCA_000598565.1	PSD	PSD_13
GCA_000599165.2	PSD	PSD_13
HMP_2012__SRS075398__bin.15	PSD	PSD_13
MCT420_49_S57_L002	PSD	PSD_13
363192559_S50_L001	PSD	PSD_14
363192591_S82_L001	PSD	PSD_14
GCA_000598645.1	PSD	PSD_14
GCA_000724795.2	PSD	PSD_14
GCA_015553495.1	PSD	PSD_14
GCA_022832325.1	PSD	PSD_14
GCA_022832355.1	PSD	PSD_14
GCA_900624785.1	PSD	PSD_14

MCT420_58_S66_L002	PSD	PSD_14
MCT420_63_S71_L002	PSD	PSD_14
SRR12280314	PSD	PSD_14
SRR12280635	PSD	PSD_14
SRR15283700	PSD	PSD_14
SRR15283722	PSD	PSD_14
SRR15283762	PSD	PSD_14
SRR15283771	PSD	PSD_14
SRR15283826	PSD	PSD_14
363192568_S59_L001	PSD	PSD_15
363192803_S112_L001.2	PSD	PSD_16
363193046_S156_L001	PSD	PSD_16
363193754_S16_L001.2	PSD	PSD_16
GCA_000599125.1	PSD	PSD_16
GCA_000601075.2	PSD	PSD_16
GCA_002811035.1	PSD	PSD_16
GCA_003438895.1	PSD	PSD_16
HMP_2012__SRS018817__bin.4	PSD	PSD_16
SRR15283876	PSD	PSD_16
BackhedF_2015__SID130_4M__bin.3	PSD	PSD_17
SRR9206393	PSD	PSD_17
SRR13269111	PSD	PSD_18
SRR15283720	PSD	PSD_18
VatanenT_2016__G78527__bin.3	PSD	PSD_18
VatanenT_2016__G78592__bin.3	PSD	PSD_18
SRR13269194	PSD	PSD_19
SRR15283749	PSD	PSD_20
SRR15283820	PSD	PSD_21
XieH_2016__YSZC12003_37880__bin.1	PSD	PSD_22
10317.X00178933_38	PSE	PSE_01
10317.X00179025_65	PSE	PSE_01
10317.X00179156_10	PSE	PSE_01
10317.X00179407_24	PSE	PSE_01
10317.X00179729_16	PSE	PSE_01
10317.X00185882_3	PSE	PSE_01
10317.X00215088_24	PSE	PSE_01
363192522_S5_L001	PSE	PSE_01
363192797_S102_L001	PSE	PSE_01

363193054_S164_L001	PSE	PSE_01
GCA_000210835.1	PSE	PSE_01
GCA_000598705.1	PSE	PSE_01
GCA_000598865.1	PSE	PSE_01
GCA_000599065.2	PSE	PSE_01
GCA_001692695.1	PSE	PSE_01
GCA_008086695.1	PSE	PSE_01
GCA_013327835.1	PSE	PSE_01
GCA_014042525.1	PSE	PSE_01
GCA_015558985.1	PSE	PSE_01
GCA_019915805.1	PSE	PSE_01
GCA_022721415.1	PSE	PSE_01
GCA_022734035.1	PSE	PSE_01
GCA_022744835.1	PSE	PSE_01
GCA_022746405.1	PSE	PSE_01
GCA_901212445.1	PSE	PSE_01
MCT420_32_S40_L002	PSE	PSE_01
MCT420_35_S43_L002	PSE	PSE_01
QinJ_2012__CON-005__bin.52	PSE	PSE_01
SRR12280315	PSE	PSE_01
SRR12280336	PSE	PSE_01
SRR12280504	PSE	PSE_01
SRR12280549	PSE	PSE_01
SRR12280643	PSE	PSE_01
SRR12280658	PSE	PSE_01
SRR13269222	PSE	PSE_01
SRR15283695	PSE	PSE_01
SRR15283757	PSE	PSE_01
SRR15283848	PSE	PSE_01
SRR15283867	PSE	PSE_01
SRR15283896	PSE	PSE_01
VatanenT_2016__G80499__bin.3	PSE	PSE_01
10317.X00178992_47	PSE	PSE_02
10317.X00179680_33	PSE	PSE_02
10317.X00179736_10317.X00179736_b	PSE	PSE_02
10317.X00185732_3	PSE	PSE_02
10317.X00185752_7	PSE	PSE_02
10317.X00214812_39	PSE	PSE_02

363192541_S20_L001	PSE	PSE_02
363192559_S50_L001	PSE	PSE_02
363192564_S55_L001	PSE	PSE_02
363192591_S82_L001	PSE	PSE_02
363192602_S93_L001	PSE	PSE_02
363192605_S96_L001	PSE	PSE_02
363192793_S98_L001	PSE	PSE_02
363193031_S141_L001.1	PSE	PSE_02
363193037_S147_L001	PSE	PSE_02
363193044_S154_L001	PSE	PSE_02
363193079_S189_L001	PSE	PSE_02
BackhedF_2015__SID172_12M__bin.1	PSE	PSE_02
BackhedF_2015__SID521_4M__bin.12	PSE	PSE_02
BackhedF_2015__SID53_4M__bin.14	PSE	PSE_02
DavidLA_2015__LD-Run1-08__bin.7	PSE	PSE_02
GCA_000273155.1	PSE	PSE_02
GCA_000598645.1	PSE	PSE_02
GCA_000599185.2	PSE	PSE_02
GCA_001058775.1	PSE	PSE_02
GCA_002811025.1	PSE	PSE_02
GCA_013867505.1	PSE	PSE_02
GCA_014042405.1	PSE	PSE_02
GCA_015553295.1	PSE	PSE_02
GCA_015560515.1	PSE	PSE_02
GCA_019733435.1	PSE	PSE_02
GCA_022733815.1	PSE	PSE_02
GCA_022780605.1	PSE	PSE_02
GCA_905188165.1	PSE	PSE_02
MCT420_10_S18_L002	PSE	PSE_02
MCT420_47_S55_L002	PSE	PSE_02
MCT420_76_S84_L002	PSE	PSE_02
MCT420_80_S88_L002	PSE	PSE_02
NielsenHB_2014__V1_UC34_0__bin.49	PSE	PSE_02
QinJ_2012__DLF009__bin.9	PSE	PSE_02
QinN_2014__LD-34__bin.5	PSE	PSE_02
QinN_2014__LD-35__bin.21	PSE	PSE_02
SRR12280583	PSE	PSE_02
SRR12280591	PSE	PSE_02

SRR12280608	PSE	PSE_02
SRR12280635	PSE	PSE_02
SRR12280651	PSE	PSE_02
SRR13269161	PSE	PSE_02
SRR13269167	PSE	PSE_02
SRR13269223	PSE	PSE_02
SRR13269224	PSE	PSE_02
SRR15283713	PSE	PSE_02
SRR15283747	PSE	PSE_02
SRR15283795	PSE	PSE_02
SRR15283873	PSE	PSE_02
VatanenT_2016__G78715__bin.6	PSE	PSE_02
VincentC_2016__MM060.2__bin.1	PSE	PSE_02
10317.X00179017_10317.X00179017_b	PSE	PSE_03
10317.X00182267_21	PSE	PSE_03
10317.X00214130_23	PSE	PSE_03
363192539_S19_L001	PSE	PSE_03
363192544_S35_L001	PSE	PSE_03
363192548_S39_L001	PSE	PSE_03
363192555_S46_L001	PSE	PSE_03
363192557_S48_L001	PSE	PSE_03
363192563_S54_L001	PSE	PSE_03
363192573_S64_L001	PSE	PSE_03
363192581_S72_L001	PSE	PSE_03
363193001_S108_L001	PSE	PSE_03
363193013_S123_L001	PSE	PSE_03
363193015_S125_L001.1	PSE	PSE_03
363193022_S130_L001.1	PSE	PSE_03
363193036_S146_L001	PSE	PSE_03
363193757_S8_L001	PSE	PSE_03
BackhedF_2015__SID133_4M__bin.23	PSE	PSE_03
BackhedF_2015__SID150_12M__bin.19	PSE	PSE_03
BackhedF_2015__SID150_M__bin.47	PSE	PSE_03
BackhedF_2015__SID203_12M__bin.9	PSE	PSE_03
BackhedF_2015__SID503_12M__bin.45	PSE	PSE_03
BackhedF_2015__SID503_4M__bin.20	PSE	PSE_03
BackhedF_2015__SID546_12M__bin.30	PSE	PSE_03
BackhedF_2015__SID546_4M__bin.5	PSE	PSE_03

BackhedF_2015__SID567_12M__bin.4	PSE	PSE_03
BackhedF_2015__SID570_4M__bin.7	PSE	PSE_03
CM_madagascar__A39_02_1FE__bin.19	PSE	PSE_03
ChengpingW_2017__AS5raw__bin.14	PSE	PSE_03
GCA_000598725.1	PSE	PSE_03
GCA_000598785.2	PSE	PSE_03
GCA_000965785.1	PSE	PSE_03
GCA_001682215.1	PSE	PSE_03
GCA_003458955.1	PSE	PSE_03
GCA_003464385.1	PSE	PSE_03
GCA_003468145.1	PSE	PSE_03
GCA_003469705.1	PSE	PSE_03
GCA_003472195.1	PSE	PSE_03
GCA_020297585.1	PSE	PSE_03
GCA_020859785.1	PSE	PSE_03
GCA_022716295.1	PSE	PSE_03
GCA_022722555.1	PSE	PSE_03
GCA_024007805.1	PSE	PSE_03
GCA_900624785.1	PSE	PSE_03
HeQ_2017__RSZAXPI003078-111__bin.	PSE	PSE_03
HeQ_2017__RSZAXPI003098-132__bin.	PSE	PSE_03
KosticAD_2015__PRJNA231909.310143	PSE	PSE_03
KosticAD_2015__PRJNA231909.311383	PSE	PSE_03
KosticAD_2015__PRJNA231909.311383	PSE	PSE_03
LeChatelierE_2013__MH0410__bin.30	PSE	PSE_03
MCT420_41_S49_L002	PSE	PSE_03
MCT420_6_S14_L002	PSE	PSE_03
MCT420_81_S89_L002	PSE	PSE_03
MCT420_86_S94_L002	PSE	PSE_03
MCT420_8_S16_L002	PSE	PSE_03
QinN_2014__LD-93__bin.8	PSE	PSE_03
SRR12280416	PSE	PSE_03
SRR12280499	PSE	PSE_03
SRR12280500	PSE	PSE_03
SRR12280501	PSE	PSE_03
SRR12280511	PSE	PSE_03
SRR12280522	PSE	PSE_03
SRR12280632	PSE	PSE_03

SRR13269162	PSE	PSE_03
SRR13269220	PSE	PSE_03
SRR13269245	PSE	PSE_03
SRR15283687	PSE	PSE_03
SRR15283708	PSE	PSE_03
SRR15283720	PSE	PSE_03
SRR15283763	PSE	PSE_03
SRR15283820	PSE	PSE_03
SRR15283837	PSE	PSE_03
SRR15283842	PSE	PSE_03
SRR15283865	PSE	PSE_03
VatanenT_2016__G78592__bin.3	PSE	PSE_03
WenC_2017__RA132__bin.29	PSE	PSE_03
XieH_2016__YSZC12003_37207R1__bir	PSE	PSE_03
10317.X00179093_41	PSE	PSE_04
10317.X00179274_24	PSE	PSE_04
10317.X00179300_11	PSE	PSE_04
10317.X00179725_20	PSE	PSE_04
10317.X00180243_84	PSE	PSE_04
363193002_S105_L001	PSE	PSE_04
363193063_S173_L001.2	PSE	PSE_04
BackhedF_2015__SID582_12M__bin.6	PSE	PSE_04
BackhedF_2015__SID591_4M__bin.17	PSE	PSE_04
GCA_000599325.1	PSE	PSE_04
GCA_000601115.1	PSE	PSE_04
GCA_000724795.2	PSE	PSE_04
GCA_003458215.1	PSE	PSE_04
GCA_004167855.1	PSE	PSE_04
GCA_008369705.1	PSE	PSE_04
GCA_015553495.1	PSE	PSE_04
GCA_015556305.1	PSE	PSE_04
GCA_020297545.1	PSE	PSE_04
GCA_020558655.1	PSE	PSE_04
GCA_022832325.1	PSE	PSE_04
GCA_022832355.1	PSE	PSE_04
GCA_902374115.1	PSE	PSE_04
GeversD_2014__SKSTL0001__bin.2	PSE	PSE_04
KosticAD_2015__PRJNA231909.311234	PSE	PSE_04

LiJ_2017__H3M515950__bin.24	PSE	PSE_04
MCT420_54_S62_L002	PSE	PSE_04
Obregon-TitoAJ_2015__NO05__bin.48	PSE	PSE_04
SRR12280314	PSE	PSE_04
SRR12280646	PSE	PSE_04
SRR12280650	PSE	PSE_04
SRR13269154	PSE	PSE_04
SRR13269158	PSE	PSE_04
SRR13269219	PSE	PSE_04
SRR15283700	PSE	PSE_04
SRR15283722	PSE	PSE_04
SRR15283728	PSE	PSE_04
SRR15283762	PSE	PSE_04
SRR15283771	PSE	PSE_04
VatanenT_2016__G78716__bin.3	PSE	PSE_04
VatanenT_2016__G80469__bin.5	PSE	PSE_04
VogtmannE_2016__MMRS77321055ST-2	PSE	PSE_04
WenC_2017__N281__bin.22	PSE	PSE_04
ZeeviD_2015__PNP_Main_174__bin.22	PSE	PSE_04
10317.X00179327_33	PSE	PSE_05
10317.X00180160_15	PSE	PSE_05
363192546_S37_L001.1	PSE	PSE_05
363192568_S59_L001	PSE	PSE_05
363192577_S68_L001	PSE	PSE_05
363192579_S70_L001	PSE	PSE_05
363192583_S74_L001	PSE	PSE_05
363192585_S76_L001	PSE	PSE_05
363193039_S149_L001	PSE	PSE_05
363193047_S157_L001	PSE	PSE_05
363193059_S169_L001	PSE	PSE_05
363193072_S181_L001	PSE	PSE_05
363193078_S188_L001	PSE	PSE_05
BackhedF_2015__SID615_4M__bin.5	PSE	PSE_05
GCA_000218345.1	PSE	PSE_05
GCA_000598225.1	PSE	PSE_05
GCA_000598285.1	PSE	PSE_05
GCA_000598545.2	PSE	PSE_05
GCA_000601095.1	PSE	PSE_05

GCA_001056335.1	PSE	PSE_05
GCA_001699865.1	PSE	PSE_05
GCA_002811085.1	PSE	PSE_05
GCA_003437815.1	PSE	PSE_05
GCA_003472025.1	PSE	PSE_05
GCA_007896595.1	PSE	PSE_05
GCA_014042415.1	PSE	PSE_05
GCA_015548595.1	PSE	PSE_05
GCA_015669435.1	PSE	PSE_05
GCA_015831765.1	PSE	PSE_05
GCA_019130425.1	PSE	PSE_05
GCA_020539125.1	PSE	PSE_05
GCA_023702735.1	PSE	PSE_05
MCT420_52_S60_L002	PSE	PSE_05
QinN_2014__LD-33__bin.18	PSE	PSE_05
SRR12280493	PSE	PSE_05
SRR12280503	PSE	PSE_05
SRR13269149	PSE	PSE_05
SRR13269156	PSE	PSE_05
SRR13269157	PSE	PSE_05
SRR13269163	PSE	PSE_05
SRR13269204	PSE	PSE_05
SRR13269221	PSE	PSE_05
SRR15283685	PSE	PSE_05
SRR15283691	PSE	PSE_05
SRR15283707	PSE	PSE_05
SRR15283731	PSE	PSE_05
SRR15283766	PSE	PSE_05
SRR15283822	PSE	PSE_05
SRR15283828	PSE	PSE_05
SRR15283839	PSE	PSE_05
SRR15283850	PSE	PSE_05
SRR15283861	PSE	PSE_05
SRR15283868	PSE	PSE_05
SRR15283869	PSE	PSE_05
SRR15283894	PSE	PSE_05
VogtmannE_2016__MMRS62192467ST-2	PSE	PSE_05
XieH_2016__YSZC12003_35524__bin.2	PSE	PSE_05

XieH_2016__YSZC12003_36052__bin.3	PSE	PSE_05
10317.X00179381_10317.X00179381_b	PSE	PSE_06
10317.X00179651_55	PSE	PSE_06
10317.X00179805_8	PSE	PSE_06
BackhedF_2015__SID10_12M__bin.4	PSE	PSE_06
BackhedF_2015__SID566_12M__bin.34	PSE	PSE_06
GCA_000598665.1	PSE	PSE_06
GCA_000598905.1	PSE	PSE_06
GCA_000601055.1	PSE	PSE_06
GCA_001699855.1	PSE	PSE_06
GCA_004798445.1	PSE	PSE_06
GCA_004798525.1	PSE	PSE_06
GCA_014042445.1	PSE	PSE_06
GCA_014893755.1	PSE	PSE_06
GCA_016889925.1	PSE	PSE_06
GCA_022725225.1	PSE	PSE_06
GCA_022745625.1	PSE	PSE_06
LeChatelierE_2013__MH0193__bin.44	PSE	PSE_06
MCT420_55_S63_L002	PSE	PSE_06
MCT420_77_S85_L002	PSE	PSE_06
SRR12280572	PSE	PSE_06
SRR13269111	PSE	PSE_06
SRR13269207	PSE	PSE_06
SRR15283697	PSE	PSE_06
SRR15283749	PSE	PSE_06
SRR15283854	PSE	PSE_06
SRR15283892	PSE	PSE_06
VogtmannE_2016__MMRS68910755ST-2	PSE	PSE_06
WenC_2017__N016__bin.21	PSE	PSE_06
YuJ_2015__SZAXPI015250-39__bin.22	PSE	PSE_06
10317.X00179673_25	PSE	PSE_07
10317.X00180334_10317.X00180334_b	PSE	PSE_07
10317.X00180334_10317.X00180334_b	PSE	PSE_07
10317.X00214299_94	PSE	PSE_07
363192552_S43_L001	PSE	PSE_07
363192805_S110_L001	PSE	PSE_07
363193029_S139_L001	PSE	PSE_07
363193043_S153_L001	PSE	PSE_07

363193048_S163_L001	PSE	PSE_07
363193069_S179_L001	PSE	PSE_07
BackhedF_2015__SID238_12M__bin.7	PSE	PSE_07
BackhedF_2015__SID238_B__bin.1	PSE	PSE_07
BackhedF_2015__SID275_B__bin.6	PSE	PSE_07
GCA_000273765.1	PSE	PSE_07
GCA_000297735.1	PSE	PSE_07
GCA_000598845.1	PSE	PSE_07
GCA_003469305.1	PSE	PSE_07
GCA_014042455.1	PSE	PSE_07
GCA_015669295.1	PSE	PSE_07
GCA_018292185.1	PSE	PSE_07
GCA_022782465.1	PSE	PSE_07
HeQ_2017__SZAXPI029574-89__bin.37	PSE	PSE_07
LeChatelierE_2013__MH0068__bin.12	PSE	PSE_07
MCT420_28_S36_L002	PSE	PSE_07
MCT420_49_S57_L002	PSE	PSE_07
MCT420_50_S58_L002	PSE	PSE_07
MCT420_75_S83_L002	PSE	PSE_07
QinN_2014__LD-50__bin.33	PSE	PSE_07
SRR12280270	PSE	PSE_07
SRR12280649	PSE	PSE_07
SRR13269150	PSE	PSE_07
SRR13269159	PSE	PSE_07
SRR13269165	PSE	PSE_07
SRR13269178	PSE	PSE_07
SRR15283724	PSE	PSE_07
SRR15283834	PSE	PSE_07
SRR9206393	PSE	PSE_07
VincentC_2016__MM032.3__bin.19	PSE	PSE_07
10317.X00179954_75	PSE	PSE_08
363192524_S22_L001.1	PSE	PSE_08
363192527_S10_L001	PSE	PSE_08
363193049_S158_L001	PSE	PSE_08
363193061_S171_L001	PSE	PSE_08
363193064_S174_L001	PSE	PSE_08
363193076_S185_L001	PSE	PSE_08
363193754_S16_L001.2	PSE	PSE_08

BackhedF_2015__SID137_12M__bin.25	PSE	PSE_08
BackhedF_2015__SID137_4M__bin.2	PSE	PSE_08
BackhedF_2015__SID137_M__bin.72	PSE	PSE_08
BackhedF_2015__SID180_4M__bin.6	PSE	PSE_08
BackhedF_2015__SID526_4M__bin.6	PSE	PSE_08
GCA_000598245.1	PSE	PSE_08
GCA_000599365.1	PSE	PSE_08
GCA_001816225.2	PSE	PSE_08
GCA_002811035.1	PSE	PSE_08
GCA_003436935.1	PSE	PSE_08
GCA_003438895.1	PSE	PSE_08
GCA_009024655.1	PSE	PSE_08
GCA_009025705.1	PSE	PSE_08
GCA_012844105.1	PSE	PSE_08
GCA_020257125.1	PSE	PSE_08
GCA_020297425.1	PSE	PSE_08
GCA_020558865.1	PSE	PSE_08
GCA_022733095.1	PSE	PSE_08
GCA_022745295.1	PSE	PSE_08
GCA_022832315.1	PSE	PSE_08
HMP_2012__SRS018817__bin.4	PSE	PSE_08
KarlssonFH_2013__S248__bin.36	PSE	PSE_08
MCT420_29_S37_L002	PSE	PSE_08
MCT420_63_S71_L002	PSE	PSE_08
QinJ_2012__NLM028__bin.25	PSE	PSE_08
QinN_2014__LD-26__bin.50	PSE	PSE_08
SRR12280496	PSE	PSE_08
SRR13269166	PSE	PSE_08
SRR13269209	PSE	PSE_08
SRR15283688	PSE	PSE_08
SRR15283750	PSE	PSE_08
SRR15283753	PSE	PSE_08
SRR15283876	PSE	PSE_08
VatanenT_2016__G80590__bin.4	PSE	PSE_08
VatanenT_2016__G80602__bin.2	PSE	PSE_08
XieH_2016__YSZC12003_37880__bin.1	PSE	PSE_08
ZeeviD_2015__PNP_Main_319__bin.2	PSE	PSE_08
ZellerG_2014__CCIS76845094ST-20-0__	PSE	PSE_08

363193035_S145_L001	PSE	PSE_09
GCA_015553755.1	PSE	PSE_09
MCT420_18_S26_L002	PSE	PSE_09
MCT420_59_S67_L002	PSE	PSE_09
10317.X00178992_47	PSF	PSF_01
10317.X00179680_33	PSF	PSF_01
10317.X00214812_39	PSF	PSF_01
363192559_S50_L001	PSF	PSF_01
363192591_S82_L001	PSF	PSF_01
363192602_S93_L001	PSF	PSF_01
363192605_S96_L001	PSF	PSF_01
363192793_S98_L001	PSF	PSF_01
363193031_S141_L001.1	PSF	PSF_01
363193044_S154_L001	PSF	PSF_01
GCA_000598645.1	PSF	PSF_01
GCA_000599185.2	PSF	PSF_01
GCA_002811025.1	PSF	PSF_01
GCA_015548825.1	PSF	PSF_01
GCA_015553295.1	PSF	PSF_01
GCA_019733435.1	PSF	PSF_01
MCT420_10_S18_L002	PSF	PSF_01
MCT420_80_S88_L002	PSF	PSF_01
NielsenHB_2014__V1_UC34_0__bin.49	PSF	PSF_01
SRR12280635	PSF	PSF_01
SRR12280651	PSF	PSF_01
SRR13269161	PSF	PSF_01
SRR15283747	PSF	PSF_01
SRR15283795	PSF	PSF_01
VatanenT_2016__G78715__bin.6	PSF	PSF_01
10317.X00178995_62	PSF	PSF_02
10317.X00179306_29	PSF	PSF_02
10317.X00180464_10317.X00180464_b	PSF	PSF_02
10317.X00215482_24	PSF	PSF_02
363192528_S11_L001	PSF	PSF_02
363192589_S80_L001	PSF	PSF_02
363193015_S125_L001.1	PSF	PSF_02
BackhedF_2015__SID567_12M__bin.4	PSF	PSF_02
BackhedF_2015__SID78_4M__bin.8	PSF	PSF_02

CM_madagascar__A39_02_1FE__bin.19	PSF	PSF_02
ChengpingW_2017__AS5raw__bin.14	PSF	PSF_02
GCA_001286525.1	PSF	PSF_02
GCA_001692695.1	PSF	PSF_02
GCA_003472195.1	PSF	PSF_02
GCA_008569345.1	PSF	PSF_02
GCA_014042465.1	PSF	PSF_02
GCA_020297435.1	PSF	PSF_02
GCA_022746755.1	PSF	PSF_02
GCA_024007805.1	PSF	PSF_02
HMP_2012__SRS023346__bin.6	PSF	PSF_02
KosticAD_2015__PRJNA231909.311383	PSF	PSF_02
KosticAD_2015__PRJNA231909.311383	PSF	PSF_02
MCT420_36_S44_L002	PSF	PSF_02
MCT420_41_S49_L002	PSF	PSF_02
MCT420_6_S14_L002	PSF	PSF_02
MCT420_86_S94_L002	PSF	PSF_02
SRR12280522	PSF	PSF_02
SRR12280589	PSF	PSF_02
SRR13269245	PSF	PSF_02
VatanenT_2016__G78583__bin.3	PSF	PSF_02
VatanenT_2016__G78605__bin.3	PSF	PSF_02
VatanenT_2016__G80299__bin.1	PSF	PSF_02
10317.X00179017_10317.X00179017_b	PSF	PSF_03
10317.X00182267_21	PSF	PSF_03
363192544_S35_L001	PSF	PSF_03
363192573_S64_L001	PSF	PSF_03
363192581_S72_L001	PSF	PSF_03
363193013_S123_L001	PSF	PSF_03
363193022_S130_L001.1	PSF	PSF_03
363193036_S146_L001	PSF	PSF_03
363193757_S8_L001	PSF	PSF_03
363197054_S337_L001.2	PSF	PSF_03
BackhedF_2015__SID10_12M__bin.4	PSF	PSF_03
GCA_015831765.1	PSF	PSF_03
GCA_020297585.1	PSF	PSF_03
HeQ_2017__RSZAXPI003098-132__bin.	PSF	PSF_03
MCT420_8_S16_L002	PSF	PSF_03

SRR15283720	PSF	PSF_03
SRR15283753	PSF	PSF_03
SRR9206104	PSF	PSF_03
VatanenT_2016__G78592__bin.3	PSF	PSF_03
VatanenT_2016__G80297__bin.3	PSF	PSF_03
10317.X00179043_48	PSF	PSF_04
10317.X00179467_5	PSF	PSF_04
10317.X00180334_10317.X00180334_b	PSF	PSF_04
10317.X00185597_21_sub	PSF	PSF_04
363192575_S66_L001.1	PSF	PSF_04
363192583_S74_L001	PSF	PSF_04
363193035_S145_L001	PSF	PSF_04
363193043_S153_L001	PSF	PSF_04
363193047_S157_L001	PSF	PSF_04
363193063_S173_L001.2	PSF	PSF_04
GCA_000218345.1	PSF	PSF_04
GCA_000297735.1	PSF	PSF_04
GCA_000598585.1	PSF	PSF_04
GCA_000598845.1	PSF	PSF_04
GCA_000599325.1	PSF	PSF_04
GCA_002811085.1	PSF	PSF_04
GCA_003458215.1	PSF	PSF_04
GCA_008369705.1	PSF	PSF_04
GCA_014042455.1	PSF	PSF_04
GCA_014042505.1	PSF	PSF_04
GCA_015551365.1	PSF	PSF_04
GCA_015553755.1	PSF	PSF_04
GCA_015669295.1	PSF	PSF_04
GCA_015669435.1	PSF	PSF_04
GCA_022733395.1	PSF	PSF_04
GCA_022746405.1	PSF	PSF_04
LeChatelierE_2013__MH0068__bin.12	PSF	PSF_04
LiJ_2017__H3M515950__bin.24	PSF	PSF_04
MCT420_18_S26_L002	PSF	PSF_04
MCT420_50_S58_L002	PSF	PSF_04
MCT420_59_S67_L002	PSF	PSF_04
MCT420_75_S83_L002	PSF	PSF_04
SRR12280417	PSF	PSF_04

SRR12280493	PSF	PSF_04
SRR12280646	PSF	PSF_04
SRR12280649	PSF	PSF_04
SRR13269150	PSF	PSF_04
SRR13269157	PSF	PSF_04
SRR13269159	PSF	PSF_04
SRR15283691	PSF	PSF_04
SRR15283758	PSF	PSF_04
SRR15283766	PSF	PSF_04
VatanenT_2016__G78526__bin.12	PSF	PSF_04
VincentC_2016__MM032.3__bin.19	PSF	PSF_04
VogtmannE_2016__MMRS62192467ST-2	PSF	PSF_04
WenC_2017__N281__bin.22	PSF	PSF_04
10317.X00179047_10317.X00179047_b	PSF	PSF_05
363192563_S54_L001	PSF	PSF_05
BackhedF_2015__SID137_4M__bin.2	PSF	PSF_05
BackhedF_2015__SID137_M__bin.72	PSF	PSF_05
GCA_001816225.2	PSF	PSF_05
GCA_003458955.1	PSF	PSF_05
GCA_003464385.1	PSF	PSF_05
GCA_003468145.1	PSF	PSF_05
GCA_020558865.1	PSF	PSF_05
QinN_2014__LD-26__bin.50	PSF	PSF_05
SRR13269220	PSF	PSF_05
SRR15283767	PSF	PSF_05
VatanenT_2016__G80601__bin.2	PSF	PSF_05
10317.X00179298_6	PSF	PSF_06
10317.X00179673_25	PSF	PSF_06
10317.X00180216_72	PSF	PSF_06
363192603_S94_L001	PSF	PSF_06
363192797_S102_L001	PSF	PSF_06
363192805_S110_L001	PSF	PSF_06
363193029_S139_L001	PSF	PSF_06
363193048_S163_L001	PSF	PSF_06
363193069_S179_L001	PSF	PSF_06
363193756_S6_L001	PSF	PSF_06
363193763_S14_L001	PSF	PSF_06
GCA_008829725.1	PSF	PSF_06

GCA_018292185.1	PSF	PSF_06
MCT420_28_S36_L002	PSF	PSF_06
SRR12280650	PSF	PSF_06
SRR15283834	PSF	PSF_06
SRR9206393	PSF	PSF_06
VatanenT_2016__G80458__bin.3	PSF	PSF_06
VatanenT_2016__G80611__bin.2	PSF	PSF_06
10317.X00179327_33	PSF	PSF_07
10317.X00179407_24	PSF	PSF_07
363192577_S68_L001	PSF	PSF_07
363192585_S76_L001	PSF	PSF_07
GCA_003436935.1	PSF	PSF_07
GCA_003439675.1	PSF	PSF_07
GCA_020297425.1	PSF	PSF_07
SRR13269154	PSF	PSF_07
SRR15283749	PSF	PSF_07
WenC_2017__N016__bin.21	PSF	PSF_07
10317.X00179564_10317.X00179564_b	PSF	PSF_08
10317.X00180160_15	PSF	PSF_08
10317.X00185745_38	PSF	PSF_08
10317.X00185925_50	PSF	PSF_08
363192524_S22_L001.1	PSF	PSF_08
363193059_S169_L001	PSF	PSF_08
363193061_S171_L001	PSF	PSF_08
363193072_S181_L001	PSF	PSF_08
363193078_S188_L001	PSF	PSF_08
363197087_S326_L001.5	PSF	PSF_08
BackhedF_2015__SID180_4M__bin.6	PSF	PSF_08
GCA_000598225.1	PSF	PSF_08
GCA_000598705.1	PSF	PSF_08
GCA_001056335.1	PSF	PSF_08
GCA_001699875.1	PSF	PSF_08
GCA_003437815.1	PSF	PSF_08
GCA_003472025.1	PSF	PSF_08
GCA_007896595.1	PSF	PSF_08
GCA_009024655.1	PSF	PSF_08
GCA_009025705.1	PSF	PSF_08
GCA_012844105.1	PSF	PSF_08

GCA_014042415.1	PSF	PSF_08
GCA_014042445.1	PSF	PSF_08
GCA_015548595.1	PSF	PSF_08
GCA_015556305.1	PSF	PSF_08
GCA_016889925.1	PSF	PSF_08
GCA_020539125.1	PSF	PSF_08
KarlssonFH_2013__S248__bin.36	PSF	PSF_08
MCT420_35_S43_L002	PSF	PSF_08
MCT420_63_S71_L002	PSF	PSF_08
SRR13269149	PSF	PSF_08
SRR13269155	PSF	PSF_08
SRR13269156	PSF	PSF_08
SRR13269163	PSF	PSF_08
SRR13269194	PSF	PSF_08
SRR13269204	PSF	PSF_08
SRR15283707	PSF	PSF_08
SRR15283752	PSF	PSF_08
SRR15283828	PSF	PSF_08
SRR15283850	PSF	PSF_08
SRR15283854	PSF	PSF_08
SRR15283868	PSF	PSF_08
SRR15283869	PSF	PSF_08
SRR15283892	PSF	PSF_08
VatanenT_2016__G78910__bin.4	PSF	PSF_08
VatanenT_2016__G80507__bin.5	PSF	PSF_08
VogtmannE_2016__MMRS68910755ST-2	PSF	PSF_08
ZellerG_2014__CCIS58234805ST-4-0__b	PSF	PSF_08
10317.X00179651_55	PSF	PSF_09
363193049_S158_L001	PSF	PSF_09
363193060_S170_L001	PSF	PSF_09
363193064_S174_L001	PSF	PSF_09
363193076_S185_L001	PSF	PSF_09
GCA_000598565.1	PSF	PSF_09
GCA_000599165.2	PSF	PSF_09
GCA_020257125.1	PSF	PSF_09
MCT420_7_S15_L002	PSF	PSF_09
QinJ_2012__NLM028__bin.25	PSF	PSF_09
SRR13269209	PSF	PSF_09

SRR15283688	PSF	PSF_09
SRR15283701	PSF	PSF_09
SRR15283873	PSF	PSF_09
SRR15283891	PSF	PSF_09
10317.X00179673_25	PSF	PSF_10
10317.X00214299_94	PSF	PSF_10
363192552_S43_L001	PSF	PSF_10
363192805_S110_L001	PSF	PSF_10
363193029_S139_L001	PSF	PSF_10
363193048_S163_L001	PSF	PSF_10
363193069_S179_L001	PSF	PSF_10
GCA_003469305.1	PSF	PSF_10
GCA_018292185.1	PSF	PSF_10
MCT420_28_S36_L002	PSF	PSF_10
MCT420_49_S57_L002	PSF	PSF_10
QinN_2014__LD-50__bin.33	PSF	PSF_10
SRR15283724	PSF	PSF_10
SRR15283834	PSF	PSF_10
SRR9206393	PSF	PSF_10
10317.X00179736_10317.X00179736_b	PSF	PSF_11
363192564_S55_L001	PSF	PSF_11
DavidLA_2015__LD-Run1-08__bin.7	PSF	PSF_11
GCA_001682215.1	PSF	PSF_11
GCA_013867505.1	PSF	PSF_11
GCA_014042405.1	PSF	PSF_11
GCA_020859785.1	PSF	PSF_11
MCT420_76_S84_L002	PSF	PSF_11
QinJ_2012__DLF009__bin.9	PSF	PSF_11
QinN_2014__LD-34__bin.5	PSF	PSF_11
QinN_2014__LD-35__bin.21	PSF	PSF_11
SRR12280583	PSF	PSF_11
SRR12280608	PSF	PSF_11
SRR13269167	PSF	PSF_11
10317.X00179805_8	PSF	PSF_12
363192539_S19_L001	PSF	PSF_12
363192601_S92_L001	PSF	PSF_12
363192795_S100_L001	PSF	PSF_12
363193002_S105_L001	PSF	PSF_12

363193054_S164_L001	PSF	PSF_12
363193068_S178_L001	PSF	PSF_12
BackhedF_2015__SID582_12M__bin.6	PSF	PSF_12
BackhedF_2015__SID591_4M__bin.17	PSF	PSF_12
GCA_000599065.2	PSF	PSF_12
GCA_000601115.1	PSF	PSF_12
GCA_014893755.1	PSF	PSF_12
GCA_020297545.1	PSF	PSF_12
GCA_902374115.1	PSF	PSF_12
HeQ_2017__RSZAXPI003062-102__bin.	PSF	PSF_12
MCT420_54_S62_L002	PSF	PSF_12
NielsenHB_2014__V1_CD21_4__bin.5	PSF	PSF_12
Obregon-TitoAJ_2015__NO05__bin.48	PSF	PSF_12
SRR12280500	PSF	PSF_12
SRR12280591	PSF	PSF_12
SRR13269158	PSF	PSF_12
SRR13269165	PSF	PSF_12
SRR13269219	PSF	PSF_12
SRR13269224	PSF	PSF_12
SRR15283708	PSF	PSF_12
SRR15283728	PSF	PSF_12
SRR15283738	PSF	PSF_12
SRR15283837	PSF	PSF_12
SRR15283842	PSF	PSF_12
SRR15283864	PSF	PSF_12
VatanenT_2016__G78716__bin.3	PSF	PSF_12
VatanenT_2016__G80469__bin.5	PSF	PSF_12
VatanenT_2016__G80584__bin.1	PSF	PSF_12
VogtmannE_2016__MMRS77321055ST-2	PSF	PSF_12
XieH_2016__YSZC12003_37269__bin.2	PSF	PSF_12
XieH_2016__YSZC12003_37879__bin.3	PSF	PSF_12
YuJ_2015__SZAXPI015250-39__bin.22	PSF	PSF_12
10317.X00179954_75	PSF	PSF_13
363192566_S57_L001	PSF	PSF_13
363193027_S137_L001	PSF	PSF_13
363193754_S16_L001.2	PSF	PSF_13
GCA_000273765.1	PSF	PSF_13
GCA_002811035.1	PSF	PSF_13

SRR12280496	PSF	PSF_13
SRR12280498	PSF	PSF_13
SRR13269166	PSF	PSF_13
SRR15283750	PSF	PSF_13
SRR9206239	PSF	PSF_13
10317.X00185882_3	PSF	PSF_14
10317.X00214130_23	PSF	PSF_14
BackhedF_2015__SID130_12M__bin.13	PSF	PSF_14
MCT420_52_S60_L002	PSF	PSF_14
SRR12280315	PSF	PSF_14
SRR12280501	PSF	PSF_14
SRR15283714	PSF	PSF_14
SRR15283757	PSF	PSF_14
10317.X00214026_19	PSF	PSF_15
10317.X00214096_10317.X00214096_b	PSF	PSF_15
363192541_S20_L001	PSF	PSF_15
363192542_S33_L001.3	PSF	PSF_15
363192554_S45_L001.2	PSF	PSF_15
363192555_S46_L001	PSF	PSF_15
363192560_S51_L001	PSF	PSF_15
363192579_S70_L001	PSF	PSF_15
363192998_S106_L001	PSF	PSF_15
363193028_S138_L001	PSF	PSF_15
363193037_S147_L001	PSF	PSF_15
363193046_S156_L001	PSF	PSF_15
363193079_S189_L001	PSF	PSF_15
BackhedF_2015__SID1_4M__bin.8	PSF	PSF_15
BackhedF_2015__SID274_4M__bin.2	PSF	PSF_15
BackhedF_2015__SID521_4M__bin.12	PSF	PSF_15
BackhedF_2015__SID53_4M__bin.14	PSF	PSF_15
BackhedF_2015__SID626_4M__bin.17	PSF	PSF_15
GCA_000210835.1	PSF	PSF_15
GCA_000273155.1	PSF	PSF_15
GCA_000598205.1	PSF	PSF_15
GCA_000598525.1	PSF	PSF_15
GCA_000598905.1	PSF	PSF_15
GCA_001699855.1	PSF	PSF_15
GCA_003439505.1	PSF	PSF_15

GCA_003463555.1	PSF	PSF_15
GCA_003475745.1	PSF	PSF_15
GCA_004798445.1	PSF	PSF_15
GCA_004798525.1	PSF	PSF_15
GCA_015548495.1	PSF	PSF_15
GCA_015560515.1	PSF	PSF_15
GCA_018369215.1	PSF	PSF_15
GCA_019915805.1	PSF	PSF_15
GCA_021202455.1	PSF	PSF_15
GCA_022780605.1	PSF	PSF_15
GCA_905188165.1	PSF	PSF_15
LiJ_2014__MH0221__bin.8	PSF	PSF_15
MCT420_47_S55_L002	PSF	PSF_15
MCT420_77_S85_L002	PSF	PSF_15
NielsenHB_2014__V1_UC15_0__bin.1	PSF	PSF_15
SRR12280492	PSF	PSF_15
SRR12280549	PSF	PSF_15
SRR12280561	PSF	PSF_15
SRR12280645	PSF	PSF_15
SRR12280658	PSF	PSF_15
SRR13269207	PSF	PSF_15
SRR13269222	PSF	PSF_15
SRR15283695	PSF	PSF_15
SRR15283713	PSF	PSF_15
SRR15283820	PSF	PSF_15
SRR15283861	PSF	PSF_15
SRR15283894	PSF	PSF_15
VatanenT_2016__G78890__bin.1	PSF	PSF_15
VatanenT_2016__G80590__bin.4	PSF	PSF_15
VatanenT_2016__G80592__bin.1	PSF	PSF_15
XieH_2016__YSZC12003_37880__bin.1	PSF	PSF_15
10317.X00214097_10317.X00214097_b	PSF	PSF_16
GCA_022745625.1	PSF	PSF_16
363192525_S32_L001.2	PSF	PSF_17
363192595_S86_L001	PSF	PSF_17
BackhedF_2015__SID275_B__bin.6	PSF	PSF_17
BackhedF_2015__SID338_B__bin.1	PSF	PSF_17
BackhedF_2015__SID587_12M__bin.17	PSF	PSF_17

GCA_000598505.1	PSF	PSF_17
GCA_000598545.2	PSF	PSF_17
GCA_000601095.1	PSF	PSF_17
GCA_015558985.1	PSF	PSF_17
LiuW_2016__SRR3993044__bin.123	PSF	PSF_17
MCT420_2_S10_L002	PSF	PSF_17
SRR12280504	PSF	PSF_17
SRR13269223	PSF	PSF_17
SRR15283706	PSF	PSF_17
SRR15283731	PSF	PSF_17
SRR15283826	PSF	PSF_17
SRR15283838	PSF	PSF_17
SRR15283871	PSF	PSF_17
SRR15283876	PSF	PSF_17
363192548_S39_L001	PSF	PSF_18
363192557_S48_L001	PSF	PSF_18
BackhedF_2015__SID150_12M__bin.19	PSF	PSF_18
BackhedF_2015__SID150_M__bin.47	PSF	PSF_18
BackhedF_2015__SID238_12M__bin.7	PSF	PSF_18
BackhedF_2015__SID238_B__bin.1	PSF	PSF_18
BackhedF_2015__SID503_12M__bin.45	PSF	PSF_18
BackhedF_2015__SID503_4M__bin.20	PSF	PSF_18
BackhedF_2015__SID570_4M__bin.7	PSF	PSF_18
GCA_000598725.1	PSF	PSF_18
GCA_000598785.2	PSF	PSF_18
GCA_000965785.1	PSF	PSF_18
GCA_003469705.1	PSF	PSF_18
GCA_018786025.1	PSF	PSF_18
GCA_019130425.1	PSF	PSF_18
GeversD_2014__SKBSTL063__bin.2	PSF	PSF_18
LeChatelierE_2013__MH0410__bin.30	PSF	PSF_18
SRR12280270	PSF	PSF_18
SRR12280416	PSF	PSF_18
SRR12280499	PSF	PSF_18
SRR12280511	PSF	PSF_18
SRR12280632	PSF	PSF_18
SRR13269178	PSF	PSF_18
SRR13269221	PSF	PSF_18

SRR15283687	PSF	PSF_18
SRR15283839	PSF	PSF_18
SRR15283865	PSF	PSF_18
VatanenT_2016__G80290__bin.3	PSF	PSF_18
VatanenT_2016__G80313__bin.13	PSF	PSF_18
XieH_2016__YSZC12003_35524__bin.2	PSF	PSF_18
YuJ_2015__SZAXPI015216-169__bin.42	PSF	PSF_18
363192568_S59_L001	PSF	PSF_19
GCA_000598145.1	PSF	PSF_19
GCA_000598825.1	PSF	PSF_19
GCA_000724795.2	PSF	PSF_19
GCA_022832325.1	PSF	PSF_19
GCA_022832355.1	PSF	PSF_19
MCT420_10_S18_L002	PSF	PSF_19
SRR15283700	PSF	PSF_19
SRR15283722	PSF	PSF_19
SRR15283762	PSF	PSF_19
SRR15283771	PSF	PSF_19
363193001_S108_L001	PSF	PSF_20
BackhedF_2015__SID566_12M__bin.34	PSF	PSF_20
GCA_000599365.1	PSF	PSF_20
GCA_001699865.1	PSF	PSF_20
GCA_023702735.1	PSF	PSF_20
SchirmerM_2016__G88708__bin.4	PSF	PSF_20
BackhedF_2015__SID137_M__bin.72	PSF	PSF_21
BackhedF_2015__SID526_4M__bin.6	PSF	PSF_21
GCA_001816225.2	PSF	PSF_21
GCA_020558865.1	PSF	PSF_21
QinN_2014__LD-26__bin.50	PSF	PSF_21
VatanenT_2016__G80601__bin.2	PSF	PSF_21
GCA_003436285.1	PSF	PSF_22
GCA_003475385.1	PSF	PSF_22
MCT420_60_S68_L002	PSF	PSF_22
SRR13269164	PSF	PSF_22
SRR15283844	PSF	PSF_23
VatanenT_2016__G80518__bin.7	PSF	PSF_24
WenC_2017__RA132__bin.29	PSF	PSF_24
10317.X00179017_10317.X00179017_b	PSG	PSG_01

363192044_S216_L001.3	PSC	PSG_01
363192544_S35_L001	PSC	PSG_01
363192573_S64_L001	PSC	PSG_01
363192581_S72_L001	PSC	PSG_01
363193001_S108_L001	PSC	PSG_01
363193022_S130_L001.1	PSC	PSG_01
363193036_S146_L001	PSC	PSG_01
363193757_S8_L001	PSC	PSG_01
363197054_S337_L001.2	PSC	PSG_01
BackhedF_2015__SID10_12M__bin.4	PSC	PSG_01
BackhedF_2015__SID203_12M__bin.9	PSC	PSG_01
BackhedF_2015__SID566_12M__bin.34	PSC	PSG_01
BackhedF_2015__SID567_12M__bin.4	PSC	PSG_01
CM_madagascar__A39_02_1FE__bin.19	PSC	PSG_01
GCA_003472195.1	PSC	PSG_01
GCA_020297585.1	PSC	PSG_01
GCA_022722555.1	PSC	PSG_01
GCA_024007805.1	PSC	PSG_01
HeQ_2017__RSZAXPI003078-111__bin.	PSC	PSG_01
HeQ_2017__RSZAXPI003098-132__bin.	PSC	PSG_01
KosticAD_2015__PRJNA231909.311383	PSC	PSG_01
KosticAD_2015__PRJNA231909.311383	PSC	PSG_01
MCT420_35_S43_L002	PSC	PSG_01
MCT420_41_S49_L002	PSC	PSG_01
MCT420_6_S14_L002	PSC	PSG_01
MCT420_81_S89_L002	PSC	PSG_01
MCT420_86_S94_L002	PSC	PSG_01
MCT420_8_S16_L002	PSC	PSG_01
SRR12280522	PSC	PSG_01
SRR13269245	PSC	PSG_01
SRR15283720	PSC	PSG_01
SRR15283747	PSC	PSG_01
SRR15283820	PSC	PSG_01
VatanenT_2016__G80297__bin.3	PSC	PSG_01
XieH_2016__YSZC12003_37207R1__bir	PSC	PSG_01
10317.X00179025_65	PSC	PSG_02
10317.X00179298_6	PSC	PSG_02
10317.X00179673_25	PSC	PSG_02

10317.X00179954_75	PSG	PSG_02
10317.X00214694_67	PSG	PSG_02
363192527_S10_L001	PSG	PSG_02
363192603_S94_L001	PSG	PSG_02
363192797_S102_L001	PSG	PSG_02
363192805_S110_L001	PSG	PSG_02
363193029_S139_L001	PSG	PSG_02
363193046_S156_L001	PSG	PSG_02
363193048_S163_L001	PSG	PSG_02
363193069_S179_L001	PSG	PSG_02
363193754_S16_L001.2	PSG	PSG_02
363193756_S6_L001	PSG	PSG_02
363193763_S14_L001	PSG	PSG_02
BackhedF_2015__SID1_4M__bin.8	PSG	PSG_02
BackhedF_2015__SID229_B__bin.3	PSG	PSG_02
BackhedF_2015__SID274_4M__bin.2	PSG	PSG_02
GCA_000210835.1	PSG	PSG_02
GCA_000598245.1	PSG	PSG_02
GCA_000598525.1	PSG	PSG_02
GCA_000598905.1	PSG	PSG_02
GCA_000599125.1	PSG	PSG_02
GCA_000601075.2	PSG	PSG_02
GCA_001699855.1	PSG	PSG_02
GCA_002811035.1	PSG	PSG_02
GCA_003436285.1	PSG	PSG_02
GCA_003436935.1	PSG	PSG_02
GCA_003438895.1	PSG	PSG_02
GCA_003475385.1	PSG	PSG_02
GCA_004798445.1	PSG	PSG_02
GCA_004798525.1	PSG	PSG_02
GCA_018292185.1	PSG	PSG_02
GCA_018369215.1	PSG	PSG_02
GCA_019733435.1	PSG	PSG_02
GCA_019915805.1	PSG	PSG_02
GCA_022733395.1	PSG	PSG_02
GCA_022744835.1	PSG	PSG_02
GCA_022746405.1	PSG	PSG_02
GCA_022832315.1	PSG	PSG_02

GCA_900624785.1	PS(	PSG_02
HMP_2012__SRS018817__bin.4	PS(	PSG_02
HeQ_2017__SZAXPI029574-89__bin.37	PS(	PSG_02
LiJ_2014__MH0221__bin.8	PS(	PSG_02
MCT420_28_S36_L002	PS(	PSG_02
MCT420_29_S37_L002	PS(	PSG_02
MCT420_60_S68_L002	PS(	PSG_02
SRR12280496	PS(	PSG_02
SRR13269164	PS(	PSG_02
SRR13269166	PS(	PSG_02
SRR13269207	PS(	PSG_02
SRR13269222	PS(	PSG_02
SRR15283697	PS(	PSG_02
SRR15283750	PS(	PSG_02
SRR15283834	PS(	PSG_02
VatanenT_2016__G78583__bin.3	PS(	PSG_02
VatanenT_2016__G78605__bin.3	PS(	PSG_02
ZellerG_2014__CCIS76845094ST-20-0__	PS(	PSG_02
10317.X00179093_41	PS(	PSG_03
10317.X00179805_8	PS(	PSG_03
363192539_S19_L001	PS(	PSG_03
363192601_S92_L001	PS(	PSG_03
363192795_S100_L001	PS(	PSG_03
363193002_S105_L001	PS(	PSG_03
363193003_S113_L001.4	PS(	PSG_03
363193054_S164_L001	PS(	PSG_03
363193068_S178_L001	PS(	PSG_03
BackhedF_2015__SID582_12M__bin.6	PS(	PSG_03
BackhedF_2015__SID591_4M__bin.17	PS(	PSG_03
GCA_000599065.2	PS(	PSG_03
GCA_000601115.1	PS(	PSG_03
GCA_014893755.1	PS(	PSG_03
GCA_020297545.1	PS(	PSG_03
GCA_022716295.1	PS(	PSG_03
GCA_022734965.1	PS(	PSG_03
GCA_902374115.1	PS(	PSG_03
HeQ_2017__RSZAXPI003062-102__bin.	PS(	PSG_03
KosticAD_2015__PRJNA231909.311234	PS(	PSG_03

MCT420_54_S62_L002	PS(	PSG_03
NielsenHB_2014__V1_CD21_4__bin.5	PS(	PSG_03
Obregon-TitoAJ_2015__NO05__bin.48	PS(	PSG_03
SRR12280500	PS(	PSG_03
SRR12280591	PS(	PSG_03
SRR13269158	PS(	PSG_03
SRR13269165	PS(	PSG_03
SRR13269219	PS(	PSG_03
SRR13269224	PS(	PSG_03
SRR15283708	PS(	PSG_03
SRR15283728	PS(	PSG_03
SRR15283738	PS(	PSG_03
SRR15283837	PS(	PSG_03
SRR15283842	PS(	PSG_03
SRR15283864	PS(	PSG_03
VatanenT_2016__G78716__bin.3	PS(	PSG_03
VatanenT_2016__G80469__bin.5	PS(	PSG_03
VatanenT_2016__G80584__bin.1	PS(	PSG_03
VincentC_2016__MM022.2__bin.34	PS(	PSG_03
VogtmannE_2016__MMRS77321055ST-2	PS(	PSG_03
XieH_2016__YSZC12003_37269__bin.2	PS(	PSG_03
XieH_2016__YSZC12003_37879__bin.3	PS(	PSG_03
YuJ_2015__SZAXPI003435-11__bin.64	PS(	PSG_03
YuJ_2015__SZAXPI015250-39__bin.22	PS(	PSG_03
ZeeviD_2015__PNP_Main_174__bin.22	PS(	PSG_03
ZeeviD_2015__PNP_Validation_92__bin.	PS(	PSG_03
10317.X00179300_11	PS(	PSG_04
10317.X00180243_84	PS(	PSG_04
10317.X00180463_38	PS(	PSG_04
10317.X00214812_39	PS(	PSG_04
GCA_000273765.1	PS(	PSG_04
GCA_000724795.2	PS(	PSG_04
GCA_015553495.1	PS(	PSG_04
GCA_015831765.1	PS(	PSG_04
GCA_022832325.1	PS(	PSG_04
GCA_022832355.1	PS(	PSG_04
LiJ_2014__V1.FI21__bin.42	PS(	PSG_04
MCT420_58_S66_L002	PS(	PSG_04

SRR12280314	PSC	PSG_04
SRR13269155	PSC	PSG_04
SRR15283700	PSC	PSG_04
SRR15283722	PSC	PSG_04
SRR15283752	PSC	PSG_04
SRR15283762	PSC	PSG_04
SRR15283771	PSC	PSG_04
10317.X00179306_29	PSC	PSG_05
10317.X00180464_10317.X00180464_b	PSC	PSG_05
10317.X00215482_24	PSC	PSG_05
363192528_S11_L001	PSC	PSG_05
363192589_S80_L001	PSC	PSG_05
BackhedF_2015__SID275_B__bin.6	PSC	PSG_05
BackhedF_2015__SID78_4M__bin.8	PSC	PSG_05
GCA_001286525.1	PSC	PSG_05
GCA_008569345.1	PSC	PSG_05
GCA_014042465.1	PSC	PSG_05
GCA_020297435.1	PSC	PSG_05
MCT420_36_S44_L002	PSC	PSG_05
SRR12280589	PSC	PSG_05
SRR15283742	PSC	PSG_05
VatanenT_2016__G80299__bin.1	PSC	PSG_05
10317.X00179327_33	PSC	PSG_06
363192577_S68_L001	PSC	PSG_06
363192585_S76_L001	PSC	PSG_06
VatanenT_2016__G80532__bin.12	PSC	PSG_06
VatanenT_2016__G80556__bin.10	PSC	PSG_06
10317.X00179564_10317.X00179564_b	PSC	PSG_07
10317.X00180095_68	PSC	PSG_07
10317.X00180160_15	PSC	PSG_07
10317.X00185745_38	PSC	PSG_07
10317.X00185925_50	PSC	PSG_07
363192524_S22_L001.1	PSC	PSG_07
363193016_S126_L001.1	PSC	PSG_07
363193039_S149_L001	PSC	PSG_07
363193059_S169_L001	PSC	PSG_07
363193061_S171_L001	PSC	PSG_07
363193072_S181_L001	PSC	PSG_07

363193078_S188_L001	PSC	PSG_07
BackhedF_2015__SID587_12M__bin.17	PSC	PSG_07
BackhedF_2015__SID615_12M__bin.2	PSC	PSG_07
BackhedF_2015__SID615_4M__bin.5	PSC	PSG_07
GCA_000598545.2	PSC	PSG_07
GCA_000601095.1	PSC	PSG_07
GCA_001056335.1	PSC	PSG_07
GCA_001699865.1	PSC	PSG_07
GCA_003437815.1	PSC	PSG_07
GCA_003472025.1	PSC	PSG_07
GCA_007896595.1	PSC	PSG_07
GCA_009025705.1	PSC	PSG_07
GCA_012844105.1	PSC	PSG_07
GCA_014042415.1	PSC	PSG_07
GCA_014042445.1	PSC	PSG_07
GCA_015548595.1	PSC	PSG_07
GCA_016889925.1	PSC	PSG_07
GCA_020539125.1	PSC	PSG_07
GCA_023702735.1	PSC	PSG_07
MCT420_55_S63_L002	PSC	PSG_07
MCT420_63_S71_L002	PSC	PSG_07
SRR12280503	PSC	PSG_07
SRR13269149	PSC	PSG_07
SRR13269156	PSC	PSG_07
SRR13269163	PSC	PSG_07
SRR15283685	PSC	PSG_07
SRR15283707	PSC	PSG_07
SRR15283731	PSC	PSG_07
SRR15283822	PSC	PSG_07
SRR15283826	PSC	PSG_07
SRR15283828	PSC	PSG_07
SRR15283850	PSC	PSG_07
SRR15283854	PSC	PSG_07
SRR15283869	PSC	PSG_07
SRR15283892	PSC	PSG_07
VogtmannE_2016__MMRS68910755ST-2	PSC	PSG_07
ZeeviD_2015__PNP_Main_319__bin.2	PSC	PSG_07
ZellerG_2014__CCIS58234805ST-4-0__b	PSC	PSG_07

10317.X00179729_16	PSC	PSG_08
363192999_S107_L001	PSC	PSG_08
363193004_S114_L001	PSC	PSG_08
363197065_S315_L001	PSC	PSG_08
GCA_000598705.1	PSC	PSG_08
GCA_000598865.1	PSC	PSG_08
GCA_003439675.1	PSC	PSG_08
GCA_008086695.1	PSC	PSG_08
GCA_901212445.1	PSC	PSG_08
LiJ_2017__H1M213648__bin.18	PSC	PSG_08
MCT420_32_S40_L002	PSC	PSG_08
VatanenT_2016__G78739__bin.3	PSC	PSG_08
VatanenT_2016__G80499__bin.3	PSC	PSG_08
10317.X00180154_10317.X00180154_b	PSC	PSG_09
10317.X00182267_21	PSC	PSG_09
BackhedF_2015__SID180_4M__bin.6	PSC	PSG_09
GCA_009024655.1	PSC	PSG_09
KarlssonFH_2013__S248__bin.36	PSC	PSG_09
VatanenT_2016__G80507__bin.5	PSC	PSG_09
10317.X00180334_10317.X00180334_b	PSC	PSG_10
10317.X00180334_10317.X00180334_b	PSC	PSG_10
363193043_S153_L001	PSC	PSG_10
GCA_000297735.1	PSC	PSG_10
GCA_000598845.1	PSC	PSG_10
GCA_014042455.1	PSC	PSG_10
GCA_015669295.1	PSC	PSG_10
GCA_022782465.1	PSC	PSG_10
LeChatelierE_2013__MH0068__bin.12	PSC	PSG_10
MCT420_49_S57_L002	PSC	PSG_10
MCT420_50_S58_L002	PSC	PSG_10
MCT420_75_S83_L002	PSC	PSG_10
QinN_2014__LD-50__bin.33	PSC	PSG_10
SRR12280649	PSC	PSG_10
SRR13269150	PSC	PSG_10
SRR13269159	PSC	PSG_10
SRR15283724	PSC	PSG_10
SRR9206393	PSC	PSG_10
VincentC_2016__MM032.3__bin.19	PSC	PSG_10

10317.X00185752_7	PS(	PSG_11
363192564_S55_L001	PS(	PSG_11
DavidLA_2015__LD-Run1-08__bin.7	PS(	PSG_11
GCA_001682215.1	PS(	PSG_11
GCA_013867505.1	PS(	PSG_11
GCA_014042405.1	PS(	PSG_11
GCA_020859785.1	PS(	PSG_11
GCA_022733815.1	PS(	PSG_11
MCT420_76_S84_L002	PS(	PSG_11
QinN_2014__LD-34__bin.5	PS(	PSG_11
QinN_2014__LD-35__bin.21	PS(	PSG_11
SRR12280583	PS(	PSG_11
SRR12280608	PS(	PSG_11
SRR13269167	PS(	PSG_11
10317.X00214130_23	PS(	PSG_12
BackhedF_2015__SID130_12M__bin.13	PS(	PSG_12
BackhedF_2015__SID130_4M__bin.3	PS(	PSG_12
MCT420_52_S60_L002	PS(	PSG_12
SRR12280315	PS(	PSG_12
SRR12280501	PS(	PSG_12
SRR15283714	PS(	PSG_12
SRR15283757	PS(	PSG_12
363192525_S32_L001.2	PS(	PSG_13
363192595_S86_L001	PS(	PSG_13
BackhedF_2015__SID133_4M__bin.23	PS(	PSG_13
GCA_000598985.1	PS(	PSG_13
GCA_015558985.1	PS(	PSG_13
MCT420_10_S18_L002	PS(	PSG_13
MCT420_2_S10_L002	PS(	PSG_13
SRR12280504	PS(	PSG_13
SRR12280651	PS(	PSG_13
SRR13269122	PS(	PSG_13
SRR13269162	PS(	PSG_13
SRR15283749	PS(	PSG_13
SRR15283763	PS(	PSG_13
SRR15283838	PS(	PSG_13
363192548_S39_L001	PS(	PSG_14
363192557_S48_L001	PS(	PSG_14

363192579_S70_L001	PSC	PSG_14
BackhedF_2015__SID150_12M__bin.19	PSC	PSG_14
BackhedF_2015__SID150_M__bin.47	PSC	PSG_14
BackhedF_2015__SID238_12M__bin.7	PSC	PSG_14
BackhedF_2015__SID238_B__bin.1	PSC	PSG_14
BackhedF_2015__SID503_12M__bin.45	PSC	PSG_14
BackhedF_2015__SID503_4M__bin.20	PSC	PSG_14
BackhedF_2015__SID570_4M__bin.7	PSC	PSG_14
GCA_000598725.1	PSC	PSG_14
GCA_000598785.2	PSC	PSG_14
GCA_000965785.1	PSC	PSG_14
GCA_003469705.1	PSC	PSG_14
GCA_018786025.1	PSC	PSG_14
GCA_019130425.1	PSC	PSG_14
GCA_022725025.1	PSC	PSG_14
GCA_022734285.1	PSC	PSG_14
GCA_022741265.1	PSC	PSG_14
GeversD_2014__SKBSTL063__bin.2	PSC	PSG_14
LeChatelierE_2013__MH0410__bin.30	PSC	PSG_14
SRR12280270	PSC	PSG_14
SRR12280416	PSC	PSG_14
SRR12280499	PSC	PSG_14
SRR12280511	PSC	PSG_14
SRR12280632	PSC	PSG_14
SRR13269178	PSC	PSG_14
SRR13269221	PSC	PSG_14
SRR15283687	PSC	PSG_14
SRR15283839	PSC	PSG_14
SRR15283861	PSC	PSG_14
SRR15283865	PSC	PSG_14
SRR15283894	PSC	PSG_14
VatanenT_2016__G80290__bin.3	PSC	PSG_14
VatanenT_2016__G80313__bin.13	PSC	PSG_14
XieH_2016__YSZC12003_35524__bin.2	PSC	PSG_14
XieH_2016__YSZC12003_37205__bin.2	PSC	PSG_14
YuJ_2015__SZAXPI015216-169__bin.42	PSC	PSG_14
363192552_S43_L001	PSC	PSG_15
GCA_003469305.1	PSC	PSG_15

363193035_S145_L001	PSG	PSG_16
GCA_015553755.1	PSG	PSG_16
MCT420_18_S26_L002	PSG	PSG_16
MCT420_59_S67_L002	PSG	PSG_16
363193047_S157_L001	PSG	PSG_17
GCA_000218345.1	PSG	PSG_17
GCA_008369705.1	PSG	PSG_17
SRR12280646	PSG	PSG_17
VatanenT_2016__G80602__bin.2	PSG	PSG_17
363193049_S158_L001	PSG	PSG_18
363193060_S170_L001	PSG	PSG_18
363193064_S174_L001	PSG	PSG_18
363193076_S185_L001	PSG	PSG_18
BackhedF_2015__SID137_12M__bin.25	PSG	PSG_18
GCA_000598565.1	PSG	PSG_18
GCA_000599165.2	PSG	PSG_18
GCA_020257125.1	PSG	PSG_18
HMP_2012__SRS075398__bin.15	PSG	PSG_18
MCT420_7_S15_L002	PSG	PSG_18
QinJ_2012__NLM028__bin.25	PSG	PSG_18
SRR13269209	PSG	PSG_18
SRR15283688	PSG	PSG_18
SRR15283701	PSG	PSG_18
SRR15283873	PSG	PSG_18
SRR15283891	PSG	PSG_18
GCA_000598145.1	PSG	PSG_19
GCA_000598285.1	PSG	PSG_19
GCA_000598825.1	PSG	PSG_19
GCA_000599365.1	PSG	PSG_19
GCA_022733095.1	PSG	PSG_19
GCA_022745295.1	PSG	PSG_19
SRR12280650	PSG	PSG_19
SRR15283753	PSG	PSG_19
GCA_014042525.1	PSG	PSG_20
SRR15283695	PSG	PSG_20
SRR15283896	PSG	PSG_20
SRR15283767	PSG	PSG_21
10317.X00178995_62	PSH	PSH_01

10317.X00179306_29	PSH	PSH_01
10317.X00179317_4	PSH	PSH_01
10317.X00180464_10317.X00180464_b	PSH	PSH_01
10317.X00214299_94	PSH	PSH_01
10317.X00215482_24	PSH	PSH_01
363192528_S11_L001	PSH	PSH_01
363192554_S45_L001.2	PSH	PSH_01
363192555_S46_L001	PSH	PSH_01
363192589_S80_L001	PSH	PSH_01
363193039_S149_L001	PSH	PSH_01
363193079_S189_L001	PSH	PSH_01
BackhedF_2015__SID78_4M__bin.8	PSH	PSH_01
GCA_000598725.1	PSH	PSH_01
GCA_001286525.1	PSH	PSH_01
GCA_003439675.1	PSH	PSH_01
GCA_003469305.1	PSH	PSH_01
GCA_008569345.1	PSH	PSH_01
GCA_014042465.1	PSH	PSH_01
GCA_015553295.1	PSH	PSH_01
GCA_019041875.1	PSH	PSH_01
GCA_020297435.1	PSH	PSH_01
MCT420_32_S40_L002	PSH	PSH_01
MCT420_36_S44_L002	PSH	PSH_01
SRR12280503	PSH	PSH_01
SRR12280589	PSH	PSH_01
SRR15283685	PSH	PSH_01
SRR15283742	PSH	PSH_01
SRR15283822	PSH	PSH_01
SchirmerM_2016__G88708__bin.4	PSH	PSH_01
VatanenT_2016__G78583__bin.3	PSH	PSH_01
VatanenT_2016__G80590__bin.4	PSH	PSH_01
VatanenT_2016__G80602__bin.2	PSH	PSH_01
10317.X00179025_65	PSH	PSH_02
10317.X00179300_11	PSH	PSH_02
10317.X00180243_84	PSH	PSH_02
10317.X00185696_11	PSH	PSH_02
GCA_000210835.1	PSH	PSH_02
GCA_000598665.1	PSH	PSH_02

GCA_000601055.1	PSH	PSH_02
GCA_004798445.1	PSH	PSH_02
GCA_004798525.1	PSH	PSH_02
GCA_015553495.1	PSH	PSH_02
GCA_019915805.1	PSH	PSH_02
GCA_020558655.1	PSH	PSH_02
GCA_022714305.1	PSH	PSH_02
GCA_022733395.1	PSH	PSH_02
GCA_022744835.1	PSH	PSH_02
GCA_022746405.1	PSH	PSH_02
GCA_022832325.1	PSH	PSH_02
GCA_022832355.1	PSH	PSH_02
GeversD_2014__SKSTL0001__bin.2	PSH	PSH_02
MCT420_58_S66_L002	PSH	PSH_02
NielsenHB_2014__O2_UC34_2__bin.41	PSH	PSH_02
SRR12280314	PSH	PSH_02
SRR12280572	PSH	PSH_02
SRR13269222	PSH	PSH_02
SRR15283700	PSH	PSH_02
SRR15283722	PSH	PSH_02
SRR15283762	PSH	PSH_02
SRR15283771	PSH	PSH_02
10317.X00179043_48	PSH	PSH_03
10317.X00179274_24	PSH	PSH_03
10317.X00179487_25	PSH	PSH_03
10317.X00180463_38	PSH	PSH_03
363193035_S145_L001	PSH	PSH_03
GCA_000598585.1	PSH	PSH_03
GCA_014042505.1	PSH	PSH_03
GCA_015553755.1	PSH	PSH_03
MCT420_18_S26_L002	PSH	PSH_03
SRR12280417	PSH	PSH_03
SRR13269155	PSH	PSH_03
SRR15283706	PSH	PSH_03
SRR15283752	PSH	PSH_03
SRR15283758	PSH	PSH_03
VatanenT_2016__G78526__bin.12	PSH	PSH_03
10317.X00179093_41	PSH	PSH_04

10317.X00179805_8	PSI PSH_04
363192539_S19_L001	PSI PSH_04
363192601_S92_L001	PSI PSH_04
363192795_S100_L001	PSI PSH_04
363193002_S105_L001	PSI PSH_04
363193003_S113_L001.4	PSI PSH_04
363193054_S164_L001	PSI PSH_04
363193068_S178_L001	PSI PSH_04
BackhedF_2015__SID130_12M__bin.13	PSI PSH_04
BackhedF_2015__SID130_4M__bin.3	PSI PSH_04
BackhedF_2015__SID582_12M__bin.6	PSI PSH_04
BackhedF_2015__SID591_4M__bin.17	PSI PSH_04
GCA_000599065.2	PSI PSH_04
GCA_000601115.1	PSI PSH_04
GCA_014893755.1	PSI PSH_04
GCA_020297545.1	PSI PSH_04
GCA_022716295.1	PSI PSH_04
GCA_022734965.1	PSI PSH_04
GCA_902374115.1	PSI PSH_04
HeQ_2017__RSZAXPI003062-102__bin.	PSI PSH_04
KosticAD_2015__PRJNA231909.311234	PSI PSH_04
MCT420_54_S62_L002	PSI PSH_04
NielsenHB_2014__V1_CD21_4__bin.5	PSI PSH_04
Obregon-TitoAJ_2015__NO05__bin.48	PSI PSH_04
SRR12280500	PSI PSH_04
SRR12280591	PSI PSH_04
SRR13269158	PSI PSH_04
SRR13269165	PSI PSH_04
SRR13269219	PSI PSH_04
SRR13269224	PSI PSH_04
SRR15283708	PSI PSH_04
SRR15283728	PSI PSH_04
SRR15283738	PSI PSH_04
SRR15283837	PSI PSH_04
SRR15283842	PSI PSH_04
SRR15283864	PSI PSH_04
VatanenT_2016__G78716__bin.3	PSI PSH_04
VatanenT_2016__G80469__bin.5	PSI PSH_04

VatanenT_2016__G80584__bin.1	PSH	PSH_04
VincentC_2016__MM022.2__bin.34	PSH	PSH_04
VogtmannE_2016__MMRS77321055ST-2	PSH	PSH_04
XieH_2016__YSZC12003_37269__bin.2	PSH	PSH_04
XieH_2016__YSZC12003_37879__bin.3	PSH	PSH_04
YuJ_2015__SZAXPI003435-11__bin.64	PSH	PSH_04
YuJ_2015__SZAXPI015250-39__bin.22	PSH	PSH_04
ZeeviD_2015__PNP_Main_174__bin.22	PSH	PSH_04
ZeeviD_2015__PNP_Validation_92__bin.	PSH	PSH_04
10317.X00179153_19	PSH	PSH_05
10317.X00179156_10	PSH	PSH_05
10317.X00179407_24	PSH	PSH_05
10317.X00180216_72	PSH	PSH_05
10317.X00214812_39	PSH	PSH_05
363192568_S59_L001	PSH	PSH_05
363193060_S170_L001	PSH	PSH_05
BackhedF_2015__SID275_B__bin.6	PSH	PSH_05
BackhedF_2015__SID275_M__bin.26	PSH	PSH_05
GCA_000598565.1	PSH	PSH_05
GCA_000599165.2	PSH	PSH_05
GCA_000599325.1	PSH	PSH_05
GCA_003436285.1	PSH	PSH_05
GCA_003475385.1	PSH	PSH_05
GCA_008369705.1	PSH	PSH_05
GCA_009025705.1	PSH	PSH_05
GCA_014042445.1	PSH	PSH_05
GCA_015558985.1	PSH	PSH_05
GCA_016889925.1	PSH	PSH_05
GCA_019013355.1	PSH	PSH_05
GCA_022720585.1	PSH	PSH_05
GCA_022721415.1	PSH	PSH_05
GCA_022737795.1	PSH	PSH_05
GCA_022747335.1	PSH	PSH_05
HMP_2012__SRS075398__bin.15	PSH	PSH_05
MCT420_60_S68_L002	PSH	PSH_05
MCT420_7_S15_L002	PSH	PSH_05
SRR12280315	PSH	PSH_05
SRR12280504	PSH	PSH_05

SRR12280646	PSI PSH_05
SRR13269154	PSI PSH_05
SRR15283701	PSI PSH_05
SRR15283720	PSI PSH_05
SRR15283757	PSI PSH_05
SRR15283826	PSI PSH_05
SRR15283854	PSI PSH_05
SRR15283892	PSI PSH_05
VogtmannE_2016__MMRS68910755ST-2	PSI PSH_05
ZeeviD_2015__PNP_Main_319__bin.2	PSI PSH_05
10317.X00179298_6	PSI PSH_06
10317.X00179673_25	PSI PSH_06
363192575_S66_L001.1	PSI PSH_06
363192583_S74_L001	PSI PSH_06
363192603_S94_L001	PSI PSH_06
363192805_S110_L001	PSI PSH_06
363193029_S139_L001	PSI PSH_06
363193048_S163_L001	PSI PSH_06
363193069_S179_L001	PSI PSH_06
BackhedF_2015__SID1_4M__bin.8	PSI PSH_06
GCA_004167855.1	PSI PSH_06
GCA_015551365.1	PSI PSH_06
GCA_018292185.1	PSI PSH_06
HeQ_2017__SZAXPI029574-89__bin.37	PSI PSH_06
MCT420_28_S36_L002	PSI PSH_06
SRR12280493	PSI PSH_06
SRR13269204	PSI PSH_06
SRR15283687	PSI PSH_06
SRR15283691	PSI PSH_06
SRR15283834	PSI PSH_06
XieH_2016__YSZC12003_36052__bin.34	PSI PSH_06
10317.X00179381_10317.X00179381_b	PSI PSH_07
10317.X00180334_10317.X00180334_b	PSI PSH_07
10317.X00180334_10317.X00180334_b	PSI PSH_07
363193043_S153_L001	PSI PSH_07
BackhedF_2015__SID238_12M__bin.7	PSI PSH_07
BackhedF_2015__SID238_B__bin.1	PSI PSH_07
GCA_000297735.1	PSI PSH_07

GCA_000598845.1	PSH	PSH_07
GCA_014042455.1	PSH	PSH_07
GCA_015669295.1	PSH	PSH_07
GCA_022782465.1	PSH	PSH_07
GCA_905188165.1	PSH	PSH_07
LeChatelierE_2013__MH0068__bin.12	PSH	PSH_07
MCT420_29_S37_L002	PSH	PSH_07
MCT420_50_S58_L002	PSH	PSH_07
MCT420_75_S83_L002	PSH	PSH_07
SRR12280649	PSH	PSH_07
SRR13269150	PSH	PSH_07
SRR13269159	PSH	PSH_07
SRR13269178	PSH	PSH_07
SRR15283844	PSH	PSH_07
SRR9206393	PSH	PSH_07
VincentC_2016__MM032.3__bin.19	PSH	PSH_07
WenC_2017__N016__bin.21	PSH	PSH_07
10317.X00179467_5	PSH	PSH_08
10317.X00179564_10317.X00179564_b	PSH	PSH_08
10317.X00180160_15	PSH	PSH_08
10317.X00185745_38	PSH	PSH_08
10317.X00185925_50	PSH	PSH_08
363192533_S31_L001	PSH	PSH_08
363192548_S39_L001	PSH	PSH_08
363192552_S43_L001	PSH	PSH_08
363192557_S48_L001	PSH	PSH_08
363192566_S57_L001	PSH	PSH_08
363192579_S70_L001	PSH	PSH_08
363192797_S102_L001	PSH	PSH_08
363193028_S138_L001	PSH	PSH_08
363193032_S142_L001	PSH	PSH_08
363193047_S157_L001	PSH	PSH_08
363193059_S169_L001	PSH	PSH_08
363193072_S181_L001	PSH	PSH_08
363193078_S188_L001	PSH	PSH_08
BackhedF_2015__SID150_12M__bin.19	PSH	PSH_08
BackhedF_2015__SID150_M__bin.47	PSH	PSH_08
BackhedF_2015__SID503_12M__bin.45	PSH	PSH_08

BackhedF_2015__SID503_4M__bin.20	PSH	PSH_08
BritoIL_2016__M1.10.ST__bin.49	PSH	PSH_08
ChengpingW_2017__AS123raw__bin.19	PSH	PSH_08
GCA_000218345.1	PSH	PSH_08
GCA_000598225.1	PSH	PSH_08
GCA_000598785.2	PSH	PSH_08
GCA_000598985.1	PSH	PSH_08
GCA_000965785.1	PSH	PSH_08
GCA_001056335.1	PSH	PSH_08
GCA_001699865.1	PSH	PSH_08
GCA_003437815.1	PSH	PSH_08
GCA_003469705.1	PSH	PSH_08
GCA_003472025.1	PSH	PSH_08
GCA_007896595.1	PSH	PSH_08
GCA_014042415.1	PSH	PSH_08
GCA_015548595.1	PSH	PSH_08
GCA_015669435.1	PSH	PSH_08
GCA_019130425.1	PSH	PSH_08
GCA_020539125.1	PSH	PSH_08
GCA_022725025.1	PSH	PSH_08
GCA_022725225.1	PSH	PSH_08
GCA_022727875.1	PSH	PSH_08
GCA_022731325.1	PSH	PSH_08
GCA_022734285.1	PSH	PSH_08
GCA_022741265.1	PSH	PSH_08
GCA_022745625.1	PSH	PSH_08
GCA_022746345.1	PSH	PSH_08
GCA_023702735.1	PSH	PSH_08
LeChatelierE_2013__MH0193__bin.44	PSH	PSH_08
LiJ_2014__V1.CD3-0-PN__bin.42	PSH	PSH_08
QinN_2014__LD-33__bin.18	PSH	PSH_08
QinN_2014__LD-93__bin.8	PSH	PSH_08
SRR12280416	PSH	PSH_08
SRR12280499	PSH	PSH_08
SRR12280511	PSH	PSH_08
SRR12280549	PSH	PSH_08
SRR12280632	PSH	PSH_08
SRR12280658	PSH	PSH_08

SRR13269122	PSH	PSH_08
SRR13269149	PSH	PSH_08
SRR13269156	PSH	PSH_08
SRR13269162	PSH	PSH_08
SRR13269163	PSH	PSH_08
SRR13269221	PSH	PSH_08
SRR13269223	PSH	PSH_08
SRR15283707	PSH	PSH_08
SRR15283763	PSH	PSH_08
SRR15283766	PSH	PSH_08
SRR15283828	PSH	PSH_08
SRR15283839	PSH	PSH_08
SRR15283850	PSH	PSH_08
SRR15283861	PSH	PSH_08
SRR15283865	PSH	PSH_08
SRR15283869	PSH	PSH_08
SRR15283894	PSH	PSH_08
VatanenT_2016__G80518__bin.7	PSH	PSH_08
VogtmannE_2016__MMRS62192467ST-2	PSH	PSH_08
WenC_2017__RA132__bin.29	PSH	PSH_08
XieH_2016__YSZC12003_35524__bin.2	PSH	PSH_08
XieH_2016__YSZC12003_37205__bin.2	PSH	PSH_08
YuJ_2015__SZAXPI015216-169__bin.42	PSH	PSH_08
ZellerG_2014__CCIS58234805ST-4-0__b	PSH	PSH_08
10317.X00180154_10317.X00180154_b	PSH	PSH_09
10317.X00182267_21	PSH	PSH_09
BackhedF_2015__SID180_4M__bin.6	PSH	PSH_09
GCA_009024655.1	PSH	PSH_09
KarlssonFH_2013__S248__bin.36	PSH	PSH_09
QinN_2014__LD-50__bin.33	PSH	PSH_09
SRR15283820	PSH	PSH_09
VatanenT_2016__G80507__bin.5	PSH	PSH_09
10317.X00214127_44_sub	PSH	PSH_10
363192044_S216_L001.3	PSH	PSH_10
363193015_S125_L001.1	PSH	PSH_10
BackhedF_2015__SID567_12M__bin.4	PSH	PSH_10
CM_madagascar__A39_02_1FE__bin.19	PSH	PSH_10
ChengpingW_2017__AS5raw__bin.14	PSH	PSH_10

GCA_003472195.1	PSI	PSH_10
GCA_024007805.1	PSI	PSH_10
KosticAD_2015__PRJNA231909.311383	PSI	PSH_10
KosticAD_2015__PRJNA231909.311383	PSI	PSH_10
MCT420_41_S49_L002	PSI	PSH_10
MCT420_6_S14_L002	PSI	PSH_10
MCT420_81_S89_L002	PSI	PSH_10
MCT420_86_S94_L002	PSI	PSH_10
SRR13269111	PSI	PSH_10
XieH_2016__YSZC12003_37207R1__bir	PSI	PSH_10
10317.X00214130_23	PSI	PSH_11
GCA_019733435.1	PSI	PSH_11
MCT420_52_S60_L002	PSI	PSH_11
MCT420_55_S63_L002	PSI	PSH_11
363192544_S35_L001	PSI	PSH_12
GCA_000601075.2	PSI	PSH_12
363192560_S51_L001	PSI	PSH_13
363193013_S123_L001	PSI	PSH_14
363193027_S137_L001	PSI	PSH_14
GCA_000273765.1	PSI	PSH_14
GCA_000598905.1	PSI	PSH_14
GCA_001699855.1	PSI	PSH_14
MCT420_77_S85_L002	PSI	PSH_14
RaymondF_2016__P22E7__bin.4	PSI	PSH_14
SRR12280498	PSI	PSH_14
SRR12280650	PSI	PSH_14
SRR13269207	PSI	PSH_14
SRR9206239	PSI	PSH_14
363193061_S171_L001	PSI	PSH_15
GCA_012844105.1	PSI	PSH_15
MCT420_63_S71_L002	PSI	PSH_15
GCA_000724795.2	PSI	PSH_16
GCA_001682215.1	PSI	PSH_17
GCA_020859785.1	PSI	PSH_17
SRR15283767	PSI	PSH_17
SRR15283868	PSI	PSH_18

TABLE 6

variant	af	filter.pvalue	lrt.pvalue	beta	variant_h2	bh_pv_adj
ATACCGCCAAG	0.256	3.24E-07	2.46E-07	-3.35E-01	2.98E-01	2.73E-03
TACGGCATACC(	0.26	6.45E-07	5.23E-07	-3.25E-01	2.90E-01	4.22E-03
AAGGTCTGGAG	2.66E-01	8.70E-06	6.86E-06	-2.94E-01	2.61E-01	2.30E-02
GCTGAAAGGTCT	2.70E-01	1.55E-05	1.24E-05	-2.85E-01	2.54E-01	3.26E-02
TGCTGCGCCAG(	2.70E-01	2.84E-07	1.08E-07	-3.44E-01	3.06E-01	1.74E-03
CCTCGAAGTGCT	2.73E-01	5.56E-07	2.46E-07	-3.33E-01	2.98E-01	2.73E-03
GAAAACCTCGA/	2.91E-01	1.15E-05	5.99E-06	-2.88E-01	2.63E-01	2.15E-02
GAACAACGACT/	3.01E-01	5.27E-06	3.29E-06	-2.90E-01	2.70E-01	1.50E-02
CATCAAGAACA,	3.08E-01	1.58E-05	1.17E-05	-2.72E-01	2.55E-01	3.17E-02
GCCAACCGCGT	2.94E-01	1.98E-05	1.09E-05	-2.78E-01	2.55E-01	3.09E-02
TATCCCTCGCA(	2.60E-01	9.46E-06	5.83E-06	-2.97E-01	2.63E-01	2.13E-02
TCTATCCCTCG(	2.84E-01	1.28E-05	6.80E-06	-2.86E-01	2.61E-01	2.30E-02
CAGGAGCGTCT	2.70E-01	1.55E-05	8.11E-06	-2.88E-01	2.59E-01	2.62E-02
GCTGGCAAGCC(	2.46E-01	1.10E-05	6.65E-06	-3.01E-01	2.61E-01	2.30E-02
GATGTATAACCG	2.80E-01	2.45E-05	1.60E-05	-2.75E-01	2.51E-01	3.94E-02
AGAGAAGGCGG	1.25E-01	3.98E-06	1.22E-06	-4.22E-01	2.81E-01	7.21E-03
TGATAGAGAAGG	1.21E-01	8.70E-06	2.87E-06	-4.14E-01	2.71E-01	1.34E-02
AGCTGGTGATG/	1.04E-01	1.31E-05	4.34E-06	-4.35E-01	2.67E-01	1.90E-02
GAACGTGAAGCT	1.00E-01	2.87E-05	1.36E-05	-4.17E-01	2.53E-01	3.42E-02
TCATGCGTTAC(	1.00E-01	4.57E-06	1.38E-06	-4.66E-01	2.79E-01	7.82E-03
CTGAACACCTGT	1.00E-01	4.57E-06	1.40E-06	-4.62E-01	2.79E-01	7.82E-03
CCTACGAGCGG	2.63E-01	1.70E-05	1.13E-05	-2.87E-01	2.55E-01	3.11E-02
GACGCCTACG/	2.60E-01	9.46E-06	6.26E-06	-2.97E-01	2.62E-01	2.22E-02
AGTATGTTTTAC	2.77E-01	1.41E-05	1.03E-05	-2.84E-01	2.56E-01	3.00E-02
AGAAGGAGAAG(	2.56E-01	5.16E-06	2.80E-06	-3.09E-01	2.72E-01	1.33E-02
CCTGAAAGGCTA	2.63E-01	1.70E-05	9.95E-06	-2.89E-01	2.57E-01	3.00E-02
TCTGCACGAGG(	1.04E-01	2.01E-06	5.77E-07	-4.69E-01	2.89E-01	4.45E-03
CCGCTATGGTCT	1.04E-01	2.01E-06	5.77E-07	-4.69E-01	2.89E-01	4.45E-03
ACGGGGGCAA(	2.15E-01	7.20E-06	5.29E-06	-3.16E-01	2.64E-01	2.04E-02
GTCTTCCGATTA	2.63E-01	1.70E-05	1.32E-05	-2.83E-01	2.53E-01	3.37E-02
ATGACACTGCT	1.00E-01	2.87E-05	1.15E-05	-4.26E-01	2.55E-01	3.14E-02

AGTCTTCCTAT	2.18E-01	1.39E-05	1.11E-05	-3.04E-01	2.55E-01	3.10E-02
TGTTCCCGGA	2.21E-01	6.94E-06	5.32E-06	-3.13E-01	2.64E-01	2.04E-02
GTTCGTGTTCC	2.66E-01	8.70E-06	5.50E-06	-2.96E-01	2.64E-01	2.04E-02
CAAGAACTTCG	2.25E-01	1.32E-05	1.10E-05	-3.01E-01	2.55E-01	3.10E-02
GGGCAGGATG	3.15E-01	4.17E-06	2.76E-06	-2.89E-01	2.72E-01	1.33E-02
TGCGGCACGG	3.18E-01	7.24E-06	5.36E-06	-2.80E-01	2.64E-01	2.04E-02
CAAGTGCGGC	3.18E-01	7.24E-06	5.52E-06	-2.79E-01	2.64E-01	2.04E-02
GAAGACGATG	2.56E-01	3.24E-07	1.84E-07	-3.39E-01	3.01E-01	2.55E-03
GATGAAGGAC	2.94E-01	4.24E-07	2.01E-07	-3.26E-01	3.00E-01	2.64E-03
GCGATGAAGG	1.28E-01	1.01E-05	5.01E-06	-3.93E-01	2.65E-01	2.04E-02
GGGCAAGGAA	1.28E-01	1.01E-05	5.01E-06	-3.93E-01	2.65E-01	2.04E-02
GGCGGGCAAG	1.59E-01	6.83E-06	3.96E-06	-3.64E-01	2.68E-01	1.78E-02
GTGCCGTCTA	2.80E-01	2.45E-05	1.36E-05	-2.82E-01	2.53E-01	3.42E-02
CGAAAAATGCG	2.42E-01	2.15E-05	1.03E-05	-2.99E-01	2.56E-01	3.00E-02
GCTGCTCGCC	1.04E-01	1.31E-05	5.33E-06	-4.31E-01	2.64E-01	2.04E-02
TGTTCGCTGAA	2.35E-01	6.29E-06	4.74E-06	-3.07E-01	2.65E-01	2.03E-02
GAAGACGTGTT	2.18E-01	1.39E-05	1.04E-05	-3.05E-01	2.56E-01	3.00E-02
AAAGAACCGGC	2.84E-01	1.28E-05	8.43E-06	-2.85E-01	2.59E-01	2.67E-02
AACTTATCTTC	1.00E-01	2.87E-05	2.03E-05	-4.12E-01	2.48E-01	4.73E-02
GCATTTGTTGC	1.07E-01	5.90E-06	2.49E-06	-4.42E-01	2.73E-01	1.26E-02
CTGAAGCATTT	1.04E-01	1.31E-05	5.88E-06	-4.33E-01	2.63E-01	2.13E-02
AGATTGTGCAG	1.00E-01	2.87E-05	1.24E-05	-4.21E-01	2.54E-01	3.26E-02
CGTGACGACC	3.74E-01	7.55E-06	5.44E-06	-2.71E-01	2.64E-01	2.04E-02
GAGCGCATCG	1.42E-01	1.25E-05	6.87E-06	-3.73E-01	2.61E-01	2.30E-02
AACGAGCGCAT	1.38E-01	5.29E-06	2.11E-06	-3.98E-01	2.75E-01	1.12E-02
CAACGAGCGC	1.56E-01	2.99E-06	8.30E-07	-3.91E-01	2.85E-01	5.67E-03
TTCAACGAGC	1.38E-01	5.29E-06	2.11E-06	-3.98E-01	2.75E-01	1.12E-02
CTGCGTAAGA	1.14E-01	4.01E-05	2.15E-05	-3.89E-01	2.47E-01	4.85E-02
TCACCACACG	4.19E-01	1.37E-05	1.02E-05	-2.59E-01	2.56E-01	3.00E-02
CGTGCCGCCT	3.53E-01	3.67E-05	2.11E-05	-2.59E-01	2.47E-01	4.85E-02
TGACGGAACAG	4.19E-01	1.37E-05	1.02E-05	-2.59E-01	2.56E-01	3.00E-02
GCAAAGGTAAC	2.28E-01	2.45E-05	2.03E-05	-2.90E-01	2.48E-01	4.73E-02
CATATCCTGCA	2.08E-01	2.85E-05	1.99E-05	-3.01E-01	2.48E-01	4.73E-02

TTCCGCCACCT	1.25E-01	2.15E-05	8.40E-06	-3.88E-01	2.59E-01	2.67E-02
TATTCCTGCAA	2.91E-01	1.15E-05	4.36E-06	-2.91E-01	2.66E-01	1.90E-02
CAGGAATACCG	2.80E-01	7.23E-06	2.82E-06	-3.00E-01	2.71E-01	1.33E-02
CTGCAAGCTG	1.00E-01	2.87E-05	1.12E-05	-4.22E-01	2.55E-01	3.10E-02
CCCGTTTTGC	2.18E-01	1.39E-05	1.06E-05	-3.05E-01	2.56E-01	3.03E-02
GAACTTAGTAA	8.27E-01	3.14E-05	2.16E-05	3.19E-01	2.47E-01	4.85E-02
AAGCTGTCGAT	1.07E-01	3.45E-05	2.23E-05	-3.90E-01	2.47E-01	4.91E-02
ATGATATAAATG	1.11E-01	1.60E-05	1.04E-05	-3.99E-01	2.56E-01	3.00E-02
TCAATCGTCTG	1.00E-01	4.57E-06	3.13E-06	-4.39E-01	2.70E-01	1.44E-02
ATATCCCATAA	3.94E-01	1.96E-05	1.22E-05	2.53E-01	2.54E-01	3.26E-02
CCATGTGTGTT	3.98E-01	1.30E-05	8.11E-06	2.58E-01	2.59E-01	2.62E-02
TGAAGTATTTG	4.08E-01	3.58E-06	2.38E-06	2.71E-01	2.73E-01	1.22E-02
AATTGTGTAAC	4.12E-01	2.29E-06	1.41E-06	2.76E-01	2.79E-01	7.82E-03
ACGAAACACAAG	4.12E-01	6.70E-07	3.93E-07	2.90E-01	2.93E-01	3.41E-03
GTTTTCGTTCC	4.05E-01	1.68E-06	1.10E-06	2.80E-01	2.82E-01	7.09E-03
ATTCATTGGAAG	4.15E-01	4.17E-07	2.11E-07	2.96E-01	3.00E-01	2.67E-03
TGGAAGAAATT	4.08E-01	1.07E-06	6.35E-07	2.85E-01	2.88E-01	4.76E-03
GAAAATGCAGC	4.15E-01	4.17E-07	2.21E-07	2.96E-01	2.99E-01	2.70E-03
GGTAAAAAAAAG	4.12E-01	4.81E-08	2.05E-08	3.19E-01	3.22E-01	1.32E-03
TAGTTTCGGAC	4.22E-01	1.58E-07	7.80E-08	3.06E-01	3.10E-01	1.32E-03
ATTGAGGGATT	4.08E-01	7.96E-08	3.19E-08	3.17E-01	3.18E-01	1.32E-03
GTATATGTCTG	4.22E-01	1.58E-07	7.80E-08	3.06E-01	3.10E-01	1.32E-03
ACCAATCAGC	4.26E-01	9.61E-08	4.96E-08	3.10E-01	3.14E-01	1.32E-03
CTGATTGGTATA	4.26E-01	9.61E-08	4.96E-08	3.10E-01	3.14E-01	1.32E-03
CACCCGGAAG	4.22E-01	1.58E-07	7.80E-08	3.06E-01	3.10E-01	1.32E-03
ATAGTGGGTAG	4.26E-01	9.61E-08	4.24E-08	3.11E-01	3.15E-01	1.32E-03
TCTTGGTATGG	4.26E-01	9.61E-08	4.24E-08	3.11E-01	3.15E-01	1.32E-03
AAAGGATCGCC	4.19E-01	6.84E-08	2.80E-08	3.16E-01	3.19E-01	1.32E-03
TCTGTCATAGA	4.22E-01	1.58E-07	6.97E-08	3.07E-01	3.11E-01	1.32E-03
TTAGTTAGCTC	4.08E-01	7.96E-08	3.47E-08	3.15E-01	3.17E-01	1.32E-03
ACACATCCGGT	4.12E-01	6.70E-07	4.63E-07	2.88E-01	2.91E-01	3.82E-03
TCGTTTTGTTG	4.01E-01	7.71E-07	4.60E-07	2.90E-01	2.91E-01	3.82E-03
ATGAGATTACAA	4.15E-01	1.13E-07	5.28E-08	3.10E-01	3.13E-01	1.32E-03

ACTATTTCAAT	3.94E-01	1.92E-06	1.21E-06	2.80E-01	2.81E-01	7.21E-03
ACAGGTTCGGA	4.12E-01	6.70E-07	3.66E-07	2.91E-01	2.94E-01	3.33E-03
ATTGAGAAATA	4.05E-01	1.68E-06	1.09E-06	2.80E-01	2.82E-01	7.09E-03
GTTTTTTCTGT	4.22E-01	1.58E-07	7.00E-08	3.06E-01	3.11E-01	1.32E-03
TAACGGAAACG	4.12E-01	6.70E-07	2.96E-07	2.93E-01	2.96E-01	3.00E-03
TGAAAGTGTTG	4.22E-01	1.58E-07	7.00E-08	3.06E-01	3.11E-01	1.32E-03
TTGAATTGCAC	4.05E-01	1.31E-07	4.84E-08	3.13E-01	3.14E-01	1.32E-03
CTTCTCAAAAA	4.05E-01	1.31E-07	4.84E-08	3.13E-01	3.14E-01	1.32E-03
TTTTTAACTTG	4.08E-01	1.07E-06	6.84E-07	2.85E-01	2.87E-01	4.85E-03
TCCGGAAAAAG	4.05E-01	4.83E-07	2.78E-07	2.95E-01	2.97E-01	2.90E-03
TTATGGGTTTT	4.19E-01	6.84E-08	2.63E-08	3.17E-01	3.20E-01	1.32E-03
GGTTCTTAATTG	4.26E-01	3.56E-07	1.87E-07	2.96E-01	3.01E-01	2.55E-03
TGGCTTGTCGT	4.22E-01	1.58E-07	7.60E-08	3.06E-01	3.10E-01	1.32E-03
TGTCGTTTTAC	4.22E-01	1.58E-07	7.60E-08	3.06E-01	3.10E-01	1.32E-03
CCCCTACATTA	4.19E-01	2.58E-07	1.28E-07	3.01E-01	3.05E-01	1.97E-03
AGCTCTTTTTG	4.22E-01	5.75E-07	2.64E-07	2.93E-01	2.97E-01	2.84E-03
TCTCTGTTTCC	4.12E-01	1.85E-07	7.83E-08	3.07E-01	3.09E-01	1.32E-03
CTGAAGAAACT	4.19E-01	9.19E-07	3.94E-07	2.90E-01	2.93E-01	3.41E-03
ATCTCCGCCAT	4.15E-01	1.46E-06	6.56E-07	2.84E-01	2.88E-01	4.76E-03
GGCCGTCCGT	4.19E-01	9.19E-07	3.64E-07	2.90E-01	2.94E-01	3.33E-03
ATAGTTCGGTT	4.26E-01	3.56E-07	1.43E-07	2.99E-01	3.03E-01	2.11E-03
GCCAAGTCTTG	4.12E-01	2.29E-06	9.71E-07	2.81E-01	2.83E-01	6.50E-03
TTCTTAAGTTG	3.91E-01	2.94E-05	1.57E-05	2.51E-01	2.51E-01	3.89E-02
CGGACGGGAT	3.67E-01	1.83E-05	9.85E-06	2.59E-01	2.57E-01	3.00E-02
ATTCAAATCTGA	3.81E-01	3.31E-05	2.21E-05	2.49E-01	2.47E-01	4.90E-02
TAAGGAACTTG	3.91E-01	2.94E-05	2.21E-05	2.47E-01	2.47E-01	4.90E-02
CACCGGAGAT	3.63E-01	2.75E-05	2.04E-05	2.51E-01	2.48E-01	4.73E-02
CCGGAGTGAAT	3.67E-01	1.83E-05	1.32E-05	2.56E-01	2.53E-01	3.37E-02
TGTTTTTTTTGC	3.60E-01	1.34E-05	1.04E-05	2.60E-01	2.56E-01	3.00E-02
TACGGGAGAGT	3.63E-01	2.75E-05	2.13E-05	2.51E-01	2.47E-01	4.85E-02
CTTTGTCTGTT	3.74E-01	2.48E-05	2.14E-05	2.50E-01	2.47E-01	4.85E-02
CGCAGCCGGG	3.84E-01	2.17E-06	1.35E-06	2.81E-01	2.80E-01	7.82E-03
AATGGGGGCGA	3.67E-01	1.83E-05	1.26E-05	2.57E-01	2.54E-01	3.29E-02

CTAGTTTATCT/	3.91E-01	9.67E-06	6.55E-06	2.62E-01	2.62E-01	2.30E-02
TCCCTGCCGTG	2.94E-01	9.11E-06	7.88E-06	2.76E-01	2.59E-01	2.59E-02
AGATTTGGATTA	4.26E-01	3.39E-05	2.03E-05	-2.52E-01	2.48E-01	4.73E-02
CTTACCTTTGG	8.51E-01	2.73E-06	1.80E-06	3.99E-01	2.77E-01	9.82E-03
TTATCGCCCCA	8.86E-01	1.16E-06	1.21E-06	4.49E-01	2.81E-01	7.21E-03
GGGGCGATAAT	8.86E-01	1.16E-06	1.21E-06	4.49E-01	2.81E-01	7.21E-03
GGGCGATAATT	8.86E-01	1.16E-06	1.21E-06	4.49E-01	2.81E-01	7.21E-03
ATAAATTATCGC	8.93E-01	8.73E-07	8.31E-07	4.69E-01	2.85E-01	5.67E-03
CGATAATTTAT	8.89E-01	3.76E-07	3.16E-07	4.79E-01	2.95E-01	3.11E-03
TTTATCTATTAA	8.96E-01	2.01E-06	2.31E-06	4.59E-01	2.74E-01	1.21E-02
CCCTTTAAATCA	9.00E-01	4.57E-06	5.32E-06	4.50E-01	2.64E-01	2.04E-02
AATCATATACGT	9.00E-01	4.57E-06	5.32E-06	4.50E-01	2.64E-01	2.04E-02
CATATACGTATG	9.00E-01	4.57E-06	5.32E-06	4.50E-01	2.64E-01	2.04E-02
GACGATAGGGA	7.96E-01	1.59E-05	9.75E-06	-3.20E-01	2.57E-01	3.00E-02
GACGATAGGGA	7.96E-01	1.59E-05	9.75E-06	-3.20E-01	2.57E-01	3.00E-02
AGGGACGGTCA	8.03E-01	9.24E-06	4.87E-06	-3.35E-01	2.65E-01	2.04E-02
GACGGTCACC/	8.34E-01	2.33E-05	1.43E-05	-3.41E-01	2.52E-01	3.57E-02
CACCTTCACGA	8.03E-01	9.24E-06	4.39E-06	-3.36E-01	2.66E-01	1.90E-02
CACCTTCACGA	8.03E-01	9.24E-06	4.39E-06	-3.36E-01	2.66E-01	1.90E-02
AGCTCAGCTGG	7.89E-01	2.63E-05	1.02E-05	-3.07E-01	2.56E-01	3.00E-02
AGCTCAGCTGG	7.89E-01	2.63E-05	1.02E-05	-3.07E-01	2.56E-01	3.00E-02
AGCTCAGCTGG	7.89E-01	2.63E-05	1.02E-05	-3.07E-01	2.56E-01	3.00E-02
AATTTTGCTATA	7.79E-01	7.58E-06	2.75E-06	-3.22E-01	2.72E-01	1.33E-02
CATTGAAAAGA	7.79E-01	7.58E-06	2.75E-06	-3.22E-01	2.72E-01	1.33E-02
ATCCGTCAACG	7.85E-01	1.75E-05	7.86E-06	-3.09E-01	2.59E-01	2.59E-02
ATCCGTCAACG	7.85E-01	1.75E-05	7.86E-06	-3.09E-01	2.59E-01	2.59E-02
ATCCGTCAACG	7.85E-01	1.75E-05	7.86E-06	-3.09E-01	2.59E-01	2.59E-02
TCGAATCCGTC	8.20E-01	9.14E-07	2.40E-07	-3.80E-01	2.98E-01	2.73E-03
TCGAATCCGTC	8.20E-01	9.14E-07	2.40E-07	-3.80E-01	2.98E-01	2.73E-03
TCGAATCCGTC	8.20E-01	9.14E-07	2.40E-07	-3.80E-01	2.98E-01	2.73E-03
GCACGGAGACT	7.99E-01	2.39E-05	1.95E-05	-3.03E-01	2.48E-01	4.70E-02
TGTATATTTTGC	8.03E-01	9.24E-06	6.83E-06	-3.20E-01	2.61E-01	2.30E-02
GACGACCTTGT/	1.11E-01	1.60E-05	1.31E-05	-3.93E-01	2.53E-01	3.37E-02

AGTTCCCCCGA	1.25E-01	3.76E-05	1.77E-05	3.67E-01	2.50E-01	4.30E-02
CAAGCCCATGC	1.25E-01	3.76E-05	1.77E-05	3.67E-01	2.50E-01	4.30E-02

gene	Name	Cluster	Product	Description	PFAMs
3192529_S12_L001_07145		Group1	nserved C-terminal do	se C-terminal domain	e_C,N6_Mtase,ResIII,SNF2_N
3192529_S12_L001_07145		Group1	nserved C-terminal do	se C-terminal domain	e_C,N6_Mtase,ResIII,SNF2_N
3192529_S12_L001_07145		Group1	nserved C-terminal do	se C-terminal domain	e_C,N6_Mtase,ResIII,SNF2_N
3192529_S12_L001_07145		Group1	nserved C-terminal do	se C-terminal domain	e_C,N6_Mtase,ResIII,SNF2_N
3192529_S12_L001_07145		Group1	nserved C-terminal do	se C-terminal domain	e_C,N6_Mtase,ResIII,SNF2_N
3192529_S12_L001_07145		Group1	nserved C-terminal do	se C-terminal domain	e_C,N6_Mtase,ResIII,SNF2_N
3192529_S12_L001_07145		Group1	nserved C-terminal do	se C-terminal domain	e_C,N6_Mtase,ResIII,SNF2_N
3192529_S12_L001_07145		Group1	nserved C-terminal do	se C-terminal domain	e_C,N6_Mtase,ResIII,SNF2_N
3192529_S12_L001_07145		Group1	nserved C-terminal do	se C-terminal domain	e_C,N6_Mtase,ResIII,SNF2_N
3192529_S12_L001_07145		Group1	nserved C-terminal do	se C-terminal domain	e_C,N6_Mtase,ResIII,SNF2_N
3192529_S12_L001_07145		Group1	nserved C-terminal do	se C-terminal domain	e_C,N6_Mtase,ResIII,SNF2_N
3192529_S12_L001_07145		Group1	nserved C-terminal do	se C-terminal domain	e_C,N6_Mtase,ResIII,SNF2_N
3192529_S12_L001_07145		Group1	nserved C-terminal do	se C-terminal domain	e_C,N6_Mtase,ResIII,SNF2_N
3192529_S12_L001_07145		Group1	nserved C-terminal do	se C-terminal domain	e_C,N6_Mtase,ResIII,SNF2_N
3192529_S12_L001_07145		Group1	nserved C-terminal do	se C-terminal domain	e_C,N6_Mtase,ResIII,SNF2_N
3192529_S12_L001_07145		Group1	nserved C-terminal do	se C-terminal domain	e_C,N6_Mtase,ResIII,SNF2_N
3192529_S12_L001_07145		Group1	nserved C-terminal do	se C-terminal domain	e_C,N6_Mtase,ResIII,SNF2_N
3192529_S12_L001_07145		Group1	nserved C-terminal do	se C-terminal domain	e_C,N6_Mtase,ResIII,SNF2_N
3192529_S12_L001_07145		Group1	DNA methylase	se C-terminal domain	ase_C,N6_Mtase,SNF2_N
3192529_S12_L001_07145		Group1	nserved C-terminal do	se C-terminal domain	e_C,N6_Mtase,ResIII,SNF2_N
3192529_S12_L001_07145		Group1	nserved C-terminal do	se C-terminal domain	e_C,N6_Mtase,ResIII,SNF2_N
3192529_S12_L001_07145		Group1	nserved C-terminal do	se C-terminal domain	e_C,N6_Mtase,ResIII,SNF2_N
3192529_S12_L001_07145		Group1	nserved C-terminal do	se C-terminal domain	e_C,N6_Mtase,ResIII,SNF2_N
3192529_S12_L001_07145		Group1	nserved C-terminal do	se C-terminal domain	e_C,N6_Mtase,ResIII,SNF2_N
3192529_S12_L001_07145		Group1	nserved C-terminal do	se C-terminal domain	e_C,N6_Mtase,ResIII,SNF2_N
3192529_S12_L001_07145		Group1	nserved C-terminal do	se C-terminal domain	e_C,N6_Mtase,ResIII,SNF2_N
3192529_S12_L001_07145		Group1	nserved C-terminal do	se C-terminal domain	e_C,N6_Mtase,ResIII,SNF2_N
3192529_S12_L001_07145		Group1	nserved C-terminal do	se C-terminal domain	e_C,N6_Mtase,ResIII,SNF2_N
3192529_S12_L001_07150		Group1	hypothetical protein	cation Cytoplasmic, sc	DUF1896
3192529_S12_L001_07150		Group1	hypothetical protein	cation Cytoplasmic, sc	DUF1896
3192529_S12_L001_07150		Group1	hypothetical protein	cation Cytoplasmic, sc	DUF1896

topB	Group1	DNA topoisomerase I	G0550 Topoisomeras	om_bac,Toprim,Toprim_Crpt
topB	Group1	DNA topoisomerase I	G0550 Topoisomeras	om_bac,Toprim,Toprim_Crpt
topB	Group1	DNA topoisomerase I	G0550 Topoisomeras	om_bac,Toprim,Toprim_Crpt
topB	Group1	DNA topoisomerase I	G0550 Topoisomeras	om_bac,Toprim,Toprim_Crpt
topB	Group1	DNA topoisomerase I	G0550 Topoisomeras	om_bac,Toprim,Toprim_Crpt
topB	Group1	DNA topoisomerase III	topoisomeraes I ATP-	om_bac,Toprim,Toprim_Crpt
topB	Group1	DNA topoisomerase I	G0550 Topoisomeras	om_bac,Toprim,Toprim_Crpt
topB	Group1	DNA topoisomerase I	G0550 Topoisomeras	om_bac,Toprim,Toprim_Crpt
topB	Group1	DNA topoisomerase I	G0550 Topoisomeras	om_bac,Toprim,Toprim_Crpt
topB	Group1	DNA topoisomerase I	G0550 Topoisomeras	om_bac,Toprim,Toprim_Crpt
topB	Group1	DNA topoisomerase I	G0550 Topoisomeras	om_bac,Toprim,Toprim_Crpt
topB	Group1	DNA topoisomerase I	G0550 Topoisomeras	om_bac,Toprim,Toprim_Crpt
topB	Group1	DNA topoisomerase I	G0550 Topoisomeras	om_bac,Toprim,Toprim_Crpt
topB	Group1	DNA topoisomerase I	G0550 Topoisomeras	om_bac,Toprim,Toprim_Crpt
topB	Group1	DNA topoisomerase I	G0550 Topoisomeras	om_bac,Toprim,Toprim_Crpt
topB	Group1	DNA topoisomerase I	G0550 Topoisomeras	om_bac,Toprim,Toprim_Crpt
topB	Group1	DNA topoisomerase I	G0550 Topoisomeras	om_bac,Toprim,Toprim_Crpt
topB	Group1	DNA topoisomerase I	G0550 Topoisomeras	om_bac,Toprim,Toprim_Crpt
topB	Group1	DNA topoisomerase I	G0550 Topoisomeras	om_bac,Toprim,Toprim_Crpt
3192529_S12_L001_17745	Group1	)-C domain-containing	e IV secretory pathwa	NA_transf,TraG-D_C,YWFCY
3192529_S12_L001_17745	Group1	)-C domain-containing	e IV secretory pathwa	NA_transf,TraG-D_C,YWFCY
3192529_S12_L001_17745	Group1	)-C domain-containing	e IV secretory pathwa	NA_transf,TraG-D_C,YWFCY
3192529_S12_L001_07160	Group1	hypothetical protein	-	-
3192529_S12_L001_17745	Group1	jugal transfer protein T	e IV secretory pathwa	NA_transf,TraG-D_C,YWFCY
3192529_S12_L001_17745	Group1	jugal transfer protein T	e IV secretory pathwa	NA_transf,TraG-D_C,YWFCY
3192529_S12_L001_17745	Group1	jugal transfer protein T	e IV secretory pathwa	NA_transf,TraG-D_C,YWFCY
3192529_S12_L001_17745	Group1	jugal transfer protein T	e IV secretory pathwa	NA_transf,TraG-D_C,YWFCY
3192529_S12_L001_17745	Group1	y, VirD4 component, T	e IV secretory pathwa	sf,TraG-D_C,TrwB_AAD_bind,YWFCY
3192529_S12_L001_07170	Group1	hypothetical protein	-	-
3192529_S12_L001_07170	Group1	hypothetical protein	-	-
3192529_S12_L001_07170	Group1	hypothetical protein	-	-
3192529_S12_L001_07170	Group1	hypothetical protein	-	-
3192529_S12_L001_07180	Group1	DNA-binding protein	Helix-turn-helix domai	HTH_17

3192529_S12_L001_07180	Group1	DNA-binding protein	Helix-turn-helix domain	HTH_17
3192529_S12_L001_07180	Group1	DNA-binding protein	Helix-turn-helix domain	HTH_17
3192529_S12_L001_07180	Group1	DNA-binding protein	ding domain, excisiona	HTH_17
xerD	Group1	NA	NA	NA
xerD	Group1	NA	NA	NA
3192529_S12_L001_15570	Group8	NA	NA	NA
NA	Group1	conjugal transfer protei	nction, B. Theta Gene c	DUF3872
NA	Group1	conjugal transfer protei	nction, B. Theta Gene c	DUF3872
NA	Group1	conjugal transfer protei	nction, B. Theta Gene c	DUF3872
3192529_S12_L001_22560	Group2	Replication protein	-	-
3192529_S12_L001_22560	Group2	Replication protein	-	-
3192529_S12_L001_22560	Group2	Replication protein	-	-
3192529_S12_L001_22560	Group2	Replication protein	-	-
3192529_S12_L001_22560	Group2	Replication protein	-	-
3192529_S12_L001_22560	Group2	Replication protein	-	-
3192529_S12_L001_22560	Group2	Replication protein	-	-
3192529_S12_L001_22560	Group2	Replication protein	-	-
3192529_S12_L001_22560	Group2	Replication protein	-	-
3192529_S12_L001_22560	Group2	Replication protein	-	-
3192529_S12_L001_22560	Group2	Replication protein	-	-
3192529_S12_L001_22560	Group2	Replication protein	-	-
3192529_S12_L001_22560	Group2	Replication protein	-	-
3192529_S12_L001_22560	Group2	Replication protein	-	-
3192529_S12_L001_22560	Group2	Replication protein	-	-
3192529_S12_L001_22560	Group2	Replication protein	-	-
.001_22560|363192529_S12_L001_22565	Group2	Replication protein	-	-
.001_22560|363192529_S12_L001_22565	Group2	hypothetical protein	-	-
3192529_S12_L001_22565	Group2	hypothetical protein	-	-
3192529_S12_L001_22565	Group2	hypothetical protein	-	-
3192529_S12_L001_22565	Group2	hypothetical protein	-	-
.001_22565|363192529_S12_L001_22570	Group2	hypothetical protein	-	-
3192529_S12_L001_22570	Group2	hypothetical protein	-	-
3192529_S12_L001_22570	Group2	hypothetical protein	-	-

3192529_S12_L001_22570	Group2	hypothetical protein	-	-
3192529_S12_L001_22570	Group2	hypothetical protein	-	-
3192529_S12_L001_22570	Group2	hypothetical protein	-	-
3192529_S12_L001_22570	Group2	hypothetical protein	-	-
3192529_S12_L001_22570	Group2	hypothetical protein	-	-
3192529_S12_L001_22570	Group2	hypothetical protein	-	-
3192529_S12_L001_22570	Group2	hypothetical protein	-	-
3192529_S12_L001_22570	Group2	hypothetical protein	-	-
3192529_S12_L001_22575	Group2	Mob421 protein	cation Cytoplasmic, sc	-
3192529_S12_L001_22575	Group2	Mob421 protein	cation Cytoplasmic, sc	-
3192529_S12_L001_22575	Group2	Mob421 protein	cation Cytoplasmic, sc	-
3192529_S12_L001_22575	Group2	Mob421 protein	cation Cytoplasmic, sc	-
3192529_S12_L001_22575	Group2	Mob421 protein	cation Cytoplasmic, sc	-
3192529_S12_L001_22575	Group2	Mob421 protein	cation Cytoplasmic, sc	-
3192529_S12_L001_22575	Group2	Mob421 protein	cation Cytoplasmic, sc	-
3192529_S12_L001_22575	Group2	Mob421 protein	cation Cytoplasmic, sc	-
3192529_S12_L001_22575	Group2	Mob421 protein	cation Cytoplasmic, sc	-
3192529_S12_L001_22575	Group2	Mob421 protein	cation Cytoplasmic, sc	-
3192529_S12_L001_22575	Group2	Mob421 protein	cation Cytoplasmic, sc	-
3192529_S12_L001_22575	Group2	Mob421 protein	cation Cytoplasmic, sc	-
3192529_S12_L001_22575	Group2	Mob421 protein	cation Cytoplasmic, sc	-
3192529_S12_L001_22575	Group2	Mob421 protein	cation Cytoplasmic, sc	-
3192529_S12_L001_22575	Group2	Mob421 protein	cation Cytoplasmic, sc	-
3192529_S12_L001_22575	Group2	Mob421 protein	cation Cytoplasmic, sc	-
3192529_S12_L001_22585	Group2	growth inhibitor PemK	nent of a toxin-antitoxir	PemK_toxin
3192529_S12_L001_22590	Group2	ly-alpha-D-galacturon	cation Cytoplasmic, sc	-
3192529_S12_L001_22595	Group2	hypothetical protein		
3192529_S12_L001_22595	Group2	hypothetical protein		
3192529_S12_L001_22595	Group2	hypothetical protein		
3192529_S12_L001_22595	Group2	hypothetical protein		
3192529_S12_L001_22595	Group2	hypothetical protein		
3192529_S12_L001_22595	Group2	hypothetical protein		
3192529_S12_L001_22595	Group2	hypothetical protein		

3192529_S12_L001_22595		Group2	hypothetical protein		
NA		Group2	AbrB domain-containin	tional regulator antitox	MazE_antitoxin
85.1_07235|GCA_0000	susC	Group3	NA	NA	NA
updY		Group4	NA	NA	NA
updY		Group4	NA	NA	NA
updY		Group4	NA	NA	NA
updY		Group4	NA	NA	NA
updY		Group4	NA	NA	NA
updY		Group4	NA	NA	NA
updY		Group4	NA	NA	NA
CA_000025985.1_18510		Group4	NA	NA	NA
CA_000025985.1_18510		Group4	NA	NA	NA
CA_000025985.1_18510		Group4	NA	NA	NA
CA_000025985.1_1896	rluB	Group9	NA	NA	NA
CA_000025985.1_1896	rluB	Group9	NA	NA	NA
CA_000025985.1_1896	rluB	Group9	NA	NA	NA
CA_000025985.1_1896	rluB	Group9	NA	NA	NA
CA_000025985.1_1896	rluB	Group9	NA	NA	NA
CA_000025985.1_1896	rluB	Group9	NA	NA	NA
CA_000025985.1_1897	GH35	Group9	NA	NA	NA
CA_000025985.1_1897	GH35	Group9	NA	NA	NA
CA_000025985.1_1897	GH35	Group9	NA	NA	NA
CA_000025985.1_1897	GH35	Group9	NA	NA	NA
CA_000025985.1_1897	GH35	Group9	NA	NA	NA
CA_000025985.1_1897	GH35	Group9	NA	NA	NA
CA_000025985.1_1897	GH35	Group9	NA	NA	NA
CA_000025985.1_1897	GH35	Group9	NA	NA	NA
CA_000025985.1_1897	GH35	Group9	NA	NA	NA
CA_000025985.1_1897	GH35	Group9	NA	NA	NA
CA_000025985.1_1897	GH35	Group9	NA	NA	NA
gyrA	gyrA	Group5	NA	NA	NA
gyrA	gyrA	Group5	NA	NA	NA
NA	ardA	Group6	tirestriction protein Ar	antirestriction protein	ArdA

NA	Group7	mobilization protein	obilization nuclease do	Relaxase
NA	Group7	mobilization protein	obilization nuclease do	Relaxase

TABLE 7 Primers for species-specific qPCR

Oligonucleotides

Target	Primer name	Forward	Reverse
hsp60	B. fraghsp60	GATCGTGGTTATCTGTCA	CTGTCTACATCTTCTGCA
hsp60	B. thetasp60	GTTGGTTATCGCAGAAGA	GCACAAATCTTCAGCTGA
hsp60	B. fraghsp60	GATCGTGGTTATCTGTCA	CTGTCTACATCTTCTGCA
hsp60	B. vulghsp60	CTGGCTCAATCAATCGTA	GGATCGTTATTGGCAGAA
SSR2	mpi	CGTATTTGAATGAATAGCCGTTTT	TGTATTCTGCGGTGAATTATGACT
16S rRNA	27F / 1492R	AGAGTTTGATCMTGGCTCAG	GGTTACCTTGTTACGACTT
