# Supplementary material for: Comparative genomic analysis of Bacteroides fragilis from intestinal and extra-intestinal sites
Source: Microbiol Spectr. 2026 Jul 14;14(8):e00306-26. doi: 10.1128/spectrum.00306-26 (PMC13435655; doi:10.1128/spectrum.00306-26)
Supplement: Supplementary file 2 [file spectrum.00306-26-s0005.docx]

**Supplemental Figure 1. COG annotation coverage and source-specific functional biases**

**A)** Fraction of genes with an assigned COG category (“Known”) versus lacking an assignment (“Unknown”) stratified by core, accessory, and unique gene bins across the *B. fragilis* pangenome (664 genomes). Percent labels indicate within-bin proportions.

**B)** Left (heatmap): Observed/Expected (O/E) proportion of genes per COG in the core and accessory genome across all isolates; asterisks mark core-accessory differences by Fisher’s exact test with BH correction (*padj≤0.05, **≤0.01, ***≤0.001). Right (points): For each COG, log2 odds ratio comparing the accessory to core ratio in extra-intestinal (E) versus intestinal (C), with Haldane–Anscombe correction and 95% CIs from Fisher’s exact test. Positive values indicate a higher accessory bias in E; negative values indicate a higher core bias in E (relative to C). (MAGs excluded)

**Supplemental Figure 2. *B. fragilis* isolation source distribution among phylogroups and polysaccharide (PS) operon-path traits**

**A)** Cluster selection. Elbow (within-SS) and mean silhouette across k; k=17 was chosen based on the elbow and a local maximum in silhouette.

**B)** Silhouette scores per sample for the 17 phylogroups (points colored by cluster; dashed line at 0).

**C)** Genome size of phylogroups 1-17. No statistically significant phylogroups by Welch’s t-test at a corrected p-value of 0.05.

**D)** Distribution of *B. fragilis* isolates by isolation source across phylogroups displaying both absolute count and percentage. No statistically significant difference between isolation sources per phylogroup after chi-squared test with correction for multiple hypothesis testing.

**E)** Grouped bar plot summarizing the strength of association between polysaccharide (PS) operon paths and categorical factors across distinct PS groups. For each PS group on the y-axis, two bars represent the –log₁₀(p)-values obtained from Chi-square tests: intestinal vs. extra-intestinal and the association between PS paths and phylogroups.

**F)** Core-genome phylogeny. The colored tracks beneath the tree show PS operon paths per isolate. Colors are categorical and consistently map each PS path to a color; within each phylogroup, the dominant color reflects the PS path most common/similar in that group.

**G)** Core-genome phylogeny colored by phylogroup with color matching to phylogroup colors in B.

**Supplemental Figure 3. Polysaccharide operon structures among *B. fragilis* strains**

PSB-H operon structure per all high-quality isolates in the *B. fragilis* pangenome (n=262). Genes are annotated with gene names, if annotated by bakta. Colored by COG category, annotated with information on select strains profiled throughout the study.

**Supplemental Figure 4. Growth phenotypes and mGWAS**

**A)** Growth summary metrics by isolation source. Per-strain estimates of area under the curve (AUC), maximum density, logistic rate (k), time at mid-point (t_mid), and end-point AUC (auc_e) for intestinal (I) vs extra-intestinal (E) isolates. (ns)

**B)** QQ plot for the unitig mixed-model GWAS testing association with isolation source. Shown are observed versus expected –log10(p) values; genomic control inflation factor λGC = 1.227 (n = 354,732 tests).

**C)** Core-genome phylogeny with tip points colored by isolation source (I, navy; E, rose). Tracks beneath the tree mark presence/absence of the GWAS-associated unitig groups.

**SUPPLMENTAL TABLES**

**Table 1.** Newly sequenced Chu lab strains metadata

Includes sequence identifier, strain names, cohort, host metadata, and location of isolation.

**Table 2.** Accession identifiers for all public *Bacteroides fragilis* strains used in this study

Includes sequence identifier and source.

**Table 3.** Phylogroup designation for each strain

Details the sequence identifier with the assigned phylogroup for every strain.

**Table 4.** Quality information on all isolates analyzed

For each sequence identifier, GC, genome size, contig number, longest contig, mean contig length, coding density, translation table, and predicted genes.

**Table 5.** Designation of capsular polysaccharide paths per isolate

Includes sequence identifier, capsular polysaccharide group (A-H), and path assignment.

**Table 6.** Annotation of all genes identified through mGWAS as significantly associated with isolation source

For each significant unitig, as designated through mGWAS, the allele frequency, p-value, phylogeny-corrected p-value, effect sizes, adjusted pvalue, gene, assigned genomic cluster, and annotation per gene.

**Table 7.** Primers for species-specific qPCR

All primers used in this publication for species-identification.
